# Students' mental health during the pandemic: results of the observational cross-sectional COVID-19 MEntal health inTernational for university Students (COMET-S) study

**DOI:** 10.3389/fpsyt.2023.1320156

**Published:** 2024-01-11

**Authors:** Konstantinos N. Fountoulakis, Nor Aziah Alias, Sarah Bjedov, Nikolaos K. Fountoulakis, Xenia Gonda, Jan Hilbig, Miro Jakovljević, Barbara Kulig, Girija Mahale, Alexandros Manafis, Muftau Mohammed, Ilia Nadareishvili, Alvydas Navickas, Mikaella E. Patsali, Alexey Pavlichenko, Sindija Mairita Pilaga, Salmi Razali, Dmitry Romanov, Iakimova Rossitza, Auwal Sani Salihu, Ana Sinauridze, Maria Stoyanova, Ketaki Thosar, Julija Vorobjova, Jelena Vrublevska, Elmars Rancans, Afzal Javed, Pavlos N. Theodorakis, Joao Breda, Daria Smirnova

**Affiliations:** ^1^3rd Department of Psychiatry, School of Medicine, Aristotle University of Thessaloniki Greece, Thessaloniki, Greece; ^2^WHO Collaboration Center for Quality in Mental Health, Aristotle University of Thessaloniki, Thessaloniki, Greece; ^3^Academic Affairs Division, Faculty of Education, Universiti Teknologi MARA, Sungai Buloh, Selangor, Malaysia; ^4^Department of Psychiatry and Psychological Medicine, University Hospital Centre Zagreb, Zagreb, Croatia; ^5^Faculty of Medicine, Medical University of Sofia, Sofia, Bulgaria; ^6^Department of Psychiatry and Psychotherapy, Semmelweis University, Budapest, Hungary; ^7^Clinic of Psychiatry, Medical Faculty, Institute of Clinical Medicine, Vilnius University, Vilnius, Lithuania; ^8^School of Medicine, University of Zagreb, Zagreb, Croatia; ^9^Department of Clinical Psychology, Semmelweis University, Budapest, Hungary; ^10^Symbiosis Centre for Emotional Wellbeing, Symbiosis International (Deemed) University, Pune, India; ^11^Faculty of Medicine, School of Health Sciences, Aristotle University of Thessaloniki, Thessaloniki, Greece; ^12^Department of Mental Health, University of Abuja Teaching Hospital, Abuja, Nigeria; ^13^David Tvildiani Medical University, Tbilisi, Georgia; ^14^School of Social Sciences, Hellenic Open University, Patras, Greece; ^15^Department of Internal Medicine, Nicosia General Hospital, Nicosia, Cyprus; ^16^Education Center, Mental-Health Clinic No. 1 n.a. N.A. Alexeev of Moscow Healthcare Department, Moscow, Russia; ^17^Faculty of Medicine, Riga Stradins University, Riga, Latvia; ^18^Department of Psychiatry, Faculty of Medicine, Universiti Teknologi MARA, Sungai Buloh, Selangor, Malaysia; ^19^Department of Psychiatry, Samara State Medical University, Samara, Russia; ^20^Second Psychiatric Clinic, University Hospital for Active Treatment in Neurology and Psychiatry “Saint Naum”, Sofia, Bulgaria; ^21^Department of Psychiatry, Bayero University, Kano, Nigeria; ^22^Aminu Kano Teaching Hospital, Kano, Nigeria; ^23^Georgian Medical Students' Association, Tbilisi, Georgia; ^24^Department of Psychiatry and Narcology, Riga Stradins University, Riga, Latvia; ^25^Institute of Public Health, Riga Stradins University, Riga, Latvia; ^26^Riga Centre of Psychiatry and Narcology, Riga, Latvia; ^27^Institute of Applied Health Research, University of Birmingham, Birmingham, United Kingdom; ^28^Warwick Medical School, University of Warwick, Coventry, United Kingdom; ^29^Pakistan Psychiatric Research Centre, Fountain House, Lahore, Pakistan; ^30^Health Policy, WHO Regional Office for Europe, Copenhagen, Denmark; ^31^WHO Athens Quality of Care Office, WHO Regional Office for Europe, Copenhagen, Denmark; ^32^International Centre for Education and Research in Neuropsychiatry, Samara State Medical University, Samara, Russia

**Keywords:** depression, university students, mental health, COVID-19, suicidality, conspiracy theories

## Abstract

**Introduction:**

The aim of the study was to search rates of depression and mental health in university students, during the COVID-19 pandemic.

**Materials and methods:**

This is an observational cross-sectional study. A protocol gathering sociodemographic variables as well as depression, anxiety and suicidality and conspiracism was assembled, and data were collected anonymously and online from April 2020 through March 2021. The sample included 12,488 subjects from 11 countries, of whom 9,026 were females (72.2%; aged 21.11 ± 2.53), 3,329 males (26.65%; aged 21.61 ± 2.81) and 133 “non-binary gender” (1.06%; aged 21.02 ± 2.98). The analysis included chi-square tests, correlation analysis, ANCOVA, multiple forward stepwise linear regression analysis and Relative Risk ratios.

**Results:**

Dysphoria was present in 15.66% and probable depression in 25.81% of the total study sample. More than half reported increase in anxiety and depression and 6.34% in suicidality, while lifestyle changes were significant. The model developed explained 18.4% of the development of depression. Believing in conspiracy theories manifested a complex effect. Close to 25% was believing that the vaccines include a chip and almost 40% suggested that facemask wearing could be a method of socio-political control. Conspiracism was related to current depression but not to history of mental disorders.

**Discussion:**

The current study reports that students are at high risk for depression during the COVID-19 pandemic and identified specific risk factors. It also suggested a role of believing in conspiracy theories. Further research is important, as it is targeted intervention in students' groups that are vulnerable both concerning mental health and conspiracism.

## Introduction

University students' mental health constitutes an area of special interest. This group is considered to belong to vulnerable groups and one reason is young age (1) but also the fact that any disruption during of the studies has deep long-term effects, plus that their personality is not mature enough to deal with additional stress (2, 3).

On top of these, the COVID-19 outbreak caused significant disruption in university studies, resulting in an enduring change in the academic environment, which is expected to lead to the emergence of feelings of fear and worry in the students population. The above should be considered in the frame of an extreme threat to the community as well as the individual. Additionally, expecting an economic crisis resulting in future unemployment, in combination with changes at present in social behavior, routine and daily habits, impose further stress.

Even during the pre-pandemic period, reports were suggesting that the rate of probable depression among university students is >20% while suicidal thoughts are also unexpectedly high and above 10% (1, 4). Concerning the mental health of students during the pandemic, a lot of published empirical data exist (5–11), but the literature is also overwhelmed with papers reflecting opinions or viewpoints and perspectives, including narrations as well as guidelines of how to cope with the pandemic. All utilize previous experience from pandemics of the past and also utilize common sense. The result is that they often obscure rather than clarify matters. Within the scope of precision and personalized psychiatry, an important goal is to identify specific variables and their exact contribution, including the belief in conspiracy theories which has been shown to exert a complex effect on mental health during the pandemic (7, 12, 13).

A recent meta-analysis of ~1.5 million students altogether (6) reported that the prevalence of anxiety was 32%, of depressive symptoms was 34%, and of sleep disturbances was 33%. These authors concluded that their results are indicative of an increase in these symptoms during the pandemic, despite of the similar findings by meta-analysis of data from before the pandemic (14). Deng et al. (6) argued that their findings should be considered as reflecting an increase because they were based mainly on studies on Chinese students, who are expected to manifest lower baseline rates of these symptoms. A second smaller meta-analysis reported similar results (5, 7).

The aim of current study was to calculate the rate of probable depression and its determinants in the population of university students in eleven countries Bulgaria, Croatia, Georgia, Greece, Hungary, India, Latvia, Lithuania, Malaysia, Nigeria and Russia during the COVID-19 outbreak. Secondary aims were to investigate the changes in distress, anxiety, and suicidal ideation as well as the role of conspiracism. The paper conforms with the STROBE statement for the reporting of observational studies and the respected checklist is included in the Webappendix (15).

## Material and methods

### Method

To assess the primary objective and rate depression, the self-report CES-D scale was used. According to a previously developed method (7, 16, 17) the cut-off score of 23/24 for the CES-D and a derived algorithm were used to identify cases of probable depression. This algorithm utilized the weighted scores of selected CES-D items to arrive at the diagnosis of depression, and has already been validated. Cases identified by only either method were considered cases of distress (false positive cases in terms of depression), while cases identified by both the cut-off and the algorithm were considered as probable depression. The STAI-S (18) and the RASS (17) were used to assess anxiety and suicidality respectively.

The protocol also included the collection of sociodemographic data and previous mental and somatic health history. A long questionnaire concerning beliefs in conspiracy theories was also utilized.

The data were collected online and anonymously from April 2020 through March 2021, covering periods of full implementation of lockdowns as well as of relaxations of measures in countries around the world. Announcements and advertisements were made on social media and through news sites, but no other organized effort had been undertaken. The first page included a declaration of consent which everybody accepted by continuing with the participation. Filling of all fields was obligatory to avoid the problem of missing data.

The complete protocol used is available in the Webappendix; each question was given an ID code; these ID codes were used throughout the results for increased accuracy.

Approval was initially given by the Ethics Committee of the Faculty of Medicine, Aristotle University of Thessaloniki, Greece, and locally concerning each participating country.

### Material

Eleven countries (Bulgaria, Croatia, Georgia, Greece, Hungary, India, Latvia, Lithuania, Malaysia, Nigeria, and Russia) participated in the study, and data from 13,354 persons were initially gathered. Only data from those aged between 17 and 30 years were kept and thus, the sample included 12,488 subjects, of whom 9,026 were females (72.27 %; aged 21.11 ± 2.53), 3,329 males (26.65%; aged 21.61 ± 2.81) and 133 “non-binary gender” (1.06%; aged 21.02 ± 2.98). The sample composition in terms of country of origin (A1) by sex (A2) and of the field of studies (A8) is shown in WebTables 1, 2. Subjects were classified, depending on their studies, into three groups: group A (health and biological sciences), group B (technical sciences), and group C (arts, literature, education and related sciences). Psychology, anthropology, various therapies, and athletics were included in group A, Economics in group B, social workers, and social sciences in group C. The size and composition of each group are shown in Table 1 and WebTable 2. Each of the three groups accounted for roughly one-third of the study sample with the percentage of males being double in group B in comparison to groups A and C.

**Table 1:** Study type by sex descriptive statistics of the study sample.

**Study type**	**Females**		**Males**		**Non-binary gender**		**Total**	
	***N***	**%**	***N***	**%**	***N***	**%**	***N***	**%**
A	3,262	77.80	888	21.18	43	1.03	4,193	33.58
B	2,539	60.50	1,612	38.41	46	1.10	4,197	33.61
C	3,225	78.70	829	20.23	44	1.07	4,098	32.82
Total	9,026	72.28	3,329	26.66	133	1.07	12,488	100.00

Group A: health and biological sciences, psychology, anthropology, various therapies and athletics. Group B: technical sciences, physics, mathematics, economics. Group C: arts, literature, education and related sciences, social workers and social sciences and “non-binary gender.”

The fact that the majority were females reflects a common phenomenon in this kind of studies with online gathering of data and self-selection of participation. This means that results should be calculated separately for males and females.

The study population was self-selected. It was not possible to apply post-stratification on the sample as it was done in a previous study (7), because this would mean that we would utilize a similar methodology across much different countries and the population data needed were not available for all. There was no a-priori calculation of the sample size.

### Statistical analysis

The analysis of data included the following:

- Descriptive tables were created for the variables under investigation.
- Chi-square tests were used for the comparison of frequencies when categorical variables were present and for the *post-hoc* analysis of the results a Bonferroni-corrected method of pair-wise comparisons was utilized (19).
- Pearson Product Moment Correlation Coefficient (R) to investigate the relationship between variables
- Analysis of Covariance (ANCOVA) was used to test for the main effect as well as the interaction among categorical variables, with Schefee as *post hoc* test to investigate which variables could contribute to the development of others.
  - Multiple forward stepwise linear regression analysis (MFSLRA) was performed to investigate which variables could function as predictors and contribute to the development of others (e.g., depression).
- Relative Risk (RR) was calculated as the ratio of the incidence in two groups under comparison.

The way each of the above methods was utilized is described specifically in the Results Section.

There were no missing data since the filling of all questionnaire fields was obligatory.

## Results

### Description of the study sample

#### Demographics

The composition of the study sample is described in the “Material” Section and Table 1 and WebTables 1, 2. Additionally, 10.05% of the study sample were suffering from a chronic medical condition (B2) and 54.58% of them had a person belonging to a vulnerable group in the family (B4).

#### History of mental health (B5, O12, O13)

The detailed mental health history is shown in Table 2 and Webtables 4–6. Any such history was reported by 3,258 subjects (26.09%; B5). The lowest rate was observed in males of study type C and the highest in “non-binary gender” of study type A. Anxiety ranged from 4.95% (in males of type C studies) to 13.95% (in “non-binary gender” of study type A) and 13.27% (in females of study type C). Depression ranged from 7.96% (in males of type C studies) to 22.73% (in “non-binary gender” of study type C).

**Table 2:** Rates of history of mental health in the study sample.

**History of**	***N***	**%**
Any history of mental disorder	3,258	26.09
No history any mental disorder	9,230	73.91
Anxiety	1,210	9.69
Depression	1,365	10.93
Bipolar disorder	128	1.02
Psychosis	125	1.00
Self-harm (at least once)	3,175	25.43
Suicide attempt (at least once)	904	7.24
Other	430	3.44

History of self-injury (at least once; O12) was present in 25.43% with the highest rate in “non-binary gender” in type C studies (47.73%) and the lowest in males in type B studies (17.87%). Suicidal attempt (at least once; O13) was reported by 7.24%, with the highest rate reported by “non-binary gender” in study type A (25.58%) and the lowest in males of type C studies (5.55%).

The rate of females to males for a history of any mental disorders was 1.5:1 and the rate of “non-binary gender” was 1.48 vs. females and 2.22 vs. males.

Chi-square tests suggested that females had higher rates of self-injury and suicidal attempts (both *p* < 0.01), while the interaction of sex and type of studies produced more complex results. In females, subjects in type C had more self-injuries and in males, this was true for males of type A. The above suggests that the ranking of types of studies in terms of the history of self-injury (C > A > B) is driven by females in group C and males in group A. Concerning suicidal attempts, in females there were lower rates in type B while there were no differences among types of studies for males. The above suggest that the ranking of types of studies in terms of history of suicidal attempt (B < A = C) is driven by females in group B (see Appendix for details, section 3.2).

### Current probable depression

Probable depression was found in 29.19% of females and 16.10% of males (25.81% of the total sample) and dysphoria was present in an additional 16.10% of females and 14.29% of males (15.66% of the total sample). The detailed results are shown in Webtables 7–11.

There was a large difference among countries in terms of current probable depression with the lowest rate observed in Nigeria (4.94%) and the highest in Lithuania (43.88%).

The RR for depression was 1.81 for females in comparison to males. Rates of depression were higher for “non-binary gender” in type of study B (46.67%) and lowest for males in A and C (15.99% and 15.92%).

Chi-square test revealed an effect of type of studies by gender concerning probable depression. There was a difference among females in the three types (chi-square = 21.623, df = 2, *p* < 0.001), which was due to A vs. B (chi-square = 16.947, df = 1, *p* < 0.001), A vs. C (chi-square = 15.241, df = 1, *p* < 0.001), but not B vs. C (chi-square = 0.194, df = 1, *p* = 0.659). Concerning males, there was no difference among males in the three types (chi-square = 0.055, df = 2, *p* = 0.972). Similarly, there were no differences concerning “non-binary gender” in the three types of studies (chi-square = 1.027, df = 2, *p* = 0.598). These results suggest a lower depression rate in females in the A type of studies.

There was a difference among sexes (chi-square = 233.240, df = 2, *p* < 0.001), with males having lower rates of probable depression in comparison both to females (chi-square = 218.274, df = 1, *p* < 0.001), and “non-binary gender” (chi-square = 55.258, df = 1, *p* < 0.001), and “non-binary gender” having higher rates both to females (chi-square = 8.622, df = 1, *p* = 0.003) and males.

Four MSLRA were performed. The dependent variables were the change in anxiety, change in depressive feelings, change in suicidal thoughts and probable depression separately, while in all analyses the same set of independent predictors was used and it included sex (A2) split into dummy variables, age (A3), type of studies (split into dummy variables), people living with (A6), health status (B1-2), vulnerable relative (B4), history of specific mental disorders (B5 split in dummy variables), thoughts pertaining to COVID-19 fears (C1-4), the degree of lockdown (D2), satisfaction by information (D4), family issues (E1-7), conspiracy theories (J1-26) and spirituality/religiosity (P1).

**Figure 1:**
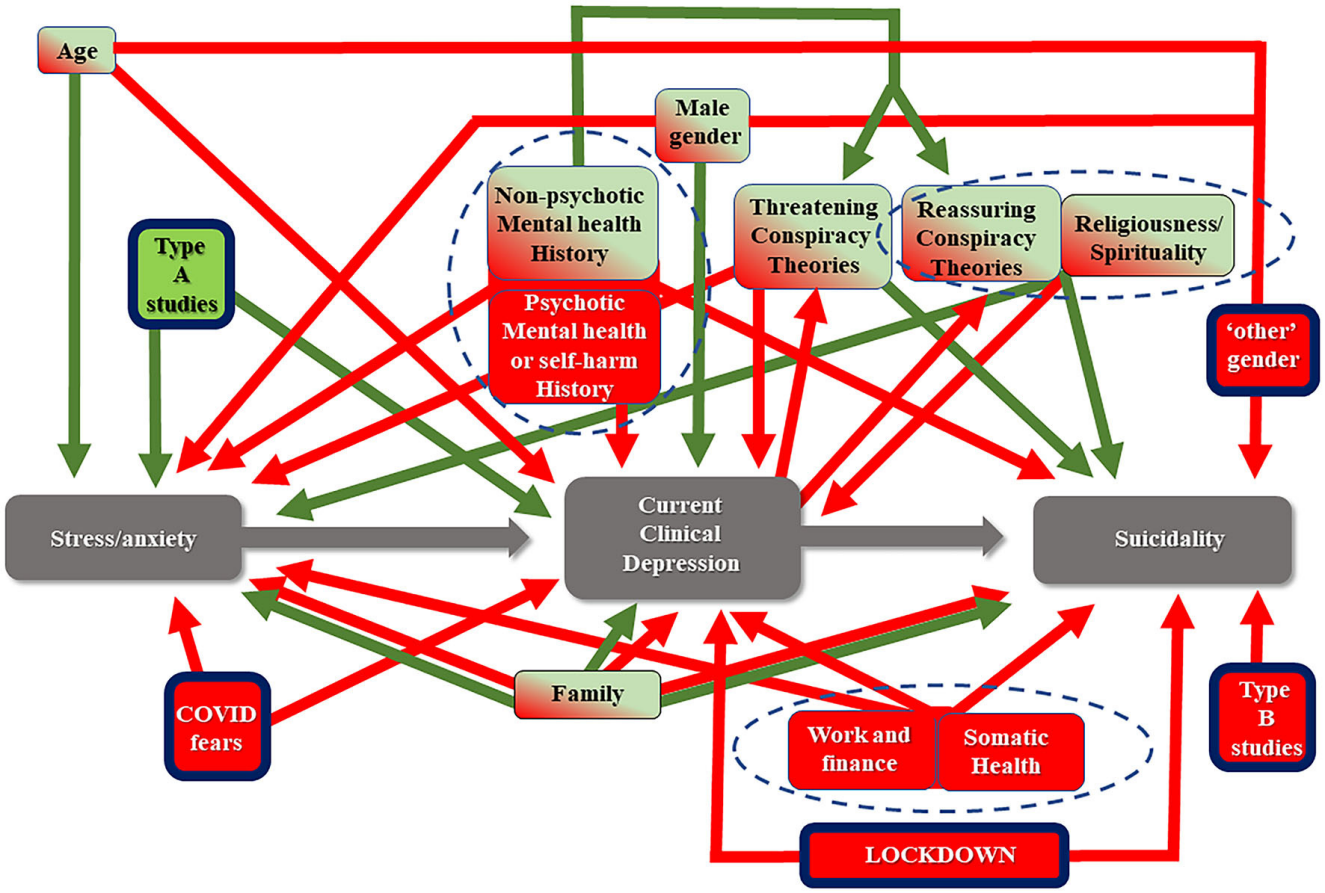
The developed multiple vulnerabilities model representing the mechanism through which the COVID-19 outbreak in combination a great number of factors could lead to depression through stress, and eventually to suicidality in university students. A number of variables act as risk factors (red) or as protective factors (green), while some of them change direction of action depending on the phase (green/red). Three core clusters emerge (delineated with the doted lines). The model differs from a more general model concerning the general population in that the type of studies, “non-binary gender” sex, COVID-related fears and strict lockdown specifically play an additional role (rectangles with thick black frame).

The detailed results are shown in Table 3 and confirm the effect of sex, history of mental disorder, fears because of the pandemic, and believing in conspiracy theories on the mental health of students during the pandemic. The complete model which can be derived based on these MSLRA is shown in Figure 1.

**Table 3:** Multiple linear stepwise regression analysis with changes in anxiety, depression, or suicidality and the presence of probable depression as dependent variables separately.

**Dependent variable: Change in anxiety**				
**R**^**2**^ = **0.238; variance expl 23.8%;** ***F***_**(35,12124)**_ = **108.41** ***p***<**0.0000 Std.Error of estimate: 0.749**				
***b***	**Sth.Err**.	***t*** **(12124)**	***p*** **–value**
Intercept	−0.71	0.04	−17.24	0.0000
Male sex	0.09	0.02	5.48	0.0000
Type A studies	0.04	0.02	2.49	0.0129
Number of people in the house	0.02	0.01	4.02	0.0001
Condition of general health	0.12	0.01	18.27	0.0000
Vulnerable person in the family	−0.05	0.01	−3.25	0.0012
History of anxiety disorder	−0.1	0.02	−4.36	0.0000
History of depression	−0.09	0.02	−4.14	0.0000
History of bipolar disorder	−0.14	0.07	−1.99	0.0462
C1. Are you afraid that you will contract the coronavirus?	−0.05	0.01	−6.47	0.0000
C3. Does the possibility that a member of your family could contract the coronavirus and die because of it, makes you frightened?	−0.05	0.01	−6.85	0.0000
C4. Are you afraid that in case you contract the coronavirus, some people will step away from your life and behave to you in a different way later?	−0.02	0.01	−3.28	0.0010
D2. Are you currently locked up in the house?	−0.06	0.01	−7.99	0.0000
E2. Do you want to receive emotional support from other members of your family during this period?	−0.12	0.01	−14.77	0.0000
E3. Are there any conflicts with the rest of your family members during this period?	−0.1	0.01	−12.54	0.0000
E4. Has the overall quality of relationships with the other members of your family changed compared to before the COVID-19?	0.13	0.01	12.65	0.0000
E5. Do you manage to maintain a basic daily routine (waking up in the morning, regular meals and sleeping hours, activities) both yourself (if you live alone) or as a family?	0.12	0.01	15.05	0.0000
E7. How are your finances as a result of the outbreak?	0.1	0.01	12.42	0.0000
J5. Do you believe that COVID-19 appeared accidentally from human contact with animals and it was something that generally happens and was generally expected?	−0.03	0.01	−5.17	0.0000
J15. Secret organizations are communicating with aliens, but they hide it from the public.	0.04	0.01	4.15	0.0000
J24. Many important pieces of information are deliberately hidden from the public for reasons of interest	−0.04	0.01	−6.58	0.0000
J3. Do you think that COVID-19 was created to be used as a biochemical weapon for the extermination of the human population?	0.02	0.01	2.45	0.0143
J6. Do you believe that COVID-19 has much lower mortality rate but there is misinformation and terror-inducing propaganda?	−0.02	0.01	−2.53	0.0113
J7. Do you think the recommended measures (e.g., wearing face masks, avoid gatherings, stay at home etc.) are an attempt to restrict human rights and lead to some kind of dictatorship rather than to keep the population safer from COVID-19?	−0.04	0.01	−5.63	0.0000
J8. Do you believe that COVID-19 outbreak is a deliberate creation of the world's powerful leaders to create a global economic crisis?	−0.03	0.01	−3.87	0.0001
J9. Do you believe that COVID-19 is a sign of divine power to destroy our planet?	0.05	0.01	5.49	0.0000
J14. The power held by the heads of state is smaller than that of small unknown groups that really control the world of politics.	0.02	0.01	2.88	0.0040
J17. The government allows or commits acts of terrorism on its territory, disguising its involvement as if someone else is responsible.	−0.03	0.01	−4.03	0.0001
J18. Do you believe that secretly a chip will be included in the COVID-19 vaccine in order to mark people?	0.02	0.01	2.37	0.0178
J23. Experiments involving new drugs or technologies are performed systematically on humans in a secret way and without their knowledge or consent.	0.03	0.01	3.38	0.0007
J26. It is possible that the earth is flat rather than spherical.	−0.03	0.01	−2.28	0.0228
P1. Over the last 2–3 weeks, have your religious/spiritual inquiries been increased?	0.02	0.01	2.03	0.0426
				
**Dependent variable: change in depressive emotions**				
**R**^2^= **0.195; explained var 19.5%**. *F*_(35,12124)_ = **83.896** ***p***<**0.0000 Std.Error of estimate: 0.763**				
	***b***	**Std.Err**.	***t*** **(12124)**	***p*** **–value**
Intercept	−0.9	0.07	−12.11	0.0000
Male sex	0.04	0.02	2.51	0.0120
Age	0.01	0	2.7	0.0069
Type A studies	0.05	0.02	3.02	0.0025
Number of people in the house	0.03	0.01	4.52	0.0000
B1. General health over the last month	0.1	0.01	15.47	0.0000
Vulnerable person in the family	−0.04	0.01	−2.84	0.0045
History of depression	−0.12	0.02	−5.32	0.0000
C1. Are you afraid that you will contract the coronavirus?	−0.03	0.01	−3.18	0.0015
C2. Do you believe that the precautions work effectively or that if you are about to contract the disease, you will contract it anyway?	0.05	0.02	3.4	0.0007
C3. Does the possibility that a member of your family could contract the coronavirus and die because of it, makes you frightened?	−0.02	0.01	−2.72	0.0065
C4. Are you afraid that in case you contract the coronavirus, some people will step away from your life and behave to you in a different way later?	−0.03	0.01	−4.11	0.0000
D2. Are you currently locked up in the house?	−0.08	0.01	−9.94	0.0000
E1. Do you feel the need to communicate with other members of your family during this period?	0.03	0.01	2.71	0.0068
E2. Do you want to receive emotional support from other members of your family during this period?	−0.12	0.01	−12.07	0.0000
E3. Are there any conflicts with the rest of your family members during this period?	−0.08	0.01	−9.07	0.0000
E4. Has the overall quality of relationships with the other members of your family changed compared to before the COVID-19?	0.14	0.01	13.24	0.0000
E5. Do you manage to maintain a basic daily routine (waking up in the morning, regular meals and sleeping hours, activities) both yourself (if you live alone) or as a family?	0.12	0.01	14.18	0.0000
E7. How are your finances as a result of the outbreak?	0.06	0.01	7.52	0.0000
J1. Do you believe that the COVID-19 vaccine was ready even before the virus broke out and they conceal it from us for the benefit of pharmaceutical companies?	0.02	0.01	2.63	0.0084
J5. Do you believe that COVID-19 appeared accidentally from human contact with animals and it was something that generally happens and was generally expected?	−0.02	0.01	−4.02	0.0001
J6. Do you believe that COVID-19 has much lower mortality rate but there is misinformation and terror–inducing propaganda?	−0.02	0.01	−3.65	0.0003
J7. Do you think the recommended measures (e.g., wearing face masks, avoid gatherings, stay at home etc.) are an attempt to restrict human rights and lead to some kind of dictatorship rather than to keep the population safer from COVID-19 ?	−0.04	0.01	−6.03	0.0000
J9. Do you believe that COVID-19 is a sign of divine power to destroy our planet?	0.04	0.01	3.98	0.0001
J13. Global warming and climate change is a greatly exaggerated myth to serve various political and financial interests.	0.02	0.01	2.38	0.0174
J15. Secret organizations are communicating with aliens, but they hide it from the public.	0.04	0.01	4.33	0.0000
J17. The government allows or commits acts of terrorism on its territory, disguising its involvement as if someone else is responsible.	−0.03	0.01	−4.63	0.0000
J18. Do you believe that secretly a chip will be included in the COVID-19 vaccine in order to mark people?	0.06	0.01	5.66	0.0000
J23. Experiments involving new drugs or technologies are performed systematically on humans in a secret way and without their knowledge or consent.	0.02	0.01	2.64	0.0084
J24. Many important pieces of information are deliberately hidden from the public for reasons of interest.	−0.03	0.01	−4.65	0.0000
J26. It is possible that the earth is flat rather than spherical.	−0.03	0.01	−2.03	0.0423
				
**Dependent variable: Change in suicidal thoughts**				
**R**^2^= **0.062; explained var: 6.2%** *F*_(29,12130)_ = **27.920** ***p***<**0.0000 Std. Error of estimate: 0.672**				
	***b***	**Std.Err**.	***t*** **(12130)**	***p*** **–value**
Intercept	0.16	0.03	5.34	0.0000
“Non–binary gender” sex	0.12	0.06	2.03	0.0427
Type B studies	0.03	0.01	2.41	0.0159
B1. General health over the last month	−0.05	0.01	−7.57	0.0000
History of depression	0.14	0.02	7.01	0.0000
History of bipolar disorder	0.25	0.06	4.16	0.0000
History of psychosis	0.12	0.06	1.97	0.0492
C1. Are you afraid that you will contract the coronavirus?	0.02	0.01	3.07	0.0021
C4. Are you afraid that in case you contract the coronavirus, some people will step away from your life and behave to you in a different way later?	0.02	0.01	2.88	0.0040
D2. Are you currently locked up in the house?	0.03	0.01	4.48	0.0000
E2. Do you want to receive emotional support from other members of your family during this period?	0.05	0.01	7.56	0.0000
E3. Are there any conflicts with the rest of your family members during this period?	0.05	0.01	6.57	0.0000
E4. Has the overall quality of relationships with the other members of your family changed compared to before the COVID-19?	−0.05	0.01	−5.42	0.0000
E5. Do you manage to maintain a basic daily routine (waking up in the morning, regular meals and sleeping hours, activities) both yourself (if you live alone) or as a family?	−0.05	0.01	−6.81	0.0000
E7. How are your finances as a result of the outbreak?	−0.02	0.01	−2.99	0.0028
J7. Do you think the recommended measures (e.g., wearing face masks, avoid gatherings, stay at home etc.) are an attempt to restrict human rights and lead to some kind of dictatorship rather than to keep the population safer from COVID-19 ?	0.02	0.01	2.81	0.0049
J11. Do you think that vaccines in general are dangerous and should be avoided?	−0.03	0.01	−3.67	0.0002
J12. The government is secretly involved in the murder of innocent citizens and/or well-known public figures.	0.02	0.01	2.24	0.0250
J14. The power held by the heads of state is smaller than that of small unknown groups that really control the world of politics.	0.02	0.01	3.25	0.0012
J15. Secret organizations are communicating with aliens, but they hide it from the public.	0.02	0.01	2.06	0.0395
J17. The government allows or commits acts of terrorism on its territory, disguising its involvement as if someone else is responsible.	0.02	0.01	3.39	0.0007
J18. Do you believe that secretly a chip will be included in the COVID-19 vaccine in order to mark people?	−0.03	0.01	−3.38	0.0007
J20. Technology and devices for mind control are used on people without their knowledge	−0.02	0.01	−2.22	0.0267
J26. It is possible that the earth is flat rather than spherical	−0.04	0.01	−3.38	0.0007
**Dependent variable: Probable depression**				
**R**^2^= **0.184; Explained var 18.4%;** *F*_(26,12133)_ = **105.33** ***p***<**0.0000 Std.Error of estimate: 0.393**				
	***b***	**Std.Err**.	***t*** **(12133)**	***p*** **–value**
Intercept	0.33	0.02	16.30	0.0000
Males	−0.08	0.01	−9.06	0.0000
“Non-binary gender” sex	0.07	0.03	2.05	0.0408
Type A studies	−0.02	0.01	−2.87	0.0041
Number of people in the house	−0.01	0.00	−3.06	0.0022
B1. General health over the last month	−0.04	0.00	−12.66	0.0000
History of anxiety disorder	0.07	0.01	5.43	0.0000
History of depression	0.21	0.01	18.08	0.0000
History of bipolar disorder	0.26	0.04	7.41	0.0000
History of psychosis	0.23	0.04	6.47	0.0000
C2. Do you believe that the precautions work effectively or that if you are about to contract the disease, you will contract it anyway?	−0.03	0.01	−3.13	0.0018
C3. Does the possibility that a member of your family could contract the coronavirus and die because of it, makes you frightened?	0.01	0.00	3.48	0.0005
C4. Are you afraid that in case you contract the coronavirus, some people will step away from your life and behave to you in a different way later?	0.02	0.00	6.82	0.0000
D2. Are you currently locked up in the house?	0.01	0.00	2.37	0.0180
E1. Do you feel the need to communicate with other members of your family during this period?	−0.02	0.01	−3.50	0.0005
E2. Do you want to receive emotional support from other members of your family during this period?	0.05	0.00	9.74	0.0000
E3. Are there any conflicts with the rest of your family members during this period?	0.03	0.00	7.87	0.0000
E4. Has the overall quality of relationships with the other members of your family changed compared to before the COVID-19?	−0.03	0.01	−5.54	0.0000
E5. Do you manage to maintain a basic daily routine (waking up in the morning, regular meals and sleeping hours, activities) both yourself (if you live alone) or as a family?	−0.08	0.00	−18.41	0.0000
E6. If you have children, how difficult is it to manage their daily life and behavior?	0.03	0.02	2.07	0.0385
E7. How are your finances as a result of the outbreak?	−0.02	0.00	−6.03	0.0000
J3. Do you think that COVID-19 was created to be used as a biochemical weapon for the extermination of the human population?	0.01	0.00	3.59	0.0003
J5. Do you believe that COVID-19 appeared accidentally from human contact with animals and it was something that generally happens and was generally expected?	0.02	0.00	6.42	0.0000
J17. The government allows or commits acts of terrorism on its territory, disguising its involvement as if someone else is responsible.	0.01	0.00	3.72	0.0002
J23. Experiments involving new drugs or technologies are performed systematically on humans in a secret way and without their knowledge or consent.	0.01	0.00	3.00	0.0027
J24. Many important pieces of information are deliberately hidden from the public for reasons of interest.	0.01	0.00	2.23	0.0257
P1. Over the last 2–3 weeks, have your religious/spiritual inquiries been increased?	0.02	0.00	3.23	0.0012

The independent variables were sex, age, type of studies, history of specific mental disorders and believing in conspiracy theories.

### Secondary aims of the study

#### Current mental health

In the total study sample, increased anxiety (at least “a little”) was present in ~60% (F21), more depressive feelings (at least “a little”) in > 55% (G21); suicidal thoughts were increased (at least “a bit”) in 6.34% (O11). The detailed results are shown in Webtables 7–11.

The presence of history of any mental disorder had a RR of 2.04 for the development of depression which was highest for “non-binary gender” in the type of studies B (RR = 2.76) and lowest for females in the same type of studies (RR = 1.66). In detail the effect of history in the development of depression by sex and type of study is shown in Webtable 12. The highest rate of current probable depression was observed in females and type of studies B or C with a history of bipolar disorder (63.33% and 72.73%) or psychosis (60.71% and 61.29%). “Non-binary gender” had very high rates with any history or type of studies. The lowest rates were observed in type A studies with “other history” in females (21.67%) and males (18.18%), and also in males with a history of anxiety and type B studies (16.16%) and type C studies and “other history” (8.70%).

ANCOVA with the presence of probable depression and history of each mental disorder separately as grouping variables, and changes in anxiety, depressive feelings, and suicidal thoughts as independent variables and sex and age as covariates returned a main effect for probable depression (wilks = 0.851; F = 725.9; effect df:3; error df:12475; *p* < 0.001), history of anxiety (wilks = 0.995; F = 19.1; effect df:3; error df:12475; *p* < 0.001), depression (wilks = 0.996; F = 17.9; effect df:3; error df:12475; *p* < 0.001) and bipolar disorder (wilks = 0.999; F = 4.5 effect df:3; error df:12475; *p* = 0.004). All scheffe *post-hoc* tests were significant at *p* < 0.01. All group means pointed toward a negative change in all three independent variables reflecting a change in mental health.

In terms of suicidality, 17.63% reported that they were thinking of committing suicide (O5) with 5.97% reporting “much” or “very much.”

#### Family, lifestyle changes, and the pandemic

At the time of the interview, 17.11%, were under strict lockdown (D2), 41.90%, to a high degree 24.95% partially, and 16.03% were under no lockdown at all. Chi-square test in pairs among the four lockdown groups and the presence of probable depression returned no differences except for the complete lockdown which manifested significantly higher depression rates in comparison to all the other states of lockdown (31.63% 24.62%; RR = 1.28; *p* < 0.01). All correlations were significant (*p* < 0.05) but minimal among the degree of lockdown and changes in anxiety (R = −0.13), changes in depressive feelings (R = −0.14), changes in suicidal thoughts (R = 0.07), STAI (R = 0.02), CES-D (0.06), RASS-Intention (R = 0.03), RASS life (R = 0.02).

More than 50% of the total sample reported that the time spent outside the house was less than humanly necessary or worse, without any differences among types of studies. More than 90% were suggesting that they were following the precaution measures suggested by the WHO at least moderately, with 2/3 saying that they were much or very much following them. One-third felt that the information was not adequate.

Family dynamics changed toward increased emotional bonding and on average conflicts did not change. Only close to 20% did not manage to keep a basic daily routine and almost half were expecting their financial status to worsen.

More than 90% considered exercise to be of high importance during the pandemic but more persons experienced a decrease in physical activity. Eating increased in 40% and ~20% was eating in an unhealthier way. More than 30% put weight.

Half of the study sample increased the time spent on the internet and almost 2/3 increased the use of social media. Close to 25% acquired new internet habits.

Sleep worsened in ~45% with more than 50% going to sleep rather late and 20% having nightmares. Smoking increased in 25%, alcohol use in close to 40%, and illegal substance use in 25%. Sexual life was poor in ~45% with a decrease in desire in ~20%.

These findings were comparable across types of studies (WebTable 13).

#### Beliefs in conspiracy theories

The rates of the beliefs in conspiracy theories depended on the nature of the belief, with more bizarre theories enjoying lower acceptance. Differences among countries were significant, but there were no overall differences among the sexes. Interestingly, the type A studies had surprisingly high acceptance rates of COVID-19 conspiracy theories with close to 25% believing that the vaccines include a chip and almost 40% suggesting that facemask wearing could be a method of socio-political control. One-third of persons of type A studies were reserved toward vaccines in general, 20% were accepting the chemtrail conspiracy, 20% disputed climate change, 40% were not precluding that mind control devices are used upon the population, 45% were believing that experiments of new drugs and technologies are systematically performed secretly on the population and 8% were accepting the flat earth theory. The rate of the flat earth theory adds validity to our study sample as it is comparable, although lower to that reported by other studies.

Rates of believing were lower in persons without dysphoria or depression, intermediate in those with dysphoria, and higher in those with current probable depression. There was no relationship between history of any mental disorders and conspiracism.

The results concerning conspiracy theories are shown in detail in WebTables 14–19.

## Discussion

The results of the current international study on a large convenient sample, across 11 countries, probable depression was present in 25.81% with an additional 15.66% experiencing severe distress. Prior history doubled the risk of developing depression. A significant number of variables contributed to the developed model and acted either as risk or as protective factors. Altogether they explained 18.4% of the development of depression. An unfortunate finding was that the individual contribution of individual variables was very small. A quarter of these 12,488 university students manifested a history of mental disorder and ~7% had attempted at least once. The fact that the majority were females reflects a common phenomenon in this kind of studies with online gathering of data and self-selection of participation (11, 20–22). During the COVID-19 pandemic, over half of these university students reported an increase in anxiety and depressive feelings and 6.34% in suicidal thoughts. A worsening of quality of life and deterioration of lifestyle issues were also found. Conspiracy theories manifested a complex effect, and the belief in them seemed widely prevalent with acceptance rates depending on the nature of the belief, with more bizarre theories enjoying lower acceptance. Differences among countries were significant, but there were no overall differences among the sexes. Interestingly, health-related university studies had surprisingly high acceptance rates of COVID-19 conspiracy theories with close to 25% believing that the vaccines include a chip and almost 40%, suggesting that facemask wearing could be a method of socio-political control. One-third of them were reserved toward vaccines in general, 20% were accepting the chemtrail conspiracy, 20% disputed climate change, 40% were not precluding that mind control devices are used upon the population, 45% believed that experiments of new drugs and technologies are systematically performed secretly on the population and 8% were accepting the flat earth theory. Rates of believing were lower in persons without dysphoria or depression, intermediate in those with dysphoria, and higher in those with current probable depression. There was no relationship between history of any mental disorders and conspiracism.

The first question that arises from this kind of study samples (online study with self-selection) is the extent to which the conclusions are valid, and the study sample does not manifest some kind of systematic bias. The features that support the validity of the study sample are the high depression levels, even in the subgroup which was not under any kind of lockdown, the large discrepancy (which makes sense) among rates of beliefs in individual conspiracy theories, with the rate of believing in flat earth being a cardinal finding, which is more or less in accord with the reports of yougov.com (23).

The female:male ratio in terms of probable depression is another feature that supports the validity of our study sample.

The basic results of the current study are in accord with the literature, which however includes many studies that report on depressive symptoms (24), but only a few on rates of probable depression. Most of them are on medical students and so far support that during the COVID-19 outbreak, the depression rates were around 20-30% while also similar rates of anxiety were present (8–11, 21, 25–32). The overall rates probable depression reported by the current study were lower than the rates reported in the literature, and this was likely a consequence of the algorithm used and of the stringent criteria it applied. Self-injuring acts were reported in up to 40% (33). Others report that up to half of students were suffering from a mental disorder (22, 34, 35). Interestingly, some report no differences between sexes (32) but this is not the rule. There are studies in the general population concerning the role of self-determined sex (36–41). Lockdown was recognized as a strong risk factor (42), along with prior history (43). The finding that rates of depression increase significantly with strict lockdown (RR = 1.28) is in accord with other reports (26) and also point to the possibility this increase is only temporary and questions whether these rates reflect true depression or an intense adjustment reaction with depressive affect. This lockdown effect has been well documented on the general population (20, 44–48), but most results seem to suggest an enduring effect (49) which might not be in accord with our findings.

However one critical element is that the rates of probable depression in university students were reported to be high even before the pandemic (1, 4, 14, 50–71). It is therefore questionable whether the findings of the current study reflect elevated rates of depression. One finding that supports this is the relationship between these rates with the intensity of lockdown.

The multivariate analysis in the current study proposed a model for the development of depression and suicidality during the pandemic. Similar but less specific or detailed models have been proposed (72), with some authors suggesting that the increase in suicidality is limited to sexual minorities (73). The developed model (Figure 1) includes a significant number of variables. They seem to act either as risk or as protective factors. Altogethere they explain 18.4% of the phenomenon of depression development. Interestingly, the individual contribution of each variable was very small. Another finding was that conspiracism manifested a complex effect. Current probable depression acted as a risk factor for the development of such beliefs. This model starts with the assumption that stress and anxiety develop first. Depression then follows, while suicidality emerges as the end result. These are distinct stages, and the basic assumption is that there is progress from earlier to later stages, which however, is not mandatory.

In line with the proposed model, as the pandemic appeared, it exerted a severe psychological impact that resulted in severe anxiety and distress. Both were determined by several sociodemographic and interpersonal variables that included sex, age, thoughts, beliefs and fears that were specific to the outbreak and to the intensity of lockdowns, as well as to relationships among family members, the ability to keep a basic daily routine, the economic situation and its changes, the presence of mental disorder history and, most important, the fear that the person or a family member will get COVID-19 and die. The role of the type of studies was important also, with studies pertaining to health sciences being protective during the early stages while studies related to polytechnic, physics, mathematics, and related sciences being risk factors for the development of suicidality. In the literature there are reports with similar findings but the contribution of the current study is that it identified their specific contribution and developed a comprehensive model.

Conspiracism is currently widely accepted as being an important contributing factor since the literature strongly supports its relationship with anxiety and depression (74, 75), but most important is their role in the resistance against vaccination of the entire population. The high rates of believing in conspiracy theories are in accord with findings from various countries (76–79). Conspiracism and especially those beliefs regarding medicine, and health-related issues are not uncommon (80), they are widely discussed in social media (76, 81) and they challenge the capacity of the average person to distill and assess the content (82, 83). Their adverse effect on health behaviors is well-documented, and this concerns especially vaccination (78, 84–97). Some relationship might be present between believing in bizarre conspiracy theories and the presence of psychotic tendencies or of a history of psychosis (98). Our current findings did not support previous reports that particular type of studies are preferentially related to conspiracism (8, 9).

What is extremely interesting is the finding of the current study concerning the rates of believing in conspiracy theories (WebTables 14–19). For example, ~20% of medical students were believing that maybe the vaccine was ready before the COVID-19 outbreak (J1) with 5% believing it strongly. The respected rates for the 5G theory (J4) were 7% and 1.5%, while concerning the possibility of the deliberately inflated mortality rates (J6) were >20% and 6% respectively. In the same group of students, the acceptance of the chemtrails conspiracy (J10) was 7% and close to 2% and that a chip will be included in the vaccines (J18) was 9% and >2% respectively. The vaccines in general were considered as dangerous (J11) by >11% and 1.77% while astonishingly, the flat earth theory (J26) was embraced by close to 5.5% and 1.5% of medical students respectively with an additional 2.8% not precluding it! Reserved toward vaccination in general were ~25% of medical students. Measures including facemask wearing were considered to be rather an attempt of socio-political control (J7) by 15% and >3% respectively, while only 72% precluded this idea. All these rates were much higher in students of nursing. These results are generally in accord with the yougov.com reports (23) and explain the resistance to measures and especially to vaccination by a minority of doctors and other health professionals.

Current probable depression is a critical factor related to conspiracism. As correlation does not imply causation, conspiracism could be any of the following: the cause of depression, a copying mechanism, or a marker of maladaptive psychological patterns of cognitive appraisal. The authors suggest that the most likely explanation is that conspiracism is probably a coping mechanism against stress and concerns the entire general population (75, 99, 100).

A question that is difficult-to-answer is the real rates of major depression since the use of questionnaires and sophisticated algorithms is not as reliable and valid as direct interview and the underlying neurobiology is unknown (101).

## Conclusion

The current study reports high rates of depression, dysphoria, and suicidal thoughts in university students during the pandemic and especially during the periods of strict lockdown. The prevalence of conspiracism was high, including medical conspiracy theories in medical students. A complex model is proposed for the development of depression, which includes female sex, strict lockdown, family and economic factors, type of studies, and prior history, while believing in conspiracy theories probably acts as a protective factor. These findings, support previous suggestions by other authors, and although they should be closely monitored longitudinally, they clearly point to the need for a proactive intervention that would aim to protect the mental health of the general population but more specifically of vulnerable groups (102, 103).

## Strengths and limitations

The strengths of the current paper include the large number of persons who filled out the questionnaire and the large bulk of in-depth information obtained. A number of anchor points e.g., rates of believing in the flat earth theory and differences in rates among conspiracy theories support the validity of the sample.

The major limitation was that the data were obtained anonymously online through the self-selection of the responders. Additionally, the assessment included only the cross-sectional application of self-report scales, although the advanced algorithm used for the diagnosis of probable depression corrected the problem to a certain degree. However, what is included under the umbrella of “probable depression” in the stressful times of the pandemic remains a matter of debate. Also, the lack of baseline data concerning the mental health of a similar study sample before the pandemic is also a problem.

## Data availability statement

The raw data supporting the conclusions of this article will be made available by the authors, without undue reservation.

## Ethics statement

The studies involving humans were approved by the Aristotle University of Thessaloniki. The studies were conducted in accordance with the local legislation and institutional requirements. The participants provided their written informed consent to participate in this study. Written informed consent was obtained from the individual(s) for the publication of any potentially identifiable images or data included in this article.

## Author contributions

KF: Conceptualization, Data curation, Formal analysis, Investigation, Methodology, Supervision, Validation, Visualization, Writing – original draft, Writing – review & editing. NA: Data curation, Formal analysis, Investigation, Methodology, Validation, Writing – review & editing. SB: Data curation, Formal analysis, Investigation, Methodology, Validation, Writing – review & editing. NF: Data curation, Formal analysis, Investigation, Methodology, Validation, Writing – review & editing. XG: Data curation, Formal analysis, Investigation, Methodology, Validation, Writing – review & editing. JH: Data curation, Formal analysis, Investigation, Methodology, Validation, Writing – review & editing. MJ: Data curation, Formal analysis, Investigation, Methodology, Validation, Writing – review & editing. BK: Data curation, Formal analysis, Investigation, Methodology, Validation, Writing – review & editing. GM: Data curation, Formal analysis, Investigation, Methodology, Validation, Writing – review & editing. AM: Data curation, Formal analysis, Investigation, Methodology, Validation, Writing – review & editing. MM: Data curation, Formal analysis, Investigation, Methodology, Validation, Writing – review & editing. IN: Data curation, Formal analysis, Investigation, Methodology, Validation, Writing – review & editing. AN: Data curation, Formal analysis, Investigation, Methodology, Validation, Writing – review & editing. MP: Data curation, Formal analysis, Investigation, Methodology, Validation, Writing – review & editing. AP: Data curation, Formal analysis, Investigation, Methodology, Validation, Writing – review & editing. SP: Data curation, Formal analysis, Investigation, Methodology, Validation, Writing – review & editing. SR: Data curation, Formal analysis, Investigation, Methodology, Validation, Writing – review & editing. DR: Data curation, Formal analysis, Investigation, Methodology, Validation, Writing – review & editing. IR: Data curation, Formal analysis, Investigation, Methodology, Validation, Writing – review & editing. ASS: Data curation, Formal analysis, Investigation, Methodology, Validation, Writing – review & editing. AS: Data curation, Formal analysis, Investigation, Methodology, Validation, Writing – review & editing. MS: Data curation, Formal analysis, Investigation, Methodology, Validation, Writing – review & editing. KT: Data curation, Formal analysis, Investigation, Methodology, Validation, Writing – review & editing. JVo: Data curation, Formal analysis, Investigation, Methodology, Validation, Writing – review & editing. ER: Data curation, Formal analysis, Investigation, Methodology, Validation, Writing – review & editing. JVr: Data curation, Formal analysis, Investigation, Methodology, Validation, Writing – review & editing. AJ: Data curation, Formal analysis, Investigation, Methodology, Validation, Writing – review & editing. PT: Data curation, Formal analysis, Investigation, Methodology, Validation, Writing – review & editing. JB: Data curation, Formal analysis, Investigation, Methodology, Validation, Writing – review & editing. DS: Data curation, Formal analysis, Investigation, Methodology, Validation, Writing – review & editing.
