# Supplementary material for: Students' mental health during the pandemic: results of the observational cross-sectional COVID-19 MEntal health inTernational for university Students (COMET-S) study
Source: Front Psychiatry. 2024 Jan 11;14:1320156. doi: 10.3389/fpsyt.2023.1320156 (PMC10825874; doi:10.3389/fpsyt.2023.1320156)
Supplement: Supplementary file 1 [file Data_Sheet_1.docx]

**Students’ mental health during the pandemic: Results of the observational cross-sectional COVID-19 MEntal health inTernational for university Students (COMET-S) study.**

**Konstantinos N Fountoulakis* et al**

**Correspondence:** Konstantinos N Fountoulakis. E-mail: kostasfountoulakis@gmail.com

**Supplementary data**

PART 1: Material and methods

PART 2: The study protocol

PART 3: Results

3.1 Demographics

3.2 History of mental disorder and of self-injury or suicidal attempt

3.3 Present mental status during the pandemic

3.4 Lifestyle changes during the pandemic

3.5 Beliefs in conspiracy theories

3.6 Determinants of worsening of students’ mental health during the pandemic

**PART 1: Material and methods**

**Conforming with the STROBE guidelines for the reporting of observational studies ^1^.**

|  | | Item No | Recommendation | Page  No |
| --- | --- | --- | --- | --- |
| **Title and abstract** | | 1 | (*a*) Indicate the study’s design with a commonly used term in the title or the abstract | 1 |
|  |  |  | (*b*) Provide in the abstract an informative and balanced summary of what was done and what was found | 7 |
| Introduction | | | | |
| Background/rationale | | 2 | Explain the scientific background and rationale for the investigation being reported | 8 |
| Objectives | | 3 | State specific objectives, including any prespecified hypotheses | 8 |
| Methods | | | | |
| Study design | | 4 | Present key elements of study design early in the paper | 9 |
| Setting | | 5 | Describe the setting, locations, and relevant dates, including periods of recruitment, exposure, follow-up, and data collection | 9 |
| Participants | | 6 | (*a*) *Cohort study*—Give the eligibility criteria, and the sources and methods of selection of participants. Describe methods of follow-up  *Case-control study*—Give the eligibility criteria, and the sources and methods of case ascertainment and control selection. Give the rationale for the choice of cases and controls  *Cross-sectional study*—Give the eligibility criteria, and the sources and methods of selection of participants | 9 |
|  |  |  | (*b*) *Cohort study*—For matched studies, give matching criteria and number of exposed and unexposed  *Case-control study*—For matched studies, give matching criteria and the number of controls per case |  |
| Variables | | 7 | Clearly define all outcomes, exposures, predictors, potential confounders, and effect modifiers. Give diagnostic criteria, if applicable | 9 |
| Data sources/ measurement | | 8* | For each variable of interest, give sources of data and details of methods of assessment (measurement). Describe comparability of assessment methods if there is more than one group | *9, webappendix* |
| Bias | | 9 | Describe any efforts to address potential sources of bias | 10 |
| Study size | | 10 | Explain how the study size was arrived at | 10 |
| Quantitative variables | | 11 | Explain how quantitative variables were handled in the analyses. If applicable, describe which groupings were chosen and why | 10 |
| Statistical methods | | 12 | (*a*) Describe all statistical methods, including those used to control for confounding | 10 |
|  |  |  | (*b*) Describe any methods used to examine subgroups and interactions | 10 |
|  |  |  | (*c*) Explain how missing data were addressed | 9 |
|  |  |  | (*d*) *Cohort study*—If applicable, explain how loss to follow-up was addressed  *Case-control study*—If applicable, explain how matching of cases and controls was addressed  *Cross-sectional study*—If applicable, describe analytical methods taking account of sampling strategy |  |
|  |  |  | (*e*) Describe any sensitivity analyses |  |
| Results | | | | |
| Participants | 13* | (a) Report numbers of individuals at each stage of study—eg numbers potentially eligible, examined for eligibility, confirmed eligible, included in the study, completing follow-up, and analysed | | N/A |
|  |  | (b) Give reasons for non-participation at each stage | |  |
|  |  | (c) Consider use of a flow diagram | |  |
| Descriptive data | 14* | (a) Give characteristics of study participants (eg demographic, clinical, social) and information on exposures and potential confounders | | 11-12 |
|  |  | (b) Indicate number of participants with missing data for each variable of interest | | N/A |
|  |  | (c) *Cohort study*—Summarise follow-up time (eg, average and total amount) | |  |
| Outcome data | 15* | *Cohort study*—Report numbers of outcome events or summary measures over time | |  |
|  |  | *Case-control study—*Report numbers in each exposure category, or summary measures of exposure | |  |
|  |  | *Cross-sectional study—*Report numbers of outcome events or summary measures | | *11-12* |
| Main results | 16 | (*a*) Give unadjusted estimates and, if applicable, confounder-adjusted estimates and their precision (eg, 95% confidence interval). Make clear which confounders were adjusted for and why they were included | | 11-14 |
|  |  | (*b*) Report category boundaries when continuous variables were categorized | |  |
|  |  | (*c*) If relevant, consider translating estimates of relative risk into absolute risk for a meaningful time period | |  |
| Other analyses | 17 | Report other analyses done—eg analyses of subgroups and interactions, and sensitivity analyses | | N/A |
| Discussion | | | | |
| Key results | 18 | Summarise key results with reference to study objectives | | 15 |
| Limitations | 19 | Discuss limitations of the study, taking into account sources of potential bias or imprecision. Discuss both direction and magnitude of any potential bias | | 18 |
| Interpretation | 20 | Give a cautious overall interpretation of results considering objectives, limitations, multiplicity of analyses, results from similar studies, and other relevant evidence | | 18 |
| Generalisability | 21 | Discuss the generalisability (external validity) of the study results | | 18 |
| Other information | | | | |
| Funding | 22 | Give the source of funding and the role of the funders for the present study and, if applicable, for the original study on which the present article is based | | 18 |

**Methods**

To study the effect on the mental health of university students, a protocol was assembled to gather demographic data and also data pertaining to general health, previous psychiatric history, current symptoms of anxiety (STAI-Y1 state) ^2^, depression (CES-D) ^3^ and suicidality (RASS), ^4^ as well as a detailed protocol to investigate changes because of the lockdown in sleep, sex, family relationships, finance, eating and exercising and religion/spirituality. Additionally, the beliefs concerning the COVID-19 outbreak, including the measures taken and conspiracy theories, were investigated.

The full protocol is shown in the part 2 of the webappendix.

Each question of the protocol was given an ID code, reflecting the part of the protocol it belongs to with a capital letter and a number to denote its position within that protocol part. Throughout the results these ID codes are used for increased accuracy.

According to a previously developed method, ^3,4^ the cut-off score 23/24 for the CES-D score and a previously derived algorithm were used to identify cases of probable clinical major depression. Cases of clinical depression were considered those positively identified by both methods. Those identified by only one of them, were considered to be cases of distress (false positive cases in terms of depression).

The data were collected online and anonymously from January 2020 to May 2021, covering periods of full implementation of lockdowns as well as of relaxations of measures in countries around the world. Announcements and advertisements were done in the social media and through news sites, but no other organized effort had been undertaken. The first page included a declaration of consent which everybody accepted by continuing with the participation.

Approval was initially given by the Ethics Committee of the Faculty of Medicine, Aristotle University of Thessaloniki, Greece and locally concerning each participating country.

**Materials and Populations**

The study sample included:

- 9026 females (72.27 %; aged 21.11±2.53)
- 3329 males (26.65%; aged 21.61±2.81)
- 133 ‘non-binary gender’ (1.06%; aged 21.02±2.98)

**Webtable 1:** In terms of country of origin, they were as follows

| **A1. Country** | **Ν** | **%** | | | |
| --- | --- | --- | --- | --- | --- |
|  |  | **Total** | **A2. Sex** | | |
|  |  |  | **Females** | **Males** | **Non-binary gender** |
| **Bulgaria** | 266 | 2.13 | 60.90 | 38.35 | 0.75 |
| **Croatia** | 1560 | 12.49 | 80.38 | 19.04 | 0.58 |
| **Georgia** | 984 | 7.88 | 70.93 | 28.46 | 0.61 |
| **Greece** | 5456 | 43.69 | 72.10 | 26.63 | 1.26 |
| **Hungary** | 87 | 0.70 | 81.61 | 18.39 | 0.00 |
| **India** | 521 | 4.17 | 58.93 | 40.88 | 0.19 |
| **Latvia** | 1047 | 8.38 | 79.08 | 20.25 | 0.67 |
| **Lithuania** | 335 | 2.68 | 82.69 | 16.42 | 0.90 |
| **Malaysia** | 218 | 1.75 | 82.11 | 16.97 | 0.92 |
| **Nigeria** | 587 | 4.70 | 44.63 | 55.20 | 0.17 |
| **Russia** | 1427 | 11.43 | 73.86 | 23.83 | 2.31 |
| **total** | 12488 | 100.00 | 72.28 | 26.66 | 1.07 |

**Webtable 2:** In terms of type of studies they were as follows

| **Α8. Field of studies** | **Group** | **N** | **%** |
| --- | --- | --- | --- |
| Aeronautics and Aviation science | B | 31 | 0.25 |
| Agriculture. Geoponic and Fishery Science | B | 437 | 3.50 |
| Anthropology | A | 15 | 0.12 |
| Architecture. Design and Planning | B | 323 | 2.59 |
| Athletics. Sports science and Physical Education | A | 182 | 1.46 |
| Automotive Engineering | B | 5 | 0.04 |
| Biology | A | 309 | 2.47 |
| Business Administration (Accounting. Management. Finance. Marketing) | B | 554 | 4.44 |
| Chemistry | B | 278 | 2.23 |
| Civil Engineering | B | 240 | 1.92 |
| Computer Science / Informatics | B | 432 | 3.46 |
| Dentistry | A | 363 | 2.91 |
| Earth sciences / Geology / Soil Sciences | B | 12 | 0.10 |
| Economics | B | 623 | 4.99 |
| Electrical and Electronic engineering | B | 302 | 2.42 |
| Fine arts (visual arts. performing arts. culinary art) | C | 322 | 2.58 |
| Foreign languages. Translation and Interpretation | C | 162 | 1.30 |
| Forestry and Natural Environment studies | B | 103 | 0.82 |
| Geography | B | 33 | 0.26 |
| History and Archaeology | C | 184 | 1.47 |
| Information Systems. Library and Museum studies | B | 45 | 0.36 |
| Journalism. mass media and communication | C | 245 | 1.96 |
| Languages. Literature & Linguistics | C | 502 | 4.02 |
| Law | C | 446 | 3.57 |
| Maritime studies | B | 5 | 0.04 |
| Mathematics and Statistics | B | 215 | 1.72 |
| Mechanical engineering and Materials science | B | 150 | 1.20 |
| Medicine | A | 1983 | 15.88 |
| Military training | B | 14 | 0.11 |
| Nursing and Midwifery | A | 218 | 1.75 |
| Nutrition and Dietetics | A | 17 | 0.14 |
| Occupational therapy | A | 1 | 0.01 |
| Pharmaceutical sciences | A | 287 | 2.30 |
| Philosophy | C | 114 | 0.91 |
| Physical and Chemical engineering | B | 117 | 0.94 |
| Physics | B | 252 | 2.02 |
| Physiotherapy | A | 11 | 0.09 |
| Political Science and International Relations | C | 161 | 1.29 |
| Psychology | A | 561 | 4.49 |
| Public relations and Administration | C | 88 | 0.70 |
| Religion studies/Theology | C | 174 | 1.39 |
| Social work | C | 26 | 0.21 |
| Sociology/Contemporary Social sciences | C | 88 | 0.70 |
| Space sciences and cosmic research | B | 12 | 0.10 |
| Speech and language therapy | A | 5 | 0.04 |
| Teaching and Education | C | 460 | 3.68 |
| Transportation studies | B | 14 | 0.11 |
| Veterinary medicine | A | 241 | 1.93 |
| Other | C | 1126 | 9.02 |
| **Total** |  | 12488 | 100.00 |

**Statistical Analysis**

- Descriptive tables were created for the variables under investigation.
- Chi-square tests were used for the comparison of frequencies when categorical variables were present and for the post hoc analysis of the results a Bonferroni-corrected method of pair-wise comparisons was utilized ^5^.
- Pearson Product Moment Correlation Coefficient (R) to investigate the relationship among variables
- Analysis of Covariance (ANCOVA) was used to test for the main effect as well as the interaction among categorical variables, with Schefee as post hoc test to investigate which variables could contribute to the development of others.
- Multiple forward stepwise linear regression analysis (MFSLRA) was performed to investigate which variables could contribute to the development of others.
- Relative Risk (RR) was calculated as the ratio of the incidence in two groups under comparison.

**PART 2: The study protocol**

**Α. GENERAL DATA**

**A1. Country**

**A2. Sex**

- Male
- Female
- Non-binary gender/I do not wish to define

**A3. Date of Birth written with 4 digits (e.g. 1982) ***

Please enter your date of birth in the form of 4 digits (e.g. 1981, 1968, 1993 etc.)

**A4. Place of residence during this period**

- Capital City
- City > 1 million population
- City (100.000 – 1 million population)
- Town (20.000 – 100.000 inhabitants)
- Town (<20.000 inhabitants)
- Rural area - Village

**A5. Marital Status**

- Single
- Married (or in a civil partnership)
- Divorced (or estranged)
- Live with someone without an official relationship
- Widower
- Other

**A6. How many people reside in the house you are staying in during this period (including yourself)?**

- 1 (I live alone)
- 2 people
- 3 people
- 4 people
- 5 or more people

**A7. How many children do you have (regardless of whether they live in the same house with you or not)?**

- 0 (I do not have any children)
- 1 child
- 2 children
- 3 children
- 4 or more children

**A8. Field of studies**

- Aeronautics and Aviation science
- Agriculture, Geoponic and Fishery sciences
- Anthropology
- Architecture, Design and Planning
- Athletics, Sports science and Physical education
- Automotive engineering
- Biology
- Business Administration (Accounting, Management, Finance, Marketing)
- Chemistry
- Civil engineering
- Computer sciences / Informatics
- Dentistry
- Earth sciences / Geology / Soil sciences
- Economics
- Electrical and Electronic engineering
- Fine Arts (visual arts, performing arts, culinary art)
- Foreign languages, Translation and Interpretation
- Forestry and Natural Environment studies
- Geography
- History and Archaeology
- Information Systems, Library and Museum studies
- Journalism, mass media and communication
- Law
- Maritime studies
- Mathematics and Statistics
- Mechanical engineering and Materials science
- Medicine
- Military training
- Nursing and Midwifery
- Nutrition and Dietetics
- Occupational therapy
- Pharmaceutical sciences
- Philology and Literature
- Philosophy
- Physical and Chemical engineering
- Physics
- Physiotherapy
- Political sciences and International relations
- Psychology
- Public relations and Administration
- Religion studies / Theology
- Social work
- Sociology / Contemporary Social sciences
- Space sciences and cosmic research
- Speech and language therapy
- Teaching and Education
- Transportation studies
- Veterinary medicine
- Other

**A9. Employment**

- I am working
- I am not working

**A10. Did you continue to exercise your profession during the period of the lockdown?**

- Yes
- No

**B. GENERAL STATE OF HEALTH AND COVID-19**

**B1. In general, your health over the last month can be described as:**

- Excellent
- Very good
- Good
- Moderate
- Bad

**B2. Do you suffer from any chronic medical condition (for example: diabetes mellitus, hypertension, asthma, etc.)?**

- Yes
- No

**B3. If yes, please define your chronic medical condition. If no, please write the word “No”.**

**B4. Are you a close relative or caretaker of a person that belongs to a vulnerable group?**

- Yes
- No

**B5. In the past did you have any mental health problem serious enough to make you seek professional health, psychotherapy or medication treatment?**

- No
- Anxiety
- Depression
- Psychosis
- Bipolar Disorder
- Other

**B6. Are you currently under any kind of treatment for your mental state? (please check all of those that are true)**

- No
- Psychotherapy
- Antipsychotics
- Antidepressants
- Antiepileptics/mood stabilizers
- Lithium
- Tranquilizers**/**benzodiazepines (lexotanil, xanax, tavor, etc.)

**C. THOUGHTS ABOUT COVID-19**

**C1. Are you afraid that you will contract the coronavirus?**

- Never
- A little
- Moderately
- Much
- Very Much

**C2. Do you believe that the precautions work effectively or that if you are about to contract the disease, you will contract it anyway?**

- Precautions work effectively.
- Precautions cannot protect you.

**C3. Does the possibility that a member of your family could contract the coronavirus and die because of it, makes you frightened?**

- Never
- A little
- Moderately
- Much
- Very Much

**C4. Are you afraid that in case you contract the coronavirus, some people will step away from your life and behave to you in a different way later?**

- Never
- A little
- Moderately
- Much
- Very Much

**D. THOUGHTS ABOUT THE MEASURES TAKEN (PRECAUTIONS, LOCKDOWN, ETC.)**

**D1. According to your opinion, the amount of time that you spend outside of your house for reasons not regarding your work during this period is:**

- Minimum
- Less than humanly necessary
- Moderate**/**reasonable
- Enough
- A lot
- Excessive

**D2. Are you currently locked up in the house?**

- Not at all
- Partially
- To a high degree
- Completely

**D3. According to the instructions given by WHO, it is necessary for some precautions to be taken in order to prevent the spread of the virus. Do you believe that you take adequate precautions?**

- Not at all
- A little bit
- Moderately
- Much
- Very Much

**D4. Do you believe that that you have received sufficient information about the necessity of the measures taken?**

- Not at all
- A little bit
- Moderately
- Much
- Very Much

**E. FAMILY**

**E1. Do you feel the need to communicate with** **other members of your family during this period?**

- Much less
- Less
- Same
- More
- Much more

**E2. Do you want to receive emotional support from other members of your family during this period?**

- Much less
- Less
- Same
- More
- Much more

**E3. Are there any conflicts with the rest of your family members during this period?**

- Much less
- Less
- Same
- More
- Much more

**E4. Has the overall quality of relationships with the other members of your family changed compared to before the COVID – 19?**

- Much worse
- Worse
- It has not changed
- A little bit better
- Much better

**E5. Do you manage to maintain a basic daily routine (waking up in the morning, regular meals and sleeping hours, activities) both yourself (if you live alone) or as a family?**

- Not at all
- Somehow, but not always
- Generally, yes
- Clearly follow (or adhere to) a routine

**E6. If you have children, how difficult is it to manage their daily life and behavior?**

- I do not have children
- Much more difficult than before
- Somehow more difficult but not always
- Same as always
- Somehow easier but not always
- Much easier than before

**E7. How are your finances as a result of the outbreak?**

- Much more difficult than before
- Somehow more difficult
- Same as always
- Somehow easier
- Much easier than before

**F. ANXIETY**

**F1. I feel calm.**

- Not at all
- Somewhat
- Moderately so
- Very much so

**F2. I feel secure.**

- Not at all
- Somewhat
- Moderately so
- Very much so

**F3. I am tense.**

- Not at all
- Somewhat
- Moderately so
- Very much so

**F4. I feel strained.**

- Not at all
- Somewhat
- Moderately so
- Very much so

**F5. I feel at ease.**

- Not at all
- Somewhat
- Moderately so
- Very much so

**F6. I feel upset.**

- Not at all
- Somewhat
- Moderately so
- Very much so

**F7. I am presently worrying over possible misfortunes.**

- Not at all
- Somewhat
- Moderately so
- Very much so

**F8. I feel satisfied.**

- Not at all
- Somewhat
- Moderately so
- Very much so

**F9. I feel frightened.**

- Not at all
- Somewhat
- Moderately so
- Very much so

**F10. I am comfortable.**

- Not at all
- Somewhat
- Moderately so
- Very much so

**F11. I have self-confidence**

- Not at all
- Somewhat
- Moderately so
- Very much so

**F12. I feel nervous.**

- Not at all
- Somewhat
- Moderately so
- Very much so

**F13. I feel jittery.**

- Not at all
- Somewhat
- Moderately so
- Very much so

**F14. I am indecisive.**

- Not at all
- Somewhat
- Moderately so
- Very much so

**F15. I am relaxed.**

- Not at all
- Somewhat
- Moderately so
- Very much so

**F16. I feel content.**

- Not at all
- Somewhat
- Moderately so
- Very much so

**F17. I feel worried.**

- Not at all
- Somewhat
- Moderately so
- Very much so

**F18. I feel confused.**

- Not at all
- Somewhat
- Moderately so
- Very much so

**F19. I feel steady.**

- Not at all
- Somewhat
- Moderately so
- Very much so

**F20. I feel pleasant.**

- Not at all
- Somewhat
- Moderately so
- Very much so

**F21. How much has your emotional state changed in relation to the appearance of anxiety and insecurity, compared to before the COVID-19 epidemic?**

- It got a lot worse
- It got a little worse
- Neither better nor worse
- It got a little improved
- It got improved a lot

**G. SADNESS & DEPRESSION/MELANCHOLY**

Please select what you think corresponds to how you felt during the last week.

**G1. I was bothered by things that usually don’t bother me.**

- Rarely or none of the time (less than 1 day)
- Some of a little of the time (1-2 days)
- Occasionally or a moderate amount of time (3-4 days)
- Most or all of the time (5-7 days)

**G2. I did not feel like eating; my appetite was poor.**

- Rarely or none of the time (less than 1 day)
- Some of a little of the time (1-2 days)
- Occasionally or a moderate amount of time (3-4 days)
- Most or all of the time (5-7 days)

**G3. I felt that I could not shake off the blues even with help from my family or friends.**

- Rarely or none of the time (less than 1 day)
- Some of a little of the time (1-2 days)
- Occasionally or a moderate amount of time (3-4 days)
- Most or all of the time (5-7 days)

**G4. I felt I was just as good as other people.**

- Rarely or none of the time (less than 1 day)
- Some of a little of the time (1-2 days)
- Occasionally or a moderate amount of time (3-4 days)
- Most or all of the time (5-7 days)

**G5. I had trouble keeping my mind on what I was doing.**

- Rarely or none of the time (less than 1 day)
- Some of a little of the time (1-2 days)
- Occasionally or a moderate amount of time (3-4 days)
- Most or all of the time (5-7 days)

**G6. I felt depressed.**

- Rarely or none of the time (less than 1 day)
- Some of a little of the time (1-2 days)
- Occasionally or a moderate amount of time (3-4 days)
- Most or all of the time (5-7 days)

**G7. I felt that everything I did was an effort.**

- Rarely or none of the time (less than 1 day)
- Some of a little of the time (1-2 days)
- Occasionally or a moderate amount of time (3-4 days)
- Most or all of the time (5-7 days)

**G8. I felt full of hope about the future.**

- Rarely or none of the time (less than 1 day)
- Some of a little of the time (1-2 days)
- Occasionally or a moderate amount of time (3-4 days)
- Most or all of the time (5-7 days)

**G9. I thought my life had been a failure.**

- Rarely or none of the time (less than 1 day)
- Some of a little of the time (1-2 days)
- Occasionally or a moderate amount of time (3-4 days)
- Most or all of the time (5-7 days)

**G10. I felt fearful.**

- Rarely or none of the time (less than 1 day)
- Some of a little of the time (1-2 days)
- Occasionally or a moderate amount of time (3-4 days)
- Most or all of the time (5-7 days)

**G11. My sleep was restless.**

- Rarely or none of the time (less than 1 day)
- Some of a little of the time (1-2 days)
- Occasionally or a moderate amount of time (3-4 days)
- Most or all of the time (5-7 days)

**G12. I was happy.**

- Rarely or none of the time (less than 1 day)
- Some of a little of the time (1-2 days)
- Occasionally or a moderate amount of time (3-4 days)
- Most or all of the time (5-7 days)

**G13. I talked less than usual.**

- Rarely or none of the time (less than 1 day)
- Some of a little of the time (1-2 days)
- Occasionally or a moderate amount of time (3-4 days)
- Most or all of the time (5-7 days)

**G14. I felt lonely.**

- Rarely or none of the time (less than 1 day)
- Some of a little of the time (1-2 days)
- Occasionally or a moderate amount of time (3-4 days)
- Most or all of the time (5-7 days)

**G15. People were unfriendly.**

- Rarely or none of the time (less than 1 day)
- Some of a little of the time (1-2 days)
- Occasionally or a moderate amount of time (3-4 days)
- Most or all of the time (5-7 days)

**G16. I enjoyed life.**

- Rarely or none of the time (less than 1 day)
- Some of a little of the time (1-2 days)
- Occasionally or a moderate amount of time (3-4 days)
- Most or all of the time (5-7 days)

**G17. I had crying spells.**

- Rarely or none of the time (less than 1 day)
- Some of a little of the time (1-2 days)
- Occasionally or a moderate amount of time (3-4 days)
- Most or all of the time (5-7 days)

**G18. I felt sad.**

- Rarely or none of the time (less than 1 day)
- Some of a little of the time (1-2 days)
- Occasionally or a moderate amount of time (3-4 days)
- Most or all of the time (5-7 days)

**G19. I felt that people disliked me.**

- Rarely or none of the time (less than 1 day)
- Some of a little of the time (1-2 days)
- Occasionally or a moderate amount of time (3-4 days)
- Most or all of the time (5-7 days)

**G20. I could not get “going”.**

- Rarely or none of the time (less than 1 day)
- Some of a little of the time (1-2 days)
- Occasionally or a moderate amount of time (3-4 days)
- Most or all of the time (5-7 days)

**G21. How much has your emotional state related to the experience of joy or melancholy changed in comparison to before the COVID-19 epidemic?**

- It got a lot worse
- It got a little worse
- Neither better nor worse
- Improved a bit
- It has improved a lot

**H. PHYSICAL ACTIVITY**

**H1. Does exercise help you at the prevention of anxiety?**

- Not at all
- A little bit
- Moderately
- Much
- Very much

**H2. Do you consider that exercise is important during this epidemic?**

- Not at all
- A little bit
- Moderately
- Much
- Very much

**H3. Do you have increased the frequency and intensity of your physical workout during this epidemic and lockdown?**

- Not at all
- A little bit
- Moderately
- Much
- Very much

**H4. How much has your physical activity been affected by this epidemic of COVID-19?**

- It decreased much
- It decreased a little
- Neither decreased, nor increased
- It increased a little
- It increased much

**I. NUTRITION**

Please answer the following questions considering the period of the COVID-19 outbreak.

**I1. Did you notice the need to eat larger amounts of food or eat more often?**

- I eat much less than I used to
- I eat bit less than I used to
- Neither more nor less
- I eat a bit more than I used to
- I eat much more than I used to

**I2. Please mark the answer that best represents you during this period:**

- I eat in a healthier way.
- My eating habits and preferences have not changed.
- I eat in a more unhealthy way.

**I3. Please mark the answer that best represents you during this period (you can choose more than one answers):**

- My body weight has significantly decreased (more than 2-3 kilos).
- My body weight has slightly decreased (less than 2 kilos lost).
- My body weight is stable.
- My body weight has slightly increased (less than 2 kilos put).
- My body weight has significantly increased (more than 2-3 kilos).

**J. OPINIONS ABOUT THE ORIGIN OF THE EPIDEMIC AND OTHER HEALTH AND PUBLIC ISSUES**

Answer according to how much you agree with the following statements.

**J1. Do you believe that the COVID-19 vaccine was ready even before the virus broke out and they conceal it from us for the benefit of pharmaceutical companies?**

- I don’t believe it at all
- A little bit
- Maybe
- Much
- Very much

**J2. Do you believe that COVID-19 was created in a laboratory?**

- I don’t believe it at all
- A little bit
- Maybe
- Much
- Very much

**J3. Do you think that COVID-19 was created to be used as a biochemical weapon for the extermination of the human population?**

- I don’t believe it at all
- A little bit
- Maybe
- Much
- Very much

**J4. Do you believe that COVID-19 is related to the 5G technology antenna?**

- I don’t believe it at all
- A little bit
- Maybe
- Much
- Very much

**J5. Do you believe that COVID-19 appeared accidentally from human contact with animals and it was something that generally happens and was generally expected?**

- I don’t believe it at all
- A little bit
- Maybe
- Much
- Very much

**J6. Do you believe that COVID-19 has much lower mortality rate but there is misinformation and terror-inducing propaganda?**

- I don’t believe it at all
- A little bit
- Maybe
- Much
- Very much

**J7. Do you think the recommended measures (e.g. wearing face masks, avoid gatherings, stay at home etc.) are an attempt to restrict human rights and lead to some kind of dictatorship rather than to keep the population safer from COVID-19?**

- I don’t believe it at all
- A little bit
- Maybe
- Much
- Very much

**J8. Do you believe that COVID-19 outbreak is a deliberate creation of the world’s powerful leaders to create a global economic crisis?**

- I don’t believe it at all
- A little bit
- Maybe
- Much
- Very much

**J9. Do you believe that COVID-19 is a sign of divine power to destroy our planet?**

- I don’t believe it at all
- A little bit
- Maybe
- Much
- Very much

**J10. Do you believe that airplanes secretly spray people with various chemicals?**

- I don’t believe it at all
- A little bit
- Maybe
- Much
- Very much

**J11. Do you think that vaccines in general are dangerous and should be avoided?**

- I don’t believe it at all
- A little bit
- Maybe
- Much
- Very much

**J12. The government is secretly involved in the murder of innocent citizens and/or well-known public figures.**

- I don’t believe it at all
- A little bit
- Maybe
- Much
- Very much

**J13. Global warming and climate change is a greatly exaggerated myth to serve various political and financial interests.**

- I don’t believe it at all
- A little bit
- Maybe
- Much
- Very much

**J14. The power held by the heads of state is smaller than that of small unknown groups that really control the world of politics.**

- I don’t believe it at all
- A little bit
- Maybe
- Much
- Very much

**J15. Secret organizations are communicating with aliens, but they hide it from the public.**

- I don’t believe it at all
- A little bit
- Maybe
- Much
- Very much

**J16. Groups of scientists manipulate, invent or conceal evidence to deceive the public.**

- I don’t believe it at all
- A little bit
- Maybe
- Much
- Very much

**J17. The government allows or commits acts of terrorism on its territory, disguising its involvement as if someone else is responsible.**

- I don’t believe it at all
- A little bit
- Maybe
- Much
- Very much

**J18. Do you believe that secretly a chip will be included in the COVID-19 vaccine in order to mark people?**

- I don’t believe it at all
- A little bit
- Maybe
- Much
- Very much

**J19. A small, secret group of people is responsible for taking all the important decisions, such as starting wars, in a planned way and the reasons are the group’s secret interests.**

- I don’t believe it at all
- A little bit
- Maybe
- Much
- Very much

**J20. Technology and devices for mind control are used on people without their knowledge.**

- I don’t believe it at all
- A little bit
- Maybe
- Much
- Very much

**J21. New and advanced technology that will make the existing industry obsolete is being suppressed in a malicious and violent way.**

- I don’t believe it at all
- A little bit
- Maybe
- Much
- Very much

**J22. Some important events happen due to the activity of a small group who secretly manipulate world events.**

- I don’t believe it at all
- A little bit
- Maybe
- Much
- Very much

**J23. Experiments involving new drugs or technologies are performed systematically on humans in a secret way and without their knowledge or consent.**

- I don’t believe it at all
- A little bit
- Maybe
- Much
- Very much

**J24. Many important pieces of information are deliberately hidden from the public for reasons of interest.**

- I don’t believe it at all
- A little bit
- Maybe
- Much
- Very much

**J25. The spread of certain viruses and/or diseases is the result of deliberate, covert actions of an organization or government.**

- I don’t believe it at all
- A little bit
- Maybe
- Much
- Very much

**J26. It is possible that the earth is flat rather than spherical.**

- I don’t believe it at all
- A little bit
- Maybe
- Much
- Very much

**K. INTERNET**

**K1. The information and use of the internet worry me about the issue regarding the COVID-19:**

- Not at all
- A little
- Moderately
- Much
- Very much

**K2. Generally, most of the internet sources regarding information about COVID-19 are misinforming/misleading:**

- Not at all
- A little
- Moderately
- Much
- Very much

**K3. Due to the epidemic conditions, the internet takes up more of my time than usual:**

- Not at all
- A little more
- Moderately more
- Much more
- Too much

**K4. How much do you use the social media while in isolation at home?**

- More than before
- The same as before
- Less than before

**K5. Have you acquired internet-related habits that you did not have before (for example: created a facebook account, engaging in cybersex or gambling)?**

- Yes
- No

**L. SLEEP DURING THIS PERIOD**

**L1. The quality of my sleep has changed recently. It is:**

- Much worse
- A little bit worse
- The same (neither worse nor better)
- A little better
- Much better

**L2. I tend to stay up late and sleep for many hours during the day.**

- Almost never
- Rarely
- Sometimes
- Often
- Almost always

**L3. I take sleeping pills to help me sleep at night.**

- Almost never
- Rarely
- Some times
- Often
- Almost always

**L4. I am having dreams in which I feel trapped, over the last 3 weeks.**

- Almost never
- Rarely
- Some times
- Often
- Almost always

**M.** **SMOKING, ALCOHOL AND SUBSTANCES USE**

**M1. Smoking before the epidemic:**

- - I didn't smoke
  - I was smoking

**M2. Alcohol use before the epidemic:**

- - I did not drink much
  - I drank a lot (more than one drink or its equivalent every day)

**M3. Use of substances before the epidemic (e.g. hashish, drugs):**

- - I did not use it
  - Occasionally and rather rarely
  - Often

**M4. During this period, how much do you smoke compared to before?**

- - More than before
  - Same as before
  - Less than before

**M5. During this period, how much alcohol do you drink compared to before?**

- - More than before
  - Same as before
  - Less than before

**M6. During this period, how much do you use illegal substances compared to before?**

- - More than before
  - Same as before
  - Less than before

**N. SEXUALITY**

**N1. How would you characterize the frequency of your sexual intercourse?**

- Clearly inadequate
- Rather inadequate
- Neutral
- Rather satisfactory
- Clearly satisfactory

**N2. Has your desire for sexual intercourse increased or decreased due to the epidemic?**

- It has decreased a lot
- It has decreased slightly
- It remains the same
- It has increased slightly
- It has increased a lot

**N3. How much pleasure and satisfaction do you get from your current sex life?**

- Not at all
- A little bit
- Moderate
- Much
- Very much

**N4. Do you think sex helps you deal with your daily stress and anxiety?**

- Not at all
- A little bit
- Moderate
- Much
- Very much

**O. THOUGHTS ABOUT DEATH**

**O1. Are you afraid that you are going to die?**

- Not at all
- A little bit
- Much
- Very much

**O2. Do you ever think that it would be better if you were dead?**

- Not at all
- A little bit
- Much
- Very much

**O3. Do you think that it is a wonderful thing that you are alive?**

- Not at all
- A little bit
- Much
- Very much

**O4. Have you ever felt that it’s not worth living?**

- Not at all
- A little bit
- Much
- Very much

**O5. Do you think of harming yourself physically?**

- Not at all
- A little bit
- Much
- Very much

**O6. Do you often think of committing suicide if you have the chance?**

- Not at all
- A little bit
- Much
- Very much

**O7. Do you make plans concerning the method to use in order to end your life?**

- Not at all
- A little bit
- Much
- Very much

**O8. I am thinking of committing suicide, but I won’t do it.**

- Not at all
- A little bit
- Much
- Very much

**O9. Do you enjoy your life?**

- Not at all
- A little bit
- Much
- Very much

**O10. Are you feeling tired from your life?**

- Not at all
- A little bit
- Much
- Very much

**O11. How much has your tendency to think about death and/or suicide changed, compared to before the outbreak of COVID-19?**

- Very much increased
- Increased a bit
- Neither increased, nor decreased
- Decreased a bit
- Very much decreased

**O12. Have you ever hurt yourself in any way deliberately, during your whole life so far?**

- Never
- Once
- 2-3 times
- Many times

**O13. Have you ever attempted suicide, during your whole life so far?**

- Never
- Once
- 2-3 times
- Many times

**P. SPIRITUALITY AND PSYCHOLOGICAL REFECTIONS**

**P1. Over the last 2-3 weeks, have your religious/spiritual inquiries been increased?**

- Not at all.
- A little bit
- Much
- Very Much

**PART 3: Results**

***3.1 Demographics***

Eleven countries participated in the study and data from 13354 persons were initially gathered.

Only data from those with age between 17 and 30 years were kept and thus, the sample included 12488 subjects

Of these, 9026 were females (72.27 %; aged 21.11±2.53), 3329 males (26.65%; aged 21.61±2.81) and 133 ‘non-binary gender’ (1.06%; aged 21.02±2.98)

The sample composition in terms country of origin (A1) by sex (A2) and of field of studies (Α8) is shown in WebTables 1 and 2.

Subjects were classified, depending on their studies, into three groups: group A (health and biological sciences), group B (technical sciences) and group C (arts, literature, education and related sciences). Psychology, anthropology, various therapies and athletics were included in group A, Economics in group B, social workers and social sciences and ‘non-binary gender’ in group C. The size and composition of each group is shown in table 1 and WebTable 2. Each of the three groups accounted for roughly one-third of the study sample with the percentage of males being double in group B in comparison to groups A and C.

The fact that the majority were females reflects a common phenomenon in this kind of studies with online gathering of data and self-selection of participation. This means that results should be calculated separately for males and females.

The study population was self-selected. It was not possible to apply post-stratification on the sample as it was done in a previous study ^6^, because this would mean that we would utilize a similar methodology across much different countries and the population data needed were not available for all.

22.36% reported that their current physical health was poor or very poor (B1)

10.05% of the study sample were suffering from a chronic medical condition (B2).

54.58% them had a person belonging to a vulnerable group in the family (B4)

26.51% reported any kind of mental health history (webtable 3)

***3.2 History of mental disorder and self-injury or suicidal attempt***

| **History of:** | **N** | **%** |
| --- | --- | --- |
| **Any history of mental disorder** | 3258 | 26.09 |
| **No history any mental disorder** | 9230 | 73.91 |
|  |  |  |
| **Anxiety** | 1210 | 9.69 |
| **Depression** | 1365 | 10.93 |
| **Bipolar Disorder** | 128 | 1.02 |
| **Psychosis** | 125 | 1.00 |
| **Self-harm (at least once)** | 3175 | 25.43 |
| **Suicide attempt (at least once)** | 904 | 7.24 |
| **Other** | 430 | 3.44 |

**Webtable 3:** Rates of history of mental health in the study sample

|  |  | **No history any mental disorder** | **Any history of mental disorder** | **Anxiety** | **Depression** | **Bipolar**  **Disorder** | **Psychosis** | **Other** | **Self-**  **Injury**  **(O12)** | **Suicidal**  **Attempt**  **(O13)** |
| --- | --- | --- | --- | --- | --- | --- | --- | --- | --- | --- |
| **Females** | **A** | 75.02 | 24.98 | 8.95 | 10.76 | 0.83 | 0.77 | 3.68 | 26.52 | 8.06 |
|  | **B** | 71.92 | 28.08 | 11.30 | 11.54 | 1.18 | 1.10 | 2.95 | 25.36 | 6.46 |
|  | **C** | 67.69 | 32.31 | 13.27 | 12.59 | 1.02 | 0.96 | 4.47 | 30.09 | 7.91 |
|  | **Total** | 71.53 | 28.47 | 11.16 | 11.63 | 1.00 | 0.93 | 3.76 | 27.47 | 7.56 |
| **Males** | **A** | 80.86 | 19.14 | 5.41 | 8.90 | 1.01 | 1.35 | 2.48 | 21.51 | 6.98 |
|  | **B** | 80.52 | 19.48 | 6.14 | 8.93 | 1.12 | 0.99 | 2.30 | 17.87 | 5.77 |
|  | **C** | 82.15 | 17.85 | 4.95 | 7.96 | 1.09 | 1.09 | 2.77 | 18.82 | 5.55 |
|  | **Total** | 81.02 | 18.98 | 5.65 | 8.68 | 1.08 | 1.11 | 2.46 | 19.07 | 6.04 |
| **Non-binary gender** | **A** | 53.49 | 46.51 | 13.95 | 18.60 | 0.00 | 6.98 | 6.98 | 46.51 | 25.58 |
|  | **B** | 56.52 | 43.48 | 8.70 | 17.39 | 4.35 | 2.17 | 10.87 | 44.44 | 13.33 |
|  | **C** | 63.64 | 36.36 | 11.36 | 22.73 | 0.00 | 0.00 | 2.27 | 47.73 | 9.09 |
|  | **Total** | 57.89 | 42.11 | 11.28 | 19.55 | 1.50 | 3.01 | 6.77 | 46.21 | 15.91 |
| **TOTAL** |  | 73.91 | 26.09 | 9.69 | 10.93 | 1.02 | 1.00 | 3.44 | 25.43 | 7.24 |

**Webtable 4:** Rates of history of mental health by sex and type of studies

The rate of females to males for history of any mental disorders was 1.5:1 and the rate of ‘non-binary gender’ was 1.48 vs. females and 2.22 vs. males

There was a difference among sexes by type of studies concerning any mental history. In Females type C had the highest rate followed by type B while in males and non-binary gender, the sequence was the reverse.

| **Sex** | **Never** | | **Once** | | **2-3 times** | | **Many times** | | **At least once** | | **Total** |
| --- | --- | --- | --- | --- | --- | --- | --- | --- | --- | --- | --- |
|  | **N** | **%** | **N** | **%** | **N** | **%** | **N** | **%** | **N** | **%** | **N** |
| **History of self-injury behavior (O12)** | | | | | | | | | | | |
| Females | 6546 | 72.53 | 1028 | 11.39 | 881 | 9.76 | 570 | 6.32 | 2479 | 27.47 | 9025 |
| Males | 2694 | 80.93 | 290 | 8.71 | 228 | 6.85 | 117 | 3.51 | 635 | 19.07 | 3329 |
| Non-binary gender | 71 | 53.79 | 19 | 14.39 | 21 | 15.91 | 21 | 15.91 | 61 | 46.21 | 132 |
| All | 9311 | 74.57 | 1337 | 10.71 | 1130 | 9.05 | 708 | 5.67 | 3175 | 25.43 | 12486 |
| **History of suicidal attempt (O13)** | | | | | | | | | | | |
| Females | 8343 | 92.44 | 457 | 5.06 | 175 | 1.94 | 50 | 0.55 | 682 | 7.56 | 9025 |
| Males | 3128 | 93.96 | 139 | 4.18 | 47 | 1.41 | 15 | 0.45 | 201 | 6.04 | 3329 |
| Non-binary gender | 111 | 84.09 | 12 | 9.09 | 7 | 5.30 | 2 | 1.52 | 21 | 15.91 | 132 |
| All | 11582 | 92.76 | 608 | 4.87 | 229 | 1.83 | 67 | 0.54 | 904 | 7.24 | 12486 |

**Webtable 5:** rates of history of self-injury and suicidal attempt by sex

| **Studies group** | **Sex** | **Never** | | **Once** | | **2-3 times** | | **Many times** | | **At least once** | | **Total** |
| --- | --- | --- | --- | --- | --- | --- | --- | --- | --- | --- | --- | --- |
|  |  | **N** | **%** | **N** | **%** | **N** | **%** | **N** | **%** | **N** | **%** | **N** |
| **History of self-injury behavior** | | | | | | | | | | | | |
| **A** | Females | 2397 | 73.48 | 380 | 11.65 | 275 | 8.43 | 210 | 6.44 | 865 | 26.52 | 3262 |
|  | Males | 697 | 78.49 | 84 | 9.46 | 63 | 7.09 | 44 | 4.95 | 191 | 21.51 | 888 |
|  | Non-binary gender | 23 | 53.49 | 6 | 13.95 | 5 | 11.63 | 9 | 20.93 | 20 | 46.51 | 43 |
|  | All | 3117 | 74.34 | 470 | 11.21 | 343 | 8.18 | 263 | 6.27 | 1076 | ***25.66*** | 4193 |
| **B** | Females | 1895 | 74.64 | 257 | 10.12 | 243 | 9.57 | 144 | 5.67 | 644 | 25.36 | 2539 |
|  | Males | 1324 | 82.13 | 136 | 8.44 | 109 | 6.76 | 43 | 2.67 | 288 | 17.87 | 1612 |
|  | Non-binary gender | 25 | 55.56 | 9 | 20.00 | 6 | 13.33 | 5 | 11.11 | 20 | 44.44 | 45 |
|  | All | 3244 | 77.31 | 402 | 9.58 | 358 | 8.53 | 192 | 4.58 | 952 | ***22.69*** | 4196 |
| **C** | Females | 2254 | 69.91 | 391 | 12.13 | 363 | 11.26 | 216 | 6.70 | 970 | 30.09 | 3224 |
|  | Males | 673 | 81.18 | 70 | 8.44 | 56 | 6.76 | 30 | 3.62 | 156 | 18.82 | 829 |
|  | Non-binary gender | 23 | 52.27 | 4 | 9.09 | 10 | 22.73 | 7 | 15.91 | 21 | 47.73 | 44 |
|  | All | 2950 | 72.00 | 465 | 11.35 | 429 | 10.47 | 253 | 6.18 | 1147 | ***28.00*** | 4097 |
| **History of suicidal attempt** | | | | | | | | | | | | |
| **A** | Females | 2999 | 91.94 | 177 | 5.43 | 69 | 2.12 | 17 | 0.52 | 263 | 8.06 | 3262 |
|  | Males | 826 | 93.02 | 44 | 4.95 | 16 | 1.80 | 2 | 0.23 | 62 | 6.98 | 888 |
|  | Non-binary gender | 32 | 74.42 | 6 | 13.95 | 4 | 9.30 | 1 | 2.33 | 11 | 25.58 | 43 |
|  | All | 3857 | 91.99 | 227 | 5.41 | 89 | 2.12 | 20 | 0.48 | 336 | ***8.01*** | 4193 |
| **B** | Females | 2375 | 93.54 | 118 | 4.65 | 33 | 1.30 | 13 | 0.51 | 164 | 6.46 | 2539 |
|  | Males | 1519 | 94.23 | 66 | 4.09 | 21 | 1.30 | 6 | 0.37 | 93 | 5.77 | 1612 |
|  | Non-binary gender | 39 | 86.67 | 3 | 6.67 | 2 | 4.44 | 1 | 2.22 | 6 | 13.33 | 45 |
|  | All | 3933 | 93.73 | 187 | 4.46 | 56 | 1.33 | 20 | 0.48 | 263 | ***6.27*** | 4196 |
| **C** | Females | 2969 | 92.09 | 162 | 5.02 | 73 | 2.26 | 20 | 0.62 | 255 | 7.91 | 3224 |
|  | Males | 783 | 94.45 | 29 | 3.50 | 10 | 1.21 | 7 | 0.84 | 46 | 5.55 | 829 |
|  | Non-binary gender | 40 | 90.91 | 3 | 6.82 | 1 | 2.27 | 0 | 0.00 | 4 | 9.09 | 44 |
|  | All | 3792 | 92.56 | 194 | 4.74 | 84 | 2.05 | 27 | 0.66 | 305 | ***7.44*** | 4097 |

**Webtable 6:** Rates of history of self-injury and suicidal attempt by sex and type of studies

Males vs. females concerning

- history of self-injury: Chi-square=90.88, df=1, p<0.001
- history of suicidal attempts: Chi-square=7.38, df=1, p=0.006

Among types of studies by sex concerning

- history of self-injury: Chi-square=30.980, df=2, p<0.001

post-hoc chi-square tests:

A vs. B: Chi-square=10.16, df=1, p=0.001

A vs. C: Chi-square=5.754, df=1, p<0.016

B vs. C: Chi-square=30.893, df=1, p<0.001

Females only:

A vs. B: Chi-square=0.986, df=1, p=0.320

A vs. C: Chi-square=10.183, df=1, p=0.001

B vs. C: Chi-square=82.534, df=1, p<0.001

(higher rates in females of group C)

Males only:

A vs. B: Chi-square=4.906, df=1, p=0.026

A vs. C: Chi-square=1.926, df=1, p=0.165

B vs. C: Chi-square=0.333, df=1, p=0.563

(higher rates in males of group A)

The above suggest that the ranking of types of studies in terms of history of self-injury (C>A>B) is driven by females in group C and males in group A

- history of suicidal attempts: Chi-square=9.893, df=2, p=0.007

post-hoc chi-square tests:

A vs. B: Chi-square=9.637, df=1, p=0.002

A vs. C: Chi-square=0.940, df=1, p=332

B vs. C: Chi-square=4.498, df=1, p=0.034

Females only:

A vs. B: Chi-square=5382, df=1, p=0.020

A vs. C: Chi-square=0.052, df=1, p=0.820

B vs. C: Chi-square=4.431, df=1, p=0.035

(lower rates in females of group B)

Males only:

A vs. B: Chi-square=1.448, df=1, p=0.228

A vs. C: Chi-square=1.494, df=1, p=0.122

B vs. C: Chi-square=0.050, df=1, p=0.823

(no differences among males in the three groups)

The above suggest that the ranking of types of studies in terms of history of suicidal attempt (B<A=C) is driven by females in group B.

***3.3 Present mental status during the pandemic***

- Increased anxiety (at least ‘a little’) in approximately 60% (F21)
- More depressive feelings (at least ‘a little’) in > 55% (G21)
- Suicidal thoughts were increased (at least ‘a bit’) in 6.34% (O11)
- Clinical depression in 29.19% of females and 16.10% of males (25.81% of total sample)
- Dysphoria in an additional in 16.10% of females and 14.29% of males (15.66% of total sample)
- 17.63% reported that they were thinking of committing suicide (O5) with 5.97% reporting ‘much’ or ‘very much’

|  | **level** | **%** | **Worsening** |
| --- | --- | --- | --- |
| **F21. Change in anxiety** | It got a lot worse | 17.58 |  |
|  | It got a little worse | 41.46 |  |
|  | Neither better nor worse | 35.00 |  |
|  | It got a little improved | 4.65 | 59.04 |
|  | It got improved a lot | 1.31 |  |
|  |  |  |  |
| **G21. Change in depressive affect** | It got a lot worse | 15.87 |  |
|  | It got a little worse | 39.62 |  |
|  | Neither better nor worse | 38.27 |  |
|  | It got a little improved | 4.99 | 55.49 |
|  | It got improved a lot | 1.23 |  |
|  |  |  |  |
| **O11. Change in suicidal thoughts** | Very much increased | 4.00 |  |
|  | Increased a bit | 2.35 |  |
|  | Neither increased. nor decreased | 75.24 |  |
|  | Decreased a bit | 14.18 | 6.34 |
|  | Very much decreased | 4.22 |  |

**WebTable 7:** Change in anxiety, depressive feelings and suicidality in the whole sample

|  | | **Females** | | | **Males** | | | **Non-binary gender** | | | **All Grps** | **Normal expected** |
| --- | --- | --- | --- | --- | --- | --- | --- | --- | --- | --- | --- | --- |
| **Type of studies** |  | **A** | **B** | **C** | **A** | **B** | **C** | **A** | **B** | **C** |  |  |
| **Change in Anxiety** | **mean** | -0.68 | -0.81 | -0.79 | -0.48 | -0.56 | -0.47 | -0.84 | -0.76 | -0.93 | -0.69 |  |
|  | **SD** | 0.85 | 0.86 | 0.84 | 0.85 | 0.85 | 0.82 | 1.00 | 0.90 | 0.90 | 0.86 |  |
| **Chance in depressive feelings** | **mean** | -0.60 | -0.73 | -0.72 | -0.51 | -0.53 | -0.49 | -0.84 | -0.87 | -0.86 | -0.64 |  |
|  | **SD** | 0.87 | 0.84 | 0.85 | 0.82 | 0.84 | 0.80 | 0.84 | 0.92 | 0.73 | 0.85 |  |
| **Chance in suicidal thoughts** | **mean** | 0.09 | 0.17 | 0.13 | 0.10 | 0.11 | 0.12 | 0.28 | 0.47 | 0.14 | 0.12 |  |
|  | SD | 0.67 | 0.68 | 0.73 | 0.70 | 0.69 | 0.67 | 0.70 | 0.73 | 0.77 | 0.69 |  |
| **CES-D** | mean | 21.93 | 22.81 | 22.57 | 19.48 | 18.86 | 19.22 | 26.16 | 25.98 | 24.86 | 21.56 | 10.78 |
|  | SD | 9.97 | 10.04 | 9.94 | 9.30 | 9.04 | 9.80 | 9.71 | 9.62 | 9.42 | 9.92 | 9.68 |
| **STAI-S** | mean | 46.31 | 46.55 | 46.73 | 46.05 | 45.48 | 44.90 | 46.02 | 46.80 | 48.11 | 46.26 | 24.95 |
|  | SD | 6.41 | 6.10 | 6.28 | 6.71 | 6.54 | 6.88 | 6.39 | 5.76 | 5.90 | 6.40 | 11.36 |
| **RASS-Int** | mean | 81.25 | 81.48 | 82.57 | 84.83 | 73.00 | 69.09 | 146.98 | 170.67 | 143.75 | 80.79 | 32.79 |
|  | SD | 132.60 | 133.73 | 135.34 | 138.18 | 125.57 | 125.09 | 135.58 | 169.42 | 158.54 | 133.09 | 89.73 |
| **RASS-Llfe** | mean | 211.40 | 222.52 | 214.89 | 203.11 | 208.75 | 200.19 | 260.93 | 290.56 | 278.75 | 213.58 | 103.29 |
|  | SD | 145.75 | 146.38 | 146.43 | 142.73 | 141.15 | 134.36 | 162.65 | 154.88 | 123.18 | 144.79 | 94.93 |
| **RASS-His** | mean | 72.29 | 66.72 | 75.56 | 60.07 | 53.41 | 60.45 | 120.23 | 102.11 | 93.86 | 68.26 | 36.08 |
|  | SD | 71.13 | 69.96 | 73.28 | 66.46 | 65.02 | 66.77 | 90.65 | 81.37 | 80.37 | 70.73 | 48.74 |
| **RASS-total** | mean | 364.94 | 370.72 | 373.02 | 348.01 | 335.15 | 329.72 | 528.14 | 563.33 | 516.36 | 362.63 | 172.17 |
|  | SD | 287.17 | 287.23 | 291.67 | 291.70 | 272.81 | 260.79 | 329.89 | 327.45 | 282.79 | 286.36 | 180.26 |

**WebTable 8:** Change in anxiety, depressive feelings and suicidality when scores are utilized in a -2 to +2 scale, along with scores in the other scales used, in the stratified sample. ANOVA with sex and type of studies as grouping and age, perceived changes in anxiety, depression and suicidality in comparison to the pre-lockdown period, scores in STAI-S, CES-D, and RASS subscales (Intention, Life and History) returned significant results for sex (Wilks =0.955, F=31.90, effect df=18, error df=249E2, p<0.001) and the interaction of sex by type (Wilks=0.994, F=2.16, effect df=36 error df=467E2, p<0.001) but not type alone (Wilks =0.998, F=1.28, effect df=18, error df=249E2, p=0.187). Scheffe post hoc test revealed a complex picture, but without a clear trend.

| **Country** | **Sex** | **Neither dysphoria nor depression** | | **Dysphoria** | | **Depression** | | **Either dysphoria nor depression** | | **Total N** | **Female-to-male ratio** |
| --- | --- | --- | --- | --- | --- | --- | --- | --- | --- | --- | --- |
|  |  | N | % | N | % | N | % | N | % |  |  |
| **Bulgaria** | Females | 105 | 64.81 | 21 | 12.96 | 36 | 22.22 | 57 | 35.19 | 162 | 3.24 |
|  | Males | 85 | 83.33 | 10 | 9.80 | 7 | 6.86 | 17 | 16.67 | 102 |  |
|  | Non-binary gender | 2 | 100.00 | 0 | 0.00 | 0 | 0.00 | 0 | 0.00 | 2 |  |
|  | Total | 192 | 72.18 | 31 | 11.65 | 43 | 16.17 | 74 | 27.82 | 266 |  |
| **Croatia** | Females | 637 | 50.80 | 195 | 15.55 | 422 | 33.65 | 617 | 49.20 | 1254 | 1.32 |
|  | Males | 179 | 60.27 | 42 | 14.14 | 76 | 25.59 | 118 | 39.73 | 297 |  |
|  | Non-binary gender | 3 | 33.33 | 1 | 11.11 | 5 | 55.56 | 6 | 66.67 | 9 |  |
|  | Total | 819 | 52.50 | 238 | 15.26 | 503 | 32.24 | 741 | 47.50 | 1560 |  |
| **Georgia** | Females | 362 | 51.86 | 115 | 16.48 | 221 | 31.66 | 336 | 48.14 | 698 | 2.46 |
|  | Males | 203 | 72.50 | 41 | 14.64 | 36 | 12.86 | 77 | 27.50 | 280 |  |
|  | Non-binary gender | 4 | 66.67 | 0 | 0.00 | 2 | 33.33 | 2 | 33.33 | 6 |  |
|  | Total | 569 | 57.83 | 156 | 15.85 | 259 | 26.32 | 415 | 42.17 | 984 |  |
| **Greece** | Females | 2269 | 57.68 | 600 | 15.25 | 1065 | 27.07 | 1665 | 42.32 | 3934 | 1.95 |
|  | Males | 1054 | 72.54 | 197 | 13.56 | 202 | 13.90 | 399 | 27.46 | 1453 |  |
|  | Non-binary gender | 25 | 36.23 | 14 | 20.29 | 30 | 43.48 | 44 | 63.77 | 69 |  |
|  | Total | 3348 | 61.36 | 811 | 14.86 | 1297 | 23.77 | 2108 | 38.64 | 5456 |  |
| **India** | Females | 149 | 48.53 | 55 | 17.92 | 103 | 33.55 | 158 | 51.47 | 307 | 1.19 |
|  | Males | 119 | 55.87 | 34 | 15.96 | 60 | 28.17 | 94 | 44.13 | 213 |  |
|  | Non-binary gender | 0 | 0.00 | 0 | 0.00 | 1 | 100.00 | 1 | 100.00 | 1 |  |
|  | Total | 268 | 51.44 | 89 | 17.08 | 164 | 31.48 | 253 | 48.56 | 521 |  |
| **Latvia** | Females | 372 | 44.93 | 157 | 18.96 | 299 | 36.11 | 456 | 55.07 | 828 | 1.50 |
|  | Males | 133 | 62.74 | 28 | 13.21 | 51 | 24.06 | 79 | 37.26 | 212 |  |
|  | Non-binary gender | 0 | 0.00 | 2 | 28.57 | 5 | 71.43 | 7 | 100.00 | 7 |  |
|  | Total | 505 | 48.23 | 187 | 17.86 | 355 | 33.91 | 542 | 51.77 | 1047 |  |
| **Lithuania** | Females | 92 | 33.21 | 56 | 20.22 | 129 | 46.57 | 185 | 66.79 | 277 | 1.60 |
|  | Males | 27 | 49.09 | 12 | 21.82 | 16 | 29.09 | 28 | 50.91 | 55 |  |
|  | Non-binary gender | 0 | 0.00 | 1 | 33.33 | 2 | 66.67 | 3 | 100.00 | 3 |  |
|  | Total | 119 | 35.52 | 69 | 20.60 | 147 | 43.88 | 216 | 64.48 | 335 |  |
| **Nigeria** | Females | 239 | 91.22 | 10 | 3.82 | 13 | 4.96 | 23 | 8.78 | 262 | 1.07 |
|  | Males | 285 | 87.96 | 24 | 7.41 | 15 | 4.63 | 39 | 12.04 | 324 |  |
|  | Non-binary gender | 0 | 0.00 | 0 | 0.00 | 1 | 100.00 | 1 | 100.00 | 1 |  |
|  | Total | 524 | 89.27 | 34 | 5.79 | 29 | 4.94 | 63 | 10.73 | 587 |  |
| **Russia** | Females | 596 | 56.60 | 196 | 18.61 | 261 | 24.79 | 457 | 43.40 | 1053 | 1.32 |
|  | Males | 198 | 58.24 | 78 | 22.94 | 64 | 18.82 | 142 | 41.76 | 340 |  |
|  | Non-binary gender | 16 | 50.00 | 8 | 25.00 | 8 | 25.00 | 16 | 50.00 | 32 |  |
|  | Total | 810 | 56.84 | 282 | 19.79 | 333 | 23.37 | 615 | 43.16 | 1425 |  |
| **Malaysia** | Females | 72 | 40.22 | 41 | 22.91 | 66 | 36.87 | 107 | 59.78 | 179 | 1.95 |
|  | Males | 22 | 59.46 | 8 | 21.62 | 7 | 18.92 | 15 | 40.54 | 37 |  |
|  | Non-binary gender | 1 | 50.00 | 1 | 50.00 | 0 | 0.00 | 1 | 50.00 | 2 |  |
|  | Total | 95 | 43.58 | 50 | 22.94 | 73 | 33.49 | 123 | 56.42 | 218 |  |
| **Hungary** | Females | 45 | 63.38 | 7 | 9.86 | 19 | 26.76 | 26 | 36.62 | 71 | 2.14 |
|  | Males | 12 | 75.00 | 2 | 12.50 | 2 | 12.50 | 4 | 25.00 | 16 |  |
|  | Non-binary gender | 0 |  | 0 |  | 0 |  | 0 |  | 0 |  |
|  | Total | 57 | 65.52 | 9 | 10.34 | 21 | 24.14 | 30 | 34.48 | 87 |  |
| **Total** | Females | 4938 | 54.71 | 1453 | 16.10 | 2634 | 29.19 | 4087 | 45.29 | 9025 | 1.81 |
|  | Males | 2317 | 69.60 | 476 | 14.30 | 536 | 16.10 | 1012 | 30.40 | 3329 |  |
|  | Non-binary gender | 51 | 38.64 | 27 | 20.45 | 54 | 40.91 | 81 | 61.36 | 132 |  |
|  | Total | 7306 | 58.51 | 1956 | 15.67 | 3224 | 25.82 | 5180 | 41.49 | 12486 |  |

**Webtable 9:** Rates of depression and dysphoria by country and sex

|  |  | Depression | | | Row  N |  |
| --- | --- | --- | --- | --- | --- | --- |
|  |  | **No** | **Yes** | **%** |  | **Female-to-male ratio** |
| A | Females | 2406 | 856 | 26.24 | 3262 | 1.64 |
|  | Males | 746 | 142 | ***15.99*** | 888 |  |
|  | Non-binary gender | 26 | 17 | 39.53 | 43 |  |
|  | All | 3178 | 1015 | 24.21 | 4193 |  |
| B | Females | 1748 | 791 | ***31.15*** | 2539 | 1.92 |
|  | Males | 1350 | 262 | 16.25 | 1612 |  |
|  | Non-binary gender | 24 | 21 | 46.67 | 45 |  |
|  | All | 3122 | 1074 | 25.60 | 4196 |  |
| C | Females | 2237 | 987 | 30.61 | 3224 | 1.92 |
|  | Males | 697 | 132 | ***15.92*** | 829 |  |
|  | Non-binary gender | 28 | 16 | 36.36 | 44 |  |
|  | All | 2962 | 1135 | 27.70 | 4097 |  |
|  | | | | | | |
|  | Females | 6391 | 2634 | 29.19 | 9025 | 1.81 |
|  | Males | 2793 | 536 | 16.10 | 3329 |  |
|  | Non-binary gender | 78 | 54 | 40.91 | 132 |  |

**Webtable 10:** rates of depression by sex and type of studies

Rates of depression were higher for non-binary gender in studies B (46.67%) and lowest for males in A and C (15.99% and 15.92%).

Chi-square test revealed an effect of type of studies by gender. There was a difference among females in the three types (chi-square=21.623, df=2, p<0.001), which was due to A vs. B (chi-square=16.947, df=1, p<0.001), A vs. C (chi-square=15.241, df=1, p<0.001), but not B vs. C (chi-square=0.194, df=1, p=0.659). Concerning males, there was no difference among males in the three types (chi-square=0.055, df=2, p=0.972). Similarly, there were no differences concerning ‘non-binary gender’ in the three types of studies (chi-square=1.027, df=2, p=0.598).

These results suggest a lower depression rate in females in the A type of studies.

There was a difference among sexes (chi-square=233.240, df=2, p<0.001), with males having lower rates in comparison both to females (chi-square=218.274, df=1, p<0.001), and ‘non-binary gender’ (chi-square=55.258, df=1, p<0.001), and ‘non-binary gender’ having higher rates both to females (chi-square=8.622, df=1, p=0.003) and males.

| **Study type** | **Study** | **%** |
| --- | --- | --- |
| **A** | Anthropology * | 40.00 |
|  | Veterinary medicine | 32.37 |
|  | Psychology | 28.70 |
|  | Biology | 26.54 |
|  | Dentistry | 24.24 |
|  | Medicine | 23.40 |
|  | Pharmaceutical sciences | 20.91 |
|  | Athletics. Sports science and Physical Education | 19.23 |
|  | Physiotherapy * | 18.18 |
|  | Nutrition and Dietetics * | 17.65 |
|  | Nursing and Midwifery | 16.51 |
|  | Occupational therapy * | 0.00 |
|  | Speech and language therapy * | 0.00 |
| **B** | Transportation studies * | 42.86 |
|  | Forestry and Natural Environment studies | 35.92 |
|  | Business Administration (Accounting. Management. Finance. Marketing) | 33.75 |
|  | Earth sciences / Geology / Soil Sciences | 33.33 |
|  | Economics | 30.87 |
|  | Architecture. Design and Planning | 28.79 |
|  | Aeronautics and Aviation science | 25.81 |
|  | Space sciences and cosmic research * | 25.00 |
|  | Agriculture. Geoponic and Fishery Science | 24.49 |
|  | Chemistry | 24.10 |
|  | Physical and Chemical engineering | 23.08 |
|  | Mechanical engineering and Materials science | 22.00 |
|  | Computer Science / Informatics | 21.99 |
|  | Military training * | 21.43 |
|  | Mathematics and Statistics | 21.40 |
|  | Geography | 21.21 |
|  | Physics | 20.63 |
|  | Automotive Engineering * | 20.00 |
|  | Civil Engineering | 20.00 |
|  | Information Systems. Library and Museum studies | 17.78 |
|  | Electrical and Electronic engineering | 16.56 |
|  | Maritime studies * | 0.00 |
| **C** | Philosophy | 33.33 |
|  | Fine arts (visual arts. performing arts. culinary art) | 33.23 |
|  | Languages. Literature & Linguistics | 31.47 |
|  | Other | 30.20 |
|  | History and Archaeology | 29.89 |
|  | Religion studies / Theology | 28.74 |
|  | Foreign languages. Translation and Interpretation | 25.31 |
|  | Law | 25.11 |
|  | Political Science and International Relations | 24.84 |
|  | Journalism. mass media and communication | 23.67 |
|  | Teaching and Education | 21.74 |
|  | Sociology / Contemporary Social sciences | 20.45 |
|  | Social work | 16.00 |
|  | Public relations and Administration | 15.91 |
| **All Grps** |  | 25.82 |

**Webtable 11:** Rates of clinical depression by specific study school, ranked by type of studies and in descending order

* very low numbers

|  |  |  | **No clinical depression** | | **Clinical**  **depression** | | **Row** | **RATE**  **Mental history**  **vs.**  **no mental history** |
| --- | --- | --- | --- | --- | --- | --- | --- | --- |
| **Sex** | **Type of studies** | **History of mental health** | **N** | **%** | **N** | **%** | **N** |  |
| **Females** | **A** | **No history of any mental dis** | 1896 | 77.48 | 551 | 22.52 | 2447 | 1.97 |
|  |  | **History of any mental dis** | 510 | 62.58 | 305 | 37.42 | 815 |  |
|  |  | **Depression** | 186 | 52.99 | 165 | 47.01 | 351 |  |
|  |  | **Anxiety** | 206 | 70.55 | 86 | 29.45 | 292 |  |
|  |  | **Bipolar Disorder** | 12 | 44.44 | 15 | 55.56 | 27 |  |
|  |  | **Psychosis** | 12 | 48.00 | 13 | 52.00 | 25 |  |
|  |  | **Other** | 94 | 78.33 | 26 | 21.67 | 120 |  |
|  |  | **total** | 2406 | 73.76 | 856 | 26.24 | 3262 |  |
|  | **B** | **No history of any mental dis** | 1379 | 75.52 | 447 | 24.48 | 1826 | 1.66 |
|  |  | **History of any mental dis** | 369 | 51.75 | 344 | 48.25 | 713 |  |
|  |  | **Depression** | 124 | 42.32 | 169 | 57.68 | 293 |  |
|  |  | **Anxiety** | 172 | 59.93 | 115 | 40.07 | 287 |  |
|  |  | **Bipolar Disorder** | 11 | 36.67 | 19 | 63.33 | 30 |  |
|  |  | **Psychosis** | 11 | 39.29 | 17 | 60.71 | 28 |  |
|  |  | **Other** | 51 | 68.00 | 24 | 32.00 | 75 |  |
|  |  | **total** | 1748 | 68.85 | 791 | 31.15 | 2539 |  |
|  | **C** | **No history of any mental dis** | 1670 | 76.50 | 513 | 23.50 | 2183 | 1.94 |
|  |  | **History of any mental dis** | 567 | 54.47 | 474 | 45.53 | 1041 |  |
|  |  | **Depression** | 184 | 45.43 | 221 | 54.57 | 405 |  |
|  |  | **Anxiety** | 269 | 62.85 | 159 | 37.15 | 428 |  |
|  |  | **Bipolar Disorder** | 9 | 27.27 | 24 | 72.73 | 33 |  |
|  |  | **Psychosis** | 12 | 38.71 | 19 | 61.29 | 31 |  |
|  |  | **Other** | 93 | 64.58 | 51 | 35.42 | 144 |  |
|  |  | **total** | 2237 | 69.39 | 987 | 30.61 | 3224 |  |
|  | **Total** | **No history of any mental dis** | 4945 | 76.60 | 1511 | 23.40 | 6456 | 1.87 |
|  |  | **History of any mental dis** | 1446 | 56.29 | 1123 | 43.71 | 2569 |  |
| **Males** | **A** | **No history of any mental dis** | 628 | 87.47 | 90 | 12.53 | 718 | 2.43 |
|  |  | **History of any mental dis** | 118 | 69.41 | 52 | 30.59 | 170 |  |
|  |  | **Depression** | 49 | 62.03 | 30 | 37.97 | 79 |  |
|  |  | **Anxiety** | 38 | 79.17 | 10 | 20.83 | 48 |  |
|  |  | **Bipolar Disorder** | 4 | 44.44 | 5 | 55.56 | 9 |  |
|  |  | **Psychosis** | 9 | 75.00 | 3 | 25.00 | 12 |  |
|  |  | **Other** | 18 | 81.82 | 4 | 18.18 | 22 |  |
|  |  | **total** | 746 | 84.01 | 142 | 15.99 | 888 |  |
|  | **B** | **No history of any mental dis** | 1133 | 87.29 | 165 | 12.71 | 1298 | 2.44 |
|  |  | **History of any mental dis** | 217 | 69.11 | 97 | 30.89 | 314 |  |
|  |  | **Depression** | 88 | 61.11 | 56 | 38.89 | 144 |  |
|  |  | **Anxiety** | 83 | 83.84 | 16 | 16.16 | 99 |  |
|  |  | **Bipolar Disorder** | 12 | 66.67 | 6 | 33.33 | 18 |  |
|  |  | **Psychosis** | 7 | 43.75 | 9 | 56.25 | 16 |  |
|  |  | **Other** | 27 | 72.97 | 10 | 27.03 | 37 |  |
|  |  | **total** | 1350 | 83.75 | 262 | 16.25 | 1612 |  |
|  | **C** | **No history of any mental dis** | 595 | 87.37 | 86 | 12.63 | 681 | 2.46 |
|  |  | **History of any mental dis** | 102 | 68.92 | 46 | 31.08 | 148 |  |
|  |  | **Depression** | 36 | 54.55 | 30 | 45.45 | 66 |  |
|  |  | **Anxiety** | 35 | 85.37 | 6 | 14.63 | 41 |  |
|  |  | **Bipolar Disorder** | 5 | 55.56 | 4 | 44.44 | 9 |  |
|  |  | **Psychosis** | 5 | 55.56 | 4 | 44.44 | 9 |  |
|  |  | **Other** | 21 | 91.30 | 2 | 8.70 | 23 |  |
|  |  | **total** | 697 | 84.08 | 132 | 15.92 | 829 |  |
|  | **Total** | **No history of any mental dis** | 2356 | 87.36 | 341 | 12.64 | 2697 | 2.44 |
|  |  | **History of any mental dis** | 437 | 69.15 | 195 | 30.85 | 632 |  |
| **Other** | **A** | **No history of any mental dis** | 18 | 78.26 | 5 | 21.74 | 23 | 2.22 |
|  |  | **History of any mental dis** | 8 | 40.00 | 12 | 60.00 | 20 |  |
|  |  | **Depression** | 3 | 37.50 | 5 | 62.50 | 8 |  |
|  |  | **Anxiety** | 3 | 50.00 | 3 | 50.00 | 6 |  |
|  |  | **Bipolar Disorder** | 0 |  | 0 |  | 0 |  |
|  |  | **Psychosis** | 1 | 33.33 | 2 | 66.67 | 3 |  |
|  |  | **Other** | 1 | 33.33 | 2 | 66.67 | 3 |  |
|  |  | **total** | 26 | 60.47 | 17 | 39.53 | 43 |  |
|  | **B** | **No history of any mental dis** | 18 | 69.23 | 8 | 30.77 | 26 | 2.76 |
|  |  | **History of any mental dis** | 6 | 31.58 | 13 | 68.42 | 19 |  |
|  |  | **Depression** | 2 | 28.57 | 5 | 71.43 | 7 |  |
|  |  | **Anxiety** | 2 | 50.00 | 2 | 50.00 | 4 |  |
|  |  | **Bipolar Disorder** | 0 | 0.00 | 2 | 100.00 | 2 |  |
|  |  | **Psychosis** | 0 | 0.00 | 1 | 100.00 | 1 |  |
|  |  | **Other** | 2 | 40.00 | 3 | 60.00 | 5 |  |
|  |  | **total** | 24 | 53.33 | 21 | 46.67 | 45 |  |
|  | **C** | **No history of any mental dis** | 20 | 71.43 | 8 | 28.57 | 28 | 1.75 |
|  |  | **History of any mental dis** | 8 | 50.00 | 8 | 50.00 | 16 |  |
|  |  | **Depression** | 5 | 50.00 | 5 | 50.00 | 10 |  |
|  |  | **Anxiety** | 2 | 40.00 | 3 | 60.00 | 5 |  |
|  |  | **Bipolar Disorder** | 0 |  | 0 |  | 0 |  |
|  |  | **Psychosis** | 0 |  | 0 |  | 0 |  |
|  |  | **Other** | 1 | 100.00 | 0 | 0.00 | 1 |  |
|  |  | **total** | 28 | 63.64 | 16 | 36.36 | 44 |  |
|  | **Total** | **No history of any mental dis** | 56 | 72.73 | 21 | 27.27 | 77 | 2.20 |
|  |  | **History of any mental dis** | 22 | 40.00 | 33 | 60.00 | 55 |  |
| **Total** | | **No history of any mental dis** | 7357 | 79.71 | 1873 | 20.29 | 9230 | 2.04 |
|  |  | **History of any mental dis** | 1905 | 58.51 | 1351 | 41.49 | 3256 |  |
| **TOTAL** | | | 9262 | 74.18 | 3224 | 25.82 | 12486 |  |

**Webtable 12:** rates of clinical depression by sex, type of studies and history of mental disorders

ANCOVA with the presence of clinical depression and history of each mental disorder separately and changes in anxiety, depressive feelings and suicidal thoughts as independent variables and sex and age as covariates returned main effect for clinical depression (wilks=0.851; F=725.9; effect df:3; error df:12475; p<0.001), history of anxiety (wilks=0.995 F=19.1; Effect df:3 error df:12475; p<0.001), depression (wilks=0.996 F=17.9; effect df:3; error df:12475; p<0.001) and bipolar disorder (wilks=0.999 F=4.5 effect df:3; error df:12475; p=0.004). All scheffe post hoc tests were significant at p<0.01. All group means pointed toward a negative change in all three independent variables reflecting change in mental health

***3.4 Lifestyle changes during the pandemic.***

There were lifestyle changes concerning everyday activities, internet use, sex and sleep

At the time of interview, 17.11%, were under strict lockdown (D2), 41.90%, to a high degree 24.95% partially and 16.03% under no lockdown at all. Chi-square test in pairs among the four lockdown groups and the presence of clinical depression returned no differences except for the complete lockdown which manifested significantly higher depression rates in comparison to all the other states of lockdown (31.63% 24.62%; RR=1.28; p<0.01). All correlations were significant (p<0.05) but minimal among the degree of lockdown and changes in anxiety (R=-0.13), changes in depressive feelings (R=-0.14), changes in suicidal thoughts (R=0.07), STAI (R=0.02), CES-D (0.06), RASS-Intention (R=0.03), RASS life (R=0.02).

More than 50% of the total sample reported that the time spent outside the house was less than humanly necessary or worse, without any differences among types of studies.

More than 90% were suggesting that they were following the precaution measures suggested by the WHO at least moderately, with 2/3 saying that they were much or very much following them. One third was feeling that information was not adequate.

Family dynamics changed towards increased emotional bonding and on average conflicts did not change. Only close to 20% did not manage to keep a basic daily routine and almost half were expecting their financial status to worsen.

More than 90% considered exercise to be of high importance during the pandemic but more persons experienced a decrease in physical activity. Eating increased in 40% and approximately 20% was eating in an unhealthier way. More than 30% has put weight.

Half of the study sample increased the time spent in the internet and almost 2/3 increased the use of the social media. Close to 25% acquired new internet habits.

Sleep worsened in approximately 45% with more than 50% going to sleep rather late and 20% having nightmares.

Smoking increased in 25%, alcohol use in close to 40% and illegal substance use in 25%.

Sexual life was poor in approximately 45% with a decrease in desire in approximately 20%

These findings were comparable across types of studies (WebTable 13).

| **D1. According to your opinion, the amount of time that you spend outside of your house for reasons not regarding your work during this period is:** | **Minimum** | **Less than humanly necessary** | **Moderate/**  **reasonable** | **Enough** | **A lot** | **Excessive** |
| --- | --- | --- | --- | --- | --- | --- |
| **A** | 37.90 | 23.42 | 25.42 | 9.09 | 3.22 | 0.95 |
| **B** | 37.03 | 27.09 | 22.61 | 10.03 | 2.36 | 0.88 |
| **C** | 37.53 | 26.06 | 23.50 | 9.47 | 2.64 | 0.81 |
| **Total** | 37.48 | 25.52 | 23.85 | 9.53 | 2.74 | 0.88 |
|  |  |  |  |  |  |  |
| **D2. Are you currently locked up in the house?** | **Completely** | **To a high degree** | **Partially** | **Not at all** |  |  |
| **A** | 20.10 | 26.19 | 39.40 | 14.31 |  |  |
| **B** | 14.11 | 25.54 | 42.41 | 17.94 |  |  |
| **C** | 13.84 | 23.08 | 43.95 | 19.13 |  |  |
| **Total** | 16.03 | 24.95 | 41.90 | 17.11 |  |  |
|  |  |  |  |  |  |  |
| **D3. According to the instructions given by WHO, it is necessary for some precautions to be taken in order to prevent the spread of the virus. Do you believe that you take adequate precautions?** | **Not at all** | **A little bit** | **Moderately** | **Much** | **Very Much** |  |
| **A** | 1.31 | 5.94 | 25.07 | 48.39 | 19.29 |  |
| **B** | 1.95 | 5.05 | 25.95 | 46.22 | 20.82 |  |
| **C** | 1.39 | 4.15 | 25.84 | 45.95 | 22.67 |  |
| **Total** | 1.55 | 5.05 | 25.62 | 46.86 | 20.92 |  |
|  |  |  |  |  |  |  |
| **D4. Do you believe that that you have received sufficient information about the necessity of the measures taken?** | **Not at all** | **A little bit** | **Moderately** | **Much** | **Very Much** |  |
| **A** | 2.12 | 4.98 | 20.63 | 45.12 | 27.14 |  |
| **B** | 4.29 | 7.17 | 25.61 | 41.24 | 21.68 |  |
| **C** | 3.03 | 5.95 | 26.84 | 41.00 | 23.18 |  |
| **Total** | 3.15 | 6.04 | 24.34 | 42.46 | 24.01 |  |
|  |  |  |  |  |  |  |
| **E1. Do you feel the need to communicate with other members of your family during this period?** | **Much less** | **Less** | **Same** | **More** | **Much more** |  |
| **A** | 3.82 | 6.58 | 54.57 | 24.66 | 10.37 |  |
| **B** | 4.17 | 7.53 | 55.73 | 24.11 | 8.46 |  |
| **C** | 4.17 | 7.30 | 54.88 | 24.33 | 9.32 |  |
| **Total** | 4.05 | 7.13 | 55.06 | 24.37 | 9.39 |  |
|  |  |  |  |  |  |  |
| **E2. Do you want to receive emotional support from other members of your family during this period?** | **Much less** | **Less** | **Same** | **More** | **Much more** |  |
| **A** | 3.84 | 5.77 | 57.45 | 23.71 | 9.23 |  |
| **B** | 5.08 | 6.31 | 57.90 | 22.71 | 8.01 |  |
| **C** | 4.95 | 5.64 | 54.54 | 25.11 | 9.76 |  |
| **Total** | 4.62 | 5.91 | 56.65 | 23.83 | 8.99 |  |
|  |  |  |  |  |  |  |
| **E3. Are there any conflicts with the rest of your family members during this period?** | **Much less** | **Less** | **Same** | **More** | **Much more** |  |
| **A** | 10.64 | 13.21 | 54.26 | 17.08 | 4.82 |  |
| **B** | 7.10 | 10.75 | 55.44 | 20.49 | 6.22 |  |
| **C** | 7.78 | 11.20 | 53.93 | 20.23 | 6.86 |  |
| **Total** | 8.51 | 11.72 | 54.55 | 19.26 | 5.96 |  |
|  |  |  |  |  |  |  |
| **E4. Has the overall quality of relationships with the other members of your family changed compared to before the COVID – 19?** | **Much worse** | **Worse** | **It has not changed** | **A little bit better** | **Much better** |  |
| **A** | 1.91 | 11.83 | 66.40 | 15.67 | 4.20 |  |
| **B** | 1.86 | 13.51 | 64.57 | 16.51 | 3.55 |  |
| **C** | 1.66 | 13.10 | 65.42 | 15.35 | 4.47 |  |
| **Total** | 1.81 | 12.81 | 65.46 | 15.85 | 4.07 |  |
|  |  |  |  |  |  |  |
| **E5. Do you manage to maintain a basic daily routine (waking up in the morning, regular meals and sleeping hours, activities) both yourself (if you live alone) or as a family?** | **Not at all** | **Somehow, but not always** | **Generally, yes** | **Clearly follow (or adhere to) a routine** |  |  |
| **A** | 18.10 | 35.46 | 37.82 | 8.61 |  |  |
| **B** | 19.85 | 35.72 | 36.53 | 7.91 |  |  |
| **C** | 18.47 | 34.16 | 37.75 | 9.61 |  |  |
| **Total** | 18.81 | 35.12 | 37.36 | 8.70 |  |  |
|  |  |  |  |  |  |  |
| **E6. If you have children, how difficult is it to manage their daily life and behavior?** | **I do not have children** | **Much more difficult than before** | **Somehow more difficult but not always** | **Same as always** | **Somehow easier but not always** | **Much easier than before** |
| **A** | 0.68 | 1.28 | 97.11 | 0.70 | 0.23 | 0.00 |
| **B** | 0.77 | 1.28 | 97.31 | 0.51 | 0.14 | 0.00 |
| **C** | 0.92 | 1.59 | 96.93 | 0.42 | 0.15 | 0.00 |
| **Total** | 0.79 | 1.38 | 97.11 | 0.54 | 0.17 | 0.00 |
|  |  |  |  |  |  |  |
| **E7. How are your finances as a result of the outbreak?** | **Much more difficult than before** | **Somehow more difficult** | **Same as**  **always** | **Somehow**  **easier** | **Much easier than before** |  |
| **A** | 14.40 | 29.93 | 45.55 | 8.06 | 2.05 |  |
| **B** | 15.56 | 29.81 | 44.48 | 8.27 | 1.88 |  |
| **C** | 16.30 | 31.19 | 43.46 | 7.35 | 1.71 |  |
| **Total** | 15.41 | 30.30 | 44.51 | 7.90 | 1.88 |  |
|  |  |  |  |  |  |  |
| **H1. Does exercise help you at the prevention of anxiety?** | **Not at**  **all** | **A little**  **bit** | **Moderately** | **Much** | **Very**  **much** |  |
| **A** | 13.81 | 21.63 | 24.68 | 24.35 | 15.53 |  |
| **B** | 12.63 | 20.35 | 28.50 | 24.48 | 14.04 |  |
| **C** | 13.72 | 21.55 | 29.24 | 22.70 | 12.79 |  |
| **All Grps** | 13.38 | 21.18 | 27.46 | 23.85 | 14.13 |  |
|  |  |  |  |  |  |  |
| **H2. Do you consider that exercise is important during this epidemic?** |  |  |  |  |  |  |
| **A** | 2.96 | 5.96 | 18.32 | 36.56 | 36.20 |  |
| **B** | 2.53 | 6.27 | 19.90 | 36.82 | 34.49 |  |
| **C** | 2.61 | 6.13 | 23.02 | 36.27 | 31.97 |  |
| **All Grps** | 2.70 | 6.12 | 20.39 | 36.55 | 34.24 |  |
|  |  |  |  |  |  |  |
| **H3. Do you have increased the frequency and intensity of your physical workout during this epidemic and lockdown?** |  |  |  |  |  |  |
| **A** | 38.28 | 18.27 | 23.80 | 12.45 | 7.20 |  |
| **B** | 35.56 | 17.78 | 27.38 | 12.23 | 7.05 |  |
| **C** | 35.61 | 18.55 | 26.60 | 12.35 | 6.88 |  |
| **All Grps** | 36.49 | 18.20 | 25.93 | 12.34 | 7.05 |  |
|  |  |  |  |  |  |  |
| **H4. How much has your physical activity been affected by this epidemic of COVID-19?** | **It**  **decreased**  **much** | **It decreased**  **a little** | **Neither**  **Decreased**  **nor**  **increased** | **It increased**  **a little** | **It increased**  **much** |  |
| **A** | 22.97 | 19.46 | 24.61 | 21.35 | 11.61 |  |
| **B** | 27.10 | 16.30 | 28.05 | 20.59 | 7.96 |  |
| **C** | 24.21 | 15.13 | 30.39 | 21.11 | 9.15 |  |
| **All Grps** | 24.76 | 16.98 | 27.66 | 21.02 | 9.58 |  |
|  |  |  |  |  |  |  |
| **I1. Did you notice the need to eat larger amounts of food or eat more often?** | **I eat much**  **less than I**  **used to** | **I eat bit**  **less than**  **I used to** | **Neither**  **more nor**  **less** | **I eat a**  **bit more**  **than I**  **used to** | **I eat much**  **more than**  **I used to** |  |
| **A** | 7.51 | 14.17 | 37.37 | 30.46 | 10.49 |  |
| **B** | 6.67 | 15.87 | 38.61 | 29.72 | 9.13 |  |
| **C** | 7.10 | 13.20 | 37.95 | 31.63 | 10.10 |  |
| **All Grps** | 7.10 | 14.42 | 37.98 | 30.59 | 9.91 |  |
|  |  |  |  |  |  |  |
| **I2. Please mark the answer that best represents you during this period:** | **I eat in a**  **healthier**  **way.** | **My eating**  **habits and**  **preferences**  **have not**  **changed** | **I eat in a**  **more**  **unhealthy**  **way** |  |  |  |
| **A** | 23.13 | 57.07 | 19.79 |  |  |  |
| **B** | 22.31 | 58.32 | 19.38 |  |  |  |
| **C** | 22.80 | 57.60 | 19.60 |  |  |  |
| **All Grps** | 22.75 | 57.66 | 19.59 |  |  |  |
|  |  |  |  |  |  |  |
| **I3. Please mark the answer that best represents you during this period (you can choose more than one answers):** | **My body**  **weight has**  **significantly**  **decreased**  **(more than**  **2-3 kilos).** | **My body**  **weight has**  **slightly**  **decreased**  **(less than 2**  **kilos lost).** | **My body**  **weight is**  **stable** | **My body**  **weight**  **has**  **slightly**  **increased**  **(less than**  **2 kilos**  **put).** | **My body**  **weight has**  **significantly**  **increased**  **(more than**  **2-3 kilos).** |  |
| **A** | 11.10 | 13.68 | 40.57 | 22.18 | 12.46 |  |
| **B** | 9.95 | 15.27 | 41.06 | 21.88 | 11.84 |  |
| **C** | 9.18 | 15.46 | 41.08 | 22.70 | 11.58 |  |
| **All Grps** | 10.08 | 14.80 | 40.90 | 22.25 | 11.97 |  |
|  |  |  |  |  |  |  |
| **K1. The information and use of the internet worry me about the issue regarding the COVID-19** | **Not at**  **all** | **A little** | **Moderately** | **Much** | **Very**  **much** |  |
| **B** | 31.55 | 20.88 | 27.79 | 13.42 | 6.36 |  |
| **A** | 35.80 | 22.68 | 23.97 | 12.21 | 5.34 |  |
| **C** | 27.22 | 21.77 | 27.83 | 15.65 | 7.54 |  |
| **All Grps** | 31.56 | 21.78 | 26.52 | 13.74 | 6.41 |  |
|  |  |  |  |  |  |  |
| **K2. Generally. most of the internet sources regarding information about COVID-19 are misinforming/misleading:** |  |  |  |  |  |  |
| **B** | 6.65 | 23.86 | 35.03 | 23.05 | 11.42 |  |
| **A** | 10.99 | 29.22 | 30.69 | 20.08 | 9.02 |  |
| **C** | 6.86 | 22.06 | 34.71 | 24.36 | 12.01 |  |
| **All Grps** | 8.18 | 25.07 | 33.47 | 22.48 | 10.80 |  |
|  |  |  |  |  |  |  |
| **K3. Due to the epidemic conditions. the internet takes up more of my time than usual:** |  |  |  |  |  |  |
| **B** | 11.94 | 12.39 | 25.10 | 28.19 | 22.38 |  |
| **A** | 13.07 | 15.72 | 22.35 | 28.95 | 19.91 |  |
| **C** | 11.03 | 12.55 | 22.85 | 28.83 | 24.75 |  |
| **All Grps** | 12.02 | 13.56 | 23.43 | 28.66 | 22.33 |  |
|  |  |  |  |  |  |  |
| **K4. How much do you use the social media while in isolation at home?** | **Less than**  **before** | **The same**  **as before** | **More**  **than**  **before** |  |  |  |
| **B** | 5.65 | 32.79 | 61.56 |  |  |  |
| **A** | 4.20 | 31.53 | 64.27 |  |  |  |
| **C** | 5.44 | 31.80 | 62.75 |  |  |  |
| **All Grps** | 5.09 | 32.04 | 62.86 |  |  |  |
|  |  |  |  |  |  |  |
| **K5. Have you acquired internet-related habits that you did not have before (for example: created a facebook account. engaging in cybersex or gambling)?** | **No** | **Yes** |  |  |  |  |
| **B** | 75.26 | 24.74 |  |  |  |  |
| **A** | 74.86 | 25.14 |  |  |  |  |
| **C** | 75.32 | 24.68 |  |  |  |  |
| **All Grps** | 75.15 | 24.85 |  |  |  |  |
|  |  |  |  |  |  |  |
| **L1. The quality of my sleep has changed recently. It is:** | **Much**  **worse** | **A little**  **bit worse** | **The same**  **(neither**  **worse nor**  **better)** | **A little**  **better** | **Much**  **better** |  |
| **B** | 16.30 | 30.22 | 43.09 | 8.41 | 1.98 |  |
| **A** | 14.17 | 28.93 | 45.70 | 8.42 | 2.79 |  |
| **C** | 16.65 | 28.90 | 43.47 | 8.37 | 2.61 |  |
| **All Grps** | 15.70 | 29.35 | 44.09 | 8.40 | 2.46 |  |
|  |  |  |  |  |  |  |
| **L2. I tend to stay up late and sleep for many hours during the day.** | **Almost**  **never** | **Rarely** | **Some**  **times** | **Often** | **Almost**  **always** |  |
| **B** | 28.27 | 21.64 | 18.54 | 18.11 | 13.44 |  |
| **A** | 25.92 | 20.58 | 23.42 | 18.29 | 11.78 |  |
| **C** | 30.68 | 19.92 | 19.26 | 16.65 | 13.50 |  |
| **All Grps** | 28.27 | 20.72 | 20.41 | 17.69 | 12.90 |  |
|  |  |  |  |  |  |  |
| **L3. I take sleeping pills to help me sleep at night.** | **Almost**  **never** | **Rarely** | **Some**  **times** | **Often** | **Almost**  **always** |  |
| **B** | 88.85 | 4.62 | 4.67 | 1.14 | 0.71 |  |
| **A** | 86.17 | 5.29 | 6.06 | 1.53 | 0.95 |  |
| **C** | 88.31 | 4.76 | 4.64 | 1.24 | 1.05 |  |
| **All Grps** | 87.77 | 4.89 | 5.13 | 1.31 | 0.91 |  |
|  |  |  |  |  |  |  |
| **L4. I am having dreams in which I feel trapped. over the last 3 weeks.** | **Almost**  **never** | **Rarely** | **Some**  **times** | **Often** | **Almost**  **always** |  |
| **B** | 63.11 | 16.49 | 12.51 | 4.98 | 2.91 |  |
| **A** | 64.11 | 15.84 | 12.62 | 5.34 | 2.10 |  |
| **C** | 61.56 | 17.31 | 13.47 | 5.10 | 2.56 |  |
| **All Grps** | 62.93 | 16.54 | 12.86 | 5.14 | 2.52 |  |
|  |  |  |  |  |  |  |
| **M1. Smoking before the epidemic:** | **I didn't**  **smoke** | **I was**  **smoking** |  |  |  |  |
| **B** | 76.72 | 23.28 |  |  |  |  |
| **A** | 80.04 | 19.96 |  |  |  |  |
| **C** | 76.08 | 23.92 |  |  |  |  |
| **All Grps** | 77.62 | 22.38 |  |  |  |  |
|  |  |  |  |  |  |  |
| **M2. Alcohol use before the epidemic** | **I did not**  **drink**  **much** | **I drank**  **a lot (more**  **than one**  **drink or its**  **equivalent**  **every day)** |  |  |  |  |
| **B** | 90.23 | 9.77 |  |  |  |  |
| **A** | 94.80 | 5.20 |  |  |  |  |
| **C** | 91.87 | 8.13 |  |  |  |  |
| **All Grps** | 92.30 | 7.70 |  |  |  |  |
|  |  |  |  |  |  |  |
| **M3. Use of substances before the epidemic (e.g. hashish. drugs):** | **I did**  **Not**  **use it** | **Occasionally**  **and rather**  **rarely** | **Often** |  |  |  |
| **B** | 85.37 | 11.77 | 2.86 |  |  |  |
| **A** | 89.91 | 8.85 | 1.24 |  |  |  |
| **C** | 87.82 | 10.10 | 2.07 |  |  |  |
| **All Grps** | 87.70 | 10.24 | 2.06 |  |  |  |
|  |  |  |  |  |  |  |
| **M4. During this period. how much do you smoke compared to before?** | **More**  **than**  **before** | **Same as**  **before** | **Less**  **than**  **before** |  |  |  |
| **B** | 25.12 | 64.82 | 10.06 |  |  |  |
| **A** | 23.68 | 66.25 | 10.06 |  |  |  |
| **C** | 26.85 | 64.46 | 8.69 |  |  |  |
| **All Grps** | 25.20 | 65.19 | 9.61 |  |  |  |
|  |  |  |  |  |  |  |
| **M5. During this period. how much alcohol do you drink compared to before?** | **More than before** | **Same as**  **before** | **Less than**  **before** |  |  |  |
| **B** | 40.54 | 49.48 | 9.99 |  |  |  |
| **A** | 34.96 | 54.95 | 10.09 |  |  |  |
| **C** | 39.61 | 51.50 | 8.88 |  |  |  |
| **All Grps** | 38.36 | 51.98 | 9.66 |  |  |  |
|  |  |  |  |  |  |  |
| **M6. During this period. how much do you use illegal substances compared to before?** | **More than before** | **Same as**  **before** | **Less than**  **before** |  |  |  |
| **B** | 28.17 | 68.42 | 3.41 |  |  |  |
| **A** | 26.07 | 71.09 | 2.84 |  |  |  |
| **C** | 28.66 | 68.05 | 3.30 |  |  |  |
| **All Grps** | 27.62 | 69.20 | 3.18 |  |  |  |
|  |  |  |  |  |  |  |
| **N1. How would you characterize the frequency of your sexual intercourse?** | **Clearly inadequate** | **Rather**  **inadequate** | **Neutral** | **Rather satisfactory** | **Clearly satisfactory** |  |
| **B** | 38.49 | 11.73 | 30.86 | 11.13 | 7.79 |  |
| **A** | 33.58 | 11.52 | 34.37 | 11.52 | 9.02 |  |
| **C** | 37.66 | 11.84 | 31.78 | 9.69 | 9.03 |  |
| **All Grps** | 36.57 | 11.69 | 32.34 | 10.79 | 8.61 |  |
|  |  |  |  |  |  |  |
| **N2. Has your desire for sexual intercourse increased or decreased due to the epidemic?** | **It has decreased a lot** | **It has**  **decreased**  **slightly** | **It remains**  **the same** | **It has increased slightly** | **It has increased a lot** |  |
| **B** | 8.58 | 10.89 | 49.83 | 21.73 | 8.96 |  |
| **A** | 7.94 | 9.04 | 54.21 | 21.46 | 7.35 |  |
| **C** | 8.93 | 11.06 | 49.40 | 20.87 | 9.74 |  |
| **All Grps** | 8.48 | 10.32 | 51.16 | 21.36 | 8.67 |  |
|  |  |  |  |  |  |  |
| **N3. How much pleasure and satisfaction do you get from your current sex life?** | **Not at all** | **A little bit** | **Moderate** | **Much** | **Very much** |  |
| **B** | 39.04 | 14.20 | 26.72 | 12.77 | 7.27 |  |
| **A** | 36.08 | 12.81 | 29.55 | 13.64 | 7.92 |  |
| **C** | 39.18 | 12.59 | 26.21 | 14.21 | 7.81 |  |
| **All Grps** | 38.09 | 13.21 | 27.50 | 13.54 | 7.66 |  |
|  |  |  |  |  |  |  |
| **N4. Do you think sex helps you deal with your daily stress and anxiety?** | **Not at all** | **A little**  **bit** | **Moderate** | **Much** | **Very much** |  |
| **B** | 15.87 | 11.37 | 30.48 | 25.55 | 16.73 |  |
| **A** | 21.37 | 10.99 | 29.95 | 24.06 | 13.62 |  |
| **C** | 17.16 | 11.47 | 29.73 | 25.95 | 15.69 |  |
| **All Grps** | 18.14 | 11.28 | 30.06 | 25.18 | 15.35 |  |

**WebTable 13:** Lifestyle changes during the pandemic

***3.5 Beliefs in conspiracy theories***

Beliefs in conspiracy theories seem widely prevalent with acceptance rates depending on the nature of the belief. Differences among countries were significant, but there were no overall differences among sexes. Interestingly, the type A studies had surprisingly high acceptance rates of COVID-19 conspiracy theories with close to 25% believing that the vaccines include a chip and almost 40% suggest that facemask wearing could be a method of socio-political control. One third of type persons of A studies were reserved towards vaccines in general, 20% were accepting the chemtrail conspiracy, 20% disputed climate change, 40% were not precluding that mind control devices are used upon the population, 45% were believing that experiments of new drugs and technologies are systematically performed on the population secretly and 8% were accepting the flat earth theory. The rate of the flat earth theory adds validity to our study sample as it is comparable, although lower to that reported by other studies

Rates of believing were lower in persons without dysphoria or depression, intermediate in those with dysphoria and higher in those with current clinical depression

| **Country** | **Type**  **Of**  **Studies** | **Sex** | **I don’t believe it at all** | **Maybe** | **A little bit** | **Much** | **Very much** |
| --- | --- | --- | --- | --- | --- | --- | --- |
| **J1. Do you believe that the COVID-19 vaccine was ready even before the virus broke out and they conceal it from us for the benefit of pharmaceutical companies?** | | | | | | | |
| **Bulgaria** | A | Females | 48.94 | 17.02 | 29.79 | 4.26 | 0.00 |
|  |  | Males | 71.11 | 11.11 | 13.33 | 0.00 | 4.44 |
|  |  | Non-binary gender | 100.00 | 0.00 | 0.00 | 0.00 | 0.00 |
|  |  | Total | 60.64 | 13.83 | 21.28 | 2.13 | 2.13 |
|  | B | Females | 26.79 | 16.07 | 32.14 | 7.14 | 17.86 |
|  |  | Males | 43.33 | 6.67 | 30.00 | 6.67 | 13.33 |
|  |  | Non-binary gender |  |  |  |  |  |
|  |  | Total | 32.56 | 12.79 | 31.40 | 6.98 | 16.28 |
|  | C | Females | 33.90 | 10.17 | 28.81 | 13.56 | 13.56 |
|  |  | Males | 29.63 | 25.93 | 40.74 | 0.00 | 3.70 |
|  |  | Non-binary gender |  |  |  |  |  |
|  |  | Total | 32.56 | 15.12 | 32.56 | 9.30 | 10.47 |
| **Croatia** | A | Females | 57.14 | 11.04 | 23.59 | 5.19 | 3.03 |
|  |  | Males | 63.75 | 16.25 | 12.50 | 3.75 | 3.75 |
|  |  | Non-binary gender | 100.00 | 0.00 | 0.00 | 0.00 | 0.00 |
|  |  | Total | 58.20 | 11.79 | 21.92 | 4.97 | 3.13 |
|  | B | Females | 39.30 | 11.14 | 28.38 | 10.04 | 11.14 |
|  |  | Males | 52.10 | 10.18 | 22.75 | 5.99 | 8.98 |
|  |  | Non-binary gender | 66.67 | 0.00 | 33.33 | 0.00 | 0.00 |
|  |  | Total | 42.95 | 10.78 | 26.94 | 8.87 | 10.46 |
|  | C | Females | 47.60 | 11.38 | 26.35 | 5.99 | 8.68 |
|  |  | Males | 36.00 | 14.00 | 30.00 | 10.00 | 10.00 |
|  |  | Non-binary gender | 50.00 | 50.00 | 0.00 | 0.00 | 0.00 |
|  |  | Total | 46.11 | 11.92 | 26.68 | 6.48 | 8.81 |
| **Georgia** | A | Females | 58.10 | 14.80 | 21.23 | 2.51 | 3.35 |
|  |  | Males | 46.01 | 20.25 | 24.54 | 4.29 | 4.91 |
|  |  | Non-binary gender | 100.00 | 0.00 | 0.00 | 0.00 | 0.00 |
|  |  | Total | 54.49 | 16.44 | 22.18 | 3.06 | 3.82 |
|  | B | Females | 48.32 | 16.78 | 26.85 | 3.36 | 4.70 |
|  |  | Males | 60.76 | 11.39 | 16.46 | 3.80 | 7.59 |
|  |  | Non-binary gender | 33.33 | 0.00 | 66.67 | 0.00 | 0.00 |
|  |  | Total | 52.38 | 14.72 | 23.81 | 3.46 | 5.63 |
|  | C | Females | 62.30 | 10.47 | 20.42 | 2.09 | 4.71 |
|  |  | Males | 60.53 | 10.53 | 18.42 | 2.63 | 7.89 |
|  |  | Non-binary gender |  |  |  |  |  |
|  |  | Total | 61.74 | 10.43 | 20.00 | 2.17 | 5.65 |
| **Greece** | A | Females | 64.73 | 13.08 | 16.59 | 3.19 | 2.42 |
|  |  | Males | 73.57 | 10.71 | 11.43 | 1.79 | 2.50 |
|  |  | Non-binary gender | 61.54 | 15.38 | 15.38 | 7.69 | 0.00 |
|  |  | Total | 66.75 | 12.55 | 15.38 | 2.91 | 2.41 |
|  | B | Females | 56.56 | 11.30 | 23.63 | 4.77 | 3.74 |
|  |  | Males | 67.73 | 14.79 | 11.74 | 2.44 | 3.30 |
|  |  | Non-binary gender | 51.85 | 7.41 | 33.33 | 7.41 | 0.00 |
|  |  | Total | 60.85 | 12.61 | 19.12 | 3.90 | 3.52 |
|  | C | Females | 48.84 | 15.11 | 26.03 | 5.38 | 4.64 |
|  |  | Males | 62.54 | 13.80 | 17.46 | 2.25 | 3.94 |
|  |  | Non-binary gender | 51.72 | 20.69 | 20.69 | 6.90 | 0.00 |
|  |  | Total | 51.14 | 14.97 | 24.55 | 4.88 | 4.46 |
| **India** | A | Females | 38.68 | 25.47 | 28.30 | 2.83 | 4.72 |
|  |  | Males | 56.41 | 12.82 | 20.51 | 2.56 | 7.69 |
|  |  | Non-binary gender |  |  |  |  |  |
|  |  | Total | 43.45 | 22.07 | 26.21 | 2.76 | 5.52 |
|  | B | Females | 54.39 | 9.65 | 26.32 | 4.39 | 5.26 |
|  |  | Males | 51.64 | 11.48 | 29.51 | 1.64 | 5.74 |
|  |  | Non-binary gender |  |  |  |  |  |
|  |  | Total | 52.97 | 10.59 | 27.97 | 2.97 | 5.51 |
|  | C | Females | 57.47 | 12.64 | 22.99 | 4.60 | 2.30 |
|  |  | Males | 59.62 | 9.62 | 21.15 | 5.77 | 3.85 |
|  |  | Non-binary gender | 0.00 | 0.00 | 100.00 | 0.00 | 0.00 |
|  |  | Total | 57.86 | 11.43 | 22.86 | 5.00 | 2.86 |
| **Latvia** | A | Females | 80.35 | 5.28 | 12.61 | 1.76 | 0.00 |
|  |  | Males | 85.00 | 5.00 | 7.50 | 0.00 | 2.50 |
|  |  | Non-binary gender | 80.00 | 0.00 | 20.00 | 0.00 | 0.00 |
|  |  | Total | 80.83 | 5.18 | 12.18 | 1.55 | 0.26 |
|  | B | Females | 67.00 | 8.87 | 19.21 | 2.46 | 2.46 |
|  |  | Males | 73.17 | 6.50 | 11.38 | 4.07 | 4.88 |
|  |  | Non-binary gender | 100.00 | 0.00 | 0.00 | 0.00 | 0.00 |
|  |  | Total | 69.51 | 7.93 | 16.16 | 3.05 | 3.35 |
|  | C | Females | 63.73 | 11.97 | 20.77 | 2.11 | 1.41 |
|  |  | Males | 63.27 | 6.12 | 26.53 | 2.04 | 2.04 |
|  |  | Non-binary gender |  |  |  |  |  |
|  |  | Total | 63.66 | 11.11 | 21.62 | 2.10 | 1.50 |
| **Lithuania** | A | Females | 85.11 | 7.45 | 5.85 | 1.60 | 0.00 |
|  |  | Males | 85.29 | 8.82 | 2.94 | 0.00 | 2.94 |
|  |  | Non-binary gender | 100.00 | 0.00 | 0.00 | 0.00 | 0.00 |
|  |  | Total | 85.33 | 7.56 | 5.33 | 1.33 | 0.44 |
|  | B | Females | 66.67 | 6.06 | 21.21 | 6.06 | 0.00 |
|  |  | Males | 62.50 | 12.50 | 25.00 | 0.00 | 0.00 |
|  |  | Non-binary gender |  |  |  |  |  |
|  |  | Total | 65.85 | 7.32 | 21.95 | 4.88 | 0.00 |
|  | C | Females | 69.64 | 8.93 | 19.64 | 1.79 | 0.00 |
|  |  | Males | 53.85 | 15.38 | 23.08 | 7.69 | 0.00 |
|  |  | Non-binary gender |  |  |  |  |  |
|  |  | Total | 66.67 | 10.14 | 20.29 | 2.90 | 0.00 |
| **Nigeria** | A | Females | 35.29 | 22.06 | 35.29 | 4.41 | 2.94 |
|  |  | Males | 45.10 | 29.41 | 19.61 | 3.92 | 1.96 |
|  |  | Non-binary gender |  |  |  |  |  |
|  |  | Total | 39.50 | 25.21 | 28.57 | 4.20 | 2.52 |
|  | B | Females | 24.24 | 20.20 | 47.47 | 3.03 | 5.05 |
|  |  | Males | 26.52 | 20.45 | 45.45 | 3.79 | 3.79 |
|  |  | Non-binary gender |  |  |  |  |  |
|  |  | Total | 25.54 | 20.35 | 46.32 | 3.46 | 4.33 |
|  | C | Females | 20.00 | 32.63 | 41.05 | 1.05 | 5.26 |
|  |  | Males | 27.66 | 27.66 | 41.84 | 0.71 | 2.13 |
|  |  | Non-binary gender | 100.00 | 0.00 | 0.00 | 0.00 | 0.00 |
|  |  | Total | 24.89 | 29.54 | 41.35 | 0.84 | 3.38 |
| **Russia** | A | Females | 68.11 | 6.44 | 19.01 | 4.94 | 1.50 |
|  |  | Males | 64.93 | 7.46 | 22.39 | 2.99 | 2.24 |
|  |  | Non-binary gender | 62.50 | 18.75 | 12.50 | 0.00 | 6.25 |
|  |  | Total | 67.48 | 6.85 | 19.44 | 4.52 | 1.71 |
|  | B | Females | 56.25 | 9.38 | 22.66 | 10.16 | 1.56 |
|  |  | Males | 49.58 | 13.45 | 30.25 | 4.20 | 2.52 |
|  |  | Non-binary gender | 71.43 | 28.57 | 0.00 | 0.00 | 0.00 |
|  |  | Total | 53.54 | 11.81 | 25.59 | 7.09 | 1.97 |
|  | C | Females | 56.81 | 14.79 | 20.62 | 5.06 | 2.72 |
|  |  | Males | 62.07 | 9.20 | 19.54 | 4.60 | 4.60 |
|  |  | Non-binary gender | 88.89 | 0.00 | 11.11 | 0.00 | 0.00 |
|  |  | Total | 58.92 | 13.03 | 20.11 | 4.82 | 3.12 |
| **Malaysia** | A | Females | 62.20 | 12.20 | 14.63 | 9.76 | 1.22 |
|  |  | Males | 46.15 | 23.08 | 7.69 | 15.38 | 7.69 |
|  |  | Non-binary gender | 100.00 | 0.00 | 0.00 | 0.00 | 0.00 |
|  |  | Total | 60.42 | 13.54 | 13.54 | 10.42 | 2.08 |
|  | B | Females | 18.75 | 12.50 | 25.00 | 43.75 | 0.00 |
|  |  | Males | 70.00 | 20.00 | 0.00 | 10.00 | 0.00 |
|  |  | Non-binary gender |  |  |  |  |  |
|  |  | Total | 30.95 | 14.29 | 19.05 | 35.71 | 0.00 |
|  | C | Females | 30.77 | 20.00 | 13.85 | 29.23 | 6.15 |
|  |  | Males | 21.43 | 28.57 | 14.29 | 21.43 | 14.29 |
|  |  | Non-binary gender | 100.00 | 0.00 | 0.00 | 0.00 | 0.00 |
|  |  | Total | 30.00 | 21.25 | 13.75 | 27.50 | 7.50 |
| **Hungary** | A | Females | 87.50 | 6.25 | 3.13 | 0.00 | 3.13 |
|  |  | Males | 100.00 | 0.00 | 0.00 | 0.00 | 0.00 |
|  |  | Non-binary gender |  |  |  |  |  |
|  |  | Total | 90.24 | 4.88 | 2.44 | 0.00 | 2.44 |
|  | B | Females | 70.00 | 0.00 | 20.00 | 10.00 | 0.00 |
|  |  | Males | 75.00 | 0.00 | 25.00 | 0.00 | 0.00 |
|  |  | Non-binary gender |  |  |  |  |  |
|  |  | Total | 71.43 | 0.00 | 21.43 | 7.14 | 0.00 |
|  | C | Females | 75.86 | 6.90 | 17.24 | 0.00 | 0.00 |
|  |  | Males | 66.67 | 0.00 | 33.33 | 0.00 | 0.00 |
|  |  | Non-binary gender |  |  |  |  |  |
|  |  | Total | 75.00 | 6.25 | 18.75 | 0.00 | 0.00 |
| **TOTAL** |  |  | 57.03 | 12.81 | 21.77 | 4.47 | 3.92 |
| **J2. Do you believe that COVID-19 was created in a laboratory?** | | | | | | | |
| **Bulgaria** | A | Females | 36.17 | 10.64 | 29.79 | 8.51 | 14.89 |
|  |  | Males | 48.89 | 22.22 | 15.56 | 6.67 | 6.67 |
|  |  | Non-binary gender | 100.00 | 0.00 | 0.00 | 0.00 | 0.00 |
|  |  | Total | 43.62 | 15.96 | 22.34 | 7.45 | 10.64 |
|  | B | Females | 12.50 | 8.93 | 23.21 | 16.07 | 39.29 |
|  |  | Males | 13.33 | 6.67 | 33.33 | 26.67 | 20.00 |
|  |  | Non-binary gender |  |  |  |  |  |
|  |  | Total | 12.79 | 8.14 | 26.74 | 19.77 | 32.56 |
|  | C | Females | 8.47 | 5.08 | 35.59 | 30.51 | 20.34 |
|  |  | Males | 25.93 | 18.52 | 37.04 | 11.11 | 7.41 |
|  |  | Non-binary gender |  |  |  |  |  |
|  |  | Total | 13.95 | 9.30 | 36.05 | 24.42 | 16.28 |
| **Croatia** | A | Females | 30.95 | 19.26 | 25.76 | 11.90 | 12.12 |
|  |  | Males | 38.75 | 16.25 | 23.75 | 10.00 | 11.25 |
|  |  | Non-binary gender | 100.00 | 0.00 | 0.00 | 0.00 | 0.00 |
|  |  | Total | 32.23 | 18.78 | 25.41 | 11.60 | 11.97 |
|  | B | Females | 17.03 | 13.10 | 26.64 | 18.12 | 25.11 |
|  |  | Males | 29.34 | 10.18 | 27.54 | 16.17 | 16.77 |
|  |  | Non-binary gender | 50.00 | 0.00 | 16.67 | 16.67 | 16.67 |
|  |  | Total | 20.60 | 12.20 | 26.78 | 17.59 | 22.82 |
|  | C | Females | 23.05 | 16.17 | 28.74 | 13.17 | 18.86 |
|  |  | Males | 18.00 | 28.00 | 22.00 | 10.00 | 22.00 |
|  |  | Non-binary gender | 0.00 | 50.00 | 0.00 | 50.00 | 0.00 |
|  |  | Total | 22.28 | 17.88 | 27.72 | 12.95 | 19.17 |
| **Georgia** | A | Females | 26.54 | 20.95 | 31.56 | 9.50 | 11.45 |
|  |  | Males | 20.86 | 15.95 | 29.45 | 15.34 | 18.40 |
|  |  | Non-binary gender | 0.00 | 50.00 | 50.00 | 0.00 | 0.00 |
|  |  | Total | 24.67 | 19.50 | 30.98 | 11.28 | 13.58 |
|  | B | Females | 28.86 | 19.46 | 25.50 | 11.41 | 14.77 |
|  |  | Males | 27.85 | 18.99 | 31.65 | 12.66 | 8.86 |
|  |  | Non-binary gender | 33.33 | 33.33 | 0.00 | 33.33 | 0.00 |
|  |  | Total | 28.57 | 19.48 | 27.27 | 12.12 | 12.55 |
|  | C | Females | 21.99 | 21.47 | 29.84 | 14.14 | 12.57 |
|  |  | Males | 31.58 | 10.53 | 36.84 | 10.53 | 10.53 |
|  |  | Non-binary gender | 0.00 | 0.00 | 0.00 | 0.00 | 100.00 |
|  |  | Total | 23.48 | 19.57 | 30.87 | 13.48 | 12.61 |
| **Greece** | A | Females | 36.37 | 20.77 | 26.59 | 9.56 | 6.70 |
|  |  | Males | 51.43 | 17.50 | 19.29 | 6.79 | 5.00 |
|  |  | Non-binary gender | 61.54 | 7.69 | 23.08 | 0.00 | 7.69 |
|  |  | Total | 40.15 | 19.87 | 24.85 | 8.81 | 6.32 |
|  | B | Females | 32.14 | 18.93 | 25.54 | 13.44 | 9.94 |
|  |  | Males | 45.48 | 21.27 | 20.05 | 6.97 | 6.23 |
|  |  | Non-binary gender | 37.04 | 22.22 | 25.93 | 11.11 | 3.70 |
|  |  | Total | 37.39 | 19.89 | 23.41 | 10.89 | 8.42 |
|  | C | Females | 25.47 | 20.88 | 28.75 | 12.62 | 12.28 |
|  |  | Males | 35.49 | 20.28 | 25.07 | 8.73 | 10.42 |
|  |  | Non-binary gender | 51.72 | 17.24 | 20.69 | 3.45 | 6.90 |
|  |  | Total | 27.48 | 20.73 | 28.03 | 11.85 | 11.90 |
| **India** | A | Females | 19.81 | 29.25 | 33.96 | 11.32 | 5.66 |
|  |  | Males | 20.51 | 23.08 | 30.77 | 12.82 | 12.82 |
|  |  | Non-binary gender |  |  |  |  |  |
|  |  | Total | 20.00 | 27.59 | 33.10 | 11.72 | 7.59 |
|  | B | Females | 19.30 | 17.54 | 33.33 | 17.54 | 12.28 |
|  |  | Males | 16.39 | 10.66 | 40.98 | 18.85 | 13.11 |
|  |  | Non-binary gender |  |  |  |  |  |
|  |  | Total | 17.80 | 13.98 | 37.29 | 18.22 | 12.71 |
|  | C | Females | 31.03 | 22.99 | 25.29 | 9.20 | 11.49 |
|  |  | Males | 23.08 | 17.31 | 38.46 | 13.46 | 7.69 |
|  |  | Non-binary gender | 0.00 | 0.00 | 0.00 | 100.00 | 0.00 |
|  |  | Total | 27.86 | 20.71 | 30.00 | 11.43 | 10.00 |
| **Latvia** | A | Females | 45.75 | 19.06 | 24.05 | 9.09 | 2.05 |
|  |  | Males | 47.50 | 12.50 | 35.00 | 0.00 | 5.00 |
|  |  | Non-binary gender | 60.00 | 20.00 | 20.00 | 0.00 | 0.00 |
|  |  | Total | 46.11 | 18.39 | 25.13 | 8.03 | 2.33 |
|  | B | Females | 26.60 | 19.70 | 35.47 | 9.85 | 8.37 |
|  |  | Males | 30.89 | 11.38 | 34.96 | 10.57 | 12.20 |
|  |  | Non-binary gender | 0.00 | 50.00 | 50.00 | 0.00 | 0.00 |
|  |  | Total | 28.05 | 16.77 | 35.37 | 10.06 | 9.76 |
|  | C | Females | 27.82 | 25.35 | 28.17 | 11.27 | 7.39 |
|  |  | Males | 46.94 | 10.20 | 20.41 | 14.29 | 8.16 |
|  |  | Non-binary gender |  |  |  |  |  |
|  |  | Total | 30.63 | 23.12 | 27.03 | 11.71 | 7.51 |
| **Lithuania** | A | Females | 60.64 | 22.34 | 11.70 | 4.79 | 0.53 |
|  |  | Males | 70.59 | 14.71 | 11.76 | 0.00 | 2.94 |
|  |  | Non-binary gender | 66.67 | 0.00 | 0.00 | 0.00 | 33.33 |
|  |  | Total | 62.22 | 20.89 | 11.56 | 4.00 | 1.33 |
|  | B | Females | 27.27 | 27.27 | 27.27 | 9.09 | 9.09 |
|  |  | Males | 37.50 | 37.50 | 12.50 | 12.50 | 0.00 |
|  |  | Non-binary gender |  |  |  |  |  |
|  |  | Total | 29.27 | 29.27 | 24.39 | 9.76 | 7.32 |
|  | C | Females | 48.21 | 21.43 | 19.64 | 3.57 | 7.14 |
|  |  | Males | 61.54 | 7.69 | 23.08 | 7.69 | 0.00 |
|  |  | Non-binary gender |  |  |  |  |  |
|  |  | Total | 50.72 | 18.84 | 20.29 | 4.35 | 5.80 |
| **Nigeria** | A | Females | 26.47 | 20.59 | 36.76 | 7.35 | 8.82 |
|  |  | Males | 41.18 | 19.61 | 31.37 | 5.88 | 1.96 |
|  |  | Non-binary gender |  |  |  |  |  |
|  |  | Total | 32.77 | 20.17 | 34.45 | 6.72 | 5.88 |
|  | B | Females | 30.30 | 25.25 | 34.34 | 5.05 | 5.05 |
|  |  | Males | 37.88 | 15.15 | 37.88 | 6.06 | 3.03 |
|  |  | Non-binary gender |  |  |  |  |  |
|  |  | Total | 34.63 | 19.48 | 36.36 | 5.63 | 3.90 |
|  | C | Females | 38.95 | 22.11 | 32.63 | 3.16 | 3.16 |
|  |  | Males | 41.13 | 21.28 | 34.04 | 0.71 | 2.84 |
|  |  | Non-binary gender | 0.00 | 100.00 | 0.00 | 0.00 | 0.00 |
|  |  | Total | 40.08 | 21.94 | 33.33 | 1.69 | 2.95 |
| **Russia** | A | Females | 31.14 | 18.86 | 31.29 | 13.77 | 4.94 |
|  |  | Males | 31.34 | 23.13 | 35.07 | 6.72 | 3.73 |
|  |  | Non-binary gender | 31.25 | 31.25 | 6.25 | 18.75 | 12.50 |
|  |  | Total | 31.17 | 19.80 | 31.42 | 12.71 | 4.89 |
|  | B | Females | 23.44 | 18.75 | 37.50 | 15.63 | 4.69 |
|  |  | Males | 31.09 | 15.97 | 36.97 | 11.76 | 4.20 |
|  |  | Non-binary gender | 71.43 | 14.29 | 14.29 | 0.00 | 0.00 |
|  |  | Total | 28.35 | 17.32 | 36.61 | 13.39 | 4.33 |
|  | C | Females | 28.02 | 17.12 | 37.35 | 11.28 | 6.23 |
|  |  | Males | 36.78 | 13.79 | 28.74 | 14.94 | 5.75 |
|  |  | Non-binary gender | 44.44 | 11.11 | 44.44 | 0.00 | 0.00 |
|  |  | Total | 30.59 | 16.15 | 35.41 | 11.90 | 5.95 |
| **Malaysia** | A | Females | 39.02 | 28.05 | 12.20 | 13.41 | 7.32 |
|  |  | Males | 30.77 | 15.38 | 15.38 | 30.77 | 7.69 |
|  |  | Non-binary gender | 100.00 | 0.00 | 0.00 | 0.00 | 0.00 |
|  |  | Total | 38.54 | 26.04 | 12.50 | 15.63 | 7.29 |
|  | B | Females | 28.13 | 6.25 | 28.13 | 31.25 | 6.25 |
|  |  | Males | 50.00 | 20.00 | 10.00 | 20.00 | 0.00 |
|  |  | Non-binary gender |  |  |  |  |  |
|  |  | Total | 33.33 | 9.52 | 23.81 | 28.57 | 4.76 |
|  | C | Females | 27.69 | 20.00 | 13.85 | 30.77 | 7.69 |
|  |  | Males | 21.43 | 0.00 | 21.43 | 42.86 | 14.29 |
|  |  | Non-binary gender | 100.00 | 0.00 | 0.00 | 0.00 | 0.00 |
|  |  | Total | 27.50 | 16.25 | 15.00 | 32.50 | 8.75 |
| **Hungary** | A | Females | 65.63 | 9.38 | 9.38 | 9.38 | 6.25 |
|  |  | Males | 55.56 | 33.33 | 11.11 | 0.00 | 0.00 |
|  |  | Non-binary gender |  |  |  |  |  |
|  |  | Total | 63.41 | 14.63 | 9.76 | 7.32 | 4.88 |
|  | B | Females | 30.00 | 0.00 | 50.00 | 10.00 | 10.00 |
|  |  | Males | 75.00 | 25.00 | 0.00 | 0.00 | 0.00 |
|  |  | Non-binary gender |  |  |  |  |  |
|  |  | Total | 42.86 | 7.14 | 35.71 | 7.14 | 7.14 |
|  | C | Females | 48.28 | 13.79 | 31.03 | 3.45 | 3.45 |
|  |  | Males | 66.67 | 0.00 | 33.33 | 0.00 | 0.00 |
|  |  | Non-binary gender |  |  |  |  |  |
|  |  | Total | 50.00 | 12.50 | 31.25 | 3.13 | 3.13 |
| **TOTAL** |  |  | 32.06 | 19.12 | 27.57 | 11.49 | 9.76 |
| **J3. Do you think that COVID-19 was created to be used as a biochemical weapon for the extermination of the human population?** | | | | | | | |
| **Bulgaria** | A | Females | 48.94 | 8.51 | 23.40 | 4.26 | 14.89 |
|  |  | Males | 68.89 | 15.56 | 8.89 | 2.22 | 4.44 |
|  |  | Non-binary gender | 100.00 | 0.00 | 0.00 | 0.00 | 0.00 |
|  |  | Total | 59.57 | 11.70 | 15.96 | 3.19 | 9.57 |
|  | B | Females | 26.79 | 14.29 | 21.43 | 12.50 | 25.00 |
|  |  | Males | 40.00 | 26.67 | 13.33 | 10.00 | 10.00 |
|  |  | Non-binary gender |  |  |  |  |  |
|  |  | Total | 31.40 | 18.60 | 18.60 | 11.63 | 19.77 |
|  | C | Females | 22.03 | 5.08 | 35.59 | 20.34 | 16.95 |
|  |  | Males | 44.44 | 14.81 | 37.04 | 3.70 | 0.00 |
|  |  | Non-binary gender |  |  |  |  |  |
|  |  | Total | 29.07 | 8.14 | 36.05 | 15.12 | 11.63 |
| **Croatia** | A | Females | 58.44 | 13.64 | 15.37 | 7.36 | 5.19 |
|  |  | Males | 72.50 | 11.25 | 7.50 | 7.50 | 1.25 |
|  |  | Non-binary gender | 100.00 | 0.00 | 0.00 | 0.00 | 0.00 |
|  |  | Total | 60.59 | 13.26 | 14.18 | 7.37 | 4.60 |
|  | B | Females | 34.93 | 14.41 | 23.36 | 12.23 | 15.07 |
|  |  | Males | 61.08 | 16.17 | 11.38 | 4.79 | 6.59 |
|  |  | Non-binary gender | 50.00 | 0.00 | 0.00 | 33.33 | 16.67 |
|  |  | Total | 42.00 | 14.74 | 19.97 | 10.46 | 12.84 |
|  | C | Females | 40.42 | 17.07 | 23.65 | 6.59 | 12.28 |
|  |  | Males | 56.00 | 16.00 | 6.00 | 14.00 | 8.00 |
|  |  | Non-binary gender | 50.00 | 0.00 | 50.00 | 0.00 | 0.00 |
|  |  | Total | 42.49 | 16.84 | 21.50 | 7.51 | 11.66 |
| **Georgia** | A | Females | 39.66 | 22.63 | 19.83 | 8.10 | 9.78 |
|  |  | Males | 39.26 | 18.40 | 22.09 | 11.04 | 9.20 |
|  |  | Non-binary gender | 50.00 | 50.00 | 0.00 | 0.00 | 0.00 |
|  |  | Total | 39.58 | 21.41 | 20.46 | 8.99 | 9.56 |
|  | B | Females | 42.28 | 18.79 | 19.46 | 6.71 | 12.75 |
|  |  | Males | 51.90 | 21.52 | 17.72 | 5.06 | 3.80 |
|  |  | Non-binary gender | 33.33 | 0.00 | 66.67 | 0.00 | 0.00 |
|  |  | Total | 45.45 | 19.48 | 19.48 | 6.06 | 9.52 |
|  | C | Females | 41.36 | 19.90 | 20.94 | 7.33 | 10.47 |
|  |  | Males | 60.53 | 15.79 | 15.79 | 5.26 | 2.63 |
|  |  | Non-binary gender | 0.00 | 0.00 | 0.00 | 0.00 | 100.00 |
|  |  | Total | 44.35 | 19.13 | 20.00 | 6.96 | 9.57 |
| **Greece** | A | Females | 62.20 | 14.62 | 14.95 | 5.49 | 2.75 |
|  |  | Males | 74.29 | 12.14 | 7.86 | 3.57 | 2.14 |
|  |  | Non-binary gender | 53.85 | 38.46 | 7.69 | 0.00 | 0.00 |
|  |  | Total | 64.92 | 14.30 | 13.22 | 4.99 | 2.58 |
|  | B | Females | 55.45 | 15.59 | 16.79 | 6.76 | 5.41 |
|  |  | Males | 74.82 | 10.64 | 9.17 | 2.44 | 2.93 |
|  |  | Non-binary gender | 59.26 | 18.52 | 14.81 | 7.41 | 0.00 |
|  |  | Total | 63.04 | 13.70 | 13.80 | 5.09 | 4.38 |
|  | C | Females | 47.43 | 16.86 | 19.19 | 9.90 | 6.62 |
|  |  | Males | 62.25 | 13.80 | 13.80 | 5.07 | 5.07 |
|  |  | Non-binary gender | 68.97 | 10.34 | 10.34 | 3.45 | 6.90 |
|  |  | Total | 50.16 | 16.27 | 18.18 | 9.02 | 6.37 |
| **India** | A | Females | 25.47 | 26.42 | 33.96 | 10.38 | 3.77 |
|  |  | Males | 17.95 | 20.51 | 41.03 | 10.26 | 10.26 |
|  |  | Non-binary gender |  |  |  |  |  |
|  |  | Total | 23.45 | 24.83 | 35.86 | 10.34 | 5.52 |
|  | B | Females | 25.44 | 12.28 | 37.72 | 11.40 | 13.16 |
|  |  | Males | 27.87 | 13.11 | 31.97 | 16.39 | 10.66 |
|  |  | Non-binary gender |  |  |  |  |  |
|  |  | Total | 26.69 | 12.71 | 34.75 | 13.98 | 11.86 |
|  | C | Females | 44.83 | 14.94 | 22.99 | 10.34 | 6.90 |
|  |  | Males | 30.77 | 11.54 | 30.77 | 21.15 | 5.77 |
|  |  | Non-binary gender | 0.00 | 0.00 | 0.00 | 0.00 | 100.00 |
|  |  | Total | 39.29 | 13.57 | 25.71 | 14.29 | 7.14 |
| **Latvia** | A | Females | 58.94 | 14.96 | 16.13 | 7.04 | 2.93 |
|  |  | Males | 60.00 | 20.00 | 17.50 | 0.00 | 2.50 |
|  |  | Non-binary gender | 80.00 | 0.00 | 20.00 | 0.00 | 0.00 |
|  |  | Total | 59.33 | 15.28 | 16.32 | 6.22 | 2.85 |
|  | B | Females | 39.41 | 18.72 | 28.08 | 5.42 | 8.37 |
|  |  | Males | 51.22 | 15.45 | 23.58 | 4.88 | 4.88 |
|  |  | Non-binary gender | 0.00 | 100.00 | 0.00 | 0.00 | 0.00 |
|  |  | Total | 43.60 | 17.99 | 26.22 | 5.18 | 7.01 |
|  | C | Females | 39.79 | 23.59 | 18.31 | 12.32 | 5.99 |
|  |  | Males | 53.06 | 8.16 | 28.57 | 6.12 | 4.08 |
|  |  | Non-binary gender |  |  |  |  |  |
|  |  | Total | 41.74 | 21.32 | 19.82 | 11.41 | 5.71 |
| **Lithuania** | A | Females | 77.66 | 10.64 | 9.57 | 2.13 | 0.00 |
|  |  | Males | 79.41 | 11.76 | 5.88 | 0.00 | 2.94 |
|  |  | Non-binary gender | 66.67 | 0.00 | 0.00 | 33.33 | 0.00 |
|  |  | Total | 77.78 | 10.67 | 8.89 | 2.22 | 0.44 |
|  | B | Females | 57.58 | 21.21 | 9.09 | 3.03 | 9.09 |
|  |  | Males | 75.00 | 12.50 | 12.50 | 0.00 | 0.00 |
|  |  | Non-binary gender |  |  |  |  |  |
|  |  | Total | 60.98 | 19.51 | 9.76 | 2.44 | 7.32 |
|  | C | Females | 55.36 | 23.21 | 12.50 | 1.79 | 7.14 |
|  |  | Males | 69.23 | 15.38 | 7.69 | 7.69 | 0.00 |
|  |  | Non-binary gender |  |  |  |  |  |
|  |  | Total | 57.97 | 21.74 | 11.59 | 2.90 | 5.80 |
| **Nigeria** | A | Females | 30.88 | 14.71 | 38.24 | 8.82 | 7.35 |
|  |  | Males | 47.06 | 29.41 | 19.61 | 1.96 | 1.96 |
|  |  | Non-binary gender |  |  |  |  |  |
|  |  | Total | 37.82 | 21.01 | 30.25 | 5.88 | 5.04 |
|  | B | Females | 29.29 | 17.17 | 41.41 | 6.06 | 6.06 |
|  |  | Males | 34.09 | 18.18 | 39.39 | 3.79 | 4.55 |
|  |  | Non-binary gender |  |  |  |  |  |
|  |  | Total | 32.03 | 17.75 | 40.26 | 4.76 | 5.19 |
|  | C | Females | 44.21 | 16.84 | 31.58 | 2.11 | 5.26 |
|  |  | Males | 40.43 | 21.99 | 33.33 | 1.42 | 2.84 |
|  |  | Non-binary gender | 0.00 | 0.00 | 100.00 | 0.00 | 0.00 |
|  |  | Total | 41.77 | 19.83 | 32.91 | 1.69 | 3.80 |
| **Russia** | A | Females | 48.20 | 15.57 | 23.05 | 9.28 | 3.89 |
|  |  | Males | 50.75 | 14.93 | 26.87 | 4.48 | 2.99 |
|  |  | Non-binary gender | 43.75 | 12.50 | 31.25 | 0.00 | 12.50 |
|  |  | Total | 48.53 | 15.40 | 23.84 | 8.31 | 3.91 |
|  | B | Females | 38.28 | 19.53 | 28.91 | 10.16 | 3.13 |
|  |  | Males | 47.06 | 18.49 | 26.89 | 3.36 | 4.20 |
|  |  | Non-binary gender | 71.43 | 0.00 | 14.29 | 14.29 | 0.00 |
|  |  | Total | 43.31 | 18.50 | 27.56 | 7.09 | 3.54 |
|  | C | Females | 38.13 | 18.68 | 23.74 | 13.23 | 6.23 |
|  |  | Males | 55.17 | 11.49 | 18.39 | 8.05 | 6.90 |
|  |  | Non-binary gender | 66.67 | 11.11 | 11.11 | 0.00 | 11.11 |
|  |  | Total | 43.06 | 16.71 | 22.10 | 11.61 | 6.52 |
| **Malaysia** | A | Females | 51.22 | 19.51 | 13.41 | 9.76 | 6.10 |
|  |  | Males | 38.46 | 15.38 | 15.38 | 23.08 | 7.69 |
|  |  | Non-binary gender | 100.00 | 0.00 | 0.00 | 0.00 | 0.00 |
|  |  | Total | 50.00 | 18.75 | 13.54 | 11.46 | 6.25 |
|  | B | Females | 28.13 | 15.63 | 21.88 | 31.25 | 3.13 |
|  |  | Males | 50.00 | 10.00 | 10.00 | 30.00 | 0.00 |
|  |  | Non-binary gender |  |  |  |  |  |
|  |  | Total | 33.33 | 14.29 | 19.05 | 30.95 | 2.38 |
|  | C | Females | 35.38 | 21.54 | 7.69 | 27.69 | 7.69 |
|  |  | Males | 14.29 | 7.14 | 35.71 | 21.43 | 21.43 |
|  |  | Non-binary gender | 100.00 | 0.00 | 0.00 | 0.00 | 0.00 |
|  |  | Total | 32.50 | 18.75 | 12.50 | 26.25 | 10.00 |
| **Hungary** | A | Females | 81.25 | 6.25 | 0.00 | 6.25 | 6.25 |
|  |  | Males | 100.00 | 0.00 | 0.00 | 0.00 | 0.00 |
|  |  | Non-binary gender |  |  |  |  |  |
|  |  | Total | 85.37 | 4.88 | 0.00 | 4.88 | 4.88 |
|  | B | Females | 40.00 | 30.00 | 30.00 | 0.00 | 0.00 |
|  |  | Males | 100.00 | 0.00 | 0.00 | 0.00 | 0.00 |
|  |  | Non-binary gender |  |  |  |  |  |
|  |  | Total | 57.14 | 21.43 | 21.43 | 0.00 | 0.00 |
|  | C | Females | 65.52 | 10.34 | 20.69 | 3.45 | 0.00 |
|  |  | Males | 100.00 | 0.00 | 0.00 | 0.00 | 0.00 |
|  |  | Non-binary gender |  |  |  |  |  |
|  |  | Total | 68.75 | 9.38 | 18.75 | 3.13 | 0.00 |
| **TOTAL** |  |  | 51.24 | 15.92 | 19.17 | 7.62 | 6.05 |
| **J4. Do you believe that COVID-19 is related to the 5G technology antenna?** | | | | | | | |
| **Bulgaria** | A | Females | 85.11 | 6.38 | 4.26 | 4.26 | 0.00 |
|  |  | Males | 93.33 | 0.00 | 4.44 | 2.22 | 0.00 |
|  |  | Non-binary gender | 100.00 | 0.00 | 0.00 | 0.00 | 0.00 |
|  |  | Total | 89.36 | 3.19 | 4.26 | 3.19 | 0.00 |
|  | B | Females | 85.71 | 1.79 | 3.57 | 0.00 | 8.93 |
|  |  | Males | 93.33 | 0.00 | 3.33 | 3.33 | 0.00 |
|  |  | Non-binary gender |  |  |  |  |  |
|  |  | Total | 88.37 | 1.16 | 3.49 | 1.16 | 5.81 |
|  | C | Females | 64.41 | 6.78 | 15.25 | 6.78 | 6.78 |
|  |  | Males | 59.26 | 11.11 | 22.22 | 3.70 | 3.70 |
|  |  | Non-binary gender |  |  |  |  |  |
|  |  | Total | 62.79 | 8.14 | 17.44 | 5.81 | 5.81 |
| **Croatia** | A | Females | 86.80 | 7.58 | 4.98 | 0.65 | 0.00 |
|  |  | Males | 95.00 | 1.25 | 3.75 | 0.00 | 0.00 |
|  |  | Non-binary gender | 100.00 | 0.00 | 0.00 | 0.00 | 0.00 |
|  |  | Total | 88.03 | 6.63 | 4.79 | 0.55 | 0.00 |
|  | B | Females | 77.73 | 8.30 | 7.64 | 3.06 | 3.28 |
|  |  | Males | 91.02 | 2.40 | 4.19 | 1.80 | 0.60 |
|  |  | Non-binary gender | 83.33 | 0.00 | 16.67 | 0.00 | 0.00 |
|  |  | Total | 81.30 | 6.66 | 6.81 | 2.69 | 2.54 |
|  | C | Females | 82.63 | 6.59 | 6.59 | 1.80 | 2.40 |
|  |  | Males | 82.00 | 6.00 | 8.00 | 2.00 | 2.00 |
|  |  | Non-binary gender | 100.00 | 0.00 | 0.00 | 0.00 | 0.00 |
|  |  | Total | 82.64 | 6.48 | 6.74 | 1.81 | 2.33 |
| **Georgia** | A | Females | 76.54 | 9.50 | 11.17 | 1.12 | 1.68 |
|  |  | Males | 77.91 | 9.82 | 7.36 | 2.45 | 2.45 |
|  |  | Non-binary gender | 100.00 | 0.00 | 0.00 | 0.00 | 0.00 |
|  |  | Total | 77.06 | 9.56 | 9.94 | 1.53 | 1.91 |
|  | B | Females | 79.19 | 12.08 | 5.37 | 0.67 | 2.68 |
|  |  | Males | 82.28 | 6.33 | 7.59 | 3.80 | 0.00 |
|  |  | Non-binary gender | 66.67 | 33.33 | 0.00 | 0.00 | 0.00 |
|  |  | Total | 80.09 | 10.39 | 6.06 | 1.73 | 1.73 |
|  | C | Females | 76.96 | 11.52 | 7.33 | 1.57 | 2.62 |
|  |  | Males | 92.11 | 5.26 | 2.63 | 0.00 | 0.00 |
|  |  | Non-binary gender | 0.00 | 0.00 | 0.00 | 100.00 | 0.00 |
|  |  | Total | 79.13 | 10.43 | 6.52 | 1.74 | 2.17 |
| **Greece** | A | Females | 91.76 | 3.08 | 4.18 | 0.44 | 0.55 |
|  |  | Males | 95.00 | 1.79 | 2.50 | 0.36 | 0.36 |
|  |  | Non-binary gender | 92.31 | 0.00 | 0.00 | 7.69 | 0.00 |
|  |  | Total | 92.52 | 2.74 | 3.74 | 0.50 | 0.50 |
|  | B | Females | 90.29 | 4.30 | 4.30 | 0.56 | 0.56 |
|  |  | Males | 95.48 | 2.44 | 0.98 | 0.37 | 0.73 |
|  |  | Non-binary gender | 92.59 | 3.70 | 3.70 | 0.00 | 0.00 |
|  |  | Total | 92.34 | 3.57 | 3.00 | 0.48 | 0.62 |
|  | C | Females | 84.38 | 5.72 | 7.41 | 1.87 | 0.62 |
|  |  | Males | 91.83 | 2.25 | 4.79 | 0.56 | 0.56 |
|  |  | Non-binary gender | 93.10 | 3.45 | 3.45 | 0.00 | 0.00 |
|  |  | Total | 85.73 | 5.11 | 6.93 | 1.63 | 0.60 |
| **India** | A | Females | 41.51 | 21.70 | 27.36 | 8.49 | 0.94 |
|  |  | Males | 41.03 | 23.08 | 28.21 | 5.13 | 2.56 |
|  |  | Non-binary gender |  |  |  |  |  |
|  |  | Total | 41.38 | 22.07 | 27.59 | 7.59 | 1.38 |
|  | B | Females | 58.77 | 10.53 | 21.93 | 5.26 | 3.51 |
|  |  | Males | 66.39 | 9.02 | 17.21 | 6.56 | 0.82 |
|  |  | Non-binary gender |  |  |  |  |  |
|  |  | Total | 62.71 | 9.75 | 19.49 | 5.93 | 2.12 |
|  | C | Females | 66.67 | 13.79 | 17.24 | 2.30 | 0.00 |
|  |  | Males | 63.46 | 7.69 | 23.08 | 3.85 | 1.92 |
|  |  | Non-binary gender | 0.00 | 0.00 | 0.00 | 100.00 | 0.00 |
|  |  | Total | 65.00 | 11.43 | 19.29 | 3.57 | 0.71 |
| **Latvia** | A | Females | 95.01 | 2.35 | 2.05 | 0.59 | 0.00 |
|  |  | Males | 97.50 | 2.50 | 0.00 | 0.00 | 0.00 |
|  |  | Non-binary gender | 100.00 | 0.00 | 0.00 | 0.00 | 0.00 |
|  |  | Total | 95.34 | 2.33 | 1.81 | 0.52 | 0.00 |
|  | B | Females | 87.19 | 3.45 | 6.40 | 0.99 | 1.97 |
|  |  | Males | 94.31 | 2.44 | 1.63 | 0.81 | 0.81 |
|  |  | Non-binary gender | 100.00 | 0.00 | 0.00 | 0.00 | 0.00 |
|  |  | Total | 89.94 | 3.05 | 4.57 | 0.91 | 1.52 |
|  | C | Females | 89.79 | 4.93 | 4.58 | 0.70 | 0.00 |
|  |  | Males | 93.88 | 0.00 | 6.12 | 0.00 | 0.00 |
|  |  | Non-binary gender |  |  |  |  |  |
|  |  | Total | 90.39 | 4.20 | 4.80 | 0.60 | 0.00 |
| **Lithuania** | A | Females | 95.21 | 2.66 | 2.13 | 0.00 | 0.00 |
|  |  | Males | 97.06 | 2.94 | 0.00 | 0.00 | 0.00 |
|  |  | Non-binary gender | 100.00 | 0.00 | 0.00 | 0.00 | 0.00 |
|  |  | Total | 95.56 | 2.67 | 1.78 | 0.00 | 0.00 |
|  | B | Females | 84.85 | 3.03 | 12.12 | 0.00 | 0.00 |
|  |  | Males | 87.50 | 0.00 | 12.50 | 0.00 | 0.00 |
|  |  | Non-binary gender |  |  |  |  |  |
|  |  | Total | 85.37 | 2.44 | 12.20 | 0.00 | 0.00 |
|  | C | Females | 91.07 | 5.36 | 1.79 | 1.79 | 0.00 |
|  |  | Males | 84.62 | 15.38 | 0.00 | 0.00 | 0.00 |
|  |  | Non-binary gender |  |  |  |  |  |
|  |  | Total | 89.86 | 7.25 | 1.45 | 1.45 | 0.00 |
| **Nigeria** | A | Females | 60.29 | 13.24 | 20.59 | 5.88 | 0.00 |
|  |  | Males | 68.63 | 15.69 | 13.73 | 0.00 | 1.96 |
|  |  | Non-binary gender |  |  |  |  |  |
|  |  | Total | 63.87 | 14.29 | 17.65 | 3.36 | 0.84 |
|  | B | Females | 50.51 | 19.19 | 26.26 | 2.02 | 2.02 |
|  |  | Males | 52.27 | 18.94 | 24.24 | 3.03 | 1.52 |
|  |  | Non-binary gender |  |  |  |  |  |
|  |  | Total | 51.52 | 19.05 | 25.11 | 2.60 | 1.73 |
|  | C | Females | 53.68 | 18.95 | 22.11 | 3.16 | 2.11 |
|  |  | Males | 56.03 | 14.89 | 25.53 | 0.00 | 3.55 |
|  |  | Non-binary gender | 0.00 | 0.00 | 0.00 | 100.00 | 0.00 |
|  |  | Total | 54.85 | 16.46 | 24.05 | 1.69 | 2.95 |
| **Russia** | A | Females | 82.93 | 5.09 | 10.18 | 0.75 | 1.05 |
|  |  | Males | 81.34 | 7.46 | 8.21 | 2.24 | 0.75 |
|  |  | Non-binary gender | 62.50 | 6.25 | 25.00 | 6.25 | 0.00 |
|  |  | Total | 82.27 | 5.50 | 10.15 | 1.10 | 0.98 |
|  | B | Females | 72.66 | 7.03 | 10.16 | 4.69 | 5.47 |
|  |  | Males | 75.63 | 7.56 | 9.24 | 5.04 | 2.52 |
|  |  | Non-binary gender | 85.71 | 0.00 | 0.00 | 14.29 | 0.00 |
|  |  | Total | 74.41 | 7.09 | 9.45 | 5.12 | 3.94 |
|  | C | Females | 77.43 | 6.61 | 12.06 | 1.95 | 1.95 |
|  |  | Males | 75.86 | 9.20 | 9.20 | 3.45 | 2.30 |
|  |  | Non-binary gender | 88.89 | 0.00 | 11.11 | 0.00 | 0.00 |
|  |  | Total | 77.34 | 7.08 | 11.33 | 2.27 | 1.98 |
| **Malaysia** | A | Females | 79.27 | 9.76 | 4.88 | 1.22 | 4.88 |
|  |  | Males | 69.23 | 23.08 | 0.00 | 7.69 | 0.00 |
|  |  | Non-binary gender | 100.00 | 0.00 | 0.00 | 0.00 | 0.00 |
|  |  | Total | 78.13 | 11.46 | 4.17 | 2.08 | 4.17 |
|  | B | Females | 46.88 | 18.75 | 9.38 | 25.00 | 0.00 |
|  |  | Males | 90.00 | 0.00 | 10.00 | 0.00 | 0.00 |
|  |  | Non-binary gender |  |  |  |  |  |
|  |  | Total | 57.14 | 14.29 | 9.52 | 19.05 | 0.00 |
|  | C | Females | 53.85 | 15.38 | 6.15 | 21.54 | 3.08 |
|  |  | Males | 57.14 | 7.14 | 14.29 | 14.29 | 7.14 |
|  |  | Non-binary gender | 100.00 | 0.00 | 0.00 | 0.00 | 0.00 |
|  |  | Total | 55.00 | 13.75 | 7.50 | 20.00 | 3.75 |
| **Hungary** | A | Females | 100.00 | 0.00 | 0.00 | 0.00 | 0.00 |
|  |  | Males | 100.00 | 0.00 | 0.00 | 0.00 | 0.00 |
|  |  | Non-binary gender |  |  |  |  |  |
|  |  | Total | 100.00 | 0.00 | 0.00 | 0.00 | 0.00 |
|  | B | Females | 80.00 | 10.00 | 10.00 | 0.00 | 0.00 |
|  |  | Males | 100.00 | 0.00 | 0.00 | 0.00 | 0.00 |
|  |  | Non-binary gender |  |  |  |  |  |
|  |  | Total | 85.71 | 7.14 | 7.14 | 0.00 | 0.00 |
|  | C | Females | 89.66 | 0.00 | 6.90 | 3.45 | 0.00 |
|  |  | Males | 66.67 | 0.00 | 33.33 | 0.00 | 0.00 |
|  |  | Non-binary gender |  |  |  |  |  |
|  |  | Total | 87.50 | 0.00 | 9.38 | 3.13 | 0.00 |
| **TOTAL** |  |  | 83.70 | 6.11 | 7.34 | 1.70 | 1.15 |
| **J5. Do you believe that COVID-19 appeared accidentally from human contact with animals and it was something that generally happens and was generally expected?** | | | | | | | |
| **Bulgaria** | A | Females | 51.06 | 17.02 | 21.28 | 10.64 | 0.00 |
|  |  | Males | 24.44 | 26.67 | 20.00 | 17.78 | 11.11 |
|  |  | Non-binary gender | 0.00 | 50.00 | 0.00 | 0.00 | 50.00 |
|  |  | Total | 37.23 | 22.34 | 20.21 | 13.83 | 6.38 |
|  | B | Females | 21.43 | 28.57 | 32.14 | 8.93 | 8.93 |
|  |  | Males | 36.67 | 23.33 | 30.00 | 6.67 | 3.33 |
|  |  | Non-binary gender |  |  |  |  |  |
|  |  | Total | 26.74 | 26.74 | 31.40 | 8.14 | 6.98 |
|  | C | Females | 40.68 | 18.64 | 33.90 | 3.39 | 3.39 |
|  |  | Males | 22.22 | 25.93 | 25.93 | 11.11 | 14.81 |
|  |  | Non-binary gender |  |  |  |  |  |
|  |  | Total | 34.88 | 20.93 | 31.40 | 5.81 | 6.98 |
| **Croatia** | A | Females | 17.75 | 16.67 | 27.06 | 19.26 | 19.26 |
|  |  | Males | 11.25 | 15.00 | 25.00 | 20.00 | 28.75 |
|  |  | Non-binary gender | 0.00 | 0.00 | 0.00 | 0.00 | 100.00 |
|  |  | Total | 16.76 | 16.39 | 26.70 | 19.34 | 20.81 |
|  | B | Females | 31.44 | 25.33 | 24.24 | 9.83 | 9.17 |
|  |  | Males | 26.95 | 19.76 | 27.54 | 14.97 | 10.78 |
|  |  | Non-binary gender | 50.00 | 0.00 | 16.67 | 16.67 | 16.67 |
|  |  | Total | 30.43 | 23.61 | 25.04 | 11.25 | 9.67 |
|  | C | Females | 27.54 | 18.86 | 29.64 | 16.77 | 7.19 |
|  |  | Males | 30.00 | 22.00 | 22.00 | 16.00 | 10.00 |
|  |  | Non-binary gender | 0.00 | 100.00 | 0.00 | 0.00 | 0.00 |
|  |  | Total | 27.72 | 19.69 | 28.50 | 16.58 | 7.51 |
| **Georgia** | A | Females | 23.46 | 25.14 | 27.93 | 13.69 | 9.78 |
|  |  | Males | 25.77 | 18.40 | 26.38 | 16.56 | 12.88 |
|  |  | Non-binary gender | 0.00 | 0.00 | 100.00 | 0.00 | 0.00 |
|  |  | Total | 24.09 | 22.94 | 27.72 | 14.53 | 10.71 |
|  | B | Females | 31.54 | 26.85 | 20.81 | 12.75 | 8.05 |
|  |  | Males | 31.65 | 29.11 | 24.05 | 5.06 | 10.13 |
|  |  | Non-binary gender | 33.33 | 0.00 | 33.33 | 0.00 | 33.33 |
|  |  | Total | 31.60 | 27.27 | 22.08 | 9.96 | 9.09 |
|  | C | Females | 35.60 | 23.56 | 24.61 | 9.95 | 6.28 |
|  |  | Males | 18.42 | 26.32 | 21.05 | 13.16 | 21.05 |
|  |  | Non-binary gender | 100.00 | 0.00 | 0.00 | 0.00 | 0.00 |
|  |  | Total | 33.04 | 23.91 | 23.91 | 10.43 | 8.70 |
| **Greece** | A | Females | 15.71 | 18.35 | 27.03 | 25.49 | 13.41 |
|  |  | Males | 12.86 | 14.29 | 20.36 | 28.57 | 23.93 |
|  |  | Non-binary gender | 30.77 | 15.38 | 7.69 | 7.69 | 38.46 |
|  |  | Total | 15.21 | 17.37 | 25.27 | 26.02 | 16.13 |
|  | B | Females | 20.92 | 22.43 | 28.56 | 18.14 | 9.94 |
|  |  | Males | 14.30 | 14.55 | 25.92 | 28.36 | 16.87 |
|  |  | Non-binary gender | 14.81 | 14.81 | 25.93 | 7.41 | 37.04 |
|  |  | Total | 18.27 | 19.27 | 27.50 | 21.98 | 12.99 |
|  | C | Females | 28.47 | 21.28 | 27.62 | 14.43 | 8.21 |
|  |  | Males | 17.18 | 19.15 | 27.89 | 24.51 | 11.27 |
|  |  | Non-binary gender | 6.90 | 6.90 | 51.72 | 17.24 | 17.24 |
|  |  | Total | 26.31 | 20.73 | 27.99 | 16.13 | 8.83 |
| **India** | A | Females | 18.87 | 24.53 | 41.51 | 11.32 | 3.77 |
|  |  | Males | 28.21 | 35.90 | 28.21 | 5.13 | 2.56 |
|  |  | Non-binary gender |  |  |  |  |  |
|  |  | Total | 21.38 | 27.59 | 37.93 | 9.66 | 3.45 |
|  | B | Females | 22.81 | 21.05 | 31.58 | 17.54 | 7.02 |
|  |  | Males | 23.77 | 23.77 | 34.43 | 8.20 | 9.84 |
|  |  | Non-binary gender |  |  |  |  |  |
|  |  | Total | 23.31 | 22.46 | 33.05 | 12.71 | 8.47 |
|  | C | Females | 27.59 | 19.54 | 31.03 | 9.20 | 12.64 |
|  |  | Males | 21.15 | 23.08 | 34.62 | 11.54 | 9.62 |
|  |  | Non-binary gender | 0.00 | 0.00 | 100.00 | 0.00 | 0.00 |
|  |  | Total | 25.00 | 20.71 | 32.86 | 10.00 | 11.43 |
| **Latvia** | A | Females | 10.85 | 11.14 | 33.72 | 28.15 | 16.13 |
|  |  | Males | 7.50 | 2.50 | 32.50 | 37.50 | 20.00 |
|  |  | Non-binary gender | 0.00 | 20.00 | 20.00 | 20.00 | 40.00 |
|  |  | Total | 10.36 | 10.36 | 33.42 | 29.02 | 16.84 |
|  | B | Females | 23.15 | 17.24 | 33.50 | 18.23 | 7.88 |
|  |  | Males | 26.02 | 17.07 | 27.64 | 16.26 | 13.01 |
|  |  | Non-binary gender | 50.00 | 0.00 | 50.00 | 0.00 | 0.00 |
|  |  | Total | 24.39 | 17.07 | 31.40 | 17.38 | 9.76 |
|  | C | Females | 21.48 | 17.96 | 37.68 | 16.20 | 6.69 |
|  |  | Males | 16.33 | 12.24 | 48.98 | 14.29 | 8.16 |
|  |  | Non-binary gender |  |  |  |  |  |
|  |  | Total | 20.72 | 17.12 | 39.34 | 15.92 | 6.91 |
| **Lithuania** | A | Females | 10.11 | 11.70 | 19.15 | 29.26 | 29.79 |
|  |  | Males | 8.82 | 5.88 | 23.53 | 35.29 | 26.47 |
|  |  | Non-binary gender | 0.00 | 33.33 | 33.33 | 33.33 | 0.00 |
|  |  | Total | 9.78 | 11.11 | 20.00 | 30.22 | 28.89 |
|  | B | Females | 27.27 | 18.18 | 12.12 | 27.27 | 15.15 |
|  |  | Males | 0.00 | 37.50 | 25.00 | 25.00 | 12.50 |
|  |  | Non-binary gender |  |  |  |  |  |
|  |  | Total | 21.95 | 21.95 | 14.63 | 26.83 | 14.63 |
|  | C | Females | 14.29 | 17.86 | 33.93 | 21.43 | 12.50 |
|  |  | Males | 15.38 | 23.08 | 38.46 | 23.08 | 0.00 |
|  |  | Non-binary gender |  |  |  |  |  |
|  |  | Total | 14.49 | 18.84 | 34.78 | 21.74 | 10.14 |
| **Nigeria** | A | Females | 51.47 | 25.00 | 16.18 | 5.88 | 1.47 |
|  |  | Males | 45.10 | 17.65 | 29.41 | 5.88 | 1.96 |
|  |  | Non-binary gender |  |  |  |  |  |
|  |  | Total | 48.74 | 21.85 | 21.85 | 5.88 | 1.68 |
|  | B | Females | 44.44 | 15.15 | 35.35 | 3.03 | 2.02 |
|  |  | Males | 51.52 | 15.15 | 28.79 | 3.79 | 0.76 |
|  |  | Non-binary gender |  |  |  |  |  |
|  |  | Total | 48.48 | 15.15 | 31.60 | 3.46 | 1.30 |
|  | C | Females | 57.89 | 22.11 | 17.89 | 0.00 | 2.11 |
|  |  | Males | 53.90 | 18.44 | 24.11 | 1.42 | 2.13 |
|  |  | Non-binary gender | 0.00 | 100.00 | 0.00 | 0.00 | 0.00 |
|  |  | Total | 55.27 | 20.25 | 21.52 | 0.84 | 2.11 |
| **Russia** | A | Females | 23.35 | 19.01 | 30.69 | 22.60 | 4.34 |
|  |  | Males | 15.67 | 21.64 | 34.33 | 24.63 | 3.73 |
|  |  | Non-binary gender | 37.50 | 25.00 | 31.25 | 0.00 | 6.25 |
|  |  | Total | 22.37 | 19.56 | 31.30 | 22.49 | 4.28 |
|  | B | Females | 17.97 | 21.88 | 38.28 | 17.97 | 3.91 |
|  |  | Males | 26.89 | 20.17 | 29.41 | 21.01 | 2.52 |
|  |  | Non-binary gender | 14.29 | 42.86 | 28.57 | 14.29 | 0.00 |
|  |  | Total | 22.05 | 21.65 | 33.86 | 19.29 | 3.15 |
|  | C | Females | 22.18 | 17.51 | 33.07 | 20.23 | 7.00 |
|  |  | Males | 25.29 | 14.94 | 32.18 | 19.54 | 8.05 |
|  |  | Non-binary gender | 33.33 | 0.00 | 55.56 | 11.11 | 0.00 |
|  |  | Total | 23.23 | 16.43 | 33.43 | 19.83 | 7.08 |
| **Malaysia** | A | Females | 30.49 | 17.07 | 13.41 | 25.61 | 13.41 |
|  |  | Males | 23.08 | 30.77 | 0.00 | 38.46 | 7.69 |
|  |  | Non-binary gender | 0.00 | 0.00 | 100.00 | 0.00 | 0.00 |
|  |  | Total | 29.17 | 18.75 | 12.50 | 27.08 | 12.50 |
|  | B | Females | 15.63 | 15.63 | 21.88 | 43.75 | 3.13 |
|  |  | Males | 50.00 | 0.00 | 10.00 | 30.00 | 10.00 |
|  |  | Non-binary gender |  |  |  |  |  |
|  |  | Total | 23.81 | 11.90 | 19.05 | 40.48 | 4.76 |
|  | C | Females | 24.62 | 18.46 | 15.38 | 27.69 | 13.85 |
|  |  | Males | 14.29 | 35.71 | 28.57 | 21.43 | 0.00 |
|  |  | Non-binary gender | 100.00 | 0.00 | 0.00 | 0.00 | 0.00 |
|  |  | Total | 23.75 | 21.25 | 17.50 | 26.25 | 11.25 |
| **Hungary** | A | Females | 12.50 | 9.38 | 15.63 | 21.88 | 40.63 |
|  |  | Males | 0.00 | 11.11 | 0.00 | 55.56 | 33.33 |
|  |  | Non-binary gender |  |  |  |  |  |
|  |  | Total | 9.76 | 9.76 | 12.20 | 29.27 | 39.02 |
|  | B | Females | 40.00 | 10.00 | 30.00 | 10.00 | 10.00 |
|  |  | Males | 0.00 | 0.00 | 25.00 | 50.00 | 25.00 |
|  |  | Non-binary gender |  |  |  |  |  |
|  |  | Total | 28.57 | 7.14 | 28.57 | 21.43 | 14.29 |
|  | C | Females | 13.79 | 24.14 | 24.14 | 13.79 | 24.14 |
|  |  | Males | 0.00 | 0.00 | 33.33 | 66.67 | 0.00 |
|  |  | Non-binary gender |  |  |  |  |  |
|  |  | Total | 12.50 | 21.88 | 25.00 | 18.75 | 21.88 |
| **TOTAL** |  |  | 23.45 | 19.46 | 28.02 | 18.33 | 10.73 |
| **J6. Do you believe that COVID-19 has much lower mortality rate but there is misinformation and terror-inducing propaganda?** | | | | | | | |
| **Bulgaria** | A | Females | 38.30 | 12.77 | 27.66 | 10.64 | 10.64 |
|  |  | Males | 42.22 | 15.56 | 22.22 | 15.56 | 4.44 |
|  |  | Non-binary gender | 50.00 | 50.00 | 0.00 | 0.00 | 0.00 |
|  |  | Total | 40.43 | 14.89 | 24.47 | 12.77 | 7.45 |
|  | B | Females | 7.14 | 10.71 | 26.79 | 21.43 | 33.93 |
|  |  | Males | 13.33 | 23.33 | 6.67 | 26.67 | 30.00 |
|  |  | Non-binary gender |  |  |  |  |  |
|  |  | Total | 9.30 | 15.12 | 19.77 | 23.26 | 32.56 |
|  | C | Females | 11.86 | 10.17 | 20.34 | 20.34 | 37.29 |
|  |  | Males | 18.52 | 29.63 | 29.63 | 11.11 | 11.11 |
|  |  | Non-binary gender |  |  |  |  |  |
|  |  | Total | 13.95 | 16.28 | 23.26 | 17.44 | 29.07 |
| **Croatia** | A | Females | 28.14 | 16.02 | 20.13 | 19.05 | 16.67 |
|  |  | Males | 30.00 | 25.00 | 17.50 | 11.25 | 16.25 |
|  |  | Non-binary gender | 100.00 | 0.00 | 0.00 | 0.00 | 0.00 |
|  |  | Total | 28.55 | 17.31 | 19.71 | 17.86 | 16.57 |
|  | B | Females | 14.41 | 16.81 | 20.52 | 18.12 | 30.13 |
|  |  | Males | 20.96 | 14.37 | 18.56 | 14.37 | 31.74 |
|  |  | Non-binary gender | 0.00 | 50.00 | 0.00 | 16.67 | 33.33 |
|  |  | Total | 16.01 | 16.48 | 19.81 | 17.12 | 30.59 |
|  | C | Females | 22.75 | 17.96 | 22.75 | 15.27 | 21.26 |
|  |  | Males | 14.00 | 14.00 | 22.00 | 24.00 | 26.00 |
|  |  | Non-binary gender | 50.00 | 50.00 | 0.00 | 0.00 | 0.00 |
|  |  | Total | 21.76 | 17.62 | 22.54 | 16.32 | 21.76 |
| **Georgia** | A | Females | 39.39 | 21.79 | 18.99 | 12.29 | 7.54 |
|  |  | Males | 36.20 | 23.31 | 19.63 | 7.98 | 12.88 |
|  |  | Non-binary gender | 100.00 | 0.00 | 0.00 | 0.00 | 0.00 |
|  |  | Total | 38.62 | 22.18 | 19.12 | 10.90 | 9.18 |
|  | B | Females | 28.86 | 21.48 | 19.46 | 13.42 | 16.78 |
|  |  | Males | 35.44 | 22.78 | 21.52 | 13.92 | 6.33 |
|  |  | Non-binary gender | 0.00 | 0.00 | 100.00 | 0.00 | 0.00 |
|  |  | Total | 30.74 | 21.65 | 21.21 | 13.42 | 12.99 |
|  | C | Females | 31.41 | 23.56 | 18.85 | 13.61 | 12.57 |
|  |  | Males | 28.95 | 10.53 | 21.05 | 28.95 | 10.53 |
|  |  | Non-binary gender | 0.00 | 0.00 | 0.00 | 0.00 | 100.00 |
|  |  | Total | 30.87 | 21.30 | 19.13 | 16.09 | 12.61 |
| **Greece** | A | Females | 24.07 | 25.49 | 18.68 | 16.26 | 15.49 |
|  |  | Males | 24.29 | 23.57 | 19.29 | 15.71 | 17.14 |
|  |  | Non-binary gender | 7.69 | 30.77 | 38.46 | 23.08 | 0.00 |
|  |  | Total | 23.94 | 25.10 | 19.04 | 16.21 | 15.71 |
|  | B | Females | 22.28 | 24.74 | 20.29 | 18.46 | 14.24 |
|  |  | Males | 23.11 | 25.79 | 18.22 | 17.24 | 15.65 |
|  |  | Non-binary gender | 22.22 | 22.22 | 29.63 | 3.70 | 22.22 |
|  |  | Total | 22.60 | 25.12 | 19.60 | 17.79 | 14.89 |
|  | C | Females | 20.94 | 21.39 | 19.98 | 19.24 | 18.45 |
|  |  | Males | 23.66 | 24.23 | 16.90 | 17.75 | 17.46 |
|  |  | Non-binary gender | 24.14 | 24.14 | 24.14 | 10.34 | 17.24 |
|  |  | Total | 21.43 | 21.90 | 19.53 | 18.87 | 18.27 |
| **India** | A | Females | 25.47 | 28.30 | 34.91 | 7.55 | 3.77 |
|  |  | Males | 23.08 | 28.21 | 38.46 | 5.13 | 5.13 |
|  |  | Non-binary gender |  |  |  |  |  |
|  |  | Total | 24.83 | 28.28 | 35.86 | 6.90 | 4.14 |
|  | B | Females | 22.81 | 23.68 | 28.95 | 14.04 | 10.53 |
|  |  | Males | 33.61 | 13.11 | 31.97 | 9.84 | 11.48 |
|  |  | Non-binary gender |  |  |  |  |  |
|  |  | Total | 28.39 | 18.22 | 30.51 | 11.86 | 11.02 |
|  | C | Females | 31.03 | 10.34 | 37.93 | 10.34 | 10.34 |
|  |  | Males | 19.23 | 26.92 | 32.69 | 15.38 | 5.77 |
|  |  | Non-binary gender | 100.00 | 0.00 | 0.00 | 0.00 | 0.00 |
|  |  | Total | 27.14 | 16.43 | 35.71 | 12.14 | 8.57 |
| **Latvia** | A | Females | 48.09 | 20.23 | 19.06 | 7.33 | 5.28 |
|  |  | Males | 60.00 | 5.00 | 7.50 | 12.50 | 15.00 |
|  |  | Non-binary gender | 100.00 | 0.00 | 0.00 | 0.00 | 0.00 |
|  |  | Total | 50.00 | 18.39 | 17.62 | 7.77 | 6.22 |
|  | B | Females | 30.05 | 16.75 | 25.62 | 15.27 | 12.32 |
|  |  | Males | 34.96 | 18.70 | 22.76 | 15.45 | 8.13 |
|  |  | Non-binary gender | 0.00 | 50.00 | 0.00 | 0.00 | 50.00 |
|  |  | Total | 31.71 | 17.68 | 24.39 | 15.24 | 10.98 |
|  | C | Females | 34.86 | 14.44 | 21.83 | 15.85 | 13.03 |
|  |  | Males | 30.61 | 16.33 | 20.41 | 8.16 | 24.49 |
|  |  | Non-binary gender |  |  |  |  |  |
|  |  | Total | 34.23 | 14.71 | 21.62 | 14.71 | 14.71 |
| **Lithuania** | A | Females | 69.68 | 11.17 | 10.11 | 3.72 | 5.32 |
|  |  | Males | 91.18 | 5.88 | 0.00 | 0.00 | 2.94 |
|  |  | Non-binary gender | 100.00 | 0.00 | 0.00 | 0.00 | 0.00 |
|  |  | Total | 73.33 | 10.22 | 8.44 | 3.11 | 4.89 |
|  | B | Females | 54.55 | 12.12 | 12.12 | 9.09 | 12.12 |
|  |  | Males | 12.50 | 0.00 | 50.00 | 25.00 | 12.50 |
|  |  | Non-binary gender |  |  |  |  |  |
|  |  | Total | 46.34 | 9.76 | 19.51 | 12.20 | 12.20 |
|  | C | Females | 42.86 | 25.00 | 8.93 | 8.93 | 14.29 |
|  |  | Males | 61.54 | 7.69 | 15.38 | 0.00 | 15.38 |
|  |  | Non-binary gender |  |  |  |  |  |
|  |  | Total | 46.38 | 21.74 | 10.14 | 7.25 | 14.49 |
| **Nigeria** | A | Females | 44.12 | 25.00 | 26.47 | 2.94 | 1.47 |
|  |  | Males | 41.18 | 23.53 | 25.49 | 5.88 | 3.92 |
|  |  | Non-binary gender |  |  |  |  |  |
|  |  | Total | 42.86 | 24.37 | 26.05 | 4.20 | 2.52 |
|  | B | Females | 47.47 | 18.18 | 29.29 | 3.03 | 2.02 |
|  |  | Males | 53.03 | 15.15 | 27.27 | 2.27 | 2.27 |
|  |  | Non-binary gender |  |  |  |  |  |
|  |  | Total | 50.65 | 16.45 | 28.14 | 2.60 | 2.16 |
|  | C | Females | 50.53 | 22.11 | 24.21 | 1.05 | 2.11 |
|  |  | Males | 50.35 | 18.44 | 27.66 | 0.71 | 2.84 |
|  |  | Non-binary gender | 0.00 | 0.00 | 100.00 | 0.00 | 0.00 |
|  |  | Total | 50.21 | 19.83 | 26.58 | 0.84 | 2.53 |
| **Russia** | A | Females | 49.25 | 14.22 | 22.31 | 10.03 | 4.19 |
|  |  | Males | 51.49 | 15.67 | 20.90 | 6.72 | 5.22 |
|  |  | Non-binary gender | 56.25 | 12.50 | 18.75 | 6.25 | 6.25 |
|  |  | Total | 49.76 | 14.43 | 22.00 | 9.41 | 4.40 |
|  | B | Females | 30.47 | 14.84 | 32.03 | 15.63 | 7.03 |
|  |  | Males | 36.13 | 19.33 | 28.57 | 10.92 | 5.04 |
|  |  | Non-binary gender | 42.86 | 14.29 | 42.86 | 0.00 | 0.00 |
|  |  | Total | 33.46 | 16.93 | 30.71 | 12.99 | 5.91 |
|  | C | Females | 38.13 | 16.73 | 22.57 | 14.79 | 7.78 |
|  |  | Males | 50.57 | 11.49 | 22.99 | 11.49 | 3.45 |
|  |  | Non-binary gender | 55.56 | 11.11 | 33.33 | 0.00 | 0.00 |
|  |  | Total | 41.64 | 15.30 | 22.95 | 13.60 | 6.52 |
| **Malaysia** | A | Females | 65.85 | 14.63 | 3.66 | 9.76 | 6.10 |
|  |  | Males | 46.15 | 23.08 | 7.69 | 15.38 | 7.69 |
|  |  | Non-binary gender | 0.00 | 0.00 | 100.00 | 0.00 | 0.00 |
|  |  | Total | 62.50 | 15.63 | 5.21 | 10.42 | 6.25 |
|  | B | Females | 21.88 | 37.50 | 18.75 | 15.63 | 6.25 |
|  |  | Males | 50.00 | 10.00 | 10.00 | 20.00 | 10.00 |
|  |  | Non-binary gender |  |  |  |  |  |
|  |  | Total | 28.57 | 30.95 | 16.67 | 16.67 | 7.14 |
|  | C | Females | 46.15 | 12.31 | 13.85 | 21.54 | 6.15 |
|  |  | Males | 14.29 | 7.14 | 21.43 | 50.00 | 7.14 |
|  |  | Non-binary gender | 100.00 | 0.00 | 0.00 | 0.00 | 0.00 |
|  |  | Total | 41.25 | 11.25 | 15.00 | 26.25 | 6.25 |
| **Hungary** | A | Females | 62.50 | 9.38 | 12.50 | 0.00 | 15.63 |
|  |  | Males | 66.67 | 11.11 | 11.11 | 0.00 | 11.11 |
|  |  | Non-binary gender |  |  |  |  |  |
|  |  | Total | 63.41 | 9.76 | 12.20 | 0.00 | 14.63 |
|  | B | Females | 50.00 | 0.00 | 20.00 | 20.00 | 10.00 |
|  |  | Males | 25.00 | 0.00 | 50.00 | 25.00 | 0.00 |
|  |  | Non-binary gender |  |  |  |  |  |
|  |  | Total | 42.86 | 0.00 | 28.57 | 21.43 | 7.14 |
|  | C | Females | 55.17 | 6.90 | 17.24 | 13.79 | 6.90 |
|  |  | Males | 66.67 | 0.00 | 0.00 | 0.00 | 33.33 |
|  |  | Non-binary gender |  |  |  |  |  |
|  |  | Total | 56.25 | 6.25 | 15.63 | 12.50 | 9.38 |
| **TOTAL** |  |  | 30.55 | 20.20 | 20.80 | 14.67 | 13.78 |
| **J7. Do you think the recommended measures (e.g. wearing face masks, avoid gatherings, stay at home etc.) are an attempt to restrict human rights and lead to some kind of dictatorship rather than to keep the population safer from COVID-19?** | | | | | | | |
| **Bulgaria** | A | Females | 63.83 | 12.77 | 10.64 | 6.38 | 6.38 |
|  |  | Males | 77.78 | 13.33 | 4.44 | 2.22 | 2.22 |
|  |  | Non-binary gender | 100.00 | 0.00 | 0.00 | 0.00 | 0.00 |
|  |  | Total | 71.28 | 12.77 | 7.45 | 4.26 | 4.26 |
|  | B | Females | 26.79 | 23.21 | 14.29 | 17.86 | 17.86 |
|  |  | Males | 30.00 | 6.67 | 36.67 | 16.67 | 10.00 |
|  |  | Non-binary gender |  |  |  |  |  |
|  |  | Total | 27.91 | 17.44 | 22.09 | 17.44 | 15.12 |
|  | C | Females | 20.34 | 13.56 | 27.12 | 20.34 | 18.64 |
|  |  | Males | 40.74 | 18.52 | 25.93 | 7.41 | 7.41 |
|  |  | Non-binary gender |  |  |  |  |  |
|  |  | Total | 26.74 | 15.12 | 26.74 | 16.28 | 15.12 |
| **Croatia** | A | Females | 46.97 | 19.05 | 15.15 | 9.74 | 9.09 |
|  |  | Males | 52.50 | 18.75 | 8.75 | 11.25 | 8.75 |
|  |  | Non-binary gender | 100.00 | 0.00 | 0.00 | 0.00 | 0.00 |
|  |  | Total | 47.88 | 18.97 | 14.18 | 9.94 | 9.02 |
|  | B | Females | 31.88 | 15.07 | 19.21 | 13.76 | 20.09 |
|  |  | Males | 38.32 | 14.37 | 15.57 | 8.98 | 22.75 |
|  |  | Non-binary gender | 50.00 | 16.67 | 16.67 | 16.67 | 0.00 |
|  |  | Total | 33.76 | 14.90 | 18.23 | 12.52 | 20.60 |
|  | C | Females | 38.92 | 17.37 | 14.37 | 12.87 | 16.47 |
|  |  | Males | 30.00 | 20.00 | 16.00 | 12.00 | 22.00 |
|  |  | Non-binary gender | 50.00 | 50.00 | 0.00 | 0.00 | 0.00 |
|  |  | Total | 37.82 | 17.88 | 14.51 | 12.69 | 17.10 |
| **Georgia** | A | Females | 70.67 | 12.85 | 7.82 | 3.35 | 5.31 |
|  |  | Males | 62.58 | 13.50 | 12.88 | 6.13 | 4.91 |
|  |  | Non-binary gender | 100.00 | 0.00 | 0.00 | 0.00 | 0.00 |
|  |  | Total | 68.26 | 13.00 | 9.37 | 4.21 | 5.16 |
|  | B | Females | 60.40 | 19.46 | 10.07 | 3.36 | 6.71 |
|  |  | Males | 49.37 | 24.05 | 12.66 | 8.86 | 5.06 |
|  |  | Non-binary gender | 33.33 | 33.33 | 0.00 | 0.00 | 33.33 |
|  |  | Total | 56.28 | 21.21 | 10.82 | 5.19 | 6.49 |
|  | C | Females | 57.07 | 17.80 | 10.47 | 7.85 | 6.81 |
|  |  | Males | 52.63 | 28.95 | 5.26 | 5.26 | 7.89 |
|  |  | Non-binary gender | 0.00 | 0.00 | 0.00 | 0.00 | 100.00 |
|  |  | Total | 56.09 | 19.57 | 9.57 | 7.39 | 7.39 |
| **Greece** | A | Females | 51.43 | 20.77 | 13.08 | 7.91 | 6.81 |
|  |  | Males | 53.57 | 18.93 | 11.79 | 8.93 | 6.79 |
|  |  | Non-binary gender | 30.77 | 46.15 | 7.69 | 0.00 | 15.38 |
|  |  | Total | 51.70 | 20.62 | 12.72 | 8.06 | 6.90 |
|  | B | Females | 41.69 | 24.98 | 15.91 | 9.71 | 7.72 |
|  |  | Males | 49.76 | 22.49 | 13.57 | 6.85 | 7.33 |
|  |  | Non-binary gender | 29.63 | 14.81 | 22.22 | 22.22 | 11.11 |
|  |  | Total | 44.67 | 23.88 | 15.08 | 8.75 | 7.61 |
|  | C | Females | 41.26 | 20.60 | 16.53 | 12.28 | 9.34 |
|  |  | Males | 44.79 | 21.97 | 17.46 | 7.61 | 8.17 |
|  |  | Non-binary gender | 27.59 | 20.69 | 10.34 | 13.79 | 27.59 |
|  |  | Total | 41.66 | 20.83 | 16.60 | 11.53 | 9.39 |
| **India** | A | Females | 38.68 | 19.81 | 24.53 | 9.43 | 7.55 |
|  |  | Males | 41.03 | 17.95 | 23.08 | 7.69 | 10.26 |
|  |  | Non-binary gender |  |  |  |  |  |
|  |  | Total | 39.31 | 19.31 | 24.14 | 8.97 | 8.28 |
|  | B | Females | 57.02 | 12.28 | 18.42 | 5.26 | 7.02 |
|  |  | Males | 67.21 | 9.84 | 15.57 | 4.10 | 3.28 |
|  |  | Non-binary gender |  |  |  |  |  |
|  |  | Total | 62.29 | 11.02 | 16.95 | 4.66 | 5.08 |
|  | C | Females | 65.52 | 11.49 | 12.64 | 2.30 | 8.05 |
|  |  | Males | 63.46 | 9.62 | 11.54 | 3.85 | 11.54 |
|  |  | Non-binary gender | 0.00 | 100.00 | 0.00 | 0.00 | 0.00 |
|  |  | Total | 64.29 | 11.43 | 12.14 | 2.86 | 9.29 |
| **Latvia** | A | Females | 77.71 | 9.38 | 8.80 | 2.93 | 1.17 |
|  |  | Males | 85.00 | 7.50 | 7.50 | 0.00 | 0.00 |
|  |  | Non-binary gender | 100.00 | 0.00 | 0.00 | 0.00 | 0.00 |
|  |  | Total | 78.76 | 9.07 | 8.55 | 2.59 | 1.04 |
|  | B | Females | 62.56 | 10.84 | 15.27 | 5.91 | 5.42 |
|  |  | Males | 69.11 | 8.13 | 13.01 | 6.50 | 3.25 |
|  |  | Non-binary gender | 0.00 | 50.00 | 0.00 | 50.00 | 0.00 |
|  |  | Total | 64.63 | 10.06 | 14.33 | 6.40 | 4.57 |
|  | C | Females | 69.72 | 7.75 | 11.62 | 7.75 | 3.17 |
|  |  | Males | 65.31 | 12.24 | 10.20 | 4.08 | 8.16 |
|  |  | Non-binary gender |  |  |  |  |  |
|  |  | Total | 69.07 | 8.41 | 11.41 | 7.21 | 3.90 |
| **Lithuania** | A | Females | 82.98 | 9.57 | 1.06 | 3.72 | 2.66 |
|  |  | Males | 88.24 | 5.88 | 2.94 | 0.00 | 2.94 |
|  |  | Non-binary gender | 100.00 | 0.00 | 0.00 | 0.00 | 0.00 |
|  |  | Total | 84.00 | 8.89 | 1.33 | 3.11 | 2.67 |
|  | B | Females | 60.61 | 9.09 | 12.12 | 6.06 | 12.12 |
|  |  | Males | 50.00 | 0.00 | 37.50 | 0.00 | 12.50 |
|  |  | Non-binary gender |  |  |  |  |  |
|  |  | Total | 58.54 | 7.32 | 17.07 | 4.88 | 12.20 |
|  | C | Females | 75.00 | 7.14 | 7.14 | 7.14 | 3.57 |
|  |  | Males | 76.92 | 0.00 | 15.38 | 0.00 | 7.69 |
|  |  | Non-binary gender |  |  |  |  |  |
|  |  | Total | 75.36 | 5.80 | 8.70 | 5.80 | 4.35 |
| **Nigeria** | A | Females | 58.82 | 17.65 | 19.12 | 2.94 | 1.47 |
|  |  | Males | 58.82 | 17.65 | 17.65 | 3.92 | 1.96 |
|  |  | Non-binary gender |  |  |  |  |  |
|  |  | Total | 58.82 | 17.65 | 18.49 | 3.36 | 1.68 |
|  | B | Females | 43.43 | 20.20 | 32.32 | 2.02 | 2.02 |
|  |  | Males | 50.76 | 15.91 | 27.27 | 3.79 | 2.27 |
|  |  | Non-binary gender |  |  |  |  |  |
|  |  | Total | 47.62 | 17.75 | 29.44 | 3.03 | 2.16 |
|  | C | Females | 60.00 | 13.68 | 22.11 | 2.11 | 2.11 |
|  |  | Males | 52.48 | 19.86 | 24.82 | 0.71 | 2.13 |
|  |  | Non-binary gender | 0.00 | 0.00 | 0.00 | 100.00 | 0.00 |
|  |  | Total | 55.27 | 17.30 | 23.63 | 1.69 | 2.11 |
| **Russia** | A | Females | 64.82 | 13.62 | 12.57 | 6.14 | 2.84 |
|  |  | Males | 54.48 | 17.91 | 23.13 | 2.99 | 1.49 |
|  |  | Non-binary gender | 50.00 | 25.00 | 18.75 | 6.25 | 0.00 |
|  |  | Total | 62.84 | 14.55 | 14.43 | 5.62 | 2.57 |
|  | B | Females | 46.88 | 21.09 | 15.63 | 7.81 | 8.59 |
|  |  | Males | 47.90 | 19.33 | 21.85 | 8.40 | 2.52 |
|  |  | Non-binary gender | 42.86 | 28.57 | 28.57 | 0.00 | 0.00 |
|  |  | Total | 47.24 | 20.47 | 18.90 | 7.87 | 5.51 |
|  | C | Females | 47.86 | 22.18 | 17.12 | 8.95 | 3.89 |
|  |  | Males | 55.17 | 19.54 | 12.64 | 8.05 | 4.60 |
|  |  | Non-binary gender | 55.56 | 22.22 | 11.11 | 0.00 | 11.11 |
|  |  | Total | 49.86 | 21.53 | 15.86 | 8.50 | 4.25 |
| **Malaysia** | A | Females | 90.24 | 4.88 | 1.22 | 2.44 | 1.22 |
|  |  | Males | 46.15 | 15.38 | 0.00 | 15.38 | 23.08 |
|  |  | Non-binary gender | 100.00 | 0.00 | 0.00 | 0.00 | 0.00 |
|  |  | Total | 84.38 | 6.25 | 1.04 | 4.17 | 4.17 |
|  | B | Females | 50.00 | 21.88 | 9.38 | 18.75 | 0.00 |
|  |  | Males | 80.00 | 20.00 | 0.00 | 0.00 | 0.00 |
|  |  | Non-binary gender |  |  |  |  |  |
|  |  | Total | 57.14 | 21.43 | 7.14 | 14.29 | 0.00 |
|  | C | Females | 67.69 | 12.31 | 4.62 | 12.31 | 3.08 |
|  |  | Males | 35.71 | 21.43 | 7.14 | 28.57 | 7.14 |
|  |  | Non-binary gender | 100.00 | 0.00 | 0.00 | 0.00 | 0.00 |
|  |  | Total | 62.50 | 13.75 | 5.00 | 15.00 | 3.75 |
| **Hungary** | A | Females | 84.38 | 3.13 | 0.00 | 6.25 | 6.25 |
|  |  | Males | 88.89 | 11.11 | 0.00 | 0.00 | 0.00 |
|  |  | Non-binary gender |  |  |  |  |  |
|  |  | Total | 85.37 | 4.88 | 0.00 | 4.88 | 4.88 |
|  | B | Females | 40.00 | 20.00 | 20.00 | 10.00 | 10.00 |
|  |  | Males | 100.00 | 0.00 | 0.00 | 0.00 | 0.00 |
|  |  | Non-binary gender |  |  |  |  |  |
|  |  | Total | 57.14 | 14.29 | 14.29 | 7.14 | 7.14 |
|  | C | Females | 65.52 | 13.79 | 6.90 | 6.90 | 6.90 |
|  |  | Males | 66.67 | 0.00 | 0.00 | 33.33 | 0.00 |
|  |  | Non-binary gender |  |  |  |  |  |
|  |  | Total | 65.63 | 12.50 | 6.25 | 9.38 | 6.25 |
| **TOTAL** |  |  | 51.67 | 17.96 | 14.62 | 8.25 | 7.50 |
| **J8. Do you believe that COVID-19 outbreak is a deliberate creation of the world’s powerful leaders to create a global economic crisis?** | | | | | | | |
| **Bulgaria** | A | Females | 53.19 | 10.64 | 25.53 | 4.26 | 6.38 |
|  |  | Males | 71.11 | 13.33 | 8.89 | 2.22 | 4.44 |
|  |  | Non-binary gender | 100.00 | 0.00 | 0.00 | 0.00 | 0.00 |
|  |  | Total | 62.77 | 11.70 | 17.02 | 3.19 | 5.32 |
|  | B | Females | 21.43 | 17.86 | 21.43 | 14.29 | 25.00 |
|  |  | Males | 43.33 | 10.00 | 23.33 | 10.00 | 13.33 |
|  |  | Non-binary gender |  |  |  |  |  |
|  |  | Total | 29.07 | 15.12 | 22.09 | 12.79 | 20.93 |
|  | C | Females | 15.25 | 10.17 | 37.29 | 18.64 | 18.64 |
|  |  | Males | 48.15 | 7.41 | 25.93 | 11.11 | 7.41 |
|  |  | Non-binary gender |  |  |  |  |  |
|  |  | Total | 25.58 | 9.30 | 33.72 | 16.28 | 15.12 |
| **Croatia** | A | Females | 55.19 | 12.34 | 12.77 | 10.82 | 8.87 |
|  |  | Males | 62.50 | 16.25 | 7.50 | 6.25 | 7.50 |
|  |  | Non-binary gender | 100.00 | 0.00 | 0.00 | 0.00 | 0.00 |
|  |  | Total | 56.35 | 12.89 | 11.97 | 10.13 | 8.66 |
|  | B | Females | 31.88 | 13.32 | 25.11 | 12.45 | 17.25 |
|  |  | Males | 40.12 | 15.57 | 22.16 | 7.78 | 14.37 |
|  |  | Non-binary gender | 50.00 | 0.00 | 16.67 | 16.67 | 16.67 |
|  |  | Total | 34.23 | 13.79 | 24.25 | 11.25 | 16.48 |
|  | C | Females | 38.32 | 17.07 | 20.36 | 10.18 | 14.07 |
|  |  | Males | 36.00 | 18.00 | 20.00 | 12.00 | 14.00 |
|  |  | Non-binary gender | 0.00 | 100.00 | 0.00 | 0.00 | 0.00 |
|  |  | Total | 37.82 | 17.62 | 20.21 | 10.36 | 13.99 |
| **Georgia** | A | Females | 42.18 | 21.79 | 21.51 | 6.70 | 7.82 |
|  |  | Males | 42.33 | 21.47 | 17.79 | 6.75 | 11.66 |
|  |  | Non-binary gender | 50.00 | 50.00 | 0.00 | 0.00 | 0.00 |
|  |  | Total | 42.26 | 21.80 | 20.27 | 6.69 | 8.99 |
|  | B | Females | 42.95 | 17.45 | 20.81 | 7.38 | 11.41 |
|  |  | Males | 48.10 | 25.32 | 13.92 | 6.33 | 6.33 |
|  |  | Non-binary gender | 33.33 | 0.00 | 33.33 | 33.33 | 0.00 |
|  |  | Total | 44.59 | 19.91 | 18.61 | 7.36 | 9.52 |
|  | C | Females | 40.84 | 20.94 | 21.47 | 8.90 | 7.85 |
|  |  | Males | 47.37 | 23.68 | 13.16 | 10.53 | 5.26 |
|  |  | Non-binary gender | 0.00 | 0.00 | 0.00 | 0.00 | 100.00 |
|  |  | Total | 41.74 | 21.30 | 20.00 | 9.13 | 7.83 |
| **Greece** | A | Females | 58.57 | 16.70 | 14.84 | 6.26 | 3.63 |
|  |  | Males | 67.14 | 12.14 | 11.79 | 5.36 | 3.57 |
|  |  | Non-binary gender | 53.85 | 23.08 | 7.69 | 7.69 | 7.69 |
|  |  | Total | 60.52 | 15.71 | 14.05 | 6.07 | 3.66 |
|  | B | Females | 54.97 | 15.91 | 15.91 | 7.24 | 5.97 |
|  |  | Males | 65.89 | 16.50 | 8.68 | 5.38 | 3.55 |
|  |  | Non-binary gender | 51.85 | 22.22 | 14.81 | 3.70 | 7.41 |
|  |  | Total | 59.18 | 16.22 | 13.08 | 6.47 | 5.04 |
|  | C | Females | 46.18 | 16.30 | 20.43 | 9.56 | 7.53 |
|  |  | Males | 55.21 | 19.44 | 14.37 | 5.35 | 5.63 |
|  |  | Non-binary gender | 62.07 | 13.79 | 13.79 | 10.34 | 0.00 |
|  |  | Total | 47.88 | 16.78 | 19.34 | 8.88 | 7.11 |
| **India** | A | Females | 33.96 | 19.81 | 34.91 | 7.55 | 3.77 |
|  |  | Males | 30.77 | 25.64 | 28.21 | 7.69 | 7.69 |
|  |  | Non-binary gender |  |  |  |  |  |
|  |  | Total | 33.10 | 21.38 | 33.10 | 7.59 | 4.83 |
|  | B | Females | 35.96 | 18.42 | 28.07 | 10.53 | 7.02 |
|  |  | Males | 40.98 | 14.75 | 29.51 | 7.38 | 7.38 |
|  |  | Non-binary gender |  |  |  |  |  |
|  |  | Total | 38.56 | 16.53 | 28.81 | 8.90 | 7.20 |
|  | C | Females | 48.28 | 10.34 | 27.59 | 8.05 | 5.75 |
|  |  | Males | 34.62 | 21.15 | 28.85 | 7.69 | 7.69 |
|  |  | Non-binary gender | 0.00 | 0.00 | 100.00 | 0.00 | 0.00 |
|  |  | Total | 42.86 | 14.29 | 28.57 | 7.86 | 6.43 |
| **Latvia** | A | Females | 70.09 | 11.73 | 13.20 | 3.23 | 1.76 |
|  |  | Males | 77.50 | 5.00 | 15.00 | 0.00 | 2.50 |
|  |  | Non-binary gender | 100.00 | 0.00 | 0.00 | 0.00 | 0.00 |
|  |  | Total | 71.24 | 10.88 | 13.21 | 2.85 | 1.81 |
|  | B | Females | 57.64 | 14.29 | 17.73 | 2.46 | 7.88 |
|  |  | Males | 59.35 | 7.32 | 26.83 | 4.07 | 2.44 |
|  |  | Non-binary gender | 0.00 | 50.00 | 50.00 | 0.00 | 0.00 |
|  |  | Total | 57.93 | 11.89 | 21.34 | 3.05 | 5.79 |
|  | C | Females | 57.04 | 14.08 | 17.61 | 5.63 | 5.63 |
|  |  | Males | 67.35 | 12.24 | 2.04 | 6.12 | 12.24 |
|  |  | Non-binary gender |  |  |  |  |  |
|  |  | Total | 58.56 | 13.81 | 15.32 | 5.71 | 6.61 |
| **Lithuania** | A | Females | 82.98 | 7.45 | 6.91 | 1.60 | 1.06 |
|  |  | Males | 91.18 | 2.94 | 2.94 | 0.00 | 2.94 |
|  |  | Non-binary gender | 66.67 | 0.00 | 33.33 | 0.00 | 0.00 |
|  |  | Total | 84.00 | 6.67 | 6.67 | 1.33 | 1.33 |
|  | B | Females | 63.64 | 6.06 | 15.15 | 9.09 | 6.06 |
|  |  | Males | 37.50 | 25.00 | 25.00 | 12.50 | 0.00 |
|  |  | Non-binary gender |  |  |  |  |  |
|  |  | Total | 58.54 | 9.76 | 17.07 | 9.76 | 4.88 |
|  | C | Females | 71.43 | 12.50 | 12.50 | 1.79 | 1.79 |
|  |  | Males | 69.23 | 7.69 | 7.69 | 7.69 | 7.69 |
|  |  | Non-binary gender |  |  |  |  |  |
|  |  | Total | 71.01 | 11.59 | 11.59 | 2.90 | 2.90 |
| **Nigeria** | A | Females | 42.65 | 16.18 | 36.76 | 4.41 | 0.00 |
|  |  | Males | 45.10 | 23.53 | 27.45 | 1.96 | 1.96 |
|  |  | Non-binary gender |  |  |  |  |  |
|  |  | Total | 43.70 | 19.33 | 32.77 | 3.36 | 0.84 |
|  | B | Females | 47.47 | 18.18 | 28.28 | 4.04 | 2.02 |
|  |  | Males | 49.24 | 10.61 | 31.82 | 3.79 | 4.55 |
|  |  | Non-binary gender |  |  |  |  |  |
|  |  | Total | 48.48 | 13.85 | 30.30 | 3.90 | 3.46 |
|  | C | Females | 55.79 | 15.79 | 26.32 | 1.05 | 1.05 |
|  |  | Males | 48.94 | 21.28 | 26.95 | 0.00 | 2.84 |
|  |  | Non-binary gender | 0.00 | 0.00 | 100.00 | 0.00 | 0.00 |
|  |  | Total | 51.48 | 18.99 | 27.00 | 0.42 | 2.11 |
| **Russia** | A | Females | 55.24 | 15.27 | 19.76 | 7.19 | 2.54 |
|  |  | Males | 58.96 | 19.40 | 16.42 | 3.73 | 1.49 |
|  |  | Non-binary gender | 43.75 | 31.25 | 6.25 | 6.25 | 12.50 |
|  |  | Total | 55.62 | 16.26 | 18.95 | 6.60 | 2.57 |
|  | B | Females | 43.75 | 16.41 | 28.91 | 3.13 | 7.81 |
|  |  | Males | 46.22 | 19.33 | 25.21 | 5.04 | 4.20 |
|  |  | Non-binary gender | 42.86 | 57.14 | 0.00 | 0.00 | 0.00 |
|  |  | Total | 44.88 | 18.90 | 26.38 | 3.94 | 5.91 |
|  | C | Females | 48.64 | 17.90 | 24.12 | 7.39 | 1.95 |
|  |  | Males | 55.17 | 12.64 | 22.99 | 4.60 | 4.60 |
|  |  | Non-binary gender | 77.78 | 11.11 | 11.11 | 0.00 | 0.00 |
|  |  | Total | 50.99 | 16.43 | 23.51 | 6.52 | 2.55 |
| **Malaysia** | A | Females | 69.51 | 17.07 | 4.88 | 4.88 | 3.66 |
|  |  | Males | 53.85 | 23.08 | 0.00 | 15.38 | 7.69 |
|  |  | Non-binary gender | 100.00 | 0.00 | 0.00 | 0.00 | 0.00 |
|  |  | Total | 67.71 | 17.71 | 4.17 | 6.25 | 4.17 |
|  | B | Females | 37.50 | 31.25 | 15.63 | 12.50 | 3.13 |
|  |  | Males | 50.00 | 30.00 | 10.00 | 10.00 | 0.00 |
|  |  | Non-binary gender |  |  |  |  |  |
|  |  | Total | 40.48 | 30.95 | 14.29 | 11.90 | 2.38 |
|  | C | Females | 49.23 | 12.31 | 10.77 | 21.54 | 6.15 |
|  |  | Males | 21.43 | 7.14 | 35.71 | 28.57 | 7.14 |
|  |  | Non-binary gender | 100.00 | 0.00 | 0.00 | 0.00 | 0.00 |
|  |  | Total | 45.00 | 11.25 | 15.00 | 22.50 | 6.25 |
| **Hungary** | A | Females | 71.88 | 15.63 | 3.13 | 0.00 | 9.38 |
|  |  | Males | 77.78 | 11.11 | 11.11 | 0.00 | 0.00 |
|  |  | Non-binary gender |  |  |  |  |  |
|  |  | Total | 73.17 | 14.63 | 4.88 | 0.00 | 7.32 |
|  | B | Females | 50.00 | 10.00 | 20.00 | 0.00 | 20.00 |
|  |  | Males | 75.00 | 0.00 | 25.00 | 0.00 | 0.00 |
|  |  | Non-binary gender |  |  |  |  |  |
|  |  | Total | 57.14 | 7.14 | 21.43 | 0.00 | 14.29 |
|  | C | Females | 62.07 | 20.69 | 10.34 | 6.90 | 0.00 |
|  |  | Males | 66.67 | 0.00 | 33.33 | 0.00 | 0.00 |
|  |  | Non-binary gender |  |  |  |  |  |
|  |  | Total | 62.50 | 18.75 | 12.50 | 6.25 | 0.00 |
| **TOTAL** |  |  | 52.28 | 15.95 | 18.28 | 7.14 | 6.35 |
| **J9. Do you believe that COVID-19 is a sign of divine power to destroy our planet?** | | | | | | | |
| **Bulgaria** | A | Females | 89.36 | 4.26 | 4.26 | 2.13 | 0.00 |
|  |  | Males | 95.56 | 0.00 | 2.22 | 0.00 | 2.22 |
|  |  | Non-binary gender | 100.00 | 0.00 | 0.00 | 0.00 | 0.00 |
|  |  | Total | 92.55 | 2.13 | 3.19 | 1.06 | 1.06 |
|  | B | Females | 83.93 | 12.50 | 0.00 | 0.00 | 3.57 |
|  |  | Males | 83.33 | 3.33 | 6.67 | 3.33 | 3.33 |
|  |  | Non-binary gender |  |  |  |  |  |
|  |  | Total | 83.72 | 9.30 | 2.33 | 1.16 | 3.49 |
|  | C | Females | 81.36 | 6.78 | 8.47 | 0.00 | 3.39 |
|  |  | Males | 70.37 | 14.81 | 11.11 | 0.00 | 3.70 |
|  |  | Non-binary gender |  |  |  |  |  |
|  |  | Total | 77.91 | 9.30 | 9.30 | 0.00 | 3.49 |
| **Croatia** | A | Females | 90.91 | 5.84 | 2.60 | 0.43 | 0.22 |
|  |  | Males | 92.50 | 5.00 | 2.50 | 0.00 | 0.00 |
|  |  | Non-binary gender | 100.00 | 0.00 | 0.00 | 0.00 | 0.00 |
|  |  | Total | 91.16 | 5.71 | 2.58 | 0.37 | 0.18 |
|  | B | Females | 82.10 | 8.95 | 5.24 | 1.75 | 1.97 |
|  |  | Males | 88.62 | 5.39 | 5.39 | 0.60 | 0.00 |
|  |  | Non-binary gender | 66.67 | 0.00 | 16.67 | 0.00 | 16.67 |
|  |  | Total | 83.68 | 7.92 | 5.39 | 1.43 | 1.58 |
|  | C | Females | 85.33 | 6.89 | 6.29 | 0.60 | 0.90 |
|  |  | Males | 86.00 | 6.00 | 8.00 | 0.00 | 0.00 |
|  |  | Non-binary gender | 100.00 | 0.00 | 0.00 | 0.00 | 0.00 |
|  |  | Total | 85.49 | 6.74 | 6.48 | 0.52 | 0.78 |
| **Georgia** | A | Females | 70.67 | 11.73 | 9.78 | 5.31 | 2.51 |
|  |  | Males | 66.26 | 15.34 | 8.59 | 4.91 | 4.91 |
|  |  | Non-binary gender | 100.00 | 0.00 | 0.00 | 0.00 | 0.00 |
|  |  | Total | 69.41 | 12.81 | 9.37 | 5.16 | 3.25 |
|  | B | Females | 76.51 | 10.74 | 8.72 | 0.00 | 4.03 |
|  |  | Males | 75.95 | 8.86 | 10.13 | 1.27 | 3.80 |
|  |  | Non-binary gender | 66.67 | 0.00 | 33.33 | 0.00 | 0.00 |
|  |  | Total | 76.19 | 9.96 | 9.52 | 0.43 | 3.90 |
|  | C | Females | 78.01 | 9.42 | 8.90 | 1.05 | 2.62 |
|  |  | Males | 89.47 | 2.63 | 5.26 | 2.63 | 0.00 |
|  |  | Non-binary gender | 0.00 | 100.00 | 0.00 | 0.00 | 0.00 |
|  |  | Total | 79.57 | 8.70 | 8.26 | 1.30 | 2.17 |
| **Greece** | A | Females | 89.34 | 6.59 | 2.86 | 0.99 | 0.22 |
|  |  | Males | 92.86 | 3.21 | 2.14 | 1.79 | 0.00 |
|  |  | Non-binary gender | 76.92 | 23.08 | 0.00 | 0.00 | 0.00 |
|  |  | Total | 90.02 | 5.99 | 2.66 | 1.16 | 0.17 |
|  | B | Females | 87.19 | 6.36 | 4.93 | 0.88 | 0.64 |
|  |  | Males | 92.67 | 3.30 | 2.44 | 0.49 | 1.10 |
|  |  | Non-binary gender | 88.89 | 3.70 | 3.70 | 0.00 | 3.70 |
|  |  | Total | 89.34 | 5.14 | 3.95 | 0.71 | 0.86 |
|  | C | Females | 83.08 | 7.58 | 6.51 | 2.04 | 0.79 |
|  |  | Males | 88.17 | 5.35 | 5.35 | 0.56 | 0.56 |
|  |  | Non-binary gender | 86.21 | 6.90 | 6.90 | 0.00 | 0.00 |
|  |  | Total | 83.96 | 7.21 | 6.32 | 1.77 | 0.74 |
| **India** | A | Females | 40.57 | 25.47 | 27.36 | 4.72 | 1.89 |
|  |  | Males | 51.28 | 17.95 | 17.95 | 7.69 | 5.13 |
|  |  | Non-binary gender |  |  |  |  |  |
|  |  | Total | 43.45 | 23.45 | 24.83 | 5.52 | 2.76 |
|  | B | Females | 55.26 | 9.65 | 23.68 | 7.02 | 4.39 |
|  |  | Males | 56.56 | 14.75 | 22.13 | 4.92 | 1.64 |
|  |  | Non-binary gender |  |  |  |  |  |
|  |  | Total | 55.93 | 12.29 | 22.88 | 5.93 | 2.97 |
|  | C | Females | 62.07 | 11.49 | 20.69 | 3.45 | 2.30 |
|  |  | Males | 59.62 | 13.46 | 19.23 | 3.85 | 3.85 |
|  |  | Non-binary gender | 0.00 | 0.00 | 0.00 | 100.00 | 0.00 |
|  |  | Total | 60.71 | 12.14 | 20.00 | 4.29 | 2.86 |
| **Latvia** | A | Females | 92.08 | 2.64 | 5.28 | 0.00 | 0.00 |
|  |  | Males | 97.50 | 0.00 | 2.50 | 0.00 | 0.00 |
|  |  | Non-binary gender | 80.00 | 20.00 | 0.00 | 0.00 | 0.00 |
|  |  | Total | 92.49 | 2.59 | 4.92 | 0.00 | 0.00 |
|  | B | Females | 84.73 | 11.33 | 2.96 | 0.49 | 0.49 |
|  |  | Males | 88.62 | 2.44 | 8.94 | 0.00 | 0.00 |
|  |  | Non-binary gender | 100.00 | 0.00 | 0.00 | 0.00 | 0.00 |
|  |  | Total | 86.28 | 7.93 | 5.18 | 0.30 | 0.30 |
|  | C | Females | 87.32 | 4.93 | 6.34 | 1.06 | 0.35 |
|  |  | Males | 81.63 | 8.16 | 8.16 | 2.04 | 0.00 |
|  |  | Non-binary gender |  |  |  |  |  |
|  |  | Total | 86.49 | 5.41 | 6.61 | 1.20 | 0.30 |
| **Lithuania** | A | Females | 93.62 | 4.26 | 1.60 | 0.53 | 0.00 |
|  |  | Males | 88.24 | 5.88 | 2.94 | 0.00 | 2.94 |
|  |  | Non-binary gender | 100.00 | 0.00 | 0.00 | 0.00 | 0.00 |
|  |  | Total | 92.89 | 4.44 | 1.78 | 0.44 | 0.44 |
|  | B | Females | 87.88 | 3.03 | 6.06 | 3.03 | 0.00 |
|  |  | Males | 87.50 | 0.00 | 12.50 | 0.00 | 0.00 |
|  |  | Non-binary gender |  |  |  |  |  |
|  |  | Total | 87.80 | 2.44 | 7.32 | 2.44 | 0.00 |
|  | C | Females | 94.64 | 1.79 | 1.79 | 0.00 | 1.79 |
|  |  | Males | 76.92 | 15.38 | 7.69 | 0.00 | 0.00 |
|  |  | Non-binary gender |  |  |  |  |  |
|  |  | Total | 91.30 | 4.35 | 2.90 | 0.00 | 1.45 |
| **Nigeria** | A | Females | 60.29 | 10.29 | 25.00 | 1.47 | 2.94 |
|  |  | Males | 49.02 | 9.80 | 33.33 | 3.92 | 3.92 |
|  |  | Non-binary gender |  |  |  |  |  |
|  |  | Total | 55.46 | 10.08 | 28.57 | 2.52 | 3.36 |
|  | B | Females | 30.30 | 11.11 | 46.46 | 3.03 | 9.09 |
|  |  | Males | 37.12 | 12.88 | 38.64 | 4.55 | 6.82 |
|  |  | Non-binary gender |  |  |  |  |  |
|  |  | Total | 34.20 | 12.12 | 41.99 | 3.90 | 7.79 |
|  | C | Females | 32.63 | 13.68 | 38.95 | 3.16 | 11.58 |
|  |  | Males | 34.75 | 14.18 | 34.75 | 3.55 | 12.77 |
|  |  | Non-binary gender | 0.00 | 100.00 | 0.00 | 0.00 | 0.00 |
|  |  | Total | 33.76 | 14.35 | 36.29 | 3.38 | 12.24 |
| **Russia** | A | Females | 80.09 | 6.74 | 10.48 | 1.50 | 1.20 |
|  |  | Males | 77.61 | 8.21 | 11.19 | 0.00 | 2.99 |
|  |  | Non-binary gender | 56.25 | 31.25 | 6.25 | 0.00 | 6.25 |
|  |  | Total | 79.22 | 7.46 | 10.51 | 1.22 | 1.59 |
|  | B | Females | 75.00 | 9.38 | 10.94 | 3.91 | 0.78 |
|  |  | Males | 75.63 | 5.04 | 13.45 | 4.20 | 1.68 |
|  |  | Non-binary gender | 42.86 | 28.57 | 14.29 | 14.29 | 0.00 |
|  |  | Total | 74.41 | 7.87 | 12.20 | 4.33 | 1.18 |
|  | C | Females | 75.10 | 9.34 | 12.06 | 1.95 | 1.56 |
|  |  | Males | 77.01 | 8.05 | 8.05 | 4.60 | 2.30 |
|  |  | Non-binary gender | 66.67 | 11.11 | 22.22 | 0.00 | 0.00 |
|  |  | Total | 75.35 | 9.07 | 11.33 | 2.55 | 1.70 |
| **Malaysia** | A | Females | 73.17 | 14.63 | 6.10 | 4.88 | 1.22 |
|  |  | Males | 38.46 | 7.69 | 7.69 | 30.77 | 15.38 |
|  |  | Non-binary gender | 100.00 | 0.00 | 0.00 | 0.00 | 0.00 |
|  |  | Total | 68.75 | 13.54 | 6.25 | 8.33 | 3.13 |
|  | B | Females | 28.13 | 15.63 | 12.50 | 40.63 | 3.13 |
|  |  | Males | 50.00 | 10.00 | 0.00 | 30.00 | 10.00 |
|  |  | Non-binary gender |  |  |  |  |  |
|  |  | Total | 33.33 | 14.29 | 9.52 | 38.10 | 4.76 |
|  | C | Females | 35.38 | 26.15 | 9.23 | 18.46 | 10.77 |
|  |  | Males | 28.57 | 21.43 | 28.57 | 21.43 | 0.00 |
|  |  | Non-binary gender | 100.00 | 0.00 | 0.00 | 0.00 | 0.00 |
|  |  | Total | 35.00 | 25.00 | 12.50 | 18.75 | 8.75 |
| **Hungary** | A | Females | 93.75 | 6.25 | 0.00 | 0.00 | 0.00 |
|  |  | Males | 100.00 | 0.00 | 0.00 | 0.00 | 0.00 |
|  |  | Non-binary gender |  |  |  |  |  |
|  |  | Total | 95.12 | 4.88 | 0.00 | 0.00 | 0.00 |
|  | B | Females | 90.00 | 0.00 | 10.00 | 0.00 | 0.00 |
|  |  | Males | 100.00 | 0.00 | 0.00 | 0.00 | 0.00 |
|  |  | Non-binary gender |  |  |  |  |  |
|  |  | Total | 92.86 | 0.00 | 7.14 | 0.00 | 0.00 |
|  | C | Females | 89.66 | 6.90 | 3.45 | 0.00 | 0.00 |
|  |  | Males | 100.00 | 0.00 | 0.00 | 0.00 | 0.00 |
|  |  | Non-binary gender |  |  |  |  |  |
|  |  | Total | 90.63 | 6.25 | 3.13 | 0.00 | 0.00 |
| **TOTAL** |  |  | 80.90 | 7.59 | 8.07 | 1.90 | 1.54 |
| **J10. Do you believe that airplanes secretly spray people with various chemicals?** | | | | | | | |
| **Bulgaria** | A | Females | 80.85 | 6.38 | 4.26 | 4.26 | 4.26 |
|  |  | Males | 91.11 | 0.00 | 4.44 | 4.44 | 0.00 |
|  |  | Non-binary gender | 50.00 | 50.00 | 0.00 | 0.00 | 0.00 |
|  |  | Total | 85.11 | 4.26 | 4.26 | 4.26 | 2.13 |
|  | B | Females | 55.36 | 12.50 | 19.64 | 3.57 | 8.93 |
|  |  | Males | 70.00 | 10.00 | 6.67 | 6.67 | 6.67 |
|  |  | Non-binary gender |  |  |  |  |  |
|  |  | Total | 60.47 | 11.63 | 15.12 | 4.65 | 8.14 |
|  | C | Females | 37.29 | 16.95 | 23.73 | 15.25 | 6.78 |
|  |  | Males | 66.67 | 11.11 | 3.70 | 7.41 | 11.11 |
|  |  | Non-binary gender |  |  |  |  |  |
|  |  | Total | 46.51 | 15.12 | 17.44 | 12.79 | 8.14 |
| **Croatia** | A | Females | 83.33 | 8.23 | 5.84 | 1.95 | 0.65 |
|  |  | Males | 83.75 | 7.50 | 6.25 | 0.00 | 2.50 |
|  |  | Non-binary gender | 100.00 | 0.00 | 0.00 | 0.00 | 0.00 |
|  |  | Total | 83.43 | 8.10 | 5.89 | 1.66 | 0.92 |
|  | B | Females | 70.31 | 12.45 | 10.48 | 3.28 | 3.49 |
|  |  | Males | 77.84 | 8.38 | 5.99 | 3.59 | 4.19 |
|  |  | Non-binary gender | 66.67 | 16.67 | 16.67 | 0.00 | 0.00 |
|  |  | Total | 72.27 | 11.41 | 9.35 | 3.33 | 3.65 |
|  | C | Females | 76.35 | 10.18 | 8.68 | 1.50 | 3.29 |
|  |  | Males | 76.00 | 6.00 | 8.00 | 2.00 | 8.00 |
|  |  | Non-binary gender | 100.00 | 0.00 | 0.00 | 0.00 | 0.00 |
|  |  | Total | 76.42 | 9.59 | 8.55 | 1.55 | 3.89 |
| **Georgia** | A | Females | 78.49 | 10.34 | 7.82 | 0.84 | 2.51 |
|  |  | Males | 77.91 | 7.98 | 9.20 | 1.84 | 3.07 |
|  |  | Non-binary gender | 100.00 | 0.00 | 0.00 | 0.00 | 0.00 |
|  |  | Total | 78.39 | 9.56 | 8.22 | 1.15 | 2.68 |
|  | B | Females | 85.23 | 6.71 | 6.71 | 1.34 | 0.00 |
|  |  | Males | 81.01 | 7.59 | 6.33 | 5.06 | 0.00 |
|  |  | Non-binary gender | 66.67 | 0.00 | 33.33 | 0.00 | 0.00 |
|  |  | Total | 83.55 | 6.93 | 6.93 | 2.60 | 0.00 |
|  | C | Females | 80.63 | 7.85 | 6.81 | 2.09 | 2.62 |
|  |  | Males | 89.47 | 2.63 | 7.89 | 0.00 | 0.00 |
|  |  | Non-binary gender | 0.00 | 100.00 | 0.00 | 0.00 | 0.00 |
|  |  | Total | 81.74 | 7.39 | 6.96 | 1.74 | 2.17 |
| **Greece** | A | Females | 83.52 | 8.35 | 5.93 | 1.32 | 0.88 |
|  |  | Males | 85.00 | 7.50 | 5.36 | 1.07 | 1.07 |
|  |  | Non-binary gender | 84.62 | 7.69 | 7.69 | 0.00 | 0.00 |
|  |  | Total | 83.87 | 8.15 | 5.82 | 1.25 | 0.91 |
|  | B | Females | 81.30 | 8.99 | 6.60 | 2.23 | 0.88 |
|  |  | Males | 90.10 | 4.40 | 3.06 | 1.47 | 0.98 |
|  |  | Non-binary gender | 77.78 | 7.41 | 14.81 | 0.00 | 0.00 |
|  |  | Total | 84.68 | 7.18 | 5.33 | 1.90 | 0.90 |
|  | C | Females | 77.02 | 10.92 | 8.32 | 2.09 | 1.64 |
|  |  | Males | 81.97 | 9.30 | 5.63 | 1.97 | 1.13 |
|  |  | Non-binary gender | 82.76 | 10.34 | 0.00 | 3.45 | 3.45 |
|  |  | Total | 77.92 | 10.65 | 7.76 | 2.09 | 1.58 |
| **India** | A | Females | 50.00 | 23.58 | 22.64 | 2.83 | 0.94 |
|  |  | Males | 61.54 | 10.26 | 17.95 | 10.26 | 0.00 |
|  |  | Non-binary gender |  |  |  |  |  |
|  |  | Total | 53.10 | 20.00 | 21.38 | 4.83 | 0.69 |
|  | B | Females | 68.42 | 4.39 | 19.30 | 6.14 | 1.75 |
|  |  | Males | 70.49 | 8.20 | 18.85 | 1.64 | 0.82 |
|  |  | Non-binary gender |  |  |  |  |  |
|  |  | Total | 69.49 | 6.36 | 19.07 | 3.81 | 1.27 |
|  | C | Females | 72.41 | 11.49 | 12.64 | 3.45 | 0.00 |
|  |  | Males | 73.08 | 9.62 | 11.54 | 1.92 | 3.85 |
|  |  | Non-binary gender | 0.00 | 0.00 | 0.00 | 0.00 | 100.00 |
|  |  | Total | 72.14 | 10.71 | 12.14 | 2.86 | 2.14 |
| **Latvia** | A | Females | 92.38 | 3.52 | 3.52 | 0.59 | 0.00 |
|  |  | Males | 90.00 | 5.00 | 5.00 | 0.00 | 0.00 |
|  |  | Non-binary gender | 100.00 | 0.00 | 0.00 | 0.00 | 0.00 |
|  |  | Total | 92.23 | 3.63 | 3.63 | 0.52 | 0.00 |
|  | B | Females | 86.21 | 2.96 | 9.85 | 0.49 | 0.49 |
|  |  | Males | 84.55 | 8.13 | 6.50 | 0.81 | 0.00 |
|  |  | Non-binary gender | 100.00 | 0.00 | 0.00 | 0.00 | 0.00 |
|  |  | Total | 85.67 | 4.88 | 8.54 | 0.61 | 0.30 |
|  | C | Females | 85.56 | 8.10 | 5.28 | 1.06 | 0.00 |
|  |  | Males | 83.67 | 4.08 | 10.20 | 0.00 | 2.04 |
|  |  | Non-binary gender |  |  |  |  |  |
|  |  | Total | 85.29 | 7.51 | 6.01 | 0.90 | 0.30 |
| **Lithuania** | A | Females | 95.21 | 2.13 | 2.66 | 0.00 | 0.00 |
|  |  | Males | 97.06 | 2.94 | 0.00 | 0.00 | 0.00 |
|  |  | Non-binary gender | 66.67 | 33.33 | 0.00 | 0.00 | 0.00 |
|  |  | Total | 95.11 | 2.67 | 2.22 | 0.00 | 0.00 |
|  | B | Females | 87.88 | 3.03 | 3.03 | 0.00 | 6.06 |
|  |  | Males | 87.50 | 0.00 | 12.50 | 0.00 | 0.00 |
|  |  | Non-binary gender |  |  |  |  |  |
|  |  | Total | 87.80 | 2.44 | 4.88 | 0.00 | 4.88 |
|  | C | Females | 91.07 | 3.57 | 3.57 | 1.79 | 0.00 |
|  |  | Males | 84.62 | 7.69 | 7.69 | 0.00 | 0.00 |
|  |  | Non-binary gender |  |  |  |  |  |
|  |  | Total | 89.86 | 4.35 | 4.35 | 1.45 | 0.00 |
| **Nigeria** | A | Females | 64.71 | 11.76 | 22.06 | 1.47 | 0.00 |
|  |  | Males | 64.71 | 13.73 | 19.61 | 1.96 | 0.00 |
|  |  | Non-binary gender |  |  |  |  |  |
|  |  | Total | 64.71 | 12.61 | 21.01 | 1.68 | 0.00 |
|  | B | Females | 49.49 | 16.16 | 30.30 | 3.03 | 1.01 |
|  |  | Males | 54.55 | 15.91 | 24.24 | 3.79 | 1.52 |
|  |  | Non-binary gender |  |  |  |  |  |
|  |  | Total | 52.38 | 16.02 | 26.84 | 3.46 | 1.30 |
|  | C | Females | 63.16 | 23.16 | 10.53 | 2.11 | 1.05 |
|  |  | Males | 58.16 | 24.82 | 15.60 | 0.71 | 0.71 |
|  |  | Non-binary gender | 100.00 | 0.00 | 0.00 | 0.00 | 0.00 |
|  |  | Total | 60.34 | 24.05 | 13.50 | 1.27 | 0.84 |
| **Russia** | A | Females | 83.08 | 4.94 | 9.58 | 1.95 | 0.45 |
|  |  | Males | 79.85 | 8.96 | 6.72 | 1.49 | 2.99 |
|  |  | Non-binary gender | 56.25 | 18.75 | 18.75 | 0.00 | 6.25 |
|  |  | Total | 82.03 | 5.87 | 9.29 | 1.83 | 0.98 |
|  | B | Females | 70.31 | 10.16 | 9.38 | 7.81 | 2.34 |
|  |  | Males | 68.07 | 8.40 | 13.45 | 7.56 | 2.52 |
|  |  | Non-binary gender | 71.43 | 14.29 | 0.00 | 14.29 | 0.00 |
|  |  | Total | 69.29 | 9.45 | 11.02 | 7.87 | 2.36 |
|  | C | Females | 74.71 | 9.34 | 12.45 | 2.33 | 1.17 |
|  |  | Males | 80.46 | 5.75 | 8.05 | 4.60 | 1.15 |
|  |  | Non-binary gender | 88.89 | 0.00 | 11.11 | 0.00 | 0.00 |
|  |  | Total | 76.49 | 8.22 | 11.33 | 2.83 | 1.13 |
| **Malaysia** | A | Females | 90.24 | 6.10 | 0.00 | 3.66 | 0.00 |
|  |  | Males | 76.92 | 7.69 | 0.00 | 15.38 | 0.00 |
|  |  | Non-binary gender | 100.00 | 0.00 | 0.00 | 0.00 | 0.00 |
|  |  | Total | 88.54 | 6.25 | 0.00 | 5.21 | 0.00 |
|  | B | Females | 65.63 | 9.38 | 6.25 | 18.75 | 0.00 |
|  |  | Males | 90.00 | 0.00 | 0.00 | 10.00 | 0.00 |
|  |  | Non-binary gender |  |  |  |  |  |
|  |  | Total | 71.43 | 7.14 | 4.76 | 16.67 | 0.00 |
|  | C | Females | 63.08 | 15.38 | 4.62 | 15.38 | 1.54 |
|  |  | Males | 50.00 | 21.43 | 0.00 | 21.43 | 7.14 |
|  |  | Non-binary gender | 100.00 | 0.00 | 0.00 | 0.00 | 0.00 |
|  |  | Total | 61.25 | 16.25 | 3.75 | 16.25 | 2.50 |
| **Hungary** | A | Females | 96.88 | 0.00 | 3.13 | 0.00 | 0.00 |
|  |  | Males | 100.00 | 0.00 | 0.00 | 0.00 | 0.00 |
|  |  | Non-binary gender |  |  |  |  |  |
|  |  | Total | 97.56 | 0.00 | 2.44 | 0.00 | 0.00 |
|  | B | Females | 90.00 | 10.00 | 0.00 | 0.00 | 0.00 |
|  |  | Males | 75.00 | 25.00 | 0.00 | 0.00 | 0.00 |
|  |  | Non-binary gender |  |  |  |  |  |
|  |  | Total | 85.71 | 14.29 | 0.00 | 0.00 | 0.00 |
|  | C | Females | 86.21 | 10.34 | 3.45 | 0.00 | 0.00 |
|  |  | Males | 100.00 | 0.00 | 0.00 | 0.00 | 0.00 |
|  |  | Non-binary gender |  |  |  |  |  |
|  |  | Total | 87.50 | 9.38 | 3.13 | 0.00 | 0.00 |
| **TOTAL** |  |  | 79.36 | 8.83 | 8.13 | 2.26 | 1.43 |
| **J11. Do you think that vaccines in general are dangerous and should be avoided?** | | | | | | | |
| **Bulgaria** | A | Females | 76.60 | 10.64 | 10.64 | 0.00 | 2.13 |
|  |  | Males | 84.44 | 8.89 | 2.22 | 2.22 | 2.22 |
|  |  | Non-binary gender | 100.00 | 0.00 | 0.00 | 0.00 | 0.00 |
|  |  | Total | 80.85 | 9.57 | 6.38 | 1.06 | 2.13 |
|  | B | Females | 35.71 | 28.57 | 8.93 | 14.29 | 12.50 |
|  |  | Males | 56.67 | 13.33 | 6.67 | 13.33 | 10.00 |
|  |  | Non-binary gender |  |  |  |  |  |
|  |  | Total | 43.02 | 23.26 | 8.14 | 13.95 | 11.63 |
|  | C | Females | 32.20 | 23.73 | 27.12 | 8.47 | 8.47 |
|  |  | Males | 59.26 | 22.22 | 7.41 | 3.70 | 7.41 |
|  |  | Non-binary gender |  |  |  |  |  |
|  |  | Total | 40.70 | 23.26 | 20.93 | 6.98 | 8.14 |
| **Croatia** | A | Females | 70.78 | 15.80 | 7.36 | 3.25 | 2.81 |
|  |  | Males | 78.75 | 13.75 | 3.75 | 2.50 | 1.25 |
|  |  | Non-binary gender | 100.00 | 0.00 | 0.00 | 0.00 | 0.00 |
|  |  | Total | 72.01 | 15.47 | 6.81 | 3.13 | 2.58 |
|  | B | Females | 42.58 | 23.80 | 16.59 | 8.95 | 8.08 |
|  |  | Males | 58.68 | 19.76 | 10.78 | 4.19 | 6.59 |
|  |  | Non-binary gender | 66.67 | 16.67 | 0.00 | 16.67 | 0.00 |
|  |  | Total | 47.07 | 22.66 | 14.90 | 7.77 | 7.61 |
|  | C | Females | 53.29 | 23.95 | 12.28 | 5.69 | 4.79 |
|  |  | Males | 42.00 | 32.00 | 18.00 | 0.00 | 8.00 |
|  |  | Non-binary gender | 100.00 | 0.00 | 0.00 | 0.00 | 0.00 |
|  |  | Total | 52.07 | 24.87 | 12.95 | 4.92 | 5.18 |
| **Georgia** | A | Females | 71.51 | 14.53 | 7.82 | 4.19 | 1.96 |
|  |  | Males | 69.33 | 12.27 | 10.43 | 5.52 | 2.45 |
|  |  | Non-binary gender | 100.00 | 0.00 | 0.00 | 0.00 | 0.00 |
|  |  | Total | 70.94 | 13.77 | 8.60 | 4.59 | 2.10 |
|  | B | Females | 57.72 | 25.50 | 9.40 | 4.70 | 2.68 |
|  |  | Males | 65.82 | 16.46 | 12.66 | 2.53 | 2.53 |
|  |  | Non-binary gender | 33.33 | 33.33 | 33.33 | 0.00 | 0.00 |
|  |  | Total | 60.17 | 22.51 | 10.82 | 3.90 | 2.60 |
|  | C | Females | 54.45 | 16.23 | 15.18 | 8.90 | 5.24 |
|  |  | Males | 65.79 | 18.42 | 10.53 | 5.26 | 0.00 |
|  |  | Non-binary gender | 100.00 | 0.00 | 0.00 | 0.00 | 0.00 |
|  |  | Total | 56.52 | 16.52 | 14.35 | 8.26 | 4.35 |
| **Greece** | A | Females | 76.15 | 14.62 | 5.93 | 1.87 | 1.43 |
|  |  | Males | 86.07 | 8.57 | 2.86 | 1.79 | 0.71 |
|  |  | Non-binary gender | 76.92 | 15.38 | 7.69 | 0.00 | 0.00 |
|  |  | Total | 78.47 | 13.22 | 5.24 | 1.83 | 1.25 |
|  | B | Females | 66.75 | 19.01 | 8.59 | 3.82 | 1.83 |
|  |  | Males | 78.36 | 13.81 | 4.52 | 2.08 | 1.22 |
|  |  | Non-binary gender | 66.67 | 18.52 | 7.41 | 3.70 | 3.70 |
|  |  | Total | 71.27 | 16.98 | 6.99 | 3.14 | 1.62 |
|  | C | Females | 63.04 | 19.07 | 11.94 | 3.74 | 2.21 |
|  |  | Males | 68.73 | 18.87 | 8.17 | 2.54 | 1.69 |
|  |  | Non-binary gender | 68.97 | 13.79 | 10.34 | 6.90 | 0.00 |
|  |  | Total | 64.06 | 18.97 | 11.30 | 3.58 | 2.09 |
| **India** | A | Females | 38.68 | 27.36 | 30.19 | 2.83 | 0.94 |
|  |  | Males | 51.28 | 7.69 | 35.90 | 5.13 | 0.00 |
|  |  | Non-binary gender |  |  |  |  |  |
|  |  | Total | 42.07 | 22.07 | 31.72 | 3.45 | 0.69 |
|  | B | Females | 58.77 | 19.30 | 15.79 | 5.26 | 0.88 |
|  |  | Males | 59.84 | 14.75 | 16.39 | 5.74 | 3.28 |
|  |  | Non-binary gender |  |  |  |  |  |
|  |  | Total | 59.32 | 16.95 | 16.10 | 5.51 | 2.12 |
|  | C | Females | 65.52 | 14.94 | 17.24 | 1.15 | 1.15 |
|  |  | Males | 61.54 | 13.46 | 15.38 | 5.77 | 3.85 |
|  |  | Non-binary gender | 0.00 | 0.00 | 0.00 | 100.00 | 0.00 |
|  |  | Total | 63.57 | 14.29 | 16.43 | 3.57 | 2.14 |
| **Latvia** | A | Females | 80.94 | 9.09 | 6.74 | 1.47 | 1.76 |
|  |  | Males | 87.50 | 2.50 | 5.00 | 2.50 | 2.50 |
|  |  | Non-binary gender | 60.00 | 20.00 | 20.00 | 0.00 | 0.00 |
|  |  | Total | 81.35 | 8.55 | 6.74 | 1.55 | 1.81 |
|  | B | Females | 49.75 | 22.17 | 19.21 | 4.93 | 3.94 |
|  |  | Males | 66.67 | 15.45 | 13.01 | 1.63 | 3.25 |
|  |  | Non-binary gender | 100.00 | 0.00 | 0.00 | 0.00 | 0.00 |
|  |  | Total | 56.40 | 19.51 | 16.77 | 3.66 | 3.66 |
|  | C | Females | 52.46 | 16.90 | 19.37 | 7.04 | 4.23 |
|  |  | Males | 61.22 | 8.16 | 24.49 | 2.04 | 4.08 |
|  |  | Non-binary gender |  |  |  |  |  |
|  |  | Total | 53.75 | 15.62 | 20.12 | 6.31 | 4.20 |
| **Lithuania** | A | Females | 88.83 | 7.45 | 3.19 | 0.53 | 0.00 |
|  |  | Males | 88.24 | 2.94 | 5.88 | 0.00 | 2.94 |
|  |  | Non-binary gender | 100.00 | 0.00 | 0.00 | 0.00 | 0.00 |
|  |  | Total | 88.89 | 6.67 | 3.56 | 0.44 | 0.44 |
|  | B | Females | 69.70 | 12.12 | 12.12 | 0.00 | 6.06 |
|  |  | Males | 62.50 | 0.00 | 25.00 | 12.50 | 0.00 |
|  |  | Non-binary gender |  |  |  |  |  |
|  |  | Total | 68.29 | 9.76 | 14.63 | 2.44 | 4.88 |
|  | C | Females | 69.64 | 19.64 | 7.14 | 1.79 | 1.79 |
|  |  | Males | 61.54 | 15.38 | 15.38 | 0.00 | 7.69 |
|  |  | Non-binary gender |  |  |  |  |  |
|  |  | Total | 68.12 | 18.84 | 8.70 | 1.45 | 2.90 |
| **Nigeria** | A | Females | 51.47 | 25.00 | 19.12 | 2.94 | 1.47 |
|  |  | Males | 58.82 | 19.61 | 21.57 | 0.00 | 0.00 |
|  |  | Non-binary gender |  |  |  |  |  |
|  |  | Total | 54.62 | 22.69 | 20.17 | 1.68 | 0.84 |
|  | B | Females | 40.40 | 19.19 | 36.36 | 3.03 | 1.01 |
|  |  | Males | 43.18 | 19.70 | 28.79 | 2.27 | 6.06 |
|  |  | Non-binary gender |  |  |  |  |  |
|  |  | Total | 41.99 | 19.48 | 32.03 | 2.60 | 3.90 |
|  | C | Females | 53.68 | 31.58 | 12.63 | 0.00 | 2.11 |
|  |  | Males | 60.28 | 22.70 | 14.89 | 0.71 | 1.42 |
|  |  | Non-binary gender | 0.00 | 100.00 | 0.00 | 0.00 | 0.00 |
|  |  | Total | 57.38 | 26.58 | 13.92 | 0.42 | 1.69 |
| **Russia** | A | Females | 53.44 | 20.06 | 17.37 | 5.09 | 4.04 |
|  |  | Males | 56.72 | 19.40 | 13.43 | 5.22 | 5.22 |
|  |  | Non-binary gender | 43.75 | 31.25 | 6.25 | 6.25 | 12.50 |
|  |  | Total | 53.79 | 20.17 | 16.50 | 5.13 | 4.40 |
|  | B | Females | 41.41 | 15.63 | 25.00 | 10.16 | 7.81 |
|  |  | Males | 50.42 | 16.81 | 21.85 | 5.88 | 5.04 |
|  |  | Non-binary gender | 28.57 | 57.14 | 0.00 | 14.29 | 0.00 |
|  |  | Total | 45.28 | 17.32 | 22.83 | 8.27 | 6.30 |
|  | C | Females | 41.25 | 19.46 | 21.79 | 8.56 | 8.95 |
|  |  | Males | 52.87 | 20.69 | 17.24 | 4.60 | 4.60 |
|  |  | Non-binary gender | 55.56 | 0.00 | 44.44 | 0.00 | 0.00 |
|  |  | Total | 44.48 | 19.26 | 21.25 | 7.37 | 7.65 |
| **Malaysia** | A | Females | 92.68 | 3.66 | 0.00 | 0.00 | 3.66 |
|  |  | Males | 76.92 | 7.69 | 0.00 | 15.38 | 0.00 |
|  |  | Non-binary gender | 100.00 | 0.00 | 0.00 | 0.00 | 0.00 |
|  |  | Total | 90.63 | 4.17 | 0.00 | 2.08 | 3.13 |
|  | B | Females | 62.50 | 15.63 | 6.25 | 15.63 | 0.00 |
|  |  | Males | 100.00 | 0.00 | 0.00 | 0.00 | 0.00 |
|  |  | Non-binary gender |  |  |  |  |  |
|  |  | Total | 71.43 | 11.90 | 4.76 | 11.90 | 0.00 |
|  | C | Females | 73.85 | 10.77 | 1.54 | 12.31 | 1.54 |
|  |  | Males | 50.00 | 7.14 | 7.14 | 28.57 | 7.14 |
|  |  | Non-binary gender | 0.00 | 100.00 | 0.00 | 0.00 | 0.00 |
|  |  | Total | 68.75 | 11.25 | 2.50 | 15.00 | 2.50 |
| **Hungary** | A | Females | 87.50 | 9.38 | 0.00 | 3.13 | 0.00 |
|  |  | Males | 100.00 | 0.00 | 0.00 | 0.00 | 0.00 |
|  |  | Non-binary gender |  |  |  |  |  |
|  |  | Total | 90.24 | 7.32 | 0.00 | 2.44 | 0.00 |
|  | B | Females | 70.00 | 10.00 | 10.00 | 0.00 | 10.00 |
|  |  | Males | 75.00 | 25.00 | 0.00 | 0.00 | 0.00 |
|  |  | Non-binary gender |  |  |  |  |  |
|  |  | Total | 71.43 | 14.29 | 7.14 | 0.00 | 7.14 |
|  | C | Females | 89.66 | 6.90 | 3.45 | 0.00 | 0.00 |
|  |  | Males | 66.67 | 33.33 | 0.00 | 0.00 | 0.00 |
|  |  | Non-binary gender |  |  |  |  |  |
|  |  | Total | 87.50 | 9.38 | 3.13 | 0.00 | 0.00 |
| TOTAL |  |  | 64.05 | 17.37 | 11.60 | 4.04 | 2.95 |
| **J12. The government is secretly involved in the murder of innocent citizens and/or well-known public figures.** | | | | | | | |
| **Bulgaria** | A | Females | 57.45 | 19.15 | 12.77 | 6.38 | 4.26 |
|  |  | Males | 75.56 | 4.44 | 6.67 | 4.44 | 8.89 |
|  |  | Non-binary gender | 100.00 | 0.00 | 0.00 | 0.00 | 0.00 |
|  |  | Total | 67.02 | 11.70 | 9.57 | 5.32 | 6.38 |
|  | B | Females | 44.64 | 23.21 | 10.71 | 8.93 | 12.50 |
|  |  | Males | 33.33 | 26.67 | 20.00 | 10.00 | 10.00 |
|  |  | Non-binary gender |  |  |  |  |  |
|  |  | Total | 40.70 | 24.42 | 13.95 | 9.30 | 11.63 |
|  | C | Females | 37.29 | 16.95 | 23.73 | 11.86 | 10.17 |
|  |  | Males | 48.15 | 22.22 | 14.81 | 7.41 | 7.41 |
|  |  | Non-binary gender |  |  |  |  |  |
|  |  | Total | 40.70 | 18.60 | 20.93 | 10.47 | 9.30 |
| **Croatia** | A | Females | 63.64 | 20.56 | 8.87 | 4.76 | 2.16 |
|  |  | Males | 63.75 | 15.00 | 12.50 | 2.50 | 6.25 |
|  |  | Non-binary gender | 0.00 | 0.00 | 100.00 | 0.00 | 0.00 |
|  |  | Total | 63.54 | 19.71 | 9.58 | 4.42 | 2.76 |
|  | B | Females | 53.06 | 17.47 | 16.38 | 6.33 | 6.77 |
|  |  | Males | 53.89 | 17.37 | 12.57 | 9.58 | 6.59 |
|  |  | Non-binary gender | 66.67 | 16.67 | 16.67 | 0.00 | 0.00 |
|  |  | Total | 53.41 | 17.43 | 15.37 | 7.13 | 6.66 |
|  | C | Females | 54.19 | 19.46 | 16.47 | 3.89 | 5.99 |
|  |  | Males | 42.00 | 28.00 | 12.00 | 8.00 | 10.00 |
|  |  | Non-binary gender | 0.00 | 100.00 | 0.00 | 0.00 | 0.00 |
|  |  | Total | 52.33 | 20.98 | 15.80 | 4.40 | 6.48 |
| **Georgia** | A | Females | 61.73 | 17.60 | 11.45 | 3.91 | 5.31 |
|  |  | Males | 59.51 | 15.95 | 15.34 | 6.13 | 3.07 |
|  |  | Non-binary gender | 100.00 | 0.00 | 0.00 | 0.00 | 0.00 |
|  |  | Total | 61.19 | 17.02 | 12.62 | 4.59 | 4.59 |
|  | B | Females | 51.01 | 18.79 | 19.46 | 3.36 | 7.38 |
|  |  | Males | 46.84 | 22.78 | 22.78 | 2.53 | 5.06 |
|  |  | Non-binary gender | 33.33 | 0.00 | 66.67 | 0.00 | 0.00 |
|  |  | Total | 49.35 | 19.91 | 21.21 | 3.03 | 6.49 |
|  | C | Females | 57.07 | 15.18 | 16.75 | 6.28 | 4.71 |
|  |  | Males | 60.53 | 13.16 | 15.79 | 10.53 | 0.00 |
|  |  | Non-binary gender | 0.00 | 0.00 | 0.00 | 0.00 | 100.00 |
|  |  | Total | 57.39 | 14.78 | 16.52 | 6.96 | 4.35 |
| **Greece** | A | Females | 62.42 | 19.01 | 11.32 | 4.51 | 2.75 |
|  |  | Males | 65.00 | 16.79 | 10.36 | 5.36 | 2.50 |
|  |  | Non-binary gender | 46.15 | 30.77 | 15.38 | 0.00 | 7.69 |
|  |  | Total | 62.84 | 18.62 | 11.14 | 4.66 | 2.74 |
|  | B | Females | 58.87 | 20.37 | 12.17 | 5.17 | 3.42 |
|  |  | Males | 63.94 | 16.38 | 9.90 | 5.75 | 4.03 |
|  |  | Non-binary gender | 33.33 | 25.93 | 29.63 | 3.70 | 7.41 |
|  |  | Total | 60.51 | 18.89 | 11.51 | 5.38 | 3.71 |
|  | C | Females | 56.65 | 18.00 | 15.39 | 5.89 | 4.07 |
|  |  | Males | 61.97 | 18.03 | 10.14 | 6.48 | 3.38 |
|  |  | Non-binary gender | 55.17 | 10.34 | 10.34 | 17.24 | 6.90 |
|  |  | Total | 57.51 | 17.90 | 14.46 | 6.14 | 4.00 |
| **India** | A | Females | 50.94 | 18.87 | 22.64 | 5.66 | 1.89 |
|  |  | Males | 53.85 | 20.51 | 15.38 | 7.69 | 2.56 |
|  |  | Non-binary gender |  |  |  |  |  |
|  |  | Total | 51.72 | 19.31 | 20.69 | 6.21 | 2.07 |
|  | B | Females | 57.02 | 8.77 | 24.56 | 5.26 | 4.39 |
|  |  | Males | 56.56 | 13.11 | 22.13 | 3.28 | 4.92 |
|  |  | Non-binary gender |  |  |  |  |  |
|  |  | Total | 56.78 | 11.02 | 23.31 | 4.24 | 4.66 |
|  | C | Females | 58.62 | 18.39 | 18.39 | 2.30 | 2.30 |
|  |  | Males | 53.85 | 13.46 | 19.23 | 3.85 | 9.62 |
|  |  | Non-binary gender | 0.00 | 0.00 | 100.00 | 0.00 | 0.00 |
|  |  | Total | 56.43 | 16.43 | 19.29 | 2.86 | 5.00 |
| **Latvia** | A | Females | 78.89 | 12.61 | 6.74 | 0.59 | 1.17 |
|  |  | Males | 60.00 | 12.50 | 25.00 | 0.00 | 2.50 |
|  |  | Non-binary gender | 80.00 | 20.00 | 0.00 | 0.00 | 0.00 |
|  |  | Total | 76.94 | 12.69 | 8.55 | 0.52 | 1.30 |
|  | B | Females | 64.53 | 14.29 | 15.27 | 4.43 | 1.48 |
|  |  | Males | 59.35 | 8.94 | 19.51 | 10.57 | 1.63 |
|  |  | Non-binary gender | 0.00 | 0.00 | 100.00 | 0.00 | 0.00 |
|  |  | Total | 62.20 | 12.20 | 17.38 | 6.71 | 1.52 |
|  | C | Females | 69.72 | 12.68 | 13.38 | 1.76 | 2.46 |
|  |  | Males | 59.18 | 6.12 | 26.53 | 4.08 | 4.08 |
|  |  | Non-binary gender |  |  |  |  |  |
|  |  | Total | 68.17 | 11.71 | 15.32 | 2.10 | 2.70 |
| **Lithuania** | A | Females | 80.85 | 11.70 | 6.38 | 1.06 | 0.00 |
|  |  | Males | 82.35 | 8.82 | 5.88 | 0.00 | 2.94 |
|  |  | Non-binary gender | 33.33 | 0.00 | 33.33 | 33.33 | 0.00 |
|  |  | Total | 80.44 | 11.11 | 6.67 | 1.33 | 0.44 |
|  | B | Females | 69.70 | 6.06 | 18.18 | 0.00 | 6.06 |
|  |  | Males | 50.00 | 25.00 | 25.00 | 0.00 | 0.00 |
|  |  | Non-binary gender |  |  |  |  |  |
|  |  | Total | 65.85 | 9.76 | 19.51 | 0.00 | 4.88 |
|  | C | Females | 80.36 | 7.14 | 7.14 | 1.79 | 3.57 |
|  |  | Males | 76.92 | 0.00 | 7.69 | 7.69 | 7.69 |
|  |  | Non-binary gender |  |  |  |  |  |
|  |  | Total | 79.71 | 5.80 | 7.25 | 2.90 | 4.35 |
| **Nigeria** | A | Females | 55.88 | 13.24 | 27.94 | 2.94 | 0.00 |
|  |  | Males | 62.75 | 15.69 | 19.61 | 1.96 | 0.00 |
|  |  | Non-binary gender |  |  |  |  |  |
|  |  | Total | 58.82 | 14.29 | 24.37 | 2.52 | 0.00 |
|  | B | Females | 44.44 | 25.25 | 27.27 | 3.03 | 0.00 |
|  |  | Males | 48.48 | 16.67 | 28.79 | 3.03 | 3.03 |
|  |  | Non-binary gender |  |  |  |  |  |
|  |  | Total | 46.75 | 20.35 | 28.14 | 3.03 | 1.73 |
|  | C | Females | 60.00 | 20.00 | 16.84 | 0.00 | 3.16 |
|  |  | Males | 61.70 | 19.15 | 17.02 | 0.71 | 1.42 |
|  |  | Non-binary gender | 0.00 | 0.00 | 100.00 | 0.00 | 0.00 |
|  |  | Total | 60.76 | 19.41 | 17.30 | 0.42 | 2.11 |
| **Russia** | A | Females | 46.41 | 13.17 | 24.85 | 9.58 | 5.99 |
|  |  | Males | 41.79 | 20.15 | 20.15 | 5.97 | 11.94 |
|  |  | Non-binary gender | 37.50 | 25.00 | 12.50 | 18.75 | 6.25 |
|  |  | Total | 45.48 | 14.55 | 23.84 | 9.17 | 6.97 |
|  | B | Females | 45.31 | 24.22 | 17.97 | 7.81 | 4.69 |
|  |  | Males | 43.70 | 17.65 | 23.53 | 6.72 | 8.40 |
|  |  | Non-binary gender | 42.86 | 0.00 | 14.29 | 42.86 | 0.00 |
|  |  | Total | 44.49 | 20.47 | 20.47 | 8.27 | 6.30 |
|  | C | Females | 38.52 | 18.29 | 24.12 | 10.89 | 8.17 |
|  |  | Males | 42.53 | 19.54 | 18.39 | 3.45 | 16.09 |
|  |  | Non-binary gender | 55.56 | 22.22 | 22.22 | 0.00 | 0.00 |
|  |  | Total | 39.94 | 18.70 | 22.66 | 8.78 | 9.92 |
| **Malaysia** | A | Females | 80.49 | 15.85 | 2.44 | 0.00 | 1.22 |
|  |  | Males | 53.85 | 15.38 | 0.00 | 23.08 | 7.69 |
|  |  | Non-binary gender | 100.00 | 0.00 | 0.00 | 0.00 | 0.00 |
|  |  | Total | 77.08 | 15.63 | 2.08 | 3.13 | 2.08 |
|  | B | Females | 53.13 | 25.00 | 6.25 | 15.63 | 0.00 |
|  |  | Males | 80.00 | 10.00 | 0.00 | 10.00 | 0.00 |
|  |  | Non-binary gender |  |  |  |  |  |
|  |  | Total | 59.52 | 21.43 | 4.76 | 14.29 | 0.00 |
|  | C | Females | 52.31 | 9.23 | 10.77 | 20.00 | 7.69 |
|  |  | Males | 50.00 | 21.43 | 0.00 | 14.29 | 14.29 |
|  |  | Non-binary gender | 100.00 | 0.00 | 0.00 | 0.00 | 0.00 |
|  |  | Total | 52.50 | 11.25 | 8.75 | 18.75 | 8.75 |
| **Hungary** | A | Females | 78.13 | 15.63 | 6.25 | 0.00 | 0.00 |
|  |  | Males | 88.89 | 11.11 | 0.00 | 0.00 | 0.00 |
|  |  | Non-binary gender |  |  |  |  |  |
|  |  | Total | 80.49 | 14.63 | 4.88 | 0.00 | 0.00 |
|  | B | Females | 60.00 | 10.00 | 20.00 | 0.00 | 10.00 |
|  |  | Males | 50.00 | 0.00 | 50.00 | 0.00 | 0.00 |
|  |  | Non-binary gender |  |  |  |  |  |
|  |  | Total | 57.14 | 7.14 | 28.57 | 0.00 | 7.14 |
|  | C | Females | 62.07 | 20.69 | 10.34 | 3.45 | 3.45 |
|  |  | Males | 33.33 | 0.00 | 33.33 | 0.00 | 33.33 |
|  |  | Non-binary gender |  |  |  |  |  |
|  |  | Total | 59.38 | 18.75 | 12.50 | 3.13 | 6.25 |
| **TOTAL** |  |  | 58.27 | 17.24 | 14.84 | 5.43 | 4.22 |
| **J13. Global warming and climate change is a greatly exaggerated myth to serve various political and financial interests.** | | | | | | | |
| **Bulgaria** | A | Females | 80.85 | 10.64 | 2.13 | 4.26 | 2.13 |
|  |  | Males | 73.33 | 20.00 | 4.44 | 2.22 | 0.00 |
|  |  | Non-binary gender | 100.00 | 0.00 | 0.00 | 0.00 | 0.00 |
|  |  | Total | 77.66 | 14.89 | 3.19 | 3.19 | 1.06 |
|  | B | Females | 66.07 | 17.86 | 10.71 | 0.00 | 5.36 |
|  |  | Males | 76.67 | 10.00 | 10.00 | 0.00 | 3.33 |
|  |  | Non-binary gender |  |  |  |  |  |
|  |  | Total | 69.77 | 15.12 | 10.47 | 0.00 | 4.65 |
|  | C | Females | 40.68 | 22.03 | 27.12 | 3.39 | 6.78 |
|  |  | Males | 51.85 | 18.52 | 22.22 | 3.70 | 3.70 |
|  |  | Non-binary gender |  |  |  |  |  |
|  |  | Total | 44.19 | 20.93 | 25.58 | 3.49 | 5.81 |
| **Croatia** | A | Females | 81.60 | 9.74 | 4.55 | 2.60 | 1.52 |
|  |  | Males | 80.00 | 5.00 | 7.50 | 6.25 | 1.25 |
|  |  | Non-binary gender | 100.00 | 0.00 | 0.00 | 0.00 | 0.00 |
|  |  | Total | 81.40 | 9.02 | 4.97 | 3.13 | 1.47 |
|  | B | Females | 70.74 | 10.04 | 10.92 | 4.80 | 3.49 |
|  |  | Males | 59.28 | 11.98 | 12.57 | 8.38 | 7.78 |
|  |  | Non-binary gender | 83.33 | 0.00 | 16.67 | 0.00 | 0.00 |
|  |  | Total | 67.83 | 10.46 | 11.41 | 5.71 | 4.60 |
|  | C | Females | 77.84 | 9.88 | 10.48 | 0.90 | 0.90 |
|  |  | Males | 56.00 | 26.00 | 12.00 | 2.00 | 4.00 |
|  |  | Non-binary gender | 100.00 | 0.00 | 0.00 | 0.00 | 0.00 |
|  |  | Total | 75.13 | 11.92 | 10.62 | 1.04 | 1.30 |
| **Georgia** | A | Females | 75.98 | 9.78 | 8.38 | 3.07 | 2.79 |
|  |  | Males | 58.28 | 17.18 | 10.43 | 8.59 | 5.52 |
|  |  | Non-binary gender | 50.00 | 0.00 | 50.00 | 0.00 | 0.00 |
|  |  | Total | 70.36 | 12.05 | 9.18 | 4.78 | 3.63 |
|  | B | Females | 73.15 | 15.44 | 8.72 | 1.34 | 1.34 |
|  |  | Males | 75.95 | 8.86 | 12.66 | 2.53 | 0.00 |
|  |  | Non-binary gender | 66.67 | 33.33 | 0.00 | 0.00 | 0.00 |
|  |  | Total | 74.03 | 13.42 | 9.96 | 1.73 | 0.87 |
|  | C | Females | 71.73 | 13.09 | 8.90 | 3.66 | 2.62 |
|  |  | Males | 71.05 | 13.16 | 7.89 | 5.26 | 2.63 |
|  |  | Non-binary gender | 100.00 | 0.00 | 0.00 | 0.00 | 0.00 |
|  |  | Total | 71.74 | 13.04 | 8.70 | 3.91 | 2.61 |
| **Greece** | A | Females | 91.87 | 5.71 | 1.76 | 0.55 | 0.11 |
|  |  | Males | 85.71 | 8.93 | 3.57 | 1.07 | 0.71 |
|  |  | Non-binary gender | 100.00 | 0.00 | 0.00 | 0.00 | 0.00 |
|  |  | Total | 90.52 | 6.40 | 2.16 | 0.67 | 0.25 |
|  | B | Females | 90.14 | 4.06 | 3.74 | 0.95 | 1.11 |
|  |  | Males | 87.04 | 6.36 | 3.67 | 1.96 | 0.98 |
|  |  | Non-binary gender | 88.89 | 7.41 | 3.70 | 0.00 | 0.00 |
|  |  | Total | 88.92 | 5.00 | 3.71 | 1.33 | 1.05 |
|  | C | Females | 85.74 | 6.85 | 5.43 | 1.53 | 0.45 |
|  |  | Males | 80.56 | 8.73 | 6.76 | 2.25 | 1.69 |
|  |  | Non-binary gender | 100.00 | 0.00 | 0.00 | 0.00 | 0.00 |
|  |  | Total | 85.08 | 7.07 | 5.58 | 1.63 | 0.65 |
| **India** | A | Females | 42.45 | 24.53 | 29.25 | 1.89 | 1.89 |
|  |  | Males | 51.28 | 17.95 | 23.08 | 5.13 | 2.56 |
|  |  | Non-binary gender |  |  |  |  |  |
|  |  | Total | 44.83 | 22.76 | 27.59 | 2.76 | 2.07 |
|  | B | Females | 58.77 | 13.16 | 21.05 | 5.26 | 1.75 |
|  |  | Males | 70.49 | 9.02 | 17.21 | 1.64 | 1.64 |
|  |  | Non-binary gender |  |  |  |  |  |
|  |  | Total | 64.83 | 11.02 | 19.07 | 3.39 | 1.69 |
|  | C | Females | 70.11 | 12.64 | 12.64 | 1.15 | 3.45 |
|  |  | Males | 69.23 | 7.69 | 17.31 | 1.92 | 3.85 |
|  |  | Non-binary gender | 0.00 | 100.00 | 0.00 | 0.00 | 0.00 |
|  |  | Total | 69.29 | 11.43 | 14.29 | 1.43 | 3.57 |
| **Latvia** | A | Females | 87.39 | 5.87 | 4.11 | 1.47 | 1.17 |
|  |  | Males | 70.00 | 10.00 | 12.50 | 5.00 | 2.50 |
|  |  | Non-binary gender | 80.00 | 0.00 | 0.00 | 20.00 | 0.00 |
|  |  | Total | 85.49 | 6.22 | 4.92 | 2.07 | 1.30 |
|  | B | Females | 82.27 | 5.91 | 7.39 | 1.97 | 2.46 |
|  |  | Males | 69.92 | 12.20 | 14.63 | 1.63 | 1.63 |
|  |  | Non-binary gender | 50.00 | 50.00 | 0.00 | 0.00 | 0.00 |
|  |  | Total | 77.44 | 8.54 | 10.06 | 1.83 | 2.13 |
|  | C | Females | 83.10 | 8.10 | 6.69 | 1.41 | 0.70 |
|  |  | Males | 75.51 | 10.20 | 8.16 | 6.12 | 0.00 |
|  |  | Non-binary gender |  |  |  |  |  |
|  |  | Total | 81.98 | 8.41 | 6.91 | 2.10 | 0.60 |
| **Lithuania** | A | Females | 96.28 | 2.13 | 1.06 | 0.00 | 0.53 |
|  |  | Males | 97.06 | 0.00 | 0.00 | 2.94 | 0.00 |
|  |  | Non-binary gender | 100.00 | 0.00 | 0.00 | 0.00 | 0.00 |
|  |  | Total | 96.44 | 1.78 | 0.89 | 0.44 | 0.44 |
|  | B | Females | 78.79 | 12.12 | 9.09 | 0.00 | 0.00 |
|  |  | Males | 87.50 | 0.00 | 12.50 | 0.00 | 0.00 |
|  |  | Non-binary gender |  |  |  |  |  |
|  |  | Total | 80.49 | 9.76 | 9.76 | 0.00 | 0.00 |
|  | C | Females | 91.07 | 3.57 | 5.36 | 0.00 | 0.00 |
|  |  | Males | 76.92 | 7.69 | 15.38 | 0.00 | 0.00 |
|  |  | Non-binary gender |  |  |  |  |  |
|  |  | Total | 88.41 | 4.35 | 7.25 | 0.00 | 0.00 |
| **Nigeria** | A | Females | 63.24 | 16.18 | 17.65 | 1.47 | 1.47 |
|  |  | Males | 64.71 | 17.65 | 17.65 | 0.00 | 0.00 |
|  |  | Non-binary gender |  |  |  |  |  |
|  |  | Total | 63.87 | 16.81 | 17.65 | 0.84 | 0.84 |
|  | B | Females | 43.43 | 24.24 | 27.27 | 5.05 | 0.00 |
|  |  | Males | 47.73 | 23.48 | 22.73 | 4.55 | 1.52 |
|  |  | Non-binary gender |  |  |  |  |  |
|  |  | Total | 45.89 | 23.81 | 24.68 | 4.76 | 0.87 |
|  | C | Females | 57.89 | 23.16 | 16.84 | 1.05 | 1.05 |
|  |  | Males | 56.03 | 25.53 | 17.02 | 0.71 | 0.71 |
|  |  | Non-binary gender | 0.00 | 0.00 | 0.00 | 100.00 | 0.00 |
|  |  | Total | 56.54 | 24.47 | 16.88 | 1.27 | 0.84 |
| **Russia** | A | Females | 67.22 | 14.82 | 13.47 | 3.29 | 1.20 |
|  |  | Males | 73.13 | 12.69 | 10.45 | 0.75 | 2.99 |
|  |  | Non-binary gender | 62.50 | 0.00 | 31.25 | 0.00 | 6.25 |
|  |  | Total | 68.09 | 14.18 | 13.33 | 2.81 | 1.59 |
|  | B | Females | 52.34 | 17.19 | 24.22 | 3.13 | 3.13 |
|  |  | Males | 52.10 | 19.33 | 20.17 | 6.72 | 1.68 |
|  |  | Non-binary gender | 57.14 | 14.29 | 14.29 | 14.29 | 0.00 |
|  |  | Total | 52.36 | 18.11 | 22.05 | 5.12 | 2.36 |
|  | C | Females | 64.59 | 15.95 | 13.62 | 3.50 | 2.33 |
|  |  | Males | 62.07 | 14.94 | 18.39 | 2.30 | 2.30 |
|  |  | Non-binary gender | 77.78 | 0.00 | 22.22 | 0.00 | 0.00 |
|  |  | Total | 64.31 | 15.30 | 15.01 | 3.12 | 2.27 |
| **Malaysia** | A | Females | 93.90 | 4.88 | 0.00 | 1.22 | 0.00 |
|  |  | Males | 76.92 | 7.69 | 0.00 | 15.38 | 0.00 |
|  |  | Non-binary gender | 100.00 | 0.00 | 0.00 | 0.00 | 0.00 |
|  |  | Total | 91.67 | 5.21 | 0.00 | 3.13 | 0.00 |
|  | B | Females | 40.63 | 28.13 | 15.63 | 12.50 | 3.13 |
|  |  | Males | 60.00 | 10.00 | 20.00 | 10.00 | 0.00 |
|  |  | Non-binary gender |  |  |  |  |  |
|  |  | Total | 45.24 | 23.81 | 16.67 | 11.90 | 2.38 |
|  | C | Females | 64.62 | 13.85 | 4.62 | 12.31 | 4.62 |
|  |  | Males | 50.00 | 14.29 | 14.29 | 14.29 | 7.14 |
|  |  | Non-binary gender | 100.00 | 0.00 | 0.00 | 0.00 | 0.00 |
|  |  | Total | 62.50 | 13.75 | 6.25 | 12.50 | 5.00 |
| **Hungary** | A | Females | 90.63 | 3.13 | 6.25 | 0.00 | 0.00 |
|  |  | Males | 100.00 | 0.00 | 0.00 | 0.00 | 0.00 |
|  |  | Non-binary gender |  |  |  |  |  |
|  |  | Total | 92.68 | 2.44 | 4.88 | 0.00 | 0.00 |
|  | B | Females | 70.00 | 10.00 | 10.00 | 0.00 | 10.00 |
|  |  | Males | 25.00 | 50.00 | 25.00 | 0.00 | 0.00 |
|  |  | Non-binary gender |  |  |  |  |  |
|  |  | Total | 57.14 | 21.43 | 14.29 | 0.00 | 7.14 |
|  | C | Females | 96.55 | 0.00 | 0.00 | 0.00 | 3.45 |
|  |  | Males | 100.00 | 0.00 | 0.00 | 0.00 | 0.00 |
|  |  | Non-binary gender |  |  |  |  |  |
|  |  | Total | 96.88 | 0.00 | 0.00 | 0.00 | 3.13 |
| **TOTAL** |  |  | 78.27 | 9.68 | 8.27 | 2.31 | 1.47 |
| **J14. The power held by the heads of state is smaller than that of small unknown groups that really control the world of politics.** | | | | | | | |
| **Bulgaria** | A | Females | 42.55 | 21.28 | 17.02 | 12.77 | 6.38 |
|  |  | Males | 55.56 | 22.22 | 4.44 | 11.11 | 6.67 |
|  |  | Non-binary gender | 50.00 | 50.00 | 0.00 | 0.00 | 0.00 |
|  |  | Total | 48.94 | 22.34 | 10.64 | 11.70 | 6.38 |
|  | B | Females | 17.86 | 21.43 | 32.14 | 3.57 | 25.00 |
|  |  | Males | 33.33 | 20.00 | 23.33 | 6.67 | 16.67 |
|  |  | Non-binary gender |  |  |  |  |  |
|  |  | Total | 23.26 | 20.93 | 29.07 | 4.65 | 22.09 |
|  | C | Females | 18.64 | 16.95 | 37.29 | 8.47 | 18.64 |
|  |  | Males | 44.44 | 29.63 | 18.52 | 3.70 | 3.70 |
|  |  | Non-binary gender |  |  |  |  |  |
|  |  | Total | 26.74 | 20.93 | 31.40 | 6.98 | 13.95 |
| **Croatia** | A | Females | 36.36 | 23.59 | 19.48 | 11.04 | 9.52 |
|  |  | Males | 36.25 | 23.75 | 22.50 | 8.75 | 8.75 |
|  |  | Non-binary gender | 0.00 | 100.00 | 0.00 | 0.00 | 0.00 |
|  |  | Total | 36.28 | 23.76 | 19.89 | 10.68 | 9.39 |
|  | B | Females | 31.22 | 19.21 | 23.14 | 12.88 | 13.54 |
|  |  | Males | 31.14 | 20.96 | 17.37 | 15.57 | 14.97 |
|  |  | Non-binary gender | 50.00 | 0.00 | 50.00 | 0.00 | 0.00 |
|  |  | Total | 31.38 | 19.49 | 21.87 | 13.47 | 13.79 |
|  | C | Females | 35.03 | 22.75 | 21.56 | 8.38 | 12.28 |
|  |  | Males | 32.00 | 26.00 | 18.00 | 14.00 | 10.00 |
|  |  | Non-binary gender | 50.00 | 0.00 | 50.00 | 0.00 | 0.00 |
|  |  | Total | 34.72 | 23.06 | 21.24 | 9.07 | 11.92 |
| **Georgia** | A | Females | 47.49 | 21.79 | 17.32 | 6.98 | 6.42 |
|  |  | Males | 40.49 | 30.06 | 15.95 | 7.36 | 6.13 |
|  |  | Non-binary gender | 0.00 | 0.00 | 100.00 | 0.00 | 0.00 |
|  |  | Total | 45.12 | 24.28 | 17.21 | 7.07 | 6.31 |
|  | B | Females | 40.94 | 24.16 | 21.48 | 7.38 | 6.04 |
|  |  | Males | 49.37 | 15.19 | 21.52 | 6.33 | 7.59 |
|  |  | Non-binary gender | 33.33 | 66.67 | 0.00 | 0.00 | 0.00 |
|  |  | Total | 43.72 | 21.65 | 21.21 | 6.93 | 6.49 |
|  | C | Females | 42.41 | 23.56 | 20.42 | 3.14 | 10.47 |
|  |  | Males | 44.74 | 21.05 | 21.05 | 13.16 | 0.00 |
|  |  | Non-binary gender | 0.00 | 0.00 | 0.00 | 0.00 | 100.00 |
|  |  | Total | 42.61 | 23.04 | 20.43 | 4.78 | 9.13 |
| **Greece** | A | Females | 42.42 | 23.63 | 18.35 | 10.33 | 5.27 |
|  |  | Males | 37.86 | 21.43 | 17.50 | 13.57 | 9.64 |
|  |  | Non-binary gender | 23.08 | 15.38 | 53.85 | 0.00 | 7.69 |
|  |  | Total | 41.15 | 23.03 | 18.54 | 10.97 | 6.32 |
|  | B | Females | 35.24 | 26.41 | 20.21 | 11.06 | 7.08 |
|  |  | Males | 36.55 | 24.45 | 18.95 | 12.59 | 7.46 |
|  |  | Non-binary gender | 25.93 | 37.04 | 14.81 | 11.11 | 11.11 |
|  |  | Total | 35.63 | 25.78 | 19.65 | 11.66 | 7.28 |
|  | C | Females | 37.80 | 20.77 | 21.90 | 12.34 | 7.19 |
|  |  | Males | 33.80 | 23.94 | 20.28 | 10.70 | 11.27 |
|  |  | Non-binary gender | 48.28 | 20.69 | 20.69 | 6.90 | 3.45 |
|  |  | Total | 37.28 | 21.29 | 21.62 | 11.99 | 7.81 |
| **India** | A | Females | 38.68 | 25.47 | 27.36 | 5.66 | 2.83 |
|  |  | Males | 35.90 | 17.95 | 33.33 | 7.69 | 5.13 |
|  |  | Non-binary gender |  |  |  |  |  |
|  |  | Total | 37.93 | 23.45 | 28.97 | 6.21 | 3.45 |
|  | B | Females | 37.72 | 17.54 | 32.46 | 9.65 | 2.63 |
|  |  | Males | 33.61 | 20.49 | 31.15 | 10.66 | 4.10 |
|  |  | Non-binary gender |  |  |  |  |  |
|  |  | Total | 35.59 | 19.07 | 31.78 | 10.17 | 3.39 |
|  | C | Females | 39.08 | 18.39 | 28.74 | 2.30 | 11.49 |
|  |  | Males | 30.77 | 28.85 | 23.08 | 7.69 | 9.62 |
|  |  | Non-binary gender | 100.00 | 0.00 | 0.00 | 0.00 | 0.00 |
|  |  | Total | 36.43 | 22.14 | 26.43 | 4.29 | 10.71 |
| **Latvia** | A | Females | 52.20 | 23.17 | 16.13 | 6.74 | 1.76 |
|  |  | Males | 52.50 | 22.50 | 15.00 | 2.50 | 7.50 |
|  |  | Non-binary gender | 60.00 | 40.00 | 0.00 | 0.00 | 0.00 |
|  |  | Total | 52.33 | 23.32 | 15.80 | 6.22 | 2.33 |
|  | B | Females | 43.35 | 17.24 | 22.17 | 12.32 | 4.93 |
|  |  | Males | 45.53 | 21.14 | 17.89 | 7.32 | 8.13 |
|  |  | Non-binary gender | 50.00 | 50.00 | 0.00 | 0.00 | 0.00 |
|  |  | Total | 44.21 | 18.90 | 20.43 | 10.37 | 6.10 |
|  | C | Females | 41.55 | 21.48 | 27.11 | 6.34 | 3.52 |
|  |  | Males | 40.82 | 22.45 | 26.53 | 6.12 | 4.08 |
|  |  | Non-binary gender |  |  |  |  |  |
|  |  | Total | 41.44 | 21.62 | 27.03 | 6.31 | 3.60 |
| **Lithuania** | A | Females | 75.00 | 14.89 | 7.98 | 1.60 | 0.53 |
|  |  | Males | 73.53 | 17.65 | 5.88 | 0.00 | 2.94 |
|  |  | Non-binary gender | 33.33 | 0.00 | 33.33 | 0.00 | 33.33 |
|  |  | Total | 74.22 | 15.11 | 8.00 | 1.33 | 1.33 |
|  | B | Females | 69.70 | 0.00 | 27.27 | 3.03 | 0.00 |
|  |  | Males | 37.50 | 37.50 | 25.00 | 0.00 | 0.00 |
|  |  | Non-binary gender |  |  |  |  |  |
|  |  | Total | 63.41 | 7.32 | 26.83 | 2.44 | 0.00 |
|  | C | Females | 62.50 | 19.64 | 12.50 | 1.79 | 3.57 |
|  |  | Males | 61.54 | 15.38 | 7.69 | 15.38 | 0.00 |
|  |  | Non-binary gender |  |  |  |  |  |
|  |  | Total | 62.32 | 18.84 | 11.59 | 4.35 | 2.90 |
| **Nigeria** | A | Females | 42.65 | 13.24 | 39.71 | 4.41 | 0.00 |
|  |  | Males | 54.90 | 23.53 | 17.65 | 3.92 | 0.00 |
|  |  | Non-binary gender |  |  |  |  |  |
|  |  | Total | 47.90 | 17.65 | 30.25 | 4.20 | 0.00 |
|  | B | Females | 41.41 | 20.20 | 33.33 | 5.05 | 0.00 |
|  |  | Males | 50.76 | 22.73 | 20.45 | 3.79 | 2.27 |
|  |  | Non-binary gender |  |  |  |  |  |
|  |  | Total | 46.75 | 21.65 | 25.97 | 4.33 | 1.30 |
|  | C | Females | 61.05 | 20.00 | 16.84 | 1.05 | 1.05 |
|  |  | Males | 58.87 | 19.86 | 19.86 | 0.00 | 1.42 |
|  |  | Non-binary gender | 0.00 | 0.00 | 0.00 | 100.00 | 0.00 |
|  |  | Total | 59.49 | 19.83 | 18.57 | 0.84 | 1.27 |
| **Russia** | A | Females | 46.86 | 19.16 | 23.05 | 7.19 | 3.74 |
|  |  | Males | 49.25 | 16.42 | 26.87 | 4.48 | 2.99 |
|  |  | Non-binary gender | 56.25 | 18.75 | 18.75 | 0.00 | 6.25 |
|  |  | Total | 47.43 | 18.70 | 23.59 | 6.60 | 3.67 |
|  | B | Females | 38.28 | 23.44 | 25.00 | 5.47 | 7.81 |
|  |  | Males | 39.50 | 23.53 | 22.69 | 10.08 | 4.20 |
|  |  | Non-binary gender | 71.43 | 14.29 | 14.29 | 0.00 | 0.00 |
|  |  | Total | 39.76 | 23.23 | 23.62 | 7.48 | 5.91 |
|  | C | Females | 38.52 | 21.79 | 28.02 | 8.56 | 3.11 |
|  |  | Males | 45.98 | 20.69 | 26.44 | 4.60 | 2.30 |
|  |  | Non-binary gender | 66.67 | 11.11 | 22.22 | 0.00 | 0.00 |
|  |  | Total | 41.08 | 21.25 | 27.48 | 7.37 | 2.83 |
| **Malaysia** | A | Females | 69.51 | 14.63 | 9.76 | 3.66 | 2.44 |
|  |  | Males | 53.85 | 15.38 | 0.00 | 30.77 | 0.00 |
|  |  | Non-binary gender | 100.00 | 0.00 | 0.00 | 0.00 | 0.00 |
|  |  | Total | 67.71 | 14.58 | 8.33 | 7.29 | 2.08 |
|  | B | Females | 34.38 | 37.50 | 9.38 | 12.50 | 6.25 |
|  |  | Males | 30.00 | 40.00 | 30.00 | 0.00 | 0.00 |
|  |  | Non-binary gender |  |  |  |  |  |
|  |  | Total | 33.33 | 38.10 | 14.29 | 9.52 | 4.76 |
|  | C | Females | 36.92 | 20.00 | 10.77 | 26.15 | 6.15 |
|  |  | Males | 28.57 | 7.14 | 14.29 | 42.86 | 7.14 |
|  |  | Non-binary gender | 100.00 | 0.00 | 0.00 | 0.00 | 0.00 |
|  |  | Total | 36.25 | 17.50 | 11.25 | 28.75 | 6.25 |
| **Hungary** | A | Females | 65.63 | 12.50 | 6.25 | 12.50 | 3.13 |
|  |  | Males | 66.67 | 22.22 | 11.11 | 0.00 | 0.00 |
|  |  | Non-binary gender |  |  |  |  |  |
|  |  | Total | 65.85 | 14.63 | 7.32 | 9.76 | 2.44 |
|  | B | Females | 20.00 | 30.00 | 20.00 | 10.00 | 20.00 |
|  |  | Males | 50.00 | 25.00 | 25.00 | 0.00 | 0.00 |
|  |  | Non-binary gender |  |  |  |  |  |
|  |  | Total | 28.57 | 28.57 | 21.43 | 7.14 | 14.29 |
|  | C | Females | 65.52 | 10.34 | 10.34 | 10.34 | 3.45 |
|  |  | Males | 33.33 | 33.33 | 0.00 | 33.33 | 0.00 |
|  |  | Non-binary gender |  |  |  |  |  |
|  |  | Total | 62.50 | 12.50 | 9.38 | 12.50 | 3.13 |
|  |  | TOTAL | 40.92 | 22.04 | 20.89 | 9.47 | 6.69 |
| **J15. Secret organizations are communicating with aliens, but they hide it from the public.** | | | | | | | |
| **Bulgaria** | A | Females | 80.85 | 4.26 | 8.51 | 4.26 | 2.13 |
|  |  | Males | 86.67 | 4.44 | 2.22 | 2.22 | 4.44 |
|  |  | Non-binary gender | 100.00 | 0.00 | 0.00 | 0.00 | 0.00 |
|  |  | Total | 84.04 | 4.26 | 5.32 | 3.19 | 3.19 |
|  | B | Females | 57.14 | 17.86 | 17.86 | 1.79 | 5.36 |
|  |  | Males | 40.00 | 26.67 | 23.33 | 0.00 | 10.00 |
|  |  | Non-binary gender |  |  |  |  |  |
|  |  | Total | 51.16 | 20.93 | 19.77 | 1.16 | 6.98 |
|  | C | Females | 42.37 | 20.34 | 18.64 | 10.17 | 8.47 |
|  |  | Males | 48.15 | 29.63 | 18.52 | 0.00 | 3.70 |
|  |  | Non-binary gender |  |  |  |  |  |
|  |  | Total | 44.19 | 23.26 | 18.60 | 6.98 | 6.98 |
| **Croatia** | A | Females | 83.12 | 8.23 | 6.49 | 1.73 | 0.43 |
|  |  | Males | 85.00 | 8.75 | 5.00 | 1.25 | 0.00 |
|  |  | Non-binary gender | 100.00 | 0.00 | 0.00 | 0.00 | 0.00 |
|  |  | Total | 83.43 | 8.29 | 6.26 | 1.66 | 0.37 |
|  | B | Females | 75.76 | 9.17 | 9.83 | 1.31 | 3.93 |
|  |  | Males | 71.86 | 14.37 | 7.19 | 1.20 | 5.39 |
|  |  | Non-binary gender | 66.67 | 16.67 | 16.67 | 0.00 | 0.00 |
|  |  | Total | 74.64 | 10.62 | 9.19 | 1.27 | 4.28 |
|  | C | Females | 80.24 | 6.59 | 7.19 | 2.69 | 3.29 |
|  |  | Males | 70.00 | 8.00 | 14.00 | 2.00 | 6.00 |
|  |  | Non-binary gender | 100.00 | 0.00 | 0.00 | 0.00 | 0.00 |
|  |  | Total | 79.02 | 6.74 | 8.03 | 2.59 | 3.63 |
| **Georgia** | A | Females | 79.61 | 10.89 | 5.59 | 2.79 | 1.12 |
|  |  | Males | 61.96 | 17.18 | 11.04 | 4.29 | 5.52 |
|  |  | Non-binary gender | 100.00 | 0.00 | 0.00 | 0.00 | 0.00 |
|  |  | Total | 74.19 | 12.81 | 7.27 | 3.25 | 2.49 |
|  | B | Females | 79.19 | 10.07 | 8.72 | 0.67 | 1.34 |
|  |  | Males | 75.95 | 10.13 | 12.66 | 0.00 | 1.27 |
|  |  | Non-binary gender | 100.00 | 0.00 | 0.00 | 0.00 | 0.00 |
|  |  | Total | 78.35 | 9.96 | 9.96 | 0.43 | 1.30 |
|  | C | Females | 79.06 | 6.81 | 8.90 | 1.05 | 4.19 |
|  |  | Males | 76.32 | 7.89 | 10.53 | 2.63 | 2.63 |
|  |  | Non-binary gender | 100.00 | 0.00 | 0.00 | 0.00 | 0.00 |
|  |  | Total | 78.70 | 6.96 | 9.13 | 1.30 | 3.91 |
| **Greece** | A | Females | 92.09 | 5.71 | 1.65 | 0.44 | 0.11 |
|  |  | Males | 91.43 | 4.64 | 2.86 | 0.36 | 0.71 |
|  |  | Non-binary gender | 76.92 | 23.08 | 0.00 | 0.00 | 0.00 |
|  |  | Total | 91.77 | 5.65 | 1.91 | 0.42 | 0.25 |
|  | B | Females | 88.94 | 6.68 | 3.34 | 0.56 | 0.48 |
|  |  | Males | 89.36 | 5.01 | 3.79 | 0.37 | 1.47 |
|  |  | Non-binary gender | 85.19 | 3.70 | 7.41 | 0.00 | 3.70 |
|  |  | Total | 89.06 | 5.99 | 3.57 | 0.48 | 0.90 |
|  | C | Females | 88.91 | 5.55 | 4.13 | 0.74 | 0.68 |
|  |  | Males | 87.32 | 6.48 | 4.23 | 0.85 | 1.13 |
|  |  | Non-binary gender | 93.10 | 3.45 | 3.45 | 0.00 | 0.00 |
|  |  | Total | 88.70 | 5.67 | 4.14 | 0.74 | 0.74 |
| **India** | A | Females | 47.17 | 17.92 | 31.13 | 2.83 | 0.94 |
|  |  | Males | 64.10 | 10.26 | 23.08 | 2.56 | 0.00 |
|  |  | Non-binary gender |  |  |  |  |  |
|  |  | Total | 51.72 | 15.86 | 28.97 | 2.76 | 0.69 |
|  | B | Females | 57.02 | 9.65 | 23.68 | 6.14 | 3.51 |
|  |  | Males | 60.66 | 8.20 | 23.77 | 6.56 | 0.82 |
|  |  | Non-binary gender |  |  |  |  |  |
|  |  | Total | 58.90 | 8.90 | 23.73 | 6.36 | 2.12 |
|  | C | Females | 52.87 | 14.94 | 20.69 | 8.05 | 3.45 |
|  |  | Males | 55.77 | 13.46 | 21.15 | 3.85 | 5.77 |
|  |  | Non-binary gender | 0.00 | 100.00 | 0.00 | 0.00 | 0.00 |
|  |  | Total | 53.57 | 15.00 | 20.71 | 6.43 | 4.29 |
| **Latvia** | A | Females | 90.32 | 4.99 | 4.11 | 0.59 | 0.00 |
|  |  | Males | 87.50 | 2.50 | 7.50 | 0.00 | 2.50 |
|  |  | Non-binary gender | 100.00 | 0.00 | 0.00 | 0.00 | 0.00 |
|  |  | Total | 90.16 | 4.66 | 4.40 | 0.52 | 0.26 |
|  | B | Females | 83.74 | 7.39 | 6.90 | 0.49 | 1.48 |
|  |  | Males | 81.30 | 8.13 | 4.88 | 4.88 | 0.81 |
|  |  | Non-binary gender | 100.00 | 0.00 | 0.00 | 0.00 | 0.00 |
|  |  | Total | 82.93 | 7.62 | 6.10 | 2.13 | 1.22 |
|  | C | Females | 87.32 | 3.52 | 7.04 | 1.41 | 0.70 |
|  |  | Males | 81.63 | 4.08 | 4.08 | 2.04 | 8.16 |
|  |  | Non-binary gender |  |  |  |  |  |
|  |  | Total | 86.49 | 3.60 | 6.61 | 1.50 | 1.80 |
| **Lithuania** | A | Females | 93.09 | 3.72 | 2.66 | 0.53 | 0.00 |
|  |  | Males | 91.18 | 8.82 | 0.00 | 0.00 | 0.00 |
|  |  | Non-binary gender | 33.33 | 33.33 | 0.00 | 33.33 | 0.00 |
|  |  | Total | 92.00 | 4.89 | 2.22 | 0.89 | 0.00 |
|  | B | Females | 87.88 | 6.06 | 6.06 | 0.00 | 0.00 |
|  |  | Males | 75.00 | 12.50 | 12.50 | 0.00 | 0.00 |
|  |  | Non-binary gender |  |  |  |  |  |
|  |  | Total | 85.37 | 7.32 | 7.32 | 0.00 | 0.00 |
|  | C | Females | 78.57 | 17.86 | 3.57 | 0.00 | 0.00 |
|  |  | Males | 84.62 | 0.00 | 7.69 | 7.69 | 0.00 |
|  |  | Non-binary gender |  |  |  |  |  |
|  |  | Total | 79.71 | 14.49 | 4.35 | 1.45 | 0.00 |
| **Nigeria** | A | Females | 47.06 | 19.12 | 30.88 | 2.94 | 0.00 |
|  |  | Males | 66.67 | 15.69 | 15.69 | 1.96 | 0.00 |
|  |  | Non-binary gender |  |  |  |  |  |
|  |  | Total | 55.46 | 17.65 | 24.37 | 2.52 | 0.00 |
|  | B | Females | 50.51 | 22.22 | 23.23 | 4.04 | 0.00 |
|  |  | Males | 48.48 | 24.24 | 21.21 | 3.03 | 3.03 |
|  |  | Non-binary gender |  |  |  |  |  |
|  |  | Total | 49.35 | 23.38 | 22.08 | 3.46 | 1.73 |
|  | C | Females | 50.53 | 27.37 | 20.00 | 0.00 | 2.11 |
|  |  | Males | 55.32 | 23.40 | 19.86 | 0.00 | 1.42 |
|  |  | Non-binary gender | 0.00 | 0.00 | 100.00 | 0.00 | 0.00 |
|  |  | Total | 53.16 | 24.89 | 20.25 | 0.00 | 1.69 |
| **Russia** | A | Females | 84.58 | 4.19 | 7.78 | 1.05 | 2.40 |
|  |  | Males | 79.85 | 7.46 | 11.19 | 0.00 | 1.49 |
|  |  | Non-binary gender | 68.75 | 6.25 | 18.75 | 0.00 | 6.25 |
|  |  | Total | 83.50 | 4.77 | 8.56 | 0.86 | 2.32 |
|  | B | Females | 71.88 | 7.81 | 9.38 | 8.59 | 2.34 |
|  |  | Males | 69.75 | 3.36 | 17.65 | 3.36 | 5.88 |
|  |  | Non-binary gender | 57.14 | 0.00 | 28.57 | 14.29 | 0.00 |
|  |  | Total | 70.47 | 5.51 | 13.78 | 6.30 | 3.94 |
|  | C | Females | 76.26 | 6.61 | 11.67 | 2.72 | 2.72 |
|  |  | Males | 78.16 | 3.45 | 12.64 | 1.15 | 4.60 |
|  |  | Non-binary gender | 88.89 | 0.00 | 11.11 | 0.00 | 0.00 |
|  |  | Total | 77.05 | 5.67 | 11.90 | 2.27 | 3.12 |
| **Malaysia** | A | Females | 95.12 | 4.88 | 0.00 | 0.00 | 0.00 |
|  |  | Males | 69.23 | 15.38 | 0.00 | 15.38 | 0.00 |
|  |  | Non-binary gender | 100.00 | 0.00 | 0.00 | 0.00 | 0.00 |
|  |  | Total | 91.67 | 6.25 | 0.00 | 2.08 | 0.00 |
|  | B | Females | 59.38 | 18.75 | 9.38 | 9.38 | 3.13 |
|  |  | Males | 90.00 | 10.00 | 0.00 | 0.00 | 0.00 |
|  |  | Non-binary gender |  |  |  |  |  |
|  |  | Total | 66.67 | 16.67 | 7.14 | 7.14 | 2.38 |
|  | C | Females | 60.00 | 13.85 | 3.08 | 13.85 | 9.23 |
|  |  | Males | 35.71 | 14.29 | 14.29 | 28.57 | 7.14 |
|  |  | Non-binary gender | 100.00 | 0.00 | 0.00 | 0.00 | 0.00 |
|  |  | Total | 56.25 | 13.75 | 5.00 | 16.25 | 8.75 |
| **Hungary** | A | Females | 96.88 | 3.13 | 0.00 | 0.00 | 0.00 |
|  |  | Males | 100.00 | 0.00 | 0.00 | 0.00 | 0.00 |
|  |  | Non-binary gender |  |  |  |  |  |
|  |  | Total | 97.56 | 2.44 | 0.00 | 0.00 | 0.00 |
|  | B | Females | 70.00 | 20.00 | 10.00 | 0.00 | 0.00 |
|  |  | Males | 100.00 | 0.00 | 0.00 | 0.00 | 0.00 |
|  |  | Non-binary gender |  |  |  |  |  |
|  |  | Total | 78.57 | 14.29 | 7.14 | 0.00 | 0.00 |
|  | C | Females | 89.66 | 3.45 | 3.45 | 0.00 | 3.45 |
|  |  | Males | 66.67 | 33.33 | 0.00 | 0.00 | 0.00 |
|  |  | Non-binary gender |  |  |  |  |  |
|  |  | Total | 87.50 | 6.25 | 3.13 | 0.00 | 3.13 |
| **TOTAL** |  |  | 81.68 | 7.86 | 7.30 | 1.55 | 1.61 |
| **J16. Groups of scientists manipulate, invent or conceal evidence to deceive the public.** | | | | | | | |
| **Bulgaria** | A | Females | 46.81 | 21.28 | 23.40 | 6.38 | 2.13 |
|  |  | Males | 62.22 | 20.00 | 6.67 | 6.67 | 4.44 |
|  |  | Non-binary gender | 0.00 | 50.00 | 0.00 | 0.00 | 50.00 |
|  |  | Total | 53.19 | 21.28 | 14.89 | 6.38 | 4.26 |
|  | B | Females | 26.79 | 16.07 | 33.93 | 7.14 | 16.07 |
|  |  | Males | 26.67 | 26.67 | 23.33 | 10.00 | 13.33 |
|  |  | Non-binary gender |  |  |  |  |  |
|  |  | Total | 26.74 | 19.77 | 30.23 | 8.14 | 15.12 |
|  | C | Females | 15.25 | 13.56 | 28.81 | 20.34 | 22.03 |
|  |  | Males | 48.15 | 29.63 | 14.81 | 7.41 | 0.00 |
|  |  | Non-binary gender |  |  |  |  |  |
|  |  | Total | 25.58 | 18.60 | 24.42 | 16.28 | 15.12 |
| **Croatia** | A | Females | 56.06 | 22.08 | 14.50 | 4.76 | 2.60 |
|  |  | Males | 68.75 | 11.25 | 10.00 | 5.00 | 5.00 |
|  |  | Non-binary gender | 100.00 | 0.00 | 0.00 | 0.00 | 0.00 |
|  |  | Total | 58.01 | 20.44 | 13.81 | 4.79 | 2.95 |
|  | B | Females | 37.34 | 23.36 | 18.56 | 7.86 | 12.88 |
|  |  | Males | 41.92 | 21.56 | 20.96 | 6.59 | 8.98 |
|  |  | Non-binary gender | 50.00 | 33.33 | 16.67 | 0.00 | 0.00 |
|  |  | Total | 38.67 | 22.98 | 19.18 | 7.45 | 11.73 |
|  | C | Females | 45.21 | 20.06 | 21.86 | 6.59 | 6.29 |
|  |  | Males | 36.00 | 22.00 | 22.00 | 10.00 | 10.00 |
|  |  | Non-binary gender | 50.00 | 50.00 | 0.00 | 0.00 | 0.00 |
|  |  | Total | 44.04 | 20.47 | 21.76 | 6.99 | 6.74 |
| **Georgia** | A | Females | 51.12 | 25.14 | 15.08 | 4.75 | 3.91 |
|  |  | Males | 41.10 | 20.86 | 23.93 | 6.75 | 7.36 |
|  |  | Non-binary gender | 50.00 | 50.00 | 0.00 | 0.00 | 0.00 |
|  |  | Total | 47.99 | 23.90 | 17.78 | 5.35 | 4.97 |
|  | B | Females | 44.97 | 28.86 | 12.75 | 8.72 | 4.70 |
|  |  | Males | 45.57 | 25.32 | 22.78 | 2.53 | 3.80 |
|  |  | Non-binary gender | 33.33 | 0.00 | 33.33 | 33.33 | 0.00 |
|  |  | Total | 45.02 | 27.27 | 16.45 | 6.93 | 4.33 |
|  | C | Females | 46.07 | 23.04 | 19.90 | 4.71 | 6.28 |
|  |  | Males | 50.00 | 26.32 | 10.53 | 5.26 | 7.89 |
|  |  | Non-binary gender | 0.00 | 0.00 | 0.00 | 0.00 | 100.00 |
|  |  | Total | 46.52 | 23.48 | 18.26 | 4.78 | 6.96 |
| **Greece** | A | Females | 49.89 | 27.25 | 12.97 | 6.92 | 2.97 |
|  |  | Males | 53.21 | 22.86 | 12.50 | 7.14 | 4.29 |
|  |  | Non-binary gender | 46.15 | 23.08 | 15.38 | 15.38 | 0.00 |
|  |  | Total | 50.62 | 26.18 | 12.88 | 7.07 | 3.24 |
|  | B | Females | 41.85 | 31.03 | 17.18 | 5.49 | 4.46 |
|  |  | Males | 50.12 | 24.94 | 13.45 | 7.09 | 4.40 |
|  |  | Non-binary gender | 29.63 | 29.63 | 25.93 | 7.41 | 7.41 |
|  |  | Total | 44.91 | 28.64 | 15.84 | 6.14 | 4.47 |
|  | C | Females | 36.50 | 28.35 | 20.32 | 9.45 | 5.38 |
|  |  | Males | 38.31 | 32.11 | 15.21 | 9.01 | 5.35 |
|  |  | Non-binary gender | 44.83 | 24.14 | 17.24 | 0.00 | 13.79 |
|  |  | Total | 36.91 | 28.92 | 19.43 | 9.25 | 5.49 |
| **India** | A | Females | 44.34 | 23.58 | 25.47 | 4.72 | 1.89 |
|  |  | Males | 43.59 | 15.38 | 33.33 | 5.13 | 2.56 |
|  |  | Non-binary gender |  |  |  |  |  |
|  |  | Total | 44.14 | 21.38 | 27.59 | 4.83 | 2.07 |
|  | B | Females | 37.72 | 18.42 | 26.32 | 12.28 | 5.26 |
|  |  | Males | 39.34 | 18.03 | 34.43 | 4.10 | 4.10 |
|  |  | Non-binary gender |  |  |  |  |  |
|  |  | Total | 38.56 | 18.22 | 30.51 | 8.05 | 4.66 |
|  | C | Females | 44.83 | 17.24 | 28.74 | 4.60 | 4.60 |
|  |  | Males | 34.62 | 15.38 | 34.62 | 7.69 | 7.69 |
|  |  | Non-binary gender | 0.00 | 0.00 | 100.00 | 0.00 | 0.00 |
|  |  | Total | 40.71 | 16.43 | 31.43 | 5.71 | 5.71 |
| **Latvia** | A | Females | 69.21 | 17.01 | 12.61 | 1.17 | 0.00 |
|  |  | Males | 82.50 | 7.50 | 10.00 | 0.00 | 0.00 |
|  |  | Non-binary gender | 80.00 | 0.00 | 20.00 | 0.00 | 0.00 |
|  |  | Total | 70.73 | 15.80 | 12.44 | 1.04 | 0.00 |
|  | B | Females | 53.20 | 22.66 | 16.26 | 4.43 | 3.45 |
|  |  | Males | 62.60 | 16.26 | 16.26 | 2.44 | 2.44 |
|  |  | Non-binary gender | 0.00 | 50.00 | 0.00 | 50.00 | 0.00 |
|  |  | Total | 56.40 | 20.43 | 16.16 | 3.96 | 3.05 |
|  | C | Females | 57.04 | 21.13 | 15.49 | 2.46 | 3.87 |
|  |  | Males | 55.10 | 16.33 | 16.33 | 6.12 | 6.12 |
|  |  | Non-binary gender |  |  |  |  |  |
|  |  | Total | 56.76 | 20.42 | 15.62 | 3.00 | 4.20 |
| **Lithuania** | A | Females | 79.79 | 12.23 | 6.91 | 1.06 | 0.00 |
|  |  | Males | 85.29 | 8.82 | 0.00 | 5.88 | 0.00 |
|  |  | Non-binary gender | 33.33 | 33.33 | 33.33 | 0.00 | 0.00 |
|  |  | Total | 80.00 | 12.00 | 6.22 | 1.78 | 0.00 |
|  | B | Females | 72.73 | 9.09 | 12.12 | 0.00 | 6.06 |
|  |  | Males | 62.50 | 12.50 | 25.00 | 0.00 | 0.00 |
|  |  | Non-binary gender |  |  |  |  |  |
|  |  | Total | 70.73 | 9.76 | 14.63 | 0.00 | 4.88 |
|  | C | Females | 51.79 | 32.14 | 10.71 | 5.36 | 0.00 |
|  |  | Males | 69.23 | 7.69 | 7.69 | 7.69 | 7.69 |
|  |  | Non-binary gender |  |  |  |  |  |
|  |  | Total | 55.07 | 27.54 | 10.14 | 5.80 | 1.45 |
| **Nigeria** | A | Females | 33.82 | 26.47 | 35.29 | 4.41 | 0.00 |
|  |  | Males | 54.90 | 17.65 | 23.53 | 3.92 | 0.00 |
|  |  | Non-binary gender |  |  |  |  |  |
|  |  | Total | 42.86 | 22.69 | 30.25 | 4.20 | 0.00 |
|  | B | Females | 48.48 | 23.23 | 23.23 | 5.05 | 0.00 |
|  |  | Males | 46.97 | 21.97 | 25.00 | 2.27 | 3.79 |
|  |  | Non-binary gender |  |  |  |  |  |
|  |  | Total | 47.62 | 22.51 | 24.24 | 3.46 | 2.16 |
|  | C | Females | 58.95 | 18.95 | 20.00 | 0.00 | 2.11 |
|  |  | Males | 60.99 | 19.15 | 18.44 | 0.00 | 1.42 |
|  |  | Non-binary gender | 0.00 | 100.00 | 0.00 | 0.00 | 0.00 |
|  |  | Total | 59.92 | 19.41 | 18.99 | 0.00 | 1.69 |
| **Russia** | A | Females | 55.09 | 18.41 | 20.66 | 2.69 | 3.14 |
|  |  | Males | 54.48 | 20.90 | 18.66 | 3.73 | 2.24 |
|  |  | Non-binary gender | 50.00 | 12.50 | 25.00 | 6.25 | 6.25 |
|  |  | Total | 54.89 | 18.70 | 20.42 | 2.93 | 3.06 |
|  | B | Females | 48.44 | 18.75 | 22.66 | 7.03 | 3.13 |
|  |  | Males | 51.26 | 15.97 | 21.85 | 7.56 | 3.36 |
|  |  | Non-binary gender | 57.14 | 0.00 | 28.57 | 14.29 | 0.00 |
|  |  | Total | 50.00 | 16.93 | 22.44 | 7.48 | 3.15 |
|  | C | Females | 47.86 | 21.79 | 22.18 | 7.39 | 0.78 |
|  |  | Males | 47.13 | 18.39 | 20.69 | 10.34 | 3.45 |
|  |  | Non-binary gender | 77.78 | 11.11 | 11.11 | 0.00 | 0.00 |
|  |  | Total | 48.44 | 20.68 | 21.53 | 7.93 | 1.42 |
| **Malaysia** | A | Females | 70.73 | 15.85 | 7.32 | 3.66 | 2.44 |
|  |  | Males | 46.15 | 23.08 | 0.00 | 23.08 | 7.69 |
|  |  | Non-binary gender | 100.00 | 0.00 | 0.00 | 0.00 | 0.00 |
|  |  | Total | 67.71 | 16.67 | 6.25 | 6.25 | 3.13 |
|  | B | Females | 40.63 | 18.75 | 18.75 | 18.75 | 3.13 |
|  |  | Males | 60.00 | 20.00 | 10.00 | 10.00 | 0.00 |
|  |  | Non-binary gender |  |  |  |  |  |
|  |  | Total | 45.24 | 19.05 | 16.67 | 16.67 | 2.38 |
|  | C | Females | 30.77 | 29.23 | 10.77 | 21.54 | 7.69 |
|  |  | Males | 28.57 | 7.14 | 0.00 | 57.14 | 7.14 |
|  |  | Non-binary gender | 100.00 | 0.00 | 0.00 | 0.00 | 0.00 |
|  |  | Total | 31.25 | 25.00 | 8.75 | 27.50 | 7.50 |
| **Hungary** | A | Females | 56.25 | 25.00 | 6.25 | 6.25 | 6.25 |
|  |  | Males | 66.67 | 22.22 | 11.11 | 0.00 | 0.00 |
|  |  | Non-binary gender |  |  |  |  |  |
|  |  | Total | 58.54 | 24.39 | 7.32 | 4.88 | 4.88 |
|  | B | Females | 50.00 | 30.00 | 0.00 | 10.00 | 10.00 |
|  |  | Males | 50.00 | 25.00 | 25.00 | 0.00 | 0.00 |
|  |  | Non-binary gender |  |  |  |  |  |
|  |  | Total | 50.00 | 28.57 | 7.14 | 7.14 | 7.14 |
|  | C | Females | 44.83 | 37.93 | 3.45 | 10.34 | 3.45 |
|  |  | Males | 66.67 | 0.00 | 33.33 | 0.00 | 0.00 |
|  |  | Non-binary gender |  |  |  |  |  |
|  |  | Total | 46.88 | 34.38 | 6.25 | 9.38 | 3.13 |
| **TOTAL** |  |  | 47.61 | 23.83 | 17.77 | 6.32 | 4.48 |
| **J17. The government allows or commits acts of terrorism on its territory, disguising its involvement as if someone else is responsible.** | | | | | | | |
| **Bulgaria** | A | Females | 55.32 | 25.53 | 12.77 | 4.26 | 2.13 |
|  |  | Males | 60.00 | 24.44 | 8.89 | 4.44 | 2.22 |
|  |  | Non-binary gender | 50.00 | 50.00 | 0.00 | 0.00 | 0.00 |
|  |  | Total | 57.45 | 25.53 | 10.64 | 4.26 | 2.13 |
|  | B | Females | 35.71 | 8.93 | 30.36 | 12.50 | 12.50 |
|  |  | Males | 33.33 | 23.33 | 20.00 | 10.00 | 13.33 |
|  |  | Non-binary gender |  |  |  |  |  |
|  |  | Total | 34.88 | 13.95 | 26.74 | 11.63 | 12.79 |
|  | C | Females | 28.81 | 13.56 | 38.98 | 15.25 | 3.39 |
|  |  | Males | 44.44 | 25.93 | 22.22 | 7.41 | 0.00 |
|  |  | Non-binary gender |  |  |  |  |  |
|  |  | Total | 33.72 | 17.44 | 33.72 | 12.79 | 2.33 |
| **Croatia** | A | Females | 53.25 | 21.00 | 16.02 | 7.14 | 2.60 |
|  |  | Males | 61.25 | 15.00 | 11.25 | 7.50 | 5.00 |
|  |  | Non-binary gender | 0.00 | 100.00 | 0.00 | 0.00 | 0.00 |
|  |  | Total | 54.33 | 20.26 | 15.29 | 7.18 | 2.95 |
|  | B | Females | 44.98 | 18.78 | 19.00 | 8.08 | 9.17 |
|  |  | Males | 44.31 | 23.35 | 10.78 | 10.78 | 10.78 |
|  |  | Non-binary gender | 50.00 | 16.67 | 16.67 | 0.00 | 16.67 |
|  |  | Total | 44.85 | 19.97 | 16.80 | 8.72 | 9.67 |
|  | C | Females | 40.72 | 23.65 | 20.66 | 6.89 | 8.08 |
|  |  | Males | 44.00 | 20.00 | 16.00 | 14.00 | 6.00 |
|  |  | Non-binary gender | 50.00 | 50.00 | 0.00 | 0.00 | 0.00 |
|  |  | Total | 41.19 | 23.32 | 19.95 | 7.77 | 7.77 |
| **Georgia** | A | Females | 56.42 | 22.35 | 15.08 | 2.23 | 3.91 |
|  |  | Males | 49.69 | 22.70 | 14.72 | 6.13 | 6.75 |
|  |  | Non-binary gender | 100.00 | 0.00 | 0.00 | 0.00 | 0.00 |
|  |  | Total | 54.49 | 22.37 | 14.91 | 3.44 | 4.78 |
|  | B | Females | 48.32 | 28.19 | 14.09 | 4.70 | 4.70 |
|  |  | Males | 54.43 | 25.32 | 13.92 | 6.33 | 0.00 |
|  |  | Non-binary gender | 33.33 | 33.33 | 33.33 | 0.00 | 0.00 |
|  |  | Total | 50.22 | 27.27 | 14.29 | 5.19 | 3.03 |
|  | C | Females | 53.93 | 23.56 | 14.14 | 5.24 | 3.14 |
|  |  | Males | 57.89 | 23.68 | 5.26 | 7.89 | 5.26 |
|  |  | Non-binary gender | 0.00 | 0.00 | 0.00 | 0.00 | 100.00 |
|  |  | Total | 54.35 | 23.48 | 12.61 | 5.65 | 3.91 |
| **Greece** | A | Females | 40.77 | 25.93 | 16.48 | 11.32 | 5.49 |
|  |  | Males | 49.29 | 22.50 | 13.57 | 7.86 | 6.79 |
|  |  | Non-binary gender | 38.46 | 15.38 | 23.08 | 15.38 | 7.69 |
|  |  | Total | 42.73 | 25.02 | 15.88 | 10.56 | 5.82 |
|  | B | Females | 38.58 | 22.28 | 19.65 | 10.42 | 9.07 |
|  |  | Males | 47.07 | 20.66 | 13.94 | 10.51 | 7.82 |
|  |  | Non-binary gender | 22.22 | 48.15 | 14.81 | 3.70 | 11.11 |
|  |  | Total | 41.67 | 21.98 | 17.36 | 10.37 | 8.61 |
|  | C | Females | 34.35 | 22.52 | 21.39 | 12.79 | 8.94 |
|  |  | Males | 45.63 | 23.66 | 12.96 | 9.58 | 8.17 |
|  |  | Non-binary gender | 20.69 | 13.79 | 6.90 | 24.14 | 34.48 |
|  |  | Total | 36.03 | 22.59 | 19.80 | 12.41 | 9.16 |
| **India** | A | Females | 42.45 | 26.42 | 26.42 | 2.83 | 1.89 |
|  |  | Males | 41.03 | 28.21 | 20.51 | 7.69 | 2.56 |
|  |  | Non-binary gender |  |  |  |  |  |
|  |  | Total | 42.07 | 26.90 | 24.83 | 4.14 | 2.07 |
|  | B | Females | 36.84 | 19.30 | 24.56 | 13.16 | 6.14 |
|  |  | Males | 40.98 | 23.77 | 25.41 | 2.46 | 7.38 |
|  |  | Non-binary gender |  |  |  |  |  |
|  |  | Total | 38.98 | 21.61 | 25.00 | 7.63 | 6.78 |
|  | C | Females | 41.38 | 19.54 | 28.74 | 2.30 | 8.05 |
|  |  | Males | 38.46 | 17.31 | 26.92 | 3.85 | 13.46 |
|  |  | Non-binary gender | 0.00 | 0.00 | 0.00 | 100.00 | 0.00 |
|  |  | Total | 40.00 | 18.57 | 27.86 | 3.57 | 10.00 |
| **Latvia** | A | Females | 76.83 | 12.61 | 9.09 | 0.88 | 0.59 |
|  |  | Males | 72.50 | 5.00 | 22.50 | 0.00 | 0.00 |
|  |  | Non-binary gender | 100.00 | 0.00 | 0.00 | 0.00 | 0.00 |
|  |  | Total | 76.68 | 11.66 | 10.36 | 0.78 | 0.52 |
|  | B | Females | 62.56 | 14.29 | 18.72 | 2.96 | 1.48 |
|  |  | Males | 63.41 | 13.01 | 15.45 | 4.88 | 3.25 |
|  |  | Non-binary gender | 0.00 | 50.00 | 50.00 | 0.00 | 0.00 |
|  |  | Total | 62.50 | 14.02 | 17.68 | 3.66 | 2.13 |
|  | C | Females | 69.01 | 14.79 | 13.03 | 1.76 | 1.41 |
|  |  | Males | 61.22 | 14.29 | 14.29 | 2.04 | 8.16 |
|  |  | Non-binary gender |  |  |  |  |  |
|  |  | Total | 67.87 | 14.71 | 13.21 | 1.80 | 2.40 |
| **Lithuania** | A | Females | 81.91 | 10.64 | 4.26 | 2.13 | 1.06 |
|  |  | Males | 76.47 | 11.76 | 0.00 | 8.82 | 2.94 |
|  |  | Non-binary gender | 33.33 | 0.00 | 33.33 | 33.33 | 0.00 |
|  |  | Total | 80.44 | 10.67 | 4.00 | 3.56 | 1.33 |
|  | B | Females | 78.79 | 9.09 | 6.06 | 6.06 | 0.00 |
|  |  | Males | 75.00 | 12.50 | 12.50 | 0.00 | 0.00 |
|  |  | Non-binary gender |  |  |  |  |  |
|  |  | Total | 78.05 | 9.76 | 7.32 | 4.88 | 0.00 |
|  | C | Females | 82.14 | 7.14 | 7.14 | 3.57 | 0.00 |
|  |  | Males | 76.92 | 0.00 | 7.69 | 15.38 | 0.00 |
|  |  | Non-binary gender |  |  |  |  |  |
|  |  | Total | 81.16 | 5.80 | 7.25 | 5.80 | 0.00 |
| **Nigeria** | A | Females | 41.18 | 20.59 | 32.35 | 5.88 | 0.00 |
|  |  | Males | 49.02 | 25.49 | 17.65 | 7.84 | 0.00 |
|  |  | Non-binary gender |  |  |  |  |  |
|  |  | Total | 44.54 | 22.69 | 26.05 | 6.72 | 0.00 |
|  | B | Females | 43.43 | 23.23 | 27.27 | 5.05 | 1.01 |
|  |  | Males | 50.76 | 25.76 | 18.18 | 2.27 | 3.03 |
|  |  | Non-binary gender |  |  |  |  |  |
|  |  | Total | 47.62 | 24.68 | 22.08 | 3.46 | 2.16 |
|  | C | Females | 60.00 | 23.16 | 14.74 | 1.05 | 1.05 |
|  |  | Males | 47.52 | 29.79 | 21.28 | 0.00 | 1.42 |
|  |  | Non-binary gender | 100.00 | 0.00 | 0.00 | 0.00 | 0.00 |
|  |  | Total | 52.74 | 27.00 | 18.57 | 0.42 | 1.27 |
| **Russia** | A | Females | 54.79 | 16.62 | 20.66 | 4.49 | 3.44 |
|  |  | Males | 52.99 | 14.18 | 26.87 | 2.99 | 2.99 |
|  |  | Non-binary gender | 43.75 | 25.00 | 12.50 | 6.25 | 12.50 |
|  |  | Total | 54.28 | 16.38 | 21.52 | 4.28 | 3.55 |
|  | B | Females | 53.13 | 14.06 | 21.09 | 10.16 | 1.56 |
|  |  | Males | 47.90 | 18.49 | 19.33 | 10.08 | 4.20 |
|  |  | Non-binary gender | 57.14 | 14.29 | 28.57 | 0.00 | 0.00 |
|  |  | Total | 50.79 | 16.14 | 20.47 | 9.84 | 2.76 |
|  | C | Females | 49.42 | 18.68 | 22.96 | 5.84 | 3.11 |
|  |  | Males | 48.28 | 19.54 | 16.09 | 8.05 | 8.05 |
|  |  | Non-binary gender | 88.89 | 0.00 | 11.11 | 0.00 | 0.00 |
|  |  | Total | 50.14 | 18.41 | 20.96 | 6.23 | 4.25 |
| **Malaysia** | A | Females | 79.27 | 13.41 | 3.66 | 1.22 | 2.44 |
|  |  | Males | 53.85 | 23.08 | 7.69 | 15.38 | 0.00 |
|  |  | Non-binary gender | 100.00 | 0.00 | 0.00 | 0.00 | 0.00 |
|  |  | Total | 76.04 | 14.58 | 4.17 | 3.13 | 2.08 |
|  | B | Females | 40.63 | 21.88 | 9.38 | 25.00 | 3.13 |
|  |  | Males | 90.00 | 0.00 | 0.00 | 10.00 | 0.00 |
|  |  | Non-binary gender |  |  |  |  |  |
|  |  | Total | 52.38 | 16.67 | 7.14 | 21.43 | 2.38 |
|  | C | Females | 40.00 | 21.54 | 4.62 | 21.54 | 12.31 |
|  |  | Males | 28.57 | 7.14 | 7.14 | 50.00 | 7.14 |
|  |  | Non-binary gender | 100.00 | 0.00 | 0.00 | 0.00 | 0.00 |
|  |  | Total | 38.75 | 18.75 | 5.00 | 26.25 | 11.25 |
| **Hungary** | A | Females | 78.13 | 9.38 | 6.25 | 6.25 | 0.00 |
|  |  | Males | 100.00 | 0.00 | 0.00 | 0.00 | 0.00 |
|  |  | Non-binary gender |  |  |  |  |  |
|  |  | Total | 82.93 | 7.32 | 4.88 | 4.88 | 0.00 |
|  | B | Females | 60.00 | 30.00 | 10.00 | 0.00 | 0.00 |
|  |  | Males | 50.00 | 0.00 | 50.00 | 0.00 | 0.00 |
|  |  | Non-binary gender |  |  |  |  |  |
|  |  | Total | 57.14 | 21.43 | 21.43 | 0.00 | 0.00 |
|  | C | Females | 72.41 | 17.24 | 0.00 | 6.90 | 3.45 |
|  |  | Males | 33.33 | 33.33 | 0.00 | 0.00 | 33.33 |
|  |  | Non-binary gender |  |  |  |  |  |
|  |  | Total | 68.75 | 18.75 | 0.00 | 6.25 | 6.25 |
| **TOTAL** |  |  | 47.85 | 20.70 | 17.52 | 8.04 | 5.90 |
| **J18. Do you believe that secretly a chip will be included in the COVID-19 vaccine in order to mark people?** | | | | | | | |
| **Bulgaria** | A | Females | 80.85 | 6.38 | 10.64 | 0.00 | 2.13 |
|  |  | Males | 88.89 | 4.44 | 4.44 | 2.22 | 0.00 |
|  |  | Non-binary gender | 100.00 | 0.00 | 0.00 | 0.00 | 0.00 |
|  |  | Total | 85.11 | 5.32 | 7.45 | 1.06 | 1.06 |
|  | B | Females | 71.43 | 16.07 | 8.93 | 0.00 | 3.57 |
|  |  | Males | 66.67 | 16.67 | 10.00 | 6.67 | 0.00 |
|  |  | Non-binary gender |  |  |  |  |  |
|  |  | Total | 69.77 | 16.28 | 9.30 | 2.33 | 2.33 |
|  | C | Females | 44.07 | 8.47 | 33.90 | 8.47 | 5.08 |
|  |  | Males | 59.26 | 22.22 | 18.52 | 0.00 | 0.00 |
|  |  | Non-binary gender |  |  |  |  |  |
|  |  | Total | 48.84 | 12.79 | 29.07 | 5.81 | 3.49 |
| **Croatia** | A | Females | 78.79 | 9.31 | 8.23 | 2.38 | 1.30 |
|  |  | Males | 86.25 | 5.00 | 6.25 | 2.50 | 0.00 |
|  |  | Non-binary gender | 100.00 | 0.00 | 0.00 | 0.00 | 0.00 |
|  |  | Total | 79.93 | 8.66 | 7.92 | 2.39 | 1.10 |
|  | B | Females | 63.97 | 10.48 | 15.28 | 4.80 | 5.46 |
|  |  | Males | 73.65 | 11.38 | 6.59 | 2.99 | 5.39 |
|  |  | Non-binary gender | 83.33 | 16.67 | 0.00 | 0.00 | 0.00 |
|  |  | Total | 66.72 | 10.78 | 12.84 | 4.28 | 5.39 |
|  | C | Females | 71.86 | 9.58 | 10.48 | 3.29 | 4.79 |
|  |  | Males | 66.00 | 6.00 | 14.00 | 6.00 | 8.00 |
|  |  | Non-binary gender | 50.00 | 50.00 | 0.00 | 0.00 | 0.00 |
|  |  | Total | 70.98 | 9.33 | 10.88 | 3.63 | 5.18 |
| **Georgia** | A | Females | 73.74 | 10.34 | 11.17 | 2.51 | 2.23 |
|  |  | Males | 69.94 | 14.72 | 7.36 | 3.07 | 4.91 |
|  |  | Non-binary gender | 100.00 | 0.00 | 0.00 | 0.00 | 0.00 |
|  |  | Total | 72.66 | 11.66 | 9.94 | 2.68 | 3.06 |
|  | B | Females | 71.81 | 15.44 | 8.72 | 0.67 | 3.36 |
|  |  | Males | 75.95 | 10.13 | 13.92 | 0.00 | 0.00 |
|  |  | Non-binary gender | 66.67 | 0.00 | 33.33 | 0.00 | 0.00 |
|  |  | Total | 73.16 | 13.42 | 10.82 | 0.43 | 2.16 |
|  | C | Females | 71.73 | 10.99 | 10.99 | 3.66 | 2.62 |
|  |  | Males | 84.21 | 2.63 | 10.53 | 0.00 | 2.63 |
|  |  | Non-binary gender | 0.00 | 0.00 | 0.00 | 0.00 | 100.00 |
|  |  | Total | 73.48 | 9.57 | 10.87 | 3.04 | 3.04 |
| **Greece** | A | Females | 82.53 | 8.46 | 5.16 | 2.31 | 1.54 |
|  |  | Males | 87.14 | 5.36 | 5.00 | 1.79 | 0.71 |
|  |  | Non-binary gender | 61.54 | 15.38 | 15.38 | 7.69 | 0.00 |
|  |  | Total | 83.37 | 7.81 | 5.24 | 2.24 | 1.33 |
|  | B | Females | 82.34 | 7.64 | 6.28 | 2.07 | 1.67 |
|  |  | Males | 87.53 | 5.26 | 4.52 | 0.73 | 1.96 |
|  |  | Non-binary gender | 74.07 | 11.11 | 11.11 | 0.00 | 3.70 |
|  |  | Total | 84.25 | 6.76 | 5.66 | 1.52 | 1.81 |
|  | C | Females | 74.65 | 10.02 | 9.28 | 3.62 | 2.43 |
|  |  | Males | 79.44 | 10.14 | 6.48 | 1.41 | 2.54 |
|  |  | Non-binary gender | 86.21 | 6.90 | 6.90 | 0.00 | 0.00 |
|  |  | Total | 75.59 | 10.00 | 8.79 | 3.21 | 2.42 |
| **India** | A | Females | 47.17 | 22.64 | 26.42 | 2.83 | 0.94 |
|  |  | Males | 53.85 | 15.38 | 17.95 | 10.26 | 2.56 |
|  |  | Non-binary gender |  |  |  |  |  |
|  |  | Total | 48.97 | 20.69 | 24.14 | 4.83 | 1.38 |
|  | B | Females | 57.89 | 12.28 | 23.68 | 4.39 | 1.75 |
|  |  | Males | 69.67 | 7.38 | 18.03 | 3.28 | 1.64 |
|  |  | Non-binary gender |  |  |  |  |  |
|  |  | Total | 63.98 | 9.75 | 20.76 | 3.81 | 1.69 |
|  | C | Females | 62.07 | 11.49 | 22.99 | 2.30 | 1.15 |
|  |  | Males | 61.54 | 11.54 | 19.23 | 3.85 | 3.85 |
|  |  | Non-binary gender | 0.00 | 0.00 | 0.00 | 0.00 | 100.00 |
|  |  | Total | 61.43 | 11.43 | 21.43 | 2.86 | 2.86 |
| **Latvia** | A | Females | 89.74 | 4.69 | 4.40 | 0.29 | 0.88 |
|  |  | Males | 90.00 | 0.00 | 10.00 | 0.00 | 0.00 |
|  |  | Non-binary gender | 100.00 | 0.00 | 0.00 | 0.00 | 0.00 |
|  |  | Total | 89.90 | 4.15 | 4.92 | 0.26 | 0.78 |
|  | B | Females | 74.38 | 11.82 | 10.84 | 0.49 | 2.46 |
|  |  | Males | 79.67 | 4.07 | 10.57 | 3.25 | 2.44 |
|  |  | Non-binary gender | 0.00 | 50.00 | 50.00 | 0.00 | 0.00 |
|  |  | Total | 75.91 | 9.15 | 10.98 | 1.52 | 2.44 |
|  | C | Females | 77.82 | 6.69 | 10.92 | 3.52 | 1.06 |
|  |  | Males | 75.51 | 4.08 | 16.33 | 4.08 | 0.00 |
|  |  | Non-binary gender |  |  |  |  |  |
|  |  | Total | 77.48 | 6.31 | 11.71 | 3.60 | 0.90 |
| **Lithuania** | A | Females | 91.49 | 5.85 | 1.60 | 0.53 | 0.53 |
|  |  | Males | 97.06 | 0.00 | 0.00 | 0.00 | 2.94 |
|  |  | Non-binary gender | 66.67 | 33.33 | 0.00 | 0.00 | 0.00 |
|  |  | Total | 92.00 | 5.33 | 1.33 | 0.44 | 0.89 |
|  | B | Females | 72.73 | 9.09 | 6.06 | 6.06 | 6.06 |
|  |  | Males | 62.50 | 0.00 | 25.00 | 12.50 | 0.00 |
|  |  | Non-binary gender |  |  |  |  |  |
|  |  | Total | 70.73 | 7.32 | 9.76 | 7.32 | 4.88 |
|  | C | Females | 73.21 | 5.36 | 12.50 | 8.93 | 0.00 |
|  |  | Males | 76.92 | 7.69 | 7.69 | 0.00 | 7.69 |
|  |  | Non-binary gender |  |  |  |  |  |
|  |  | Total | 73.91 | 5.80 | 11.59 | 7.25 | 1.45 |
| **Nigeria** | A | Females | 52.94 | 17.65 | 27.94 | 1.47 | 0.00 |
|  |  | Males | 56.86 | 21.57 | 21.57 | 0.00 | 0.00 |
|  |  | Non-binary gender |  |  |  |  |  |
|  |  | Total | 54.62 | 19.33 | 25.21 | 0.84 | 0.00 |
|  | B | Females | 46.46 | 24.24 | 23.23 | 6.06 | 0.00 |
|  |  | Males | 48.48 | 22.73 | 22.73 | 3.79 | 2.27 |
|  |  | Non-binary gender |  |  |  |  |  |
|  |  | Total | 47.62 | 23.38 | 22.94 | 4.76 | 1.30 |
|  | C | Females | 63.16 | 21.05 | 13.68 | 1.05 | 1.05 |
|  |  | Males | 57.45 | 22.70 | 18.44 | 0.00 | 1.42 |
|  |  | Non-binary gender | 0.00 | 100.00 | 0.00 | 0.00 | 0.00 |
|  |  | Total | 59.49 | 22.36 | 16.46 | 0.42 | 1.27 |
| **Russia** | A | Females | 75.60 | 8.83 | 12.43 | 1.65 | 1.50 |
|  |  | Males | 73.13 | 11.94 | 11.19 | 2.99 | 0.75 |
|  |  | Non-binary gender | 62.50 | 12.50 | 18.75 | 6.25 | 0.00 |
|  |  | Total | 74.94 | 9.41 | 12.35 | 1.96 | 1.34 |
|  | B | Females | 63.28 | 10.94 | 16.41 | 3.13 | 6.25 |
|  |  | Males | 68.91 | 7.56 | 19.33 | 0.00 | 4.20 |
|  |  | Non-binary gender | 71.43 | 14.29 | 14.29 | 0.00 | 0.00 |
|  |  | Total | 66.14 | 9.45 | 17.72 | 1.57 | 5.12 |
|  | C | Females | 64.20 | 12.84 | 17.51 | 3.89 | 1.56 |
|  |  | Males | 72.41 | 6.90 | 12.64 | 5.75 | 2.30 |
|  |  | Non-binary gender | 88.89 | 0.00 | 11.11 | 0.00 | 0.00 |
|  |  | Total | 66.86 | 11.05 | 16.15 | 4.25 | 1.70 |
| **Malaysia** | A | Females | 87.80 | 12.20 | 0.00 | 0.00 | 0.00 |
|  |  | Males | 69.23 | 15.38 | 0.00 | 15.38 | 0.00 |
|  |  | Non-binary gender | 100.00 | 0.00 | 0.00 | 0.00 | 0.00 |
|  |  | Total | 85.42 | 12.50 | 0.00 | 2.08 | 0.00 |
|  | B | Females | 56.25 | 21.88 | 12.50 | 9.38 | 0.00 |
|  |  | Males | 70.00 | 20.00 | 0.00 | 10.00 | 0.00 |
|  |  | Non-binary gender |  |  |  |  |  |
|  |  | Total | 59.52 | 21.43 | 9.52 | 9.52 | 0.00 |
|  | C | Females | 53.85 | 18.46 | 6.15 | 16.92 | 4.62 |
|  |  | Males | 35.71 | 7.14 | 35.71 | 14.29 | 7.14 |
|  |  | Non-binary gender | 100.00 | 0.00 | 0.00 | 0.00 | 0.00 |
|  |  | Total | 51.25 | 16.25 | 11.25 | 16.25 | 5.00 |
| **Hungary** | A | Females | 96.88 | 0.00 | 3.13 | 0.00 | 0.00 |
|  |  | Males | 100.00 | 0.00 | 0.00 | 0.00 | 0.00 |
|  |  | Non-binary gender |  |  |  |  |  |
|  |  | Total | 97.56 | 0.00 | 2.44 | 0.00 | 0.00 |
|  | B | Females | 90.00 | 0.00 | 0.00 | 0.00 | 10.00 |
|  |  | Males | 100.00 | 0.00 | 0.00 | 0.00 | 0.00 |
|  |  | Non-binary gender |  |  |  |  |  |
|  |  | Total | 92.86 | 0.00 | 0.00 | 0.00 | 7.14 |
|  | C | Females | 96.55 | 0.00 | 3.45 | 0.00 | 0.00 |
|  |  | Males | 100.00 | 0.00 | 0.00 | 0.00 | 0.00 |
|  |  | Non-binary gender |  |  |  |  |  |
|  |  | Total | 96.88 | 0.00 | 3.13 | 0.00 | 0.00 |
| **TOTAL** |  |  | 75.63 | 9.65 | 9.95 | 2.61 | 2.16 |
| **J19. A small, secret group of people is responsible for taking all the important decisions, such as starting wars, in a planned way and the reasons are the group’s secret interests.** | | | | | | | |
| **Bulgaria** | A | Females | 46.81 | 12.77 | 23.40 | 6.38 | 10.64 |
|  |  | Males | 62.22 | 20.00 | 6.67 | 4.44 | 6.67 |
|  |  | Non-binary gender | 100.00 | 0.00 | 0.00 | 0.00 | 0.00 |
|  |  | Total | 55.32 | 15.96 | 14.89 | 5.32 | 8.51 |
|  | B | Females | 33.93 | 19.64 | 17.86 | 8.93 | 19.64 |
|  |  | Males | 36.67 | 6.67 | 20.00 | 16.67 | 20.00 |
|  |  | Non-binary gender |  |  |  |  |  |
|  |  | Total | 34.88 | 15.12 | 18.60 | 11.63 | 19.77 |
|  | C | Females | 20.34 | 15.25 | 32.20 | 13.56 | 18.64 |
|  |  | Males | 48.15 | 22.22 | 18.52 | 11.11 | 0.00 |
|  |  | Non-binary gender |  |  |  |  |  |
|  |  | Total | 29.07 | 17.44 | 27.91 | 12.79 | 12.79 |
| **Croatia** | A | Females | 47.84 | 22.29 | 16.23 | 6.71 | 6.93 |
|  |  | Males | 52.50 | 23.75 | 12.50 | 5.00 | 6.25 |
|  |  | Non-binary gender | 0.00 | 100.00 | 0.00 | 0.00 | 0.00 |
|  |  | Total | 48.43 | 22.65 | 15.65 | 6.45 | 6.81 |
|  | B | Females | 39.52 | 19.87 | 18.12 | 12.01 | 10.48 |
|  |  | Males | 44.91 | 17.37 | 17.96 | 8.38 | 11.38 |
|  |  | Non-binary gender | 50.00 | 16.67 | 33.33 | 0.00 | 0.00 |
|  |  | Total | 41.05 | 19.18 | 18.23 | 10.94 | 10.62 |
|  | C | Females | 38.92 | 22.75 | 18.26 | 9.88 | 10.18 |
|  |  | Males | 40.00 | 22.00 | 18.00 | 4.00 | 16.00 |
|  |  | Non-binary gender | 100.00 | 0.00 | 0.00 | 0.00 | 0.00 |
|  |  | Total | 39.38 | 22.54 | 18.13 | 9.07 | 10.88 |
| **Georgia** | A | Females | 48.04 | 24.30 | 17.32 | 5.03 | 5.31 |
|  |  | Males | 42.33 | 22.09 | 20.86 | 7.98 | 6.75 |
|  |  | Non-binary gender | 100.00 | 0.00 | 0.00 | 0.00 | 0.00 |
|  |  | Total | 46.46 | 23.52 | 18.36 | 5.93 | 5.74 |
|  | B | Females | 46.98 | 29.53 | 13.42 | 3.36 | 6.71 |
|  |  | Males | 46.84 | 22.78 | 17.72 | 5.06 | 7.59 |
|  |  | Non-binary gender | 33.33 | 0.00 | 66.67 | 0.00 | 0.00 |
|  |  | Total | 46.75 | 26.84 | 15.58 | 3.90 | 6.93 |
|  | C | Females | 49.74 | 20.94 | 15.18 | 8.38 | 5.76 |
|  |  | Males | 60.53 | 15.79 | 18.42 | 5.26 | 0.00 |
|  |  | Non-binary gender | 0.00 | 0.00 | 0.00 | 0.00 | 100.00 |
|  |  | Total | 51.30 | 20.00 | 15.65 | 7.83 | 5.22 |
| **Greece** | A | Females | 54.84 | 20.88 | 13.52 | 5.60 | 5.16 |
|  |  | Males | 57.14 | 17.50 | 11.07 | 7.50 | 6.79 |
|  |  | Non-binary gender | 30.77 | 46.15 | 7.69 | 7.69 | 7.69 |
|  |  | Total | 55.11 | 20.37 | 12.88 | 6.07 | 5.57 |
|  | B | Females | 51.47 | 21.00 | 15.83 | 6.28 | 5.41 |
|  |  | Males | 59.54 | 18.22 | 12.71 | 5.50 | 4.03 |
|  |  | Non-binary gender | 40.74 | 25.93 | 14.81 | 7.41 | 11.11 |
|  |  | Total | 54.47 | 19.98 | 14.61 | 5.99 | 4.95 |
|  | C | Females | 47.26 | 20.66 | 17.54 | 8.38 | 6.17 |
|  |  | Males | 49.01 | 20.28 | 16.06 | 9.86 | 4.79 |
|  |  | Non-binary gender | 51.72 | 27.59 | 17.24 | 3.45 | 0.00 |
|  |  | Total | 47.61 | 20.69 | 17.29 | 8.55 | 5.86 |
| **India** | A | Females | 36.79 | 26.42 | 29.25 | 4.72 | 2.83 |
|  |  | Males | 46.15 | 20.51 | 20.51 | 7.69 | 5.13 |
|  |  | Non-binary gender |  |  |  |  |  |
|  |  | Total | 39.31 | 24.83 | 26.90 | 5.52 | 3.45 |
|  | B | Females | 38.60 | 18.42 | 30.70 | 10.53 | 1.75 |
|  |  | Males | 38.52 | 19.67 | 30.33 | 4.92 | 6.56 |
|  |  | Non-binary gender |  |  |  |  |  |
|  |  | Total | 38.56 | 19.07 | 30.51 | 7.63 | 4.24 |
|  | C | Females | 43.68 | 21.84 | 25.29 | 8.05 | 1.15 |
|  |  | Males | 40.38 | 23.08 | 25.00 | 5.77 | 5.77 |
|  |  | Non-binary gender | 0.00 | 0.00 | 0.00 | 100.00 | 0.00 |
|  |  | Total | 42.14 | 22.14 | 25.00 | 7.86 | 2.86 |
| **Latvia** | A | Females | 69.21 | 16.42 | 12.90 | 0.88 | 0.59 |
|  |  | Males | 72.50 | 7.50 | 12.50 | 5.00 | 2.50 |
|  |  | Non-binary gender | 80.00 | 20.00 | 0.00 | 0.00 | 0.00 |
|  |  | Total | 69.69 | 15.54 | 12.69 | 1.30 | 0.78 |
|  | B | Females | 58.13 | 16.26 | 18.23 | 4.43 | 2.96 |
|  |  | Males | 59.35 | 17.89 | 18.70 | 3.25 | 0.81 |
|  |  | Non-binary gender | 100.00 | 0.00 | 0.00 | 0.00 | 0.00 |
|  |  | Total | 58.84 | 16.77 | 18.29 | 3.96 | 2.13 |
|  | C | Females | 59.51 | 21.13 | 13.38 | 3.52 | 2.46 |
|  |  | Males | 57.14 | 22.45 | 16.33 | 2.04 | 2.04 |
|  |  | Non-binary gender |  |  |  |  |  |
|  |  | Total | 59.16 | 21.32 | 13.81 | 3.30 | 2.40 |
| **Lithuania** | A | Females | 77.13 | 15.43 | 4.26 | 2.66 | 0.53 |
|  |  | Males | 76.47 | 11.76 | 5.88 | 2.94 | 2.94 |
|  |  | Non-binary gender | 33.33 | 0.00 | 0.00 | 33.33 | 33.33 |
|  |  | Total | 76.44 | 14.67 | 4.44 | 3.11 | 1.33 |
|  | B | Females | 69.70 | 6.06 | 18.18 | 0.00 | 6.06 |
|  |  | Males | 50.00 | 12.50 | 25.00 | 12.50 | 0.00 |
|  |  | Non-binary gender |  |  |  |  |  |
|  |  | Total | 65.85 | 7.32 | 19.51 | 2.44 | 4.88 |
|  | C | Females | 75.00 | 8.93 | 7.14 | 5.36 | 3.57 |
|  |  | Males | 76.92 | 0.00 | 15.38 | 7.69 | 0.00 |
|  |  | Non-binary gender |  |  |  |  |  |
|  |  | Total | 75.36 | 7.25 | 8.70 | 5.80 | 2.90 |
| **Nigeria** | A | Females | 48.53 | 20.59 | 26.47 | 2.94 | 1.47 |
|  |  | Males | 58.82 | 17.65 | 17.65 | 5.88 | 0.00 |
|  |  | Non-binary gender |  |  |  |  |  |
|  |  | Total | 52.94 | 19.33 | 22.69 | 4.20 | 0.84 |
|  | B | Females | 49.49 | 25.25 | 21.21 | 4.04 | 0.00 |
|  |  | Males | 46.21 | 30.30 | 16.67 | 4.55 | 2.27 |
|  |  | Non-binary gender |  |  |  |  |  |
|  |  | Total | 47.62 | 28.14 | 18.61 | 4.33 | 1.30 |
|  | C | Females | 62.11 | 21.05 | 14.74 | 1.05 | 1.05 |
|  |  | Males | 63.83 | 17.02 | 17.73 | 0.71 | 0.71 |
|  |  | Non-binary gender | 0.00 | 0.00 | 100.00 | 0.00 | 0.00 |
|  |  | Total | 62.87 | 18.57 | 16.88 | 0.84 | 0.84 |
| **Russia** | A | Females | 58.68 | 16.02 | 19.16 | 4.34 | 1.80 |
|  |  | Males | 57.46 | 20.15 | 17.16 | 2.99 | 2.24 |
|  |  | Non-binary gender | 56.25 | 31.25 | 0.00 | 0.00 | 12.50 |
|  |  | Total | 58.44 | 16.99 | 18.46 | 4.03 | 2.08 |
|  | B | Females | 48.44 | 23.44 | 18.75 | 3.91 | 5.47 |
|  |  | Males | 58.82 | 12.61 | 19.33 | 6.72 | 2.52 |
|  |  | Non-binary gender | 57.14 | 42.86 | 0.00 | 0.00 | 0.00 |
|  |  | Total | 53.54 | 18.90 | 18.50 | 5.12 | 3.94 |
|  | C | Females | 47.47 | 23.35 | 21.40 | 5.84 | 1.95 |
|  |  | Males | 56.32 | 12.64 | 19.54 | 9.20 | 2.30 |
|  |  | Non-binary gender | 88.89 | 0.00 | 11.11 | 0.00 | 0.00 |
|  |  | Total | 50.71 | 20.11 | 20.68 | 6.52 | 1.98 |
| **Malaysia** | A | Females | 69.51 | 18.29 | 3.66 | 4.88 | 3.66 |
|  |  | Males | 46.15 | 23.08 | 7.69 | 23.08 | 0.00 |
|  |  | Non-binary gender | 100.00 | 0.00 | 0.00 | 0.00 | 0.00 |
|  |  | Total | 66.67 | 18.75 | 4.17 | 7.29 | 3.13 |
|  | B | Females | 37.50 | 31.25 | 12.50 | 15.63 | 3.13 |
|  |  | Males | 40.00 | 40.00 | 10.00 | 10.00 | 0.00 |
|  |  | Non-binary gender |  |  |  |  |  |
|  |  | Total | 38.10 | 33.33 | 11.90 | 14.29 | 2.38 |
|  | C | Females | 38.46 | 18.46 | 16.92 | 20.00 | 6.15 |
|  |  | Males | 28.57 | 14.29 | 7.14 | 35.71 | 14.29 |
|  |  | Non-binary gender | 100.00 | 0.00 | 0.00 | 0.00 | 0.00 |
|  |  | Total | 37.50 | 17.50 | 15.00 | 22.50 | 7.50 |
| **Hungary** | A | Females | 75.00 | 9.38 | 3.13 | 9.38 | 3.13 |
|  |  | Males | 77.78 | 22.22 | 0.00 | 0.00 | 0.00 |
|  |  | Non-binary gender |  |  |  |  |  |
|  |  | Total | 75.61 | 12.20 | 2.44 | 7.32 | 2.44 |
|  | B | Females | 50.00 | 10.00 | 20.00 | 0.00 | 20.00 |
|  |  | Males | 50.00 | 25.00 | 25.00 | 0.00 | 0.00 |
|  |  | Non-binary gender |  |  |  |  |  |
|  |  | Total | 50.00 | 14.29 | 21.43 | 0.00 | 14.29 |
|  | C | Females | 58.62 | 17.24 | 13.79 | 6.90 | 3.45 |
|  |  | Males | 33.33 | 33.33 | 0.00 | 33.33 | 0.00 |
|  |  | Non-binary gender |  |  |  |  |  |
|  |  | Total | 56.25 | 18.75 | 12.50 | 9.38 | 3.13 |
| **TOTAL** |  |  | 51.90 | 20.05 | 16.51 | 6.46 | 5.09 |
| **J20. Technology and devices for mind control are used on people without their knowledge.** | | | | | | | |
| **Bulgaria** | A | Females | 65.96 | 8.51 | 14.89 | 10.64 | 0.00 |
|  |  | Males | 80.00 | 13.33 | 0.00 | 2.22 | 4.44 |
|  |  | Non-binary gender | 50.00 | 50.00 | 0.00 | 0.00 | 0.00 |
|  |  | Total | 72.34 | 11.70 | 7.45 | 6.38 | 2.13 |
|  | B | Females | 46.43 | 25.00 | 16.07 | 0.00 | 12.50 |
|  |  | Males | 56.67 | 20.00 | 13.33 | 6.67 | 3.33 |
|  |  | Non-binary gender |  |  |  |  |  |
|  |  | Total | 50.00 | 23.26 | 15.12 | 2.33 | 9.30 |
|  | C | Females | 28.81 | 8.47 | 32.20 | 16.95 | 13.56 |
|  |  | Males | 59.26 | 25.93 | 14.81 | 0.00 | 0.00 |
|  |  | Non-binary gender |  |  |  |  |  |
|  |  | Total | 38.37 | 13.95 | 26.74 | 11.63 | 9.30 |
| **Croatia** | A | Females | 66.88 | 14.29 | 10.82 | 4.55 | 3.46 |
|  |  | Males | 75.00 | 7.50 | 6.25 | 6.25 | 5.00 |
|  |  | Non-binary gender | 100.00 | 0.00 | 0.00 | 0.00 | 0.00 |
|  |  | Total | 68.14 | 13.26 | 10.13 | 4.79 | 3.68 |
|  | B | Females | 50.22 | 15.07 | 17.47 | 7.42 | 9.83 |
|  |  | Males | 64.67 | 15.57 | 9.58 | 6.59 | 3.59 |
|  |  | Non-binary gender | 66.67 | 0.00 | 33.33 | 0.00 | 0.00 |
|  |  | Total | 54.20 | 15.06 | 15.53 | 7.13 | 8.08 |
|  | C | Females | 53.89 | 16.77 | 17.37 | 5.99 | 5.99 |
|  |  | Males | 66.00 | 14.00 | 18.00 | 2.00 | 0.00 |
|  |  | Non-binary gender | 50.00 | 50.00 | 0.00 | 0.00 | 0.00 |
|  |  | Total | 55.44 | 16.58 | 17.36 | 5.44 | 5.18 |
| **Georgia** | A | Females | 64.80 | 13.13 | 13.69 | 5.03 | 3.35 |
|  |  | Males | 57.67 | 18.40 | 14.11 | 5.52 | 4.29 |
|  |  | Non-binary gender | 100.00 | 0.00 | 0.00 | 0.00 | 0.00 |
|  |  | Total | 62.72 | 14.72 | 13.77 | 5.16 | 3.63 |
|  | B | Females | 63.09 | 18.12 | 10.07 | 4.70 | 4.03 |
|  |  | Males | 67.09 | 13.92 | 15.19 | 1.27 | 2.53 |
|  |  | Non-binary gender | 33.33 | 33.33 | 33.33 | 0.00 | 0.00 |
|  |  | Total | 64.07 | 16.88 | 12.12 | 3.46 | 3.46 |
|  | C | Females | 62.83 | 14.66 | 14.14 | 3.14 | 5.24 |
|  |  | Males | 78.95 | 7.89 | 7.89 | 2.63 | 2.63 |
|  |  | Non-binary gender | 0.00 | 0.00 | 0.00 | 0.00 | 100.00 |
|  |  | Total | 65.22 | 13.48 | 13.04 | 3.04 | 5.22 |
| **Greece** | A | Females | 65.82 | 15.60 | 10.00 | 5.27 | 3.30 |
|  |  | Males | 76.79 | 10.71 | 8.57 | 2.50 | 1.43 |
|  |  | Non-binary gender | 53.85 | 15.38 | 30.77 | 0.00 | 0.00 |
|  |  | Total | 68.25 | 14.46 | 9.89 | 4.57 | 2.83 |
|  | B | Females | 63.96 | 15.04 | 10.90 | 6.13 | 3.98 |
|  |  | Males | 75.67 | 11.00 | 7.21 | 3.18 | 2.93 |
|  |  | Non-binary gender | 62.96 | 3.70 | 14.81 | 11.11 | 7.41 |
|  |  | Total | 68.51 | 13.32 | 9.51 | 5.04 | 3.62 |
|  | C | Females | 59.37 | 14.60 | 15.22 | 6.40 | 4.41 |
|  |  | Males | 64.79 | 17.18 | 9.58 | 5.35 | 3.10 |
|  |  | Non-binary gender | 62.07 | 24.14 | 3.45 | 3.45 | 6.90 |
|  |  | Total | 60.30 | 15.16 | 14.13 | 6.18 | 4.23 |
| **India** | A | Females | 43.40 | 25.47 | 23.58 | 4.72 | 2.83 |
|  |  | Males | 46.15 | 15.38 | 23.08 | 7.69 | 7.69 |
|  |  | Non-binary gender |  |  |  |  |  |
|  |  | Total | 44.14 | 22.76 | 23.45 | 5.52 | 4.14 |
|  | B | Females | 40.35 | 15.79 | 26.32 | 14.04 | 3.51 |
|  |  | Males | 43.44 | 19.67 | 28.69 | 4.92 | 3.28 |
|  |  | Non-binary gender |  |  |  |  |  |
|  |  | Total | 41.95 | 17.80 | 27.54 | 9.32 | 3.39 |
|  | C | Females | 48.28 | 18.39 | 22.99 | 5.75 | 4.60 |
|  |  | Males | 40.38 | 23.08 | 19.23 | 11.54 | 5.77 |
|  |  | Non-binary gender | 0.00 | 0.00 | 100.00 | 0.00 | 0.00 |
|  |  | Total | 45.00 | 20.00 | 22.14 | 7.86 | 5.00 |
| **Latvia** | A | Females | 68.04 | 14.96 | 11.73 | 4.11 | 1.17 |
|  |  | Males | 85.00 | 7.50 | 7.50 | 0.00 | 0.00 |
|  |  | Non-binary gender | 80.00 | 20.00 | 0.00 | 0.00 | 0.00 |
|  |  | Total | 69.95 | 14.25 | 11.14 | 3.63 | 1.04 |
|  | B | Females | 50.25 | 23.15 | 16.26 | 6.40 | 3.94 |
|  |  | Males | 67.48 | 9.76 | 16.26 | 4.07 | 2.44 |
|  |  | Non-binary gender | 50.00 | 0.00 | 0.00 | 50.00 | 0.00 |
|  |  | Total | 56.71 | 17.99 | 16.16 | 5.79 | 3.35 |
|  | C | Females | 53.87 | 17.25 | 19.01 | 6.34 | 3.52 |
|  |  | Males | 59.18 | 18.37 | 18.37 | 0.00 | 4.08 |
|  |  | Non-binary gender |  |  |  |  |  |
|  |  | Total | 54.65 | 17.42 | 18.92 | 5.41 | 3.60 |
| **Lithuania** | A | Females | 82.98 | 10.64 | 3.72 | 1.60 | 1.06 |
|  |  | Males | 91.18 | 2.94 | 0.00 | 2.94 | 2.94 |
|  |  | Non-binary gender | 66.67 | 0.00 | 33.33 | 0.00 | 0.00 |
|  |  | Total | 84.00 | 9.33 | 3.56 | 1.78 | 1.33 |
|  | B | Females | 75.76 | 6.06 | 6.06 | 6.06 | 6.06 |
|  |  | Males | 25.00 | 50.00 | 12.50 | 12.50 | 0.00 |
|  |  | Non-binary gender |  |  |  |  |  |
|  |  | Total | 65.85 | 14.63 | 7.32 | 7.32 | 4.88 |
|  | C | Females | 73.21 | 10.71 | 10.71 | 3.57 | 1.79 |
|  |  | Males | 76.92 | 0.00 | 23.08 | 0.00 | 0.00 |
|  |  | Non-binary gender |  |  |  |  |  |
|  |  | Total | 73.91 | 8.70 | 13.04 | 2.90 | 1.45 |
| **Nigeria** | A | Females | 48.53 | 25.00 | 25.00 | 1.47 | 0.00 |
|  |  | Males | 60.78 | 23.53 | 9.80 | 5.88 | 0.00 |
|  |  | Non-binary gender |  |  |  |  |  |
|  |  | Total | 53.78 | 24.37 | 18.49 | 3.36 | 0.00 |
|  | B | Females | 48.48 | 28.28 | 21.21 | 1.01 | 1.01 |
|  |  | Males | 46.21 | 26.52 | 22.73 | 3.79 | 0.76 |
|  |  | Non-binary gender |  |  |  |  |  |
|  |  | Total | 47.19 | 27.27 | 22.08 | 2.60 | 0.87 |
|  | C | Females | 61.05 | 16.84 | 18.95 | 2.11 | 1.05 |
|  |  | Males | 60.28 | 19.15 | 19.15 | 0.71 | 0.71 |
|  |  | Non-binary gender | 0.00 | 0.00 | 0.00 | 100.00 | 0.00 |
|  |  | Total | 60.34 | 18.14 | 18.99 | 1.69 | 0.84 |
| **Russia** | A | Females | 58.38 | 15.72 | 17.96 | 4.19 | 3.74 |
|  |  | Males | 58.96 | 14.93 | 19.40 | 2.99 | 3.73 |
|  |  | Non-binary gender | 37.50 | 18.75 | 18.75 | 18.75 | 6.25 |
|  |  | Total | 58.07 | 15.65 | 18.22 | 4.28 | 3.79 |
|  | B | Females | 45.31 | 19.53 | 19.53 | 8.59 | 7.03 |
|  |  | Males | 57.14 | 14.29 | 18.49 | 4.20 | 5.88 |
|  |  | Non-binary gender | 57.14 | 14.29 | 14.29 | 14.29 | 0.00 |
|  |  | Total | 51.18 | 16.93 | 18.90 | 6.69 | 6.30 |
|  | C | Females | 52.14 | 14.40 | 18.29 | 10.12 | 5.06 |
|  |  | Males | 57.47 | 14.94 | 16.09 | 5.75 | 5.75 |
|  |  | Non-binary gender | 88.89 | 0.00 | 11.11 | 0.00 | 0.00 |
|  |  | Total | 54.39 | 14.16 | 17.56 | 8.78 | 5.10 |
| **Malaysia** | A | Females | 71.95 | 23.17 | 2.44 | 2.44 | 0.00 |
|  |  | Males | 61.54 | 15.38 | 0.00 | 23.08 | 0.00 |
|  |  | Non-binary gender | 100.00 | 0.00 | 0.00 | 0.00 | 0.00 |
|  |  | Total | 70.83 | 21.88 | 2.08 | 5.21 | 0.00 |
|  | B | Females | 43.75 | 21.88 | 12.50 | 21.88 | 0.00 |
|  |  | Males | 60.00 | 20.00 | 0.00 | 20.00 | 0.00 |
|  |  | Non-binary gender |  |  |  |  |  |
|  |  | Total | 47.62 | 21.43 | 9.52 | 21.43 | 0.00 |
|  | C | Females | 52.31 | 18.46 | 3.08 | 21.54 | 4.62 |
|  |  | Males | 28.57 | 7.14 | 14.29 | 42.86 | 7.14 |
|  |  | Non-binary gender | 100.00 | 0.00 | 0.00 | 0.00 | 0.00 |
|  |  | Total | 48.75 | 16.25 | 5.00 | 25.00 | 5.00 |
| **Hungary** | A | Females | 84.38 | 6.25 | 3.13 | 0.00 | 6.25 |
|  |  | Males | 100.00 | 0.00 | 0.00 | 0.00 | 0.00 |
|  |  | Non-binary gender |  |  |  |  |  |
|  |  | Total | 87.80 | 4.88 | 2.44 | 0.00 | 4.88 |
|  | B | Females | 80.00 | 10.00 | 0.00 | 0.00 | 10.00 |
|  |  | Males | 75.00 | 25.00 | 0.00 | 0.00 | 0.00 |
|  |  | Non-binary gender |  |  |  |  |  |
|  |  | Total | 78.57 | 14.29 | 0.00 | 0.00 | 7.14 |
|  | C | Females | 68.97 | 13.79 | 6.90 | 10.34 | 0.00 |
|  |  | Males | 33.33 | 66.67 | 0.00 | 0.00 | 0.00 |
|  |  | Non-binary gender |  |  |  |  |  |
|  |  | Total | 65.63 | 18.75 | 6.25 | 9.38 | 0.00 |
| **TOTAL** |  |  | 61.61 | 15.36 | 13.74 | 5.45 | 3.84 |
| **J21. New and advanced technology that will make the existing industry obsolete is being suppressed in a malicious and violent way.** | | | | | | | |
| **Bulgaria** | A | Females | 53.19 | 14.89 | 14.89 | 10.64 | 6.38 |
|  |  | Males | 75.56 | 6.67 | 4.44 | 6.67 | 6.67 |
|  |  | Non-binary gender | 50.00 | 50.00 | 0.00 | 0.00 | 0.00 |
|  |  | Total | 63.83 | 11.70 | 9.57 | 8.51 | 6.38 |
|  | B | Females | 41.07 | 23.21 | 10.71 | 10.71 | 14.29 |
|  |  | Males | 40.00 | 30.00 | 20.00 | 3.33 | 6.67 |
|  |  | Non-binary gender |  |  |  |  |  |
|  |  | Total | 40.70 | 25.58 | 13.95 | 8.14 | 11.63 |
|  | C | Females | 32.20 | 13.56 | 38.98 | 8.47 | 6.78 |
|  |  | Males | 44.44 | 29.63 | 18.52 | 3.70 | 3.70 |
|  |  | Non-binary gender |  |  |  |  |  |
|  |  | Total | 36.05 | 18.60 | 32.56 | 6.98 | 5.81 |
| **Croatia** | A | Females | 49.78 | 24.89 | 16.02 | 6.28 | 3.03 |
|  |  | Males | 55.00 | 21.25 | 16.25 | 3.75 | 3.75 |
|  |  | Non-binary gender | 100.00 | 0.00 | 0.00 | 0.00 | 0.00 |
|  |  | Total | 50.64 | 24.31 | 16.02 | 5.89 | 3.13 |
|  | B | Females | 39.30 | 21.62 | 19.43 | 10.04 | 9.61 |
|  |  | Males | 46.11 | 20.96 | 19.16 | 5.39 | 8.38 |
|  |  | Non-binary gender | 33.33 | 16.67 | 50.00 | 0.00 | 0.00 |
|  |  | Total | 41.05 | 21.39 | 19.65 | 8.72 | 9.19 |
|  | C | Females | 37.72 | 27.84 | 21.26 | 8.68 | 4.49 |
|  |  | Males | 40.00 | 32.00 | 12.00 | 8.00 | 8.00 |
|  |  | Non-binary gender | 100.00 | 0.00 | 0.00 | 0.00 | 0.00 |
|  |  | Total | 38.34 | 28.24 | 19.95 | 8.55 | 4.92 |
| **Georgia** | A | Females | 56.15 | 19.27 | 16.48 | 5.31 | 2.79 |
|  |  | Males | 48.47 | 23.93 | 20.25 | 3.68 | 3.68 |
|  |  | Non-binary gender | 50.00 | 50.00 | 0.00 | 0.00 | 0.00 |
|  |  | Total | 53.73 | 20.84 | 17.59 | 4.78 | 3.06 |
|  | B | Females | 59.06 | 22.15 | 12.08 | 2.01 | 4.70 |
|  |  | Males | 55.70 | 18.99 | 20.25 | 2.53 | 2.53 |
|  |  | Non-binary gender | 33.33 | 33.33 | 33.33 | 0.00 | 0.00 |
|  |  | Total | 57.58 | 21.21 | 15.15 | 2.16 | 3.90 |
|  | C | Females | 59.69 | 19.37 | 8.90 | 7.85 | 4.19 |
|  |  | Males | 68.42 | 15.79 | 10.53 | 5.26 | 0.00 |
|  |  | Non-binary gender | 0.00 | 0.00 | 0.00 | 0.00 | 100.00 |
|  |  | Total | 60.87 | 18.70 | 9.13 | 7.39 | 3.91 |
| **Greece** | A | Females | 60.99 | 18.90 | 14.84 | 3.85 | 1.43 |
|  |  | Males | 60.00 | 20.71 | 11.79 | 5.00 | 2.50 |
|  |  | Non-binary gender | 46.15 | 38.46 | 7.69 | 0.00 | 7.69 |
|  |  | Total | 60.60 | 19.53 | 14.05 | 4.07 | 1.75 |
|  | B | Females | 56.80 | 21.56 | 14.80 | 4.22 | 2.63 |
|  |  | Males | 60.39 | 19.68 | 11.86 | 5.01 | 3.06 |
|  |  | Non-binary gender | 48.15 | 22.22 | 25.93 | 0.00 | 3.70 |
|  |  | Total | 58.09 | 20.84 | 13.80 | 4.47 | 2.81 |
|  | C | Females | 52.46 | 21.73 | 19.19 | 4.41 | 2.21 |
|  |  | Males | 53.52 | 23.94 | 15.49 | 5.07 | 1.97 |
|  |  | Non-binary gender | 65.52 | 24.14 | 3.45 | 6.90 | 0.00 |
|  |  | Total | 52.81 | 22.13 | 18.36 | 4.56 | 2.14 |
| **India** | A | Females | 39.62 | 24.53 | 28.30 | 4.72 | 2.83 |
|  |  | Males | 38.46 | 17.95 | 28.21 | 7.69 | 7.69 |
|  |  | Non-binary gender |  |  |  |  |  |
|  |  | Total | 39.31 | 22.76 | 28.28 | 5.52 | 4.14 |
|  | B | Females | 31.58 | 22.81 | 34.21 | 7.89 | 3.51 |
|  |  | Males | 37.70 | 21.31 | 32.79 | 6.56 | 1.64 |
|  |  | Non-binary gender |  |  |  |  |  |
|  |  | Total | 34.75 | 22.03 | 33.47 | 7.20 | 2.54 |
|  | C | Females | 37.93 | 24.14 | 28.74 | 4.60 | 4.60 |
|  |  | Males | 42.31 | 25.00 | 19.23 | 7.69 | 5.77 |
|  |  | Non-binary gender | 0.00 | 100.00 | 0.00 | 0.00 | 0.00 |
|  |  | Total | 39.29 | 25.00 | 25.00 | 5.71 | 5.00 |
| **Latvia** | A | Females | 71.85 | 12.61 | 13.78 | 1.76 | 0.00 |
|  |  | Males | 67.50 | 17.50 | 12.50 | 0.00 | 2.50 |
|  |  | Non-binary gender | 80.00 | 20.00 | 0.00 | 0.00 | 0.00 |
|  |  | Total | 71.50 | 13.21 | 13.47 | 1.55 | 0.26 |
|  | B | Females | 68.47 | 13.79 | 13.79 | 1.97 | 1.97 |
|  |  | Males | 60.98 | 19.51 | 12.20 | 5.69 | 1.63 |
|  |  | Non-binary gender | 50.00 | 0.00 | 0.00 | 50.00 | 0.00 |
|  |  | Total | 65.55 | 15.85 | 13.11 | 3.66 | 1.83 |
|  | C | Females | 65.14 | 18.31 | 12.68 | 3.52 | 0.35 |
|  |  | Males | 63.27 | 8.16 | 20.41 | 2.04 | 6.12 |
|  |  | Non-binary gender |  |  |  |  |  |
|  |  | Total | 64.86 | 16.82 | 13.81 | 3.30 | 1.20 |
| **Lithuania** | A | Females | 80.85 | 10.64 | 6.91 | 1.60 | 0.00 |
|  |  | Males | 79.41 | 8.82 | 11.76 | 0.00 | 0.00 |
|  |  | Non-binary gender | 33.33 | 0.00 | 0.00 | 66.67 | 0.00 |
|  |  | Total | 80.00 | 10.22 | 7.56 | 2.22 | 0.00 |
|  | B | Females | 72.73 | 6.06 | 15.15 | 0.00 | 6.06 |
|  |  | Males | 25.00 | 25.00 | 25.00 | 25.00 | 0.00 |
|  |  | Non-binary gender |  |  |  |  |  |
|  |  | Total | 63.41 | 9.76 | 17.07 | 4.88 | 4.88 |
|  | C | Females | 75.00 | 12.50 | 10.71 | 1.79 | 0.00 |
|  |  | Males | 76.92 | 7.69 | 15.38 | 0.00 | 0.00 |
|  |  | Non-binary gender |  |  |  |  |  |
|  |  | Total | 75.36 | 11.59 | 11.59 | 1.45 | 0.00 |
| **Nigeria** | A | Females | 51.47 | 20.59 | 27.94 | 0.00 | 0.00 |
|  |  | Males | 52.94 | 27.45 | 13.73 | 5.88 | 0.00 |
|  |  | Non-binary gender |  |  |  |  |  |
|  |  | Total | 52.10 | 23.53 | 21.85 | 2.52 | 0.00 |
|  | B | Females | 54.55 | 26.26 | 16.16 | 2.02 | 1.01 |
|  |  | Males | 51.52 | 23.48 | 20.45 | 3.79 | 0.76 |
|  |  | Non-binary gender |  |  |  |  |  |
|  |  | Total | 52.81 | 24.68 | 18.61 | 3.03 | 0.87 |
|  | C | Females | 64.21 | 14.74 | 17.89 | 3.16 | 0.00 |
|  |  | Males | 62.41 | 19.86 | 16.31 | 0.71 | 0.71 |
|  |  | Non-binary gender | 0.00 | 0.00 | 0.00 | 100.00 | 0.00 |
|  |  | Total | 62.87 | 17.72 | 16.88 | 2.11 | 0.42 |
| **Russia** | A | Females | 55.09 | 18.26 | 20.06 | 3.74 | 2.84 |
|  |  | Males | 55.22 | 17.16 | 23.13 | 1.49 | 2.99 |
|  |  | Non-binary gender | 43.75 | 12.50 | 31.25 | 12.50 | 0.00 |
|  |  | Total | 54.89 | 17.97 | 20.78 | 3.55 | 2.81 |
|  | B | Females | 46.88 | 24.22 | 21.09 | 6.25 | 1.56 |
|  |  | Males | 39.50 | 27.73 | 22.69 | 5.04 | 5.04 |
|  |  | Non-binary gender | 42.86 | 28.57 | 0.00 | 28.57 | 0.00 |
|  |  | Total | 43.31 | 25.98 | 21.26 | 6.30 | 3.15 |
|  | C | Females | 49.81 | 21.79 | 19.46 | 7.39 | 1.56 |
|  |  | Males | 51.72 | 17.24 | 19.54 | 8.05 | 3.45 |
|  |  | Non-binary gender | 66.67 | 11.11 | 22.22 | 0.00 | 0.00 |
|  |  | Total | 50.71 | 20.40 | 19.55 | 7.37 | 1.98 |
| **Malaysia** | A | Females | 63.41 | 25.61 | 3.66 | 4.88 | 2.44 |
|  |  | Males | 53.85 | 23.08 | 0.00 | 23.08 | 0.00 |
|  |  | Non-binary gender | 100.00 | 0.00 | 0.00 | 0.00 | 0.00 |
|  |  | Total | 62.50 | 25.00 | 3.13 | 7.29 | 2.08 |
|  | B | Females | 40.63 | 31.25 | 9.38 | 18.75 | 0.00 |
|  |  | Males | 40.00 | 40.00 | 0.00 | 20.00 | 0.00 |
|  |  | Non-binary gender |  |  |  |  |  |
|  |  | Total | 40.48 | 33.33 | 7.14 | 19.05 | 0.00 |
|  | C | Females | 35.38 | 27.69 | 10.77 | 21.54 | 4.62 |
|  |  | Males | 28.57 | 0.00 | 21.43 | 42.86 | 7.14 |
|  |  | Non-binary gender | 100.00 | 0.00 | 0.00 | 0.00 | 0.00 |
|  |  | Total | 35.00 | 22.50 | 12.50 | 25.00 | 5.00 |
| **Hungary** | A | Females | 71.88 | 15.63 | 9.38 | 3.13 | 0.00 |
|  |  | Males | 66.67 | 11.11 | 11.11 | 11.11 | 0.00 |
|  |  | Non-binary gender |  |  |  |  |  |
|  |  | Total | 70.73 | 14.63 | 9.76 | 4.88 | 0.00 |
|  | B | Females | 80.00 | 10.00 | 10.00 | 0.00 | 0.00 |
|  |  | Males | 50.00 | 0.00 | 25.00 | 0.00 | 25.00 |
|  |  | Non-binary gender |  |  |  |  |  |
|  |  | Total | 71.43 | 7.14 | 14.29 | 0.00 | 7.14 |
|  | C | Females | 65.52 | 24.14 | 3.45 | 3.45 | 3.45 |
|  |  | Males | 33.33 | 33.33 | 33.33 | 0.00 | 0.00 |
|  |  | Non-binary gender |  |  |  |  |  |
|  |  | Total | 62.50 | 25.00 | 6.25 | 3.13 | 3.13 |
| **TOTAL** |  |  | 54.80 | 20.60 | 16.76 | 4.99 | 2.85 |
| **J22. Some important events happen due to the activity of a small group who secretly manipulate world events.** | | | | | | | |
| **Bulgaria** | A | Females | 46.81 | 14.89 | 21.28 | 10.64 | 6.38 |
|  |  | Males | 55.56 | 22.22 | 8.89 | 4.44 | 8.89 |
|  |  | Non-binary gender | 100.00 | 0.00 | 0.00 | 0.00 | 0.00 |
|  |  | Total | 52.13 | 18.09 | 14.89 | 7.45 | 7.45 |
|  | B | Females | 39.29 | 19.64 | 16.07 | 8.93 | 16.07 |
|  |  | Males | 36.67 | 20.00 | 20.00 | 3.33 | 20.00 |
|  |  | Non-binary gender |  |  |  |  |  |
|  |  | Total | 38.37 | 19.77 | 17.44 | 6.98 | 17.44 |
|  | C | Females | 20.34 | 15.25 | 40.68 | 11.86 | 11.86 |
|  |  | Males | 48.15 | 11.11 | 25.93 | 14.81 | 0.00 |
|  |  | Non-binary gender |  |  |  |  |  |
|  |  | Total | 29.07 | 13.95 | 36.05 | 12.79 | 8.14 |
| **Croatia** | A | Females | 47.19 | 22.51 | 19.05 | 5.41 | 5.84 |
|  |  | Males | 51.25 | 22.50 | 12.50 | 7.50 | 6.25 |
|  |  | Non-binary gender | 0.00 | 100.00 | 0.00 | 0.00 | 0.00 |
|  |  | Total | 47.70 | 22.65 | 18.05 | 5.71 | 5.89 |
|  | B | Females | 38.86 | 21.18 | 20.52 | 9.39 | 10.04 |
|  |  | Males | 40.72 | 23.35 | 15.57 | 10.18 | 10.18 |
|  |  | Non-binary gender | 50.00 | 16.67 | 33.33 | 0.00 | 0.00 |
|  |  | Total | 39.46 | 21.71 | 19.33 | 9.51 | 9.98 |
|  | C | Females | 38.02 | 22.75 | 20.96 | 10.18 | 8.08 |
|  |  | Males | 44.00 | 26.00 | 12.00 | 4.00 | 14.00 |
|  |  | Non-binary gender | 100.00 | 0.00 | 0.00 | 0.00 | 0.00 |
|  |  | Total | 39.12 | 23.06 | 19.69 | 9.33 | 8.81 |
| **Georgia** | A | Females | 47.21 | 22.35 | 17.32 | 6.70 | 6.42 |
|  |  | Males | 42.33 | 23.93 | 23.31 | 3.68 | 6.75 |
|  |  | Non-binary gender | 50.00 | 50.00 | 0.00 | 0.00 | 0.00 |
|  |  | Total | 45.70 | 22.94 | 19.12 | 5.74 | 6.50 |
|  | B | Females | 47.65 | 28.19 | 13.42 | 5.37 | 5.37 |
|  |  | Males | 45.57 | 22.78 | 21.52 | 3.80 | 6.33 |
|  |  | Non-binary gender | 33.33 | 33.33 | 33.33 | 0.00 | 0.00 |
|  |  | Total | 46.75 | 26.41 | 16.45 | 4.76 | 5.63 |
|  | C | Females | 50.79 | 20.94 | 13.61 | 9.42 | 5.24 |
|  |  | Males | 52.63 | 31.58 | 7.89 | 5.26 | 2.63 |
|  |  | Non-binary gender | 0.00 | 0.00 | 0.00 | 0.00 | 100.00 |
|  |  | Total | 50.87 | 22.61 | 12.61 | 8.70 | 5.22 |
| **Greece** | A | Females | 51.10 | 22.20 | 16.48 | 5.93 | 4.29 |
|  |  | Males | 54.29 | 17.50 | 13.21 | 7.14 | 7.86 |
|  |  | Non-binary gender | 38.46 | 23.08 | 23.08 | 7.69 | 7.69 |
|  |  | Total | 51.70 | 21.11 | 15.79 | 6.23 | 5.15 |
|  | B | Females | 50.36 | 22.59 | 15.51 | 6.68 | 4.85 |
|  |  | Males | 57.21 | 19.93 | 12.35 | 5.87 | 4.65 |
|  |  | Non-binary gender | 48.15 | 18.52 | 14.81 | 7.41 | 11.11 |
|  |  | Total | 53.00 | 21.50 | 14.27 | 6.37 | 4.85 |
|  | C | Females | 46.18 | 22.47 | 18.05 | 9.11 | 4.19 |
|  |  | Males | 44.23 | 21.97 | 20.28 | 7.32 | 6.20 |
|  |  | Non-binary gender | 55.17 | 27.59 | 17.24 | 0.00 | 0.00 |
|  |  | Total | 45.98 | 22.45 | 18.41 | 8.69 | 4.46 |
| **India** | A | Females | 39.62 | 25.47 | 26.42 | 5.66 | 2.83 |
|  |  | Males | 38.46 | 23.08 | 17.95 | 15.38 | 5.13 |
|  |  | Non-binary gender |  |  |  |  |  |
|  |  | Total | 39.31 | 24.83 | 24.14 | 8.28 | 3.45 |
|  | B | Females | 35.96 | 20.18 | 29.82 | 12.28 | 1.75 |
|  |  | Males | 36.07 | 20.49 | 32.79 | 7.38 | 3.28 |
|  |  | Non-binary gender |  |  |  |  |  |
|  |  | Total | 36.02 | 20.34 | 31.36 | 9.75 | 2.54 |
|  | C | Females | 37.93 | 21.84 | 29.89 | 3.45 | 6.90 |
|  |  | Males | 44.23 | 9.62 | 30.77 | 9.62 | 5.77 |
|  |  | Non-binary gender | 100.00 | 0.00 | 0.00 | 0.00 | 0.00 |
|  |  | Total | 40.71 | 17.14 | 30.00 | 5.71 | 6.43 |
| **Latvia** | A | Females | 65.10 | 22.87 | 11.14 | 0.29 | 0.59 |
|  |  | Males | 72.50 | 12.50 | 10.00 | 2.50 | 2.50 |
|  |  | Non-binary gender | 100.00 | 0.00 | 0.00 | 0.00 | 0.00 |
|  |  | Total | 66.32 | 21.50 | 10.88 | 0.52 | 0.78 |
|  | B | Females | 60.10 | 17.73 | 15.76 | 2.96 | 3.45 |
|  |  | Males | 59.35 | 21.95 | 16.26 | 0.81 | 1.63 |
|  |  | Non-binary gender | 100.00 | 0.00 | 0.00 | 0.00 | 0.00 |
|  |  | Total | 60.06 | 19.21 | 15.85 | 2.13 | 2.74 |
|  | C | Females | 56.34 | 26.76 | 13.03 | 2.46 | 1.41 |
|  |  | Males | 53.06 | 22.45 | 16.33 | 4.08 | 4.08 |
|  |  | Non-binary gender |  |  |  |  |  |
|  |  | Total | 55.86 | 26.13 | 13.51 | 2.70 | 1.80 |
| **Lithuania** | A | Females | 68.62 | 21.28 | 8.51 | 1.06 | 0.53 |
|  |  | Males | 70.59 | 17.65 | 2.94 | 5.88 | 2.94 |
|  |  | Non-binary gender | 33.33 | 0.00 | 33.33 | 0.00 | 33.33 |
|  |  | Total | 68.44 | 20.44 | 8.00 | 1.78 | 1.33 |
|  | B | Females | 60.61 | 18.18 | 15.15 | 3.03 | 3.03 |
|  |  | Males | 25.00 | 37.50 | 12.50 | 12.50 | 12.50 |
|  |  | Non-binary gender |  |  |  |  |  |
|  |  | Total | 53.66 | 21.95 | 14.63 | 4.88 | 4.88 |
|  | C | Females | 57.14 | 25.00 | 12.50 | 5.36 | 0.00 |
|  |  | Males | 61.54 | 7.69 | 23.08 | 7.69 | 0.00 |
|  |  | Non-binary gender |  |  |  |  |  |
|  |  | Total | 57.97 | 21.74 | 14.49 | 5.80 | 0.00 |
| **Nigeria** | A | Females | 48.53 | 20.59 | 26.47 | 4.41 | 0.00 |
|  |  | Males | 56.86 | 21.57 | 17.65 | 3.92 | 0.00 |
|  |  | Non-binary gender |  |  |  |  |  |
|  |  | Total | 52.10 | 21.01 | 22.69 | 4.20 | 0.00 |
|  | B | Females | 62.63 | 18.18 | 16.16 | 3.03 | 0.00 |
|  |  | Males | 59.09 | 18.18 | 17.42 | 3.03 | 2.27 |
|  |  | Non-binary gender |  |  |  |  |  |
|  |  | Total | 60.61 | 18.18 | 16.88 | 3.03 | 1.30 |
|  | C | Females | 53.68 | 25.26 | 18.95 | 1.05 | 1.05 |
|  |  | Males | 50.35 | 25.53 | 22.70 | 0.71 | 0.71 |
|  |  | Non-binary gender | 0.00 | 0.00 | 100.00 | 0.00 | 0.00 |
|  |  | Total | 51.48 | 25.32 | 21.52 | 0.84 | 0.84 |
| **Russia** | A | Females | 58.23 | 15.27 | 20.51 | 3.89 | 2.10 |
|  |  | Males | 56.72 | 15.67 | 20.90 | 3.73 | 2.99 |
|  |  | Non-binary gender | 56.25 | 12.50 | 18.75 | 0.00 | 12.50 |
|  |  | Total | 57.95 | 15.28 | 20.54 | 3.79 | 2.44 |
|  | B | Females | 51.56 | 19.53 | 18.75 | 7.81 | 2.34 |
|  |  | Males | 52.10 | 16.81 | 23.53 | 1.68 | 5.88 |
|  |  | Non-binary gender | 57.14 | 28.57 | 0.00 | 14.29 | 0.00 |
|  |  | Total | 51.97 | 18.50 | 20.47 | 5.12 | 3.94 |
|  | C | Females | 49.03 | 20.23 | 23.35 | 7.00 | 0.39 |
|  |  | Males | 48.28 | 19.54 | 22.99 | 6.90 | 2.30 |
|  |  | Non-binary gender | 88.89 | 0.00 | 11.11 | 0.00 | 0.00 |
|  |  | Total | 49.86 | 19.55 | 22.95 | 6.80 | 0.85 |
| **Malaysia** | A | Females | 65.85 | 17.07 | 8.54 | 6.10 | 2.44 |
|  |  | Males | 38.46 | 30.77 | 7.69 | 23.08 | 0.00 |
|  |  | Non-binary gender | 100.00 | 0.00 | 0.00 | 0.00 | 0.00 |
|  |  | Total | 62.50 | 18.75 | 8.33 | 8.33 | 2.08 |
|  | B | Females | 18.75 | 37.50 | 15.63 | 21.88 | 6.25 |
|  |  | Males | 40.00 | 40.00 | 0.00 | 20.00 | 0.00 |
|  |  | Non-binary gender |  |  |  |  |  |
|  |  | Total | 23.81 | 38.10 | 11.90 | 21.43 | 4.76 |
|  | C | Females | 30.77 | 20.00 | 10.77 | 29.23 | 9.23 |
|  |  | Males | 28.57 | 7.14 | 21.43 | 35.71 | 7.14 |
|  |  | Non-binary gender | 100.00 | 0.00 | 0.00 | 0.00 | 0.00 |
|  |  | Total | 31.25 | 17.50 | 12.50 | 30.00 | 8.75 |
| **Hungary** | A | Females | 75.00 | 15.63 | 3.13 | 3.13 | 3.13 |
|  |  | Males | 88.89 | 11.11 | 0.00 | 0.00 | 0.00 |
|  |  | Non-binary gender |  |  |  |  |  |
|  |  | Total | 78.05 | 14.63 | 2.44 | 2.44 | 2.44 |
|  | B | Females | 30.00 | 40.00 | 0.00 | 20.00 | 10.00 |
|  |  | Males | 50.00 | 0.00 | 25.00 | 25.00 | 0.00 |
|  |  | Non-binary gender |  |  |  |  |  |
|  |  | Total | 35.71 | 28.57 | 7.14 | 21.43 | 7.14 |
|  | C | Females | 72.41 | 17.24 | 3.45 | 0.00 | 6.90 |
|  |  | Males | 66.67 | 0.00 | 0.00 | 33.33 | 0.00 |
|  |  | Non-binary gender |  |  |  |  |  |
|  |  | Total | 71.88 | 15.63 | 3.13 | 3.13 | 6.25 |
| **TOTAL** |  |  | 50.22 | 21.30 | 17.44 | 6.46 | 4.59 |
| **J23. Experiments involving new drugs or technologies are performed systematically on humans in a secret way and without their knowledge or consent.** | | | | | | | |
| **Bulgaria** | A | Females | 61.70 | 10.64 | 19.15 | 6.38 | 2.13 |
|  |  | Males | 62.22 | 24.44 | 6.67 | 0.00 | 6.67 |
|  |  | Non-binary gender | 50.00 | 50.00 | 0.00 | 0.00 | 0.00 |
|  |  | Total | 61.70 | 18.09 | 12.77 | 3.19 | 4.26 |
|  | B | Females | 32.14 | 17.86 | 17.86 | 8.93 | 23.21 |
|  |  | Males | 30.00 | 20.00 | 26.67 | 13.33 | 10.00 |
|  |  | Non-binary gender |  |  |  |  |  |
|  |  | Total | 31.40 | 18.60 | 20.93 | 10.47 | 18.60 |
|  | C | Females | 20.34 | 15.25 | 30.51 | 15.25 | 18.64 |
|  |  | Males | 40.74 | 25.93 | 18.52 | 7.41 | 7.41 |
|  |  | Non-binary gender |  |  |  |  |  |
|  |  | Total | 26.74 | 18.60 | 26.74 | 12.79 | 15.12 |
| **Croatia** | A | Females | 53.90 | 25.97 | 13.20 | 4.55 | 2.38 |
|  |  | Males | 66.25 | 18.75 | 8.75 | 2.50 | 3.75 |
|  |  | Non-binary gender | 100.00 | 0.00 | 0.00 | 0.00 | 0.00 |
|  |  | Total | 55.80 | 24.86 | 12.52 | 4.24 | 2.58 |
|  | B | Females | 41.70 | 17.69 | 20.96 | 12.01 | 7.64 |
|  |  | Males | 45.51 | 24.55 | 16.77 | 7.78 | 5.39 |
|  |  | Non-binary gender | 16.67 | 33.33 | 50.00 | 0.00 | 0.00 |
|  |  | Total | 42.47 | 19.65 | 20.13 | 10.78 | 6.97 |
|  | C | Females | 42.81 | 21.56 | 21.56 | 7.19 | 6.89 |
|  |  | Males | 46.00 | 22.00 | 16.00 | 8.00 | 8.00 |
|  |  | Non-binary gender | 50.00 | 50.00 | 0.00 | 0.00 | 0.00 |
|  |  | Total | 43.26 | 21.76 | 20.73 | 7.25 | 6.99 |
| **Georgia** | A | Females | 49.72 | 21.51 | 18.44 | 6.42 | 3.91 |
|  |  | Males | 41.72 | 28.22 | 21.47 | 3.07 | 5.52 |
|  |  | Non-binary gender | 50.00 | 0.00 | 50.00 | 0.00 | 0.00 |
|  |  | Total | 47.23 | 23.52 | 19.50 | 5.35 | 4.40 |
|  | B | Females | 42.28 | 31.54 | 14.09 | 5.37 | 6.71 |
|  |  | Males | 39.24 | 24.05 | 26.58 | 6.33 | 3.80 |
|  |  | Non-binary gender | 33.33 | 0.00 | 66.67 | 0.00 | 0.00 |
|  |  | Total | 41.13 | 28.57 | 19.05 | 5.63 | 5.63 |
|  | C | Females | 41.36 | 26.70 | 18.32 | 7.85 | 5.76 |
|  |  | Males | 42.11 | 34.21 | 15.79 | 5.26 | 2.63 |
|  |  | Non-binary gender | 0.00 | 0.00 | 0.00 | 0.00 | 100.00 |
|  |  | Total | 41.30 | 27.83 | 17.83 | 7.39 | 5.65 |
| **Greece** | A | Females | 66.48 | 19.78 | 9.23 | 3.19 | 1.32 |
|  |  | Males | 68.21 | 18.93 | 9.29 | 1.79 | 1.79 |
|  |  | Non-binary gender | 61.54 | 15.38 | 7.69 | 15.38 | 0.00 |
|  |  | Total | 66.83 | 19.53 | 9.23 | 2.99 | 1.41 |
|  | B | Females | 58.71 | 21.96 | 13.29 | 3.82 | 2.23 |
|  |  | Males | 67.36 | 19.19 | 7.95 | 4.03 | 1.47 |
|  |  | Non-binary gender | 55.56 | 29.63 | 3.70 | 7.41 | 3.70 |
|  |  | Total | 62.04 | 20.98 | 11.08 | 3.95 | 1.95 |
|  | C | Females | 55.12 | 22.75 | 15.73 | 4.13 | 2.26 |
|  |  | Males | 60.28 | 20.00 | 13.52 | 4.23 | 1.97 |
|  |  | Non-binary gender | 68.97 | 17.24 | 3.45 | 3.45 | 6.90 |
|  |  | Total | 56.16 | 22.22 | 15.20 | 4.14 | 2.28 |
| **India** | A | Females | 35.85 | 26.42 | 31.13 | 4.72 | 1.89 |
|  |  | Males | 38.46 | 28.21 | 20.51 | 5.13 | 7.69 |
|  |  | Non-binary gender |  |  |  |  |  |
|  |  | Total | 36.55 | 26.90 | 28.28 | 4.83 | 3.45 |
|  | B | Females | 34.21 | 16.67 | 32.46 | 11.40 | 5.26 |
|  |  | Males | 36.89 | 26.23 | 30.33 | 3.28 | 3.28 |
|  |  | Non-binary gender |  |  |  |  |  |
|  |  | Total | 35.59 | 21.61 | 31.36 | 7.20 | 4.24 |
|  | C | Females | 44.83 | 17.24 | 26.44 | 8.05 | 3.45 |
|  |  | Males | 44.23 | 11.54 | 25.00 | 13.46 | 5.77 |
|  |  | Non-binary gender | 0.00 | 100.00 | 0.00 | 0.00 | 0.00 |
|  |  | Total | 44.29 | 15.71 | 25.71 | 10.00 | 4.29 |
| **Latvia** | A | Females | 74.78 | 12.90 | 11.44 | 0.59 | 0.29 |
|  |  | Males | 65.00 | 17.50 | 17.50 | 0.00 | 0.00 |
|  |  | Non-binary gender | 80.00 | 0.00 | 20.00 | 0.00 | 0.00 |
|  |  | Total | 73.83 | 13.21 | 12.18 | 0.52 | 0.26 |
|  | B | Females | 54.19 | 19.21 | 21.18 | 1.97 | 3.45 |
|  |  | Males | 56.91 | 18.70 | 18.70 | 5.69 | 0.00 |
|  |  | Non-binary gender | 50.00 | 0.00 | 50.00 | 0.00 | 0.00 |
|  |  | Total | 55.18 | 18.90 | 20.43 | 3.35 | 2.13 |
|  | C | Females | 59.51 | 16.20 | 17.61 | 4.58 | 2.11 |
|  |  | Males | 51.02 | 18.37 | 24.49 | 6.12 | 0.00 |
|  |  | Non-binary gender |  |  |  |  |  |
|  |  | Total | 58.26 | 16.52 | 18.62 | 4.80 | 1.80 |
| **Lithuania** | A | Females | 77.13 | 15.96 | 4.26 | 2.66 | 0.00 |
|  |  | Males | 91.18 | 2.94 | 0.00 | 2.94 | 2.94 |
|  |  | Non-binary gender | 66.67 | 0.00 | 0.00 | 33.33 | 0.00 |
|  |  | Total | 79.11 | 13.78 | 3.56 | 3.11 | 0.44 |
|  | B | Females | 66.67 | 15.15 | 12.12 | 0.00 | 6.06 |
|  |  | Males | 50.00 | 25.00 | 12.50 | 12.50 | 0.00 |
|  |  | Non-binary gender |  |  |  |  |  |
|  |  | Total | 63.41 | 17.07 | 12.20 | 2.44 | 4.88 |
|  | C | Females | 60.71 | 26.79 | 8.93 | 1.79 | 1.79 |
|  |  | Males | 61.54 | 23.08 | 15.38 | 0.00 | 0.00 |
|  |  | Non-binary gender |  |  |  |  |  |
|  |  | Total | 60.87 | 26.09 | 10.14 | 1.45 | 1.45 |
| **Nigeria** | A | Females | 50.00 | 22.06 | 26.47 | 1.47 | 0.00 |
|  |  | Males | 58.82 | 17.65 | 21.57 | 1.96 | 0.00 |
|  |  | Non-binary gender |  |  |  |  |  |
|  |  | Total | 53.78 | 20.17 | 24.37 | 1.68 | 0.00 |
|  | B | Females | 61.62 | 20.20 | 16.16 | 2.02 | 0.00 |
|  |  | Males | 51.52 | 22.73 | 21.21 | 3.79 | 0.76 |
|  |  | Non-binary gender |  |  |  |  |  |
|  |  | Total | 55.84 | 21.65 | 19.05 | 3.03 | 0.43 |
|  | C | Females | 63.16 | 22.11 | 11.58 | 1.05 | 2.11 |
|  |  | Males | 56.03 | 25.53 | 17.02 | 0.71 | 0.71 |
|  |  | Non-binary gender | 0.00 | 100.00 | 0.00 | 0.00 | 0.00 |
|  |  | Total | 58.65 | 24.47 | 14.77 | 0.84 | 1.27 |
| **Russia** | A | Females | 54.19 | 19.91 | 19.31 | 4.49 | 2.10 |
|  |  | Males | 46.27 | 26.12 | 22.39 | 2.99 | 2.24 |
|  |  | Non-binary gender | 31.25 | 31.25 | 25.00 | 6.25 | 6.25 |
|  |  | Total | 52.44 | 21.15 | 19.93 | 4.28 | 2.20 |
|  | B | Females | 42.97 | 21.88 | 23.44 | 4.69 | 7.03 |
|  |  | Males | 48.74 | 18.49 | 19.33 | 9.24 | 4.20 |
|  |  | Non-binary gender | 42.86 | 14.29 | 42.86 | 0.00 | 0.00 |
|  |  | Total | 45.67 | 20.08 | 22.05 | 6.69 | 5.51 |
|  | C | Females | 44.36 | 21.01 | 26.07 | 5.84 | 2.72 |
|  |  | Males | 55.17 | 19.54 | 17.24 | 6.90 | 1.15 |
|  |  | Non-binary gender | 66.67 | 11.11 | 22.22 | 0.00 | 0.00 |
|  |  | Total | 47.59 | 20.40 | 23.80 | 5.95 | 2.27 |
| **Malaysia** | A | Females | 67.07 | 24.39 | 4.88 | 2.44 | 1.22 |
|  |  | Males | 46.15 | 30.77 | 7.69 | 15.38 | 0.00 |
|  |  | Non-binary gender | 0.00 | 100.00 | 0.00 | 0.00 | 0.00 |
|  |  | Total | 63.54 | 26.04 | 5.21 | 4.17 | 1.04 |
|  | B | Females | 34.38 | 21.88 | 15.63 | 25.00 | 3.13 |
|  |  | Males | 40.00 | 40.00 | 0.00 | 20.00 | 0.00 |
|  |  | Non-binary gender |  |  |  |  |  |
|  |  | Total | 35.71 | 26.19 | 11.90 | 23.81 | 2.38 |
|  | C | Females | 33.85 | 27.69 | 7.69 | 23.08 | 7.69 |
|  |  | Males | 28.57 | 14.29 | 28.57 | 21.43 | 7.14 |
|  |  | Non-binary gender | 0.00 | 100.00 | 0.00 | 0.00 | 0.00 |
|  |  | Total | 32.50 | 26.25 | 11.25 | 22.50 | 7.50 |
| **Hungary** | A | Females | 84.38 | 6.25 | 3.13 | 6.25 | 0.00 |
|  |  | Males | 88.89 | 11.11 | 0.00 | 0.00 | 0.00 |
|  |  | Non-binary gender |  |  |  |  |  |
|  |  | Total | 85.37 | 7.32 | 2.44 | 4.88 | 0.00 |
|  | B | Females | 60.00 | 20.00 | 10.00 | 0.00 | 10.00 |
|  |  | Males | 50.00 | 0.00 | 50.00 | 0.00 | 0.00 |
|  |  | Non-binary gender |  |  |  |  |  |
|  |  | Total | 57.14 | 14.29 | 21.43 | 0.00 | 7.14 |
|  | C | Females | 65.52 | 17.24 | 10.34 | 3.45 | 3.45 |
|  |  | Males | 66.67 | 0.00 | 0.00 | 33.33 | 0.00 |
|  |  | Non-binary gender |  |  |  |  |  |
|  |  | Total | 65.63 | 15.63 | 9.38 | 6.25 | 3.13 |
| **TOTAL** |  |  | 55.38 | 21.06 | 15.78 | 4.84 | 2.94 |
| **J24. Many important pieces of information are deliberately hidden from the public for reasons of interest.** | | | | | | | |
| **Bulgaria** | A | Females | 36.17 | 8.51 | 29.79 | 12.77 | 12.77 |
|  |  | Males | 31.11 | 17.78 | 31.11 | 8.89 | 11.11 |
|  |  | Non-binary gender | 0.00 | 100.00 | 0.00 | 0.00 | 0.00 |
|  |  | Total | 32.98 | 14.89 | 29.79 | 10.64 | 11.70 |
|  | B | Females | 5.36 | 8.93 | 23.21 | 14.29 | 48.21 |
|  |  | Males | 13.33 | 10.00 | 20.00 | 23.33 | 33.33 |
|  |  | Non-binary gender |  |  |  |  |  |
|  |  | Total | 8.14 | 9.30 | 22.09 | 17.44 | 43.02 |
|  | C | Females | 8.47 | 5.08 | 30.51 | 16.95 | 38.98 |
|  |  | Males | 37.04 | 25.93 | 14.81 | 7.41 | 14.81 |
|  |  | Non-binary gender |  |  |  |  |  |
|  |  | Total | 17.44 | 11.63 | 25.58 | 13.95 | 31.40 |
| **Croatia** | A | Females | 15.58 | 19.05 | 23.38 | 20.56 | 21.43 |
|  |  | Males | 18.75 | 23.75 | 33.75 | 10.00 | 13.75 |
|  |  | Non-binary gender | 0.00 | 0.00 | 100.00 | 0.00 | 0.00 |
|  |  | Total | 16.02 | 19.71 | 25.05 | 18.97 | 20.26 |
|  | B | Females | 10.26 | 15.72 | 20.52 | 21.18 | 32.31 |
|  |  | Males | 17.37 | 12.57 | 19.76 | 19.16 | 31.14 |
|  |  | Non-binary gender | 16.67 | 16.67 | 33.33 | 16.67 | 16.67 |
|  |  | Total | 12.20 | 14.90 | 20.44 | 20.60 | 31.85 |
|  | C | Females | 9.58 | 19.16 | 23.65 | 16.77 | 30.84 |
|  |  | Males | 14.00 | 24.00 | 16.00 | 16.00 | 30.00 |
|  |  | Non-binary gender | 50.00 | 0.00 | 50.00 | 0.00 | 0.00 |
|  |  | Total | 10.36 | 19.69 | 22.80 | 16.58 | 30.57 |
| **Georgia** | A | Females | 23.74 | 21.23 | 28.49 | 12.01 | 14.53 |
|  |  | Males | 19.63 | 20.86 | 31.29 | 12.27 | 15.95 |
|  |  | Non-binary gender | 0.00 | 0.00 | 100.00 | 0.00 | 0.00 |
|  |  | Total | 22.37 | 21.03 | 29.64 | 12.05 | 14.91 |
|  | B | Females | 24.16 | 18.79 | 24.16 | 16.78 | 16.11 |
|  |  | Males | 22.78 | 16.46 | 22.78 | 17.72 | 20.25 |
|  |  | Non-binary gender | 33.33 | 0.00 | 66.67 | 0.00 | 0.00 |
|  |  | Total | 23.81 | 17.75 | 24.24 | 16.88 | 17.32 |
|  | C | Females | 17.80 | 24.61 | 22.51 | 13.61 | 21.47 |
|  |  | Males | 18.42 | 21.05 | 23.68 | 18.42 | 18.42 |
|  |  | Non-binary gender | 0.00 | 0.00 | 0.00 | 0.00 | 100.00 |
|  |  | Total | 17.83 | 23.91 | 22.61 | 14.35 | 21.30 |
| **Greece** | A | Females | 11.43 | 22.53 | 23.19 | 21.87 | 20.99 |
|  |  | Males | 14.64 | 23.93 | 22.14 | 20.71 | 18.57 |
|  |  | Non-binary gender | 0.00 | 15.38 | 30.77 | 30.77 | 23.08 |
|  |  | Total | 12.05 | 22.78 | 23.03 | 21.70 | 20.45 |
|  | B | Females | 9.31 | 20.37 | 26.01 | 22.75 | 21.56 |
|  |  | Males | 14.43 | 22.25 | 22.00 | 19.32 | 22.00 |
|  |  | Non-binary gender | 7.41 | 14.81 | 29.63 | 14.81 | 33.33 |
|  |  | Total | 11.27 | 21.03 | 24.50 | 21.31 | 21.88 |
|  | C | Females | 9.73 | 17.88 | 24.05 | 21.79 | 26.54 |
|  |  | Males | 11.55 | 20.85 | 24.79 | 20.00 | 22.82 |
|  |  | Non-binary gender | 10.34 | 31.03 | 10.34 | 17.24 | 31.03 |
|  |  | Total | 10.04 | 18.55 | 23.99 | 21.43 | 25.99 |
| **India** | A | Females | 25.47 | 30.19 | 30.19 | 7.55 | 6.60 |
|  |  | Males | 20.51 | 33.33 | 28.21 | 12.82 | 5.13 |
|  |  | Non-binary gender |  |  |  |  |  |
|  |  | Total | 24.14 | 31.03 | 29.66 | 8.97 | 6.21 |
|  | B | Females | 16.67 | 11.40 | 36.84 | 21.93 | 13.16 |
|  |  | Males | 17.21 | 21.31 | 37.70 | 11.48 | 12.30 |
|  |  | Non-binary gender |  |  |  |  |  |
|  |  | Total | 16.95 | 16.53 | 37.29 | 16.53 | 12.71 |
|  | C | Females | 22.99 | 17.24 | 32.18 | 12.64 | 14.94 |
|  |  | Males | 11.54 | 25.00 | 34.62 | 11.54 | 17.31 |
|  |  | Non-binary gender | 0.00 | 0.00 | 100.00 | 0.00 | 0.00 |
|  |  | Total | 18.57 | 20.00 | 33.57 | 12.14 | 15.71 |
| **Latvia** | A | Females | 33.43 | 25.51 | 24.93 | 9.97 | 6.16 |
|  |  | Males | 35.00 | 22.50 | 35.00 | 5.00 | 2.50 |
|  |  | Non-binary gender | 60.00 | 0.00 | 20.00 | 20.00 | 0.00 |
|  |  | Total | 33.94 | 24.87 | 25.91 | 9.59 | 5.70 |
|  | B | Females | 14.78 | 22.66 | 31.03 | 17.73 | 13.79 |
|  |  | Males | 27.64 | 19.51 | 26.83 | 13.01 | 13.01 |
|  |  | Non-binary gender | 0.00 | 0.00 | 50.00 | 50.00 | 0.00 |
|  |  | Total | 19.51 | 21.34 | 29.57 | 16.16 | 13.41 |
|  | C | Females | 17.61 | 22.89 | 27.46 | 19.37 | 12.68 |
|  |  | Males | 34.69 | 8.16 | 34.69 | 6.12 | 16.33 |
|  |  | Non-binary gender |  |  |  |  |  |
|  |  | Total | 20.12 | 20.72 | 28.53 | 17.42 | 13.21 |
| **Lithuania** | A | Females | 30.85 | 33.51 | 18.62 | 10.64 | 6.38 |
|  |  | Males | 44.12 | 26.47 | 11.76 | 5.88 | 11.76 |
|  |  | Non-binary gender | 0.00 | 0.00 | 33.33 | 33.33 | 33.33 |
|  |  | Total | 32.44 | 32.00 | 17.78 | 10.22 | 7.56 |
|  | B | Females | 36.36 | 24.24 | 12.12 | 12.12 | 15.15 |
|  |  | Males | 0.00 | 12.50 | 37.50 | 37.50 | 12.50 |
|  |  | Non-binary gender |  |  |  |  |  |
|  |  | Total | 29.27 | 21.95 | 17.07 | 17.07 | 14.63 |
|  | C | Females | 12.50 | 44.64 | 17.86 | 10.71 | 14.29 |
|  |  | Males | 23.08 | 23.08 | 38.46 | 7.69 | 7.69 |
|  |  | Non-binary gender |  |  |  |  |  |
|  |  | Total | 14.49 | 40.58 | 21.74 | 10.14 | 13.04 |
| **Nigeria** | A | Females | 44.12 | 26.47 | 20.59 | 8.82 | 0.00 |
|  |  | Males | 47.06 | 21.57 | 25.49 | 3.92 | 1.96 |
|  |  | Non-binary gender |  |  |  |  |  |
|  |  | Total | 45.38 | 24.37 | 22.69 | 6.72 | 0.84 |
|  | B | Females | 63.64 | 21.21 | 13.13 | 2.02 | 0.00 |
|  |  | Males | 54.55 | 19.70 | 19.70 | 4.55 | 1.52 |
|  |  | Non-binary gender |  |  |  |  |  |
|  |  | Total | 58.44 | 20.35 | 16.88 | 3.46 | 0.87 |
|  | C | Females | 64.21 | 14.74 | 18.95 | 1.05 | 1.05 |
|  |  | Males | 52.48 | 27.66 | 18.44 | 0.71 | 0.71 |
|  |  | Non-binary gender | 100.00 | 0.00 | 0.00 | 0.00 | 0.00 |
|  |  | Total | 57.38 | 22.36 | 18.57 | 0.84 | 0.84 |
| **Russia** | A | Females | 17.37 | 14.97 | 30.24 | 22.16 | 15.27 |
|  |  | Males | 15.67 | 16.42 | 30.60 | 24.63 | 12.69 |
|  |  | Non-binary gender | 37.50 | 31.25 | 12.50 | 6.25 | 12.50 |
|  |  | Total | 17.48 | 15.53 | 29.95 | 22.25 | 14.79 |
|  | B | Females | 26.56 | 13.28 | 23.44 | 17.19 | 19.53 |
|  |  | Males | 24.37 | 15.97 | 31.93 | 11.76 | 15.97 |
|  |  | Non-binary gender | 42.86 | 42.86 | 14.29 | 0.00 | 0.00 |
|  |  | Total | 25.98 | 15.35 | 27.17 | 14.17 | 17.32 |
|  | C | Females | 18.29 | 11.67 | 29.96 | 22.57 | 17.51 |
|  |  | Males | 20.69 | 19.54 | 33.33 | 10.34 | 16.09 |
|  |  | Non-binary gender | 44.44 | 11.11 | 44.44 | 0.00 | 0.00 |
|  |  | Total | 19.55 | 13.60 | 31.16 | 18.98 | 16.71 |
| **Malaysia** | A | Females | 46.34 | 24.39 | 18.29 | 7.32 | 3.66 |
|  |  | Males | 46.15 | 30.77 | 0.00 | 15.38 | 7.69 |
|  |  | Non-binary gender | 0.00 | 100.00 | 0.00 | 0.00 | 0.00 |
|  |  | Total | 45.83 | 26.04 | 15.63 | 8.33 | 4.17 |
|  | B | Females | 12.50 | 25.00 | 15.63 | 28.13 | 18.75 |
|  |  | Males | 10.00 | 30.00 | 20.00 | 40.00 | 0.00 |
|  |  | Non-binary gender |  |  |  |  |  |
|  |  | Total | 11.90 | 26.19 | 16.67 | 30.95 | 14.29 |
|  | C | Females | 20.00 | 23.08 | 7.69 | 26.15 | 23.08 |
|  |  | Males | 21.43 | 7.14 | 28.57 | 35.71 | 7.14 |
|  |  | Non-binary gender | 0.00 | 100.00 | 0.00 | 0.00 | 0.00 |
|  |  | Total | 20.00 | 21.25 | 11.25 | 27.50 | 20.00 |
| **Hungary** | A | Females | 12.50 | 37.50 | 18.75 | 12.50 | 18.75 |
|  |  | Males | 44.44 | 0.00 | 22.22 | 11.11 | 22.22 |
|  |  | Non-binary gender |  |  |  |  |  |
|  |  | Total | 19.51 | 29.27 | 19.51 | 12.20 | 19.51 |
|  | B | Females | 20.00 | 20.00 | 10.00 | 10.00 | 40.00 |
|  |  | Males | 0.00 | 0.00 | 0.00 | 75.00 | 25.00 |
|  |  | Non-binary gender |  |  |  |  |  |
|  |  | Total | 14.29 | 14.29 | 7.14 | 28.57 | 35.71 |
|  | C | Females | 13.79 | 17.24 | 6.90 | 27.59 | 34.48 |
|  |  | Males | 0.00 | 33.33 | 33.33 | 0.00 | 33.33 |
|  |  | Non-binary gender |  |  |  |  |  |
|  |  | Total | 12.50 | 18.75 | 9.38 | 25.00 | 34.38 |
| **TOTAL** |  |  | 17.68 | 20.04 | 24.84 | 18.07 | 19.37 |
| **J25. The spread of certain viruses and/or diseases is the result of deliberate, covert actions of an organization or government.** | | | | | | | |
| **Bulgaria** | A | Females | 48.94 | 10.64 | 27.66 | 4.26 | 8.51 |
|  |  | Males | 55.56 | 31.11 | 4.44 | 4.44 | 4.44 |
|  |  | Non-binary gender | 100.00 | 0.00 | 0.00 | 0.00 | 0.00 |
|  |  | Total | 53.19 | 20.21 | 15.96 | 4.26 | 6.38 |
|  | B | Females | 17.86 | 17.86 | 25.00 | 5.36 | 33.93 |
|  |  | Males | 30.00 | 26.67 | 16.67 | 13.33 | 13.33 |
|  |  | Non-binary gender |  |  |  |  |  |
|  |  | Total | 22.09 | 20.93 | 22.09 | 8.14 | 26.74 |
|  | C | Females | 11.86 | 16.95 | 28.81 | 22.03 | 20.34 |
|  |  | Males | 51.85 | 25.93 | 11.11 | 7.41 | 3.70 |
|  |  | Non-binary gender |  |  |  |  |  |
|  |  | Total | 24.42 | 19.77 | 23.26 | 17.44 | 15.12 |
| **Croatia** | A | Females | 51.52 | 17.10 | 18.61 | 8.01 | 4.76 |
|  |  | Males | 65.00 | 18.75 | 5.00 | 6.25 | 5.00 |
|  |  | Non-binary gender | 100.00 | 0.00 | 0.00 | 0.00 | 0.00 |
|  |  | Total | 53.59 | 17.31 | 16.57 | 7.73 | 4.79 |
|  | B | Females | 35.81 | 15.94 | 23.58 | 8.30 | 16.38 |
|  |  | Males | 38.92 | 23.95 | 19.76 | 7.78 | 9.58 |
|  |  | Non-binary gender | 66.67 | 16.67 | 16.67 | 0.00 | 0.00 |
|  |  | Total | 36.93 | 18.07 | 22.50 | 8.08 | 14.42 |
|  | C | Females | 38.32 | 18.86 | 23.35 | 7.78 | 11.68 |
|  |  | Males | 32.00 | 24.00 | 24.00 | 6.00 | 14.00 |
|  |  | Non-binary gender | 50.00 | 0.00 | 50.00 | 0.00 | 0.00 |
|  |  | Total | 37.56 | 19.43 | 23.58 | 7.51 | 11.92 |
| **Georgia** | A | Females | 48.88 | 22.35 | 17.32 | 5.59 | 5.87 |
|  |  | Males | 45.40 | 20.25 | 19.63 | 6.75 | 7.98 |
|  |  | Non-binary gender | 100.00 | 0.00 | 0.00 | 0.00 | 0.00 |
|  |  | Total | 47.99 | 21.61 | 17.97 | 5.93 | 6.50 |
|  | B | Females | 42.28 | 25.50 | 16.78 | 8.05 | 7.38 |
|  |  | Males | 51.90 | 21.52 | 20.25 | 5.06 | 1.27 |
|  |  | Non-binary gender | 33.33 | 33.33 | 33.33 | 0.00 | 0.00 |
|  |  | Total | 45.45 | 24.24 | 18.18 | 6.93 | 5.19 |
|  | C | Females | 43.98 | 24.08 | 18.85 | 7.33 | 5.76 |
|  |  | Males | 47.37 | 18.42 | 23.68 | 5.26 | 5.26 |
|  |  | Non-binary gender | 0.00 | 0.00 | 0.00 | 0.00 | 100.00 |
|  |  | Total | 44.35 | 23.04 | 19.57 | 6.96 | 6.09 |
| **Greece** | A | Females | 61.65 | 18.35 | 12.31 | 4.40 | 3.30 |
|  |  | Males | 70.36 | 17.14 | 5.36 | 4.29 | 2.86 |
|  |  | Non-binary gender | 46.15 | 30.77 | 15.38 | 7.69 | 0.00 |
|  |  | Total | 63.51 | 18.20 | 10.72 | 4.41 | 3.16 |
|  | B | Females | 54.81 | 20.13 | 16.15 | 5.33 | 3.58 |
|  |  | Males | 68.34 | 17.11 | 8.68 | 2.93 | 2.93 |
|  |  | Non-binary gender | 48.15 | 25.93 | 11.11 | 11.11 | 3.70 |
|  |  | Total | 59.99 | 19.03 | 13.18 | 4.47 | 3.33 |
|  | C | Females | 49.01 | 20.32 | 18.68 | 7.58 | 4.41 |
|  |  | Males | 59.15 | 18.59 | 13.24 | 3.94 | 5.07 |
|  |  | Non-binary gender | 68.97 | 20.69 | 10.34 | 0.00 | 0.00 |
|  |  | Total | 50.95 | 20.04 | 17.67 | 6.88 | 4.46 |
| **India** | A | Females | 37.74 | 29.25 | 26.42 | 4.72 | 1.89 |
|  |  | Males | 43.59 | 30.77 | 15.38 | 7.69 | 2.56 |
|  |  | Non-binary gender |  |  |  |  |  |
|  |  | Total | 39.31 | 29.66 | 23.45 | 5.52 | 2.07 |
|  | B | Females | 35.09 | 18.42 | 28.95 | 9.65 | 7.89 |
|  |  | Males | 45.08 | 18.03 | 27.05 | 4.92 | 4.92 |
|  |  | Non-binary gender |  |  |  |  |  |
|  |  | Total | 40.25 | 18.22 | 27.97 | 7.20 | 6.36 |
|  | C | Females | 47.13 | 17.24 | 24.14 | 5.75 | 5.75 |
|  |  | Males | 42.31 | 15.38 | 21.15 | 13.46 | 7.69 |
|  |  | Non-binary gender | 0.00 | 0.00 | 0.00 | 100.00 | 0.00 |
|  |  | Total | 45.00 | 16.43 | 22.86 | 9.29 | 6.43 |
| **Latvia** | A | Females | 72.43 | 12.02 | 11.44 | 2.64 | 1.47 |
|  |  | Males | 77.50 | 12.50 | 7.50 | 0.00 | 2.50 |
|  |  | Non-binary gender | 100.00 | 0.00 | 0.00 | 0.00 | 0.00 |
|  |  | Total | 73.32 | 11.92 | 10.88 | 2.33 | 1.55 |
|  | B | Females | 58.62 | 12.81 | 18.72 | 4.43 | 5.42 |
|  |  | Males | 56.91 | 13.82 | 20.33 | 6.50 | 2.44 |
|  |  | Non-binary gender | 0.00 | 50.00 | 0.00 | 50.00 | 0.00 |
|  |  | Total | 57.62 | 13.41 | 19.21 | 5.49 | 4.27 |
|  | C | Females | 58.10 | 15.49 | 18.31 | 4.58 | 3.52 |
|  |  | Males | 61.22 | 10.20 | 20.41 | 4.08 | 4.08 |
|  |  | Non-binary gender |  |  |  |  |  |
|  |  | Total | 58.56 | 14.71 | 18.62 | 4.50 | 3.60 |
| **Lithuania** | A | Females | 81.38 | 10.11 | 5.32 | 3.19 | 0.00 |
|  |  | Males | 79.41 | 14.71 | 2.94 | 0.00 | 2.94 |
|  |  | Non-binary gender | 66.67 | 0.00 | 33.33 | 0.00 | 0.00 |
|  |  | Total | 80.89 | 10.67 | 5.33 | 2.67 | 0.44 |
|  | B | Females | 69.70 | 9.09 | 6.06 | 6.06 | 9.09 |
|  |  | Males | 50.00 | 25.00 | 12.50 | 12.50 | 0.00 |
|  |  | Non-binary gender |  |  |  |  |  |
|  |  | Total | 65.85 | 12.20 | 7.32 | 7.32 | 7.32 |
|  | C | Females | 64.29 | 19.64 | 12.50 | 0.00 | 3.57 |
|  |  | Males | 69.23 | 7.69 | 23.08 | 0.00 | 0.00 |
|  |  | Non-binary gender |  |  |  |  |  |
|  |  | Total | 65.22 | 17.39 | 14.49 | 0.00 | 2.90 |
| **Nigeria** | A | Females | 58.82 | 22.06 | 19.12 | 0.00 | 0.00 |
|  |  | Males | 56.86 | 13.73 | 23.53 | 5.88 | 0.00 |
|  |  | Non-binary gender |  |  |  |  |  |
|  |  | Total | 57.98 | 18.49 | 21.01 | 2.52 | 0.00 |
|  | B | Females | 69.70 | 19.19 | 10.10 | 1.01 | 0.00 |
|  |  | Males | 60.61 | 14.39 | 20.45 | 3.03 | 1.52 |
|  |  | Non-binary gender |  |  |  |  |  |
|  |  | Total | 64.50 | 16.45 | 16.02 | 2.16 | 0.87 |
|  | C | Females | 61.05 | 21.05 | 14.74 | 2.11 | 1.05 |
|  |  | Males | 56.74 | 25.53 | 16.31 | 0.71 | 0.71 |
|  |  | Non-binary gender | 0.00 | 100.00 | 0.00 | 0.00 | 0.00 |
|  |  | Total | 58.23 | 24.05 | 15.61 | 1.27 | 0.84 |
| **Russia** | A | Females | 55.84 | 16.32 | 19.76 | 4.94 | 3.14 |
|  |  | Males | 55.22 | 18.66 | 20.90 | 2.99 | 2.24 |
|  |  | Non-binary gender | 50.00 | 25.00 | 12.50 | 0.00 | 12.50 |
|  |  | Total | 55.62 | 16.87 | 19.80 | 4.52 | 3.18 |
|  | B | Females | 48.44 | 21.09 | 20.31 | 3.91 | 6.25 |
|  |  | Males | 47.90 | 21.85 | 21.85 | 4.20 | 4.20 |
|  |  | Non-binary gender | 28.57 | 42.86 | 14.29 | 14.29 | 0.00 |
|  |  | Total | 47.64 | 22.05 | 20.87 | 4.33 | 5.12 |
|  | C | Females | 47.47 | 20.23 | 24.90 | 4.67 | 2.72 |
|  |  | Males | 50.57 | 19.54 | 20.69 | 4.60 | 4.60 |
|  |  | Non-binary gender | 77.78 | 11.11 | 11.11 | 0.00 | 0.00 |
|  |  | Total | 49.01 | 19.83 | 23.51 | 4.53 | 3.12 |
| **Malaysia** | A | Females | 64.63 | 23.17 | 3.66 | 4.88 | 3.66 |
|  |  | Males | 46.15 | 23.08 | 0.00 | 23.08 | 7.69 |
|  |  | Non-binary gender | 100.00 | 0.00 | 0.00 | 0.00 | 0.00 |
|  |  | Total | 62.50 | 22.92 | 3.13 | 7.29 | 4.17 |
|  | B | Females | 28.13 | 28.13 | 12.50 | 25.00 | 6.25 |
|  |  | Males | 20.00 | 50.00 | 10.00 | 20.00 | 0.00 |
|  |  | Non-binary gender |  |  |  |  |  |
|  |  | Total | 26.19 | 33.33 | 11.90 | 23.81 | 4.76 |
|  | C | Females | 43.08 | 23.08 | 9.23 | 18.46 | 6.15 |
|  |  | Males | 28.57 | 14.29 | 21.43 | 28.57 | 7.14 |
|  |  | Non-binary gender | 0.00 | 100.00 | 0.00 | 0.00 | 0.00 |
|  |  | Total | 40.00 | 22.50 | 11.25 | 20.00 | 6.25 |
| **Hungary** | A | Females | 75.00 | 12.50 | 6.25 | 3.13 | 3.13 |
|  |  | Males | 77.78 | 22.22 | 0.00 | 0.00 | 0.00 |
|  |  | Non-binary gender |  |  |  |  |  |
|  |  | Total | 75.61 | 14.63 | 4.88 | 2.44 | 2.44 |
|  | B | Females | 50.00 | 20.00 | 0.00 | 0.00 | 30.00 |
|  |  | Males | 100.00 | 0.00 | 0.00 | 0.00 | 0.00 |
|  |  | Non-binary gender |  |  |  |  |  |
|  |  | Total | 64.29 | 14.29 | 0.00 | 0.00 | 21.43 |
|  | C | Females | 62.07 | 20.69 | 10.34 | 6.90 | 0.00 |
|  |  | Males | 66.67 | 0.00 | 33.33 | 0.00 | 0.00 |
|  |  | Non-binary gender |  |  |  |  |  |
|  |  | Total | 62.50 | 18.75 | 12.50 | 6.25 | 0.00 |
| **TOTAL** |  |  | 54.00 | 18.80 | 16.72 | 5.65 | 4.83 |
| **J26. It is possible that the earth is flat rather than a spherical.** | | | | | | | |
| **Bulgaria** | A | Females | 93.62 | 2.13 | 0.00 | 4.26 | 0.00 |
|  |  | Males | 97.78 | 0.00 | 0.00 | 0.00 | 2.22 |
|  |  | Non-binary gender | 100.00 | 0.00 | 0.00 | 0.00 | 0.00 |
|  |  | Total | 95.74 | 1.06 | 0.00 | 2.13 | 1.06 |
|  | B | Females | 94.64 | 1.79 | 1.79 | 0.00 | 1.79 |
|  |  | Males | 90.00 | 3.33 | 6.67 | 0.00 | 0.00 |
|  |  | Non-binary gender |  |  |  |  |  |
|  |  | Total | 93.02 | 2.33 | 3.49 | 0.00 | 1.16 |
|  | C | Females | 69.49 | 6.78 | 18.64 | 0.00 | 5.08 |
|  |  | Males | 77.78 | 14.81 | 3.70 | 0.00 | 3.70 |
|  |  | Non-binary gender |  |  |  |  |  |
|  |  | Total | 72.09 | 9.30 | 13.95 | 0.00 | 4.65 |
| **Croatia** | A | Females | 95.89 | 1.73 | 1.95 | 0.43 | 0.00 |
|  |  | Males | 93.75 | 2.50 | 2.50 | 1.25 | 0.00 |
|  |  | Non-binary gender | 100.00 | 0.00 | 0.00 | 0.00 | 0.00 |
|  |  | Total | 95.58 | 1.84 | 2.03 | 0.55 | 0.00 |
|  | B | Females | 87.77 | 3.71 | 4.80 | 1.75 | 1.97 |
|  |  | Males | 91.62 | 4.19 | 1.80 | 1.80 | 0.60 |
|  |  | Non-binary gender | 100.00 | 0.00 | 0.00 | 0.00 | 0.00 |
|  |  | Total | 88.91 | 3.80 | 3.96 | 1.74 | 1.58 |
|  | C | Females | 94.91 | 2.10 | 2.69 | 0.30 | 0.00 |
|  |  | Males | 94.00 | 0.00 | 4.00 | 0.00 | 2.00 |
|  |  | Non-binary gender | 100.00 | 0.00 | 0.00 | 0.00 | 0.00 |
|  |  | Total | 94.82 | 1.81 | 2.85 | 0.26 | 0.26 |
| **Georgia** | A | Females | 85.47 | 7.82 | 5.31 | 0.84 | 0.56 |
|  |  | Males | 71.17 | 7.36 | 9.82 | 1.84 | 9.82 |
|  |  | Non-binary gender | 100.00 | 0.00 | 0.00 | 0.00 | 0.00 |
|  |  | Total | 81.07 | 7.65 | 6.69 | 1.15 | 3.44 |
|  | B | Females | 85.23 | 9.40 | 4.70 | 0.00 | 0.67 |
|  |  | Males | 78.48 | 10.13 | 5.06 | 2.53 | 3.80 |
|  |  | Non-binary gender | 100.00 | 0.00 | 0.00 | 0.00 | 0.00 |
|  |  | Total | 83.12 | 9.52 | 4.76 | 0.87 | 1.73 |
|  | C | Females | 85.86 | 5.76 | 4.71 | 2.62 | 1.05 |
|  |  | Males | 94.74 | 0.00 | 5.26 | 0.00 | 0.00 |
|  |  | Non-binary gender | 100.00 | 0.00 | 0.00 | 0.00 | 0.00 |
|  |  | Total | 87.39 | 4.78 | 4.78 | 2.17 | 0.87 |
| **Greece** | A | Females | 97.14 | 1.76 | 0.66 | 0.22 | 0.22 |
|  |  | Males | 96.79 | 1.07 | 0.71 | 0.36 | 1.07 |
|  |  | Non-binary gender | 100.00 | 0.00 | 0.00 | 0.00 | 0.00 |
|  |  | Total | 97.09 | 1.58 | 0.67 | 0.25 | 0.42 |
|  | B | Females | 97.45 | 1.11 | 0.88 | 0.00 | 0.56 |
|  |  | Males | 97.31 | 0.73 | 0.12 | 0.37 | 1.47 |
|  |  | Non-binary gender | 92.59 | 3.70 | 0.00 | 0.00 | 3.70 |
|  |  | Total | 97.34 | 1.00 | 0.57 | 0.14 | 0.95 |
|  | C | Females | 95.93 | 1.58 | 2.15 | 0.17 | 0.17 |
|  |  | Males | 96.34 | 1.13 | 1.13 | 0.56 | 0.85 |
|  |  | Non-binary gender | 100.00 | 0.00 | 0.00 | 0.00 | 0.00 |
|  |  | Total | 96.05 | 1.49 | 1.95 | 0.23 | 0.28 |
| **India** | A | Females | 53.77 | 21.70 | 18.87 | 2.83 | 2.83 |
|  |  | Males | 56.41 | 17.95 | 20.51 | 5.13 | 0.00 |
|  |  | Non-binary gender |  |  |  |  |  |
|  |  | Total | 54.48 | 20.69 | 19.31 | 3.45 | 2.07 |
|  | B | Females | 69.30 | 4.39 | 20.18 | 5.26 | 0.88 |
|  |  | Males | 73.77 | 3.28 | 21.31 | 1.64 | 0.00 |
|  |  | Non-binary gender |  |  |  |  |  |
|  |  | Total | 71.61 | 3.81 | 20.76 | 3.39 | 0.42 |
|  | C | Females | 74.71 | 8.05 | 16.09 | 0.00 | 1.15 |
|  |  | Males | 71.15 | 7.69 | 17.31 | 1.92 | 1.92 |
|  |  | Non-binary gender | 0.00 | 0.00 | 0.00 | 100.00 | 0.00 |
|  |  | Total | 72.86 | 7.86 | 16.43 | 1.43 | 1.43 |
| **Latvia** | A | Females | 94.43 | 2.93 | 2.64 | 0.00 | 0.00 |
|  |  | Males | 97.50 | 0.00 | 2.50 | 0.00 | 0.00 |
|  |  | Non-binary gender | 100.00 | 0.00 | 0.00 | 0.00 | 0.00 |
|  |  | Total | 94.82 | 2.59 | 2.59 | 0.00 | 0.00 |
|  | B | Females | 92.61 | 4.93 | 2.46 | 0.00 | 0.00 |
|  |  | Males | 95.12 | 0.81 | 2.44 | 0.00 | 1.63 |
|  |  | Non-binary gender | 100.00 | 0.00 | 0.00 | 0.00 | 0.00 |
|  |  | Total | 93.60 | 3.35 | 2.44 | 0.00 | 0.61 |
|  | C | Females | 90.14 | 3.17 | 6.34 | 0.00 | 0.35 |
|  |  | Males | 91.84 | 0.00 | 4.08 | 2.04 | 2.04 |
|  |  | Non-binary gender |  |  |  |  |  |
|  |  | Total | 90.39 | 2.70 | 6.01 | 0.30 | 0.60 |
| **Lithuania** | A | Females | 96.81 | 1.60 | 1.06 | 0.53 | 0.00 |
|  |  | Males | 97.06 | 0.00 | 0.00 | 0.00 | 2.94 |
|  |  | Non-binary gender | 100.00 | 0.00 | 0.00 | 0.00 | 0.00 |
|  |  | Total | 96.89 | 1.33 | 0.89 | 0.44 | 0.44 |
|  | B | Females | 96.97 | 0.00 | 3.03 | 0.00 | 0.00 |
|  |  | Males | 87.50 | 0.00 | 12.50 | 0.00 | 0.00 |
|  |  | Non-binary gender |  |  |  |  |  |
|  |  | Total | 95.12 | 0.00 | 4.88 | 0.00 | 0.00 |
|  | C | Females | 89.29 | 3.57 | 7.14 | 0.00 | 0.00 |
|  |  | Males | 100.00 | 0.00 | 0.00 | 0.00 | 0.00 |
|  |  | Non-binary gender |  |  |  |  |  |
|  |  | Total | 91.30 | 2.90 | 5.80 | 0.00 | 0.00 |
| **Nigeria** | A | Females | 92.65 | 2.94 | 4.41 | 0.00 | 0.00 |
|  |  | Males | 78.43 | 5.88 | 11.76 | 3.92 | 0.00 |
|  |  | Non-binary gender |  |  |  |  |  |
|  |  | Total | 86.55 | 4.20 | 7.56 | 1.68 | 0.00 |
|  | B | Females | 95.96 | 2.02 | 1.01 | 1.01 | 0.00 |
|  |  | Males | 90.15 | 2.27 | 3.79 | 2.27 | 1.52 |
|  |  | Non-binary gender |  |  |  |  |  |
|  |  | Total | 92.64 | 2.16 | 2.60 | 1.73 | 0.87 |
|  | C | Females | 90.53 | 3.16 | 5.26 | 0.00 | 1.05 |
|  |  | Males | 88.65 | 8.51 | 2.13 | 0.71 | 0.00 |
|  |  | Non-binary gender | 0.00 | 0.00 | 100.00 | 0.00 | 0.00 |
|  |  | Total | 89.03 | 6.33 | 3.80 | 0.42 | 0.42 |
| **Russia** | A | Females | 89.37 | 1.50 | 6.59 | 1.35 | 1.20 |
|  |  | Males | 91.04 | 2.99 | 3.73 | 1.49 | 0.75 |
|  |  | Non-binary gender | 68.75 | 6.25 | 6.25 | 12.50 | 6.25 |
|  |  | Total | 89.24 | 1.83 | 6.11 | 1.59 | 1.22 |
|  | B | Females | 79.69 | 6.25 | 7.03 | 2.34 | 4.69 |
|  |  | Males | 74.79 | 5.04 | 13.45 | 1.68 | 5.04 |
|  |  | Non-binary gender | 71.43 | 0.00 | 0.00 | 14.29 | 14.29 |
|  |  | Total | 77.17 | 5.51 | 9.84 | 2.36 | 5.12 |
|  | C | Females | 82.49 | 5.84 | 9.73 | 1.56 | 0.39 |
|  |  | Males | 82.76 | 5.75 | 5.75 | 4.60 | 1.15 |
|  |  | Non-binary gender | 88.89 | 0.00 | 11.11 | 0.00 | 0.00 |
|  |  | Total | 82.72 | 5.67 | 8.78 | 2.27 | 0.57 |
| **Malaysia** | A | Females | 97.56 | 2.44 | 0.00 | 0.00 | 0.00 |
|  |  | Males | 69.23 | 15.38 | 0.00 | 15.38 | 0.00 |
|  |  | Non-binary gender | 100.00 | 0.00 | 0.00 | 0.00 | 0.00 |
|  |  | Total | 93.75 | 4.17 | 0.00 | 2.08 | 0.00 |
|  | B | Females | 75.00 | 9.38 | 6.25 | 6.25 | 3.13 |
|  |  | Males | 80.00 | 0.00 | 10.00 | 10.00 | 0.00 |
|  |  | Non-binary gender |  |  |  |  |  |
|  |  | Total | 76.19 | 7.14 | 7.14 | 7.14 | 2.38 |
|  | C | Females | 83.08 | 4.62 | 3.08 | 6.15 | 3.08 |
|  |  | Males | 78.57 | 0.00 | 14.29 | 7.14 | 0.00 |
|  |  | Non-binary gender | 100.00 | 0.00 | 0.00 | 0.00 | 0.00 |
|  |  | Total | 82.50 | 3.75 | 5.00 | 6.25 | 2.50 |
| **Hungary** | A | Females | 96.88 | 0.00 | 3.13 | 0.00 | 0.00 |
|  |  | Males | 100.00 | 0.00 | 0.00 | 0.00 | 0.00 |
|  |  | Non-binary gender |  |  |  |  |  |
|  |  | Total | 97.56 | 0.00 | 2.44 | 0.00 | 0.00 |
|  | B | Females | 100.00 | 0.00 | 0.00 | 0.00 | 0.00 |
|  |  | Males | 100.00 | 0.00 | 0.00 | 0.00 | 0.00 |
|  |  | Non-binary gender |  |  |  |  |  |
|  |  | Total | 100.00 | 0.00 | 0.00 | 0.00 | 0.00 |
|  | C | Females | 96.55 | 3.45 | 0.00 | 0.00 | 0.00 |
|  |  | Males | 100.00 | 0.00 | 0.00 | 0.00 | 0.00 |
|  |  | Non-binary gender |  |  |  |  |  |
|  |  | Total | 96.88 | 3.13 | 0.00 | 0.00 | 0.00 |
| **TOTAL** |  |  | 91.61 | 2.94 | 3.72 | 0.82 | 0.91 |

**Webtable 14**: rates of accepting conspiracy theories by country, type of studies and sex

|  | **I don’t believe**  **it at all** | **A little bit** | **Maybe** | **Much** | **Very much** | **F:M** | **F:O** | **M:O** |
| --- | --- | --- | --- | --- | --- | --- | --- | --- |
| **J1. Do you believe that the COVID-19 vaccine was ready even before the virus broke out and they conceal it from us for the benefit of pharmaceutical companies?** | | | | | | | | |
| **Females** | 56.09 | 12.40 | 22.66 | 4.98 | 3.88 | 0.94 | 0.89 | 0.94 |
| **Males** | 59.36 | 13.94 | 19.44 | 3.12 | 4.15 |  |  |  |
| **Non-binary gender** | 62.88 | 12.12 | 19.70 | 3.79 | 1.52 |  |  |  |
| **Total** | 57.03 | 12.81 | 21.77 | 4.47 | 3.92 |  |  |  |
| **J2. Do you believe that COVID-19 was created in a laboratory?** | | | | | | | | |
| **Females** | 29.84 | 19.58 | 27.97 | 12.27 | 10.35 | 0.80 | 0.65 | 0.81 |
| **Males** | 37.52 | 17.87 | 26.79 | 9.52 | 8.29 |  |  |  |
| **Non-binary gender** | 46.21 | 18.94 | 19.70 | 8.33 | 6.82 |  |  |  |
| **Total** | 32.06 | 19.12 | 27.57 | 11.49 | 9.76 |  |  |  |
| **J3. Do you think that COVID-19 was created to be used as a biochemical weapon for the extermination of the human population?** | | | | | | | | |
| **Females** | 48.42 | 16.50 | 19.93 | 8.50 | 6.65 | 0.83 | 0.82 | 0.99 |
| **Males** | 58.58 | 14.42 | 17.24 | 5.32 | 4.45 |  |  |  |
| **Non-binary gender** | 59.09 | 14.39 | 15.15 | 5.30 | 6.06 |  |  |  |
| **Total** | 51.24 | 15.92 | 19.17 | 7.62 | 6.05 |  |  |  |
| **J4. Do you believe that COVID-19 is related to the 5G technology antenna?** | | | | | | | | |
| **Females** | 83.22 | 6.38 | 7.49 | 1.71 | 1.20 | 0.98 | 0.96 | 0.98 |
| **Males** | 84.89 | 5.50 | 7.00 | 1.56 | 1.05 |  |  |  |
| **Non-binary gender** | 86.36 | 3.03 | 6.06 | 4.55 | 0.00 |  |  |  |
| **Total** | 83.70 | 6.11 | 7.34 | 1.70 | 1.15 |  |  |  |
| **J5. Do you believe that COVID-19 appeared accidentally from human contact with animals and it was something that generally happens and was generally expected?** | | | | | | | | |
| **Females** | 23.99 | 20.11 | 28.31 | 17.70 | 9.89 | 1.09 | 1.17 | 1.08 |
| **Males** | 22.11 | 17.84 | 27.04 | 20.40 | 12.62 |  |  |  |
| **Non-binary gender** | 20.45 | 15.91 | 33.33 | 9.85 | 20.45 |  |  |  |
| **Total** | 23.45 | 19.46 | 28.02 | 18.33 | 10.73 |  |  |  |
| **J6. Do you believe that COVID-19 has much lower mortality rate but there is misinformation and terror-inducing propaganda?** | | | | | | | | |
| **Females** | 30.07 | 19.99 | 20.73 | 15.22 | 13.98 | 0.95 | 0.86 | 0.91 |
| **Males** | 31.69 | 20.76 | 20.79 | 13.49 | 13.28 |  |  |  |
| **Non-binary gender** | 34.85 | 20.45 | 25.76 | 6.82 | 12.12 |  |  |  |
| **Total** | 30.55 | 20.20 | 20.80 | 14.67 | 13.78 |  |  |  |
| **J7. Do you think the recommended measures (e.g. wearing face masks, avoid gatherings, stay at home etc.) are an attempt to restrict human rights and lead to some kind of dictatorship rather than to keep the population safer from COVID-19?** | | | | | | | | |
| **Females** | 51.35 | 17.84 | 14.36 | 8.81 | 7.65 | 0.97 | 1.21 | 1.25 |
| **Males** | 52.90 | 18.14 | 15.41 | 6.64 | 6.91 |  |  |  |
| **Non-binary gender** | 42.42 | 21.97 | 12.88 | 10.61 | 12.12 |  |  |  |
| **Total** | 51.67 | 17.96 | 14.62 | 8.25 | 7.50 |  |  |  |
| **J8. Do you believe that COVID-19 outbreak is a deliberate creation of the world’s powerful leaders to create a global economic crisis?** | | | | | | | | |
| **Females** | 50.84 | 15.73 | 18.98 | 7.76 | 6.69 | 0.91 | 0.92 | 1.01 |
| **Males** | 56.08 | 16.37 | 16.58 | 5.50 | 5.47 |  |  |  |
| **Non-binary gender** | 55.30 | 20.45 | 12.88 | 6.06 | 5.30 |  |  |  |
| **Total** | 52.28 | 15.95 | 18.28 | 7.14 | 6.35 |  |  |  |
| **J9. Do you believe that COVID-19 is a sign of divine power to destroy our planet?** | | | | | | | | |
| **Females** | 81.40 | 7.84 | 7.60 | 1.86 | 1.30 | 1.02 | 1.06 | 1.04 |
| **Males** | 79.72 | 6.70 | 9.40 | 2.01 | 2.16 |  |  |  |
| **Non-binary gender** | 76.52 | 12.88 | 6.82 | 1.52 | 2.27 |  |  |  |
| **Total** | 80.90 | 7.59 | 8.07 | 1.90 | 1.54 |  |  |  |
| **J10. Do you believe that airplanes secretly spray people with various chemicals?** | | | | | | | | |
| **Females** | 79.06 | 9.07 | 8.29 | 2.24 | 1.34 | 0.99 | 1.02 | 1.04 |
| **Males** | 80.26 | 8.08 | 7.69 | 2.34 | 1.62 |  |  |  |
| **Non-binary gender** | 77.27 | 10.61 | 8.33 | 1.52 | 2.27 |  |  |  |
| **Total** | 79.36 | 8.83 | 8.13 | 2.26 | 1.43 |  |  |  |
| **J11. Do you think that vaccines in general are dangerous and should be avoided?** | | | | | | | | |
| **Females** | 62.43 | 18.12 | 12.04 | 4.35 | 3.06 | 0.91 | 0.98 | 1.08 |
| **Males** | 68.46 | 15.29 | 10.45 | 3.12 | 2.67 |  |  |  |
| **Non-binary gender** | 63.64 | 18.94 | 9.85 | 5.30 | 2.27 |  |  |  |
| **Total** | 64.05 | 17.37 | 11.60 | 4.04 | 2.95 |  |  |  |
| **J12. The government is secretly involved in the murder of innocent citizens and/or well-known public figures.** | | | | | | | | |
| **Females** | 58.36 | 17.50 | 14.85 | 5.31 | 3.99 | 1.00 | 1.26 | 1.27 |
| **Males** | 58.52 | 16.49 | 14.60 | 5.59 | 4.81 |  |  |  |
| **Non-binary gender** | 46.21 | 18.18 | 20.45 | 9.85 | 5.30 |  |  |  |
| **Total** | 58.27 | 17.24 | 14.84 | 5.43 | 4.22 |  |  |  |
| **J13. Global warming and climate change is a greatly exaggerated myth to serve various political and financial interests.** | | | | | | | | |
| **Females** | 79.92 | 9.07 | 7.67 | 2.02 | 1.32 | 1.09 | 0.95 | 0.87 |
| **Males** | 73.57 | 11.53 | 9.88 | 3.09 | 1.92 |  |  |  |
| **Non-binary gender** | 84.09 | 4.55 | 8.33 | 2.27 | 0.76 |  |  |  |
| **Total** | 78.27 | 9.68 | 8.27 | 2.31 | 1.47 |  |  |  |
| **J14. The power held by the heads of state is smaller than that of small unknown groups that really control the world of politics.** | | | | | | | | |
| **Females** | 41.02 | 21.76 | 21.22 | 9.44 | 6.56 | 1.01 | 0.93 | 0.92 |
| **Males** | 40.52 | 22.77 | 19.92 | 9.73 | 7.06 |  |  |  |
| **Non-binary gender** | 43.94 | 22.73 | 22.73 | 4.55 | 6.06 |  |  |  |
| **Total** | 40.92 | 22.04 | 20.89 | 9.47 | 6.69 |  |  |  |
| **J15. Secret organizations are communicating with aliens, but they hide it from the public.** | | | | | | | | |
| **Females** | 83.02 | 7.42 | 6.68 | 1.52 | 1.35 | 1.06 | 1.01 | 0.95 |
| **Males** | 78.01 | 9.10 | 8.92 | 1.65 | 2.31 |  |  |  |
| **Non-binary gender** | 81.82 | 6.82 | 8.33 | 1.52 | 1.52 |  |  |  |
| **Total** | 81.68 | 7.86 | 7.30 | 1.55 | 1.61 |  |  |  |
| **J16. Groups of scientists manipulate, invent or conceal evidence to deceive the public.** | | | | | | | | |
| **Females** | 46.93 | 24.49 | 17.87 | 6.29 | 4.42 | 0.95 | 1.03 | 1.09 |
| **Males** | 49.53 | 22.11 | 17.42 | 6.40 | 4.54 |  |  |  |
| **Non-binary gender** | 45.45 | 21.97 | 19.70 | 6.06 | 6.82 |  |  |  |
| **Total** | 47.61 | 23.83 | 17.77 | 6.32 | 4.48 |  |  |  |
| **J17. The government allows or commits acts of terrorism on its territory, disguising its involvement as if someone else is responsible.** | | | | | | | | |
| **Females** | 47.31 | 20.55 | 18.32 | 8.12 | 5.70 | 0.95 | 1.18 | 1.24 |
| **Males** | 49.59 | 21.00 | 15.50 | 7.75 | 6.16 |  |  |  |
| **Non-binary gender** | 40.15 | 22.73 | 13.64 | 9.85 | 13.64 |  |  |  |
| **Total** | 47.85 | 20.70 | 17.52 | 8.04 | 5.90 |  |  |  |
| **J18. Do you believe that secretly a chip will be included in the COVID-19 vaccine in order to mark people?** | | | | | | | | |
| **Females** | 75.18 | 9.82 | 10.04 | 2.81 | 2.15 | 0.98 | 1.01 | 1.04 |
| **Males** | 76.90 | 9.13 | 9.67 | 2.10 | 2.19 |  |  |  |
| **Non-binary gender** | 74.24 | 11.36 | 10.61 | 1.52 | 2.27 |  |  |  |
| **Total** | 75.63 | 9.65 | 9.95 | 2.61 | 2.16 |  |  |  |
| **J19. A small, secret group of people is responsible for taking all the important decisions, such as starting wars, in a planned way and the reasons are the group’s secret interests.** | | | | | | | | |
| **Females** | 51.12 | 20.40 | 16.75 | 6.53 | 5.20 | 0.95 | 0.96 | 1.02 |
| **Males** | 53.95 | 18.92 | 16.01 | 6.37 | 4.75 |  |  |  |
| **Non-binary gender** | 53.03 | 24.24 | 12.12 | 4.55 | 6.06 |  |  |  |
| **Total** | 51.90 | 20.05 | 16.51 | 6.46 | 5.09 |  |  |  |
| **J20. Technology and devices for mind control are used on people without their knowledge.** | | | | | | | | |
| **Females** | 60.02 | 15.67 | 14.26 | 5.89 | 4.16 | 0.91 | 1.00 | 1.10 |
| **Males** | 66.00 | 14.60 | 12.29 | 4.18 | 2.94 |  |  |  |
| **Non-binary gender** | 59.85 | 13.64 | 14.39 | 7.58 | 4.55 |  |  |  |
| **Total** | 61.61 | 15.36 | 13.74 | 5.45 | 3.84 |  |  |  |
| **J21. New and advanced technology that will make the existing industry obsolete is being suppressed in a malicious and violent way.** | | | | | | | | |
| **Females** | 54.78 | 20.51 | 17.00 | 4.96 | 2.75 | 1.00 | 1.03 | 1.04 |
| **Males** | 54.91 | 20.79 | 16.19 | 4.96 | 3.15 |  |  |  |
| **Non-binary gender** | 53.03 | 21.97 | 15.15 | 7.58 | 2.27 |  |  |  |
| **Total** | 54.80 | 20.60 | 16.76 | 4.99 | 2.85 |  |  |  |
| **J22. Some important events happen due to the activity of a small group who secretly manipulate world events.** | | | | | | | | |
| **Females** | 49.71 | 21.67 | 17.55 | 6.71 | 4.35 | 0.97 | 0.87 | 0.90 |
| **Males** | 51.34 | 20.40 | 17.18 | 5.92 | 5.17 |  |  |  |
| **Non-binary gender** | 56.82 | 18.18 | 15.91 | 3.03 | 6.06 |  |  |  |
| **Total** | 50.22 | 21.30 | 17.44 | 6.46 | 4.59 |  |  |  |
| **J23. Experiments involving new drugs or technologies are performed systematically on humans in a secret way and without their knowledge or consent.** | | | | | | | | |
| **Females** | 54.91 | 21.01 | 16.06 | 4.94 | 3.08 | 0.97 | 1.04 | 1.07 |
| **Males** | 56.74 | 21.15 | 15.05 | 4.54 | 2.52 |  |  |  |
| **Non-binary gender** | 53.03 | 22.73 | 15.15 | 5.30 | 3.79 |  |  |  |
| **Total** | 55.38 | 21.06 | 15.78 | 4.84 | 2.94 |  |  |  |
| **J24. Many important pieces of information are deliberately hidden from the public for reasons of interest.** | | | | | | | | |
| **Females** | 16.37 | 19.72 | 24.76 | 19.02 | 20.12 | 0.77 | 0.86 | 1.12 |
| **Males** | 21.21 | 20.82 | 25.02 | 15.65 | 17.30 |  |  |  |
| **Non-binary gender** | 18.94 | 21.97 | 25.76 | 13.64 | 19.70 |  |  |  |
| **Total** | 17.68 | 20.04 | 24.84 | 18.07 | 19.37 |  |  |  |
| **J25. The spread of certain viruses and/or diseases is the result of deliberate, covert actions of an organization or government.** | | | | | | | | |
| **Females** | 52.44 | 18.74 | 17.63 | 6.05 | 5.14 | 0.90 | 0.92 | 1.02 |
| **Males** | 58.10 | 18.80 | 14.45 | 4.60 | 4.06 |  |  |  |
| **Non-binary gender** | 56.82 | 22.73 | 12.12 | 5.30 | 3.03 |  |  |  |
| **Total** | 54.00 | 18.80 | 16.72 | 5.65 | 4.83 |  |  |  |
| **J26. It is possible that the earth is flat rather than spherical.** | | | | | | | | |
| **Females** | 92.12 | 2.96 | 3.66 | 0.65 | 0.61 | 1.02 | 1.01 | 0.99 |
| **Males** | 90.24 | 2.94 | 3.97 | 1.17 | 1.68 |  |  |  |
| **Non-binary gender** | 90.91 | 1.52 | 2.27 | 3.03 | 2.27 |  |  |  |
| **Total** | 91.61 | 2.94 | 3.72 | 0.82 | 0.91 |  |  |  |
| **Average*** |  |  |  |  |  | 0.95 | 0.98 | 1.03 |

**Webtable 15**: rates of accepting conspiracy theories by sex

* J5 excluded

There were no differences across all 26 conspiracy beliefs between males and females (F:M=0.98 on average) although there were differences concerning individual beliefs

For comparison reasons, the YouCov studies suggested that 82.5% of millennials reject the flat earth hypothesis while 4% firmly believe it is flat ^7^.

|  | **I don’t**  **believe**  **it at all** | **A little bit** | **Maybe** | **Much** | **Very**  **much** | **A:B** | **A:C** | **B:C** |
| --- | --- | --- | --- | --- | --- | --- | --- | --- |
| **J1. Do you believe that the COVID-19 vaccine was ready even before the virus broke out and they conceal it from us for the benefit of pharmaceutical companies?** | | | | | | | | |
| **A** | 64.92 | 11.54 | 17.74 | 3.46 | 2.34 | 1.19 | 1.27 | 1.07 |
| **B** | 54.74 | 12.27 | 23.00 | 5.08 | 4.91 |  |  |  |
| **C** | 51.31 | 14.64 | 24.63 | 4.88 | 4.54 |  |  |  |
| **Total** | 57.03 | 12.81 | 21.77 | 4.47 | 3.92 |  |  |  |
| **J2. Do you believe that COVID-19 was created in a laboratory?** | | | | | | | | |
| **A** | 36.54 | 19.87 | 26.35 | 10.06 | 7.18 | 1.17 | 1.29 | 1.10 |
| **B** | 31.24 | 17.66 | 27.48 | 12.51 | 11.11 |  |  |  |
| **C** | 28.31 | 19.84 | 28.90 | 11.91 | 11.03 |  |  |  |
| **Total** | 32.06 | 19.12 | 27.57 | 11.49 | 9.76 |  |  |  |
| **J3. Do you think that COVID-19 was created to be used as a biochemical weapon for the extermination of the human population?** | | | | | | | | |
| **A** | 55.71 | 15.67 | 17.58 | 6.73 | 4.32 | 1.08 | 1.20 | 1.11 |
| **B** | 51.45 | 15.16 | 19.61 | 6.91 | 6.86 |  |  |  |
| **C** | 46.45 | 16.96 | 20.33 | 9.25 | 7.01 |  |  |  |
| **Total** | 51.24 | 15.92 | 19.17 | 7.62 | 6.05 |  |  |  |
|  | | | | | | | | |
| **J4. Do you believe that COVID-19 is related to the 5G technology antenna?** | | | | | | | | |
| **A** | 85.52 | 5.77 | 6.82 | 1.14 | 0.74 | 1.01 | 1.05 | 1.04 |
| **B** | 84.29 | 5.84 | 6.58 | 1.81 | 1.48 |  |  |  |
| **C** | 81.23 | 6.74 | 8.66 | 2.15 | 1.22 |  |  |  |
| **Total** | 83.70 | 6.11 | 7.34 | 1.70 | 1.15 |  |  |  |
| **J5. Do you believe that COVID-19 appeared accidentally from human contact with animals and it was something that generally happens and was generally expected?** | | | | | | | | |
| **A** | 19.10 | 17.93 | 27.21 | 22.18 | 13.57 | 0.80 | 0.69 | 0.86 |
| **B** | 23.78 | 20.35 | 27.93 | 17.59 | 10.34 |  |  |  |
| **C** | 27.56 | 20.11 | 28.95 | 15.16 | 8.23 |  |  |  |
| **Total** | 23.45 | 19.46 | 28.02 | 18.33 | 10.73 |  |  |  |
| **J6. Do you believe that COVID-19 has much lower mortality rate but there is misinformation and terror-inducing propaganda?** | | | | | | | | |
|  |  |  |  |  |  |  |  |  |
| **A** | 38.66 | 19.72 | 19.53 | 11.92 | 10.16 | 1.52 | 1.40 | 0.92 |
| **B** | 25.38 | 21.31 | 21.85 | 15.85 | 15.61 |  |  |  |
| **C** | 27.56 | 19.55 | 21.02 | 16.28 | 15.60 |  |  |  |
| **Total** | 30.55 | 20.20 | 20.80 | 14.67 | 13.78 |  |  |  |
| **J7. Do you think the recommended measures (e.g. wearing face masks. avoid gatherings. stay at home etc.) are an attempt to restrict human rights and lead to some kind of dictatorship rather than to keep the population safer from COVID-19?** | | | | | | | | |
| **A** | 60.96 | 15.79 | 11.88 | 6.27 | 5.10 | 1.31 | 1.28 | 0.98 |
| **B** | 46.50 | 19.69 | 16.47 | 8.53 | 8.82 |  |  |  |
| **C** | 47.45 | 18.43 | 15.55 | 9.98 | 8.59 |  |  |  |
| **Total** | 51.67 | 17.96 | 14.62 | 8.25 | 7.50 |  |  |  |
| **J8. Do you believe that COVID-19 outbreak is a deliberate creation of the world’s powerful leaders to create a global economic crisis?** | | | | | | | | |
| **A** | 57.91 | 15.53 | 15.98 | 6.08 | 4.51 | 1.13 | 1.21 | 1.07 |
| **B** | 51.10 | 15.80 | 18.61 | 7.01 | 7.48 |  |  |  |
| **C** | 47.74 | 16.55 | 20.28 | 8.35 | 7.08 |  |  |  |
| **Total** | 52.28 | 15.95 | 18.28 | 7.14 | 6.35 |  |  |  |
| **J9. Do you believe that COVID-19 is a sign of divine power to destroy our planet?** | | | | | | | | |
| **A** | 81.40 | 7.84 | 7.60 | 1.86 | 1.30 | 1.02 | 1.06 | 1.04 |
| **B** | 79.72 | 6.70 | 9.40 | 2.01 | 2.16 |  |  |  |
| **C** | 76.52 | 12.88 | 6.82 | 1.52 | 2.27 |  |  |  |
| **Total** | 80.90 | 7.59 | 8.07 | 1.90 | 1.54 |  |  |  |
| **J10. Do you believe that airplanes secretly spray people with various chemicals?** | | | | | | | | |
| **A** | 79.06 | 9.07 | 8.29 | 2.24 | 1.34 | 0.99 | 1.02 | 1.04 |
| **B** | 80.26 | 8.08 | 7.69 | 2.34 | 1.62 |  |  |  |
| **C** | 77.27 | 10.61 | 8.33 | 1.52 | 2.27 |  |  |  |
| **Total** | 79.36 | 8.83 | 8.13 | 2.26 | 1.43 |  |  |  |
| **J11. Do you think that vaccines in general are dangerous and should be avoided?** | | | | | | | | |
| **A** | 62.43 | 18.12 | 12.04 | 4.35 | 3.06 | 0.91 | 0.98 | 1.08 |
| **B** | 68.46 | 15.29 | 10.45 | 3.12 | 2.67 |  |  |  |
| **C** | 63.64 | 18.94 | 9.85 | 5.30 | 2.27 |  |  |  |
| **Total** | 64.05 | 17.37 | 11.60 | 4.04 | 2.95 |  |  |  |
| **J12. The government is secretly involved in the murder of innocent citizens and/or well-known public figures.** | | | | | | | | |
| **A** | 58.36 | 17.50 | 14.85 | 5.31 | 3.99 | 1.00 | 1.26 | 1.27 |
| **B** | 58.52 | 16.49 | 14.60 | 5.59 | 4.81 |  |  |  |
| **C** | 46.21 | 18.18 | 20.45 | 9.85 | 5.30 |  |  |  |
| **Total** | 58.27 | 17.24 | 14.84 | 5.43 | 4.22 |  |  |  |
| **J13. Global warming and climate change is a greatly exaggerated myth to serve various political and financial interests.** | | | | | | | | |
| **A** | 79.92 | 9.07 | 7.67 | 2.02 | 1.32 | 1.09 | 0.95 | 0.87 |
| **B** | 73.57 | 11.53 | 9.88 | 3.09 | 1.92 |  |  |  |
| **C** | 84.09 | 4.55 | 8.33 | 2.27 | 0.76 |  |  |  |
| **Total** | 78.27 | 9.68 | 8.27 | 2.31 | 1.47 |  |  |  |
| **J14. The power held by the heads of state is smaller than that of small unknown groups that really control the world of politics.** | | | | | | | | |
| **A** | 41.02 | 21.76 | 21.22 | 9.44 | 6.56 | 1.01 | 0.93 | 0.92 |
| **B** | 40.52 | 22.77 | 19.92 | 9.73 | 7.06 |  |  |  |
| **C** | 43.94 | 22.73 | 22.73 | 4.55 | 6.06 |  |  |  |
| **Total** | 40.92 | 22.04 | 20.89 | 9.47 | 6.69 |  |  |  |
| **J15. Secret organizations are communicating with aliens. but they hide it from the public.** | | | | | | | | |
| **A** | 83.02 | 7.42 | 6.68 | 1.52 | 1.35 | 1.06 | 1.01 | 0.95 |
| **B** | 78.01 | 9.10 | 8.92 | 1.65 | 2.31 |  |  |  |
| **C** | 81.82 | 6.82 | 8.33 | 1.52 | 1.52 |  |  |  |
| **Total** | 81.68 | 7.86 | 7.30 | 1.55 | 1.61 |  |  |  |
| **J16. Groups of scientists manipulate. invent or conceal evidence to deceive the public.** | | | | | | | | |
| **A** | 46.93 | 24.49 | 17.87 | 6.29 | 4.42 | 0.95 | 1.03 | 1.09 |
| **B** | 49.53 | 22.11 | 17.42 | 6.40 | 4.54 |  |  |  |
| **C** | 45.45 | 21.97 | 19.70 | 6.06 | 6.82 |  |  |  |
| **Total** | 47.61 | 23.83 | 17.77 | 6.32 | 4.48 |  |  |  |
| **J17. The government allows or commits acts of terrorism on its territory. disguising its involvement as if someone else is responsible.** | | | | | | | | |
| **A** | 47.31 | 20.55 | 18.32 | 8.12 | 5.70 | 0.95 | 1.18 | 1.24 |
| **B** | 49.59 | 21.00 | 15.50 | 7.75 | 6.16 |  |  |  |
| **C** | 40.15 | 22.73 | 13.64 | 9.85 | 13.64 |  |  |  |
| **Total** | 47.85 | 20.70 | 17.52 | 8.04 | 5.90 |  |  |  |
| **J18. Do you believe that secretly a chip will be included in the COVID-19 vaccine in order to mark people?** | | | | | | | | |
| **A** | 75.18 | 9.82 | 10.04 | 2.81 | 2.15 | 0.98 | 1.01 | 1.04 |
| **B** | 76.90 | 9.13 | 9.67 | 2.10 | 2.19 |  |  |  |
| **C** | 74.24 | 11.36 | 10.61 | 1.52 | 2.27 |  |  |  |
| **Total** | 75.63 | 9.65 | 9.95 | 2.61 | 2.16 |  |  |  |
| **J19. A small. secret group of people is responsible for taking all the important decisions. such as starting wars. in a planned way and the reasons are the group’s secret interests.** | | | | | | | | |
| **A** | 51.12 | 20.40 | 16.75 | 6.53 | 5.20 | 0.95 | 0.96 | 1.02 |
| **B** | 53.95 | 18.92 | 16.01 | 6.37 | 4.75 |  |  |  |
| **C** | 53.03 | 24.24 | 12.12 | 4.55 | 6.06 |  |  |  |
| **Total** | 51.90 | 20.05 | 16.51 | 6.46 | 5.09 |  |  |  |
| **J20. Technology and devices for mind control are used on people without their knowledge.** | | | | | | | | |
|  | | | | | | | | |
| **A** | 60.02 | 15.67 | 14.26 | 5.89 | 4.16 | 0.91 | 1.00 | 1.10 |
| **B** | 66.00 | 14.60 | 12.29 | 4.18 | 2.94 |  |  |  |
| **C** | 59.85 | 13.64 | 14.39 | 7.58 | 4.55 |  |  |  |
| **Total** | 61.61 | 15.36 | 13.74 | 5.45 | 3.84 |  |  |  |
| **J21. New and advanced technology that will make the existing industry obsolete is being suppressed in a malicious and violent way.** | | | | | | | | |
| **A** | 54.78 | 20.51 | 17.00 | 4.96 | 2.75 | 1.00 | 1.03 | 1.04 |
| **B** | 54.91 | 20.79 | 16.19 | 4.96 | 3.15 |  |  |  |
| **C** | 53.03 | 21.97 | 15.15 | 7.58 | 2.27 |  |  |  |
| **Total** | 54.80 | 20.60 | 16.76 | 4.99 | 2.85 |  |  |  |
| **J22. Some important events happen due to the activity of a small group who secretly manipulate world events.** | | | | | | | | |
| **A** | 49.71 | 21.67 | 17.55 | 6.71 | 4.35 | 0.97 | 0.87 | 0.90 |
| **B** | 51.34 | 20.40 | 17.18 | 5.92 | 5.17 |  |  |  |
| **C** | 56.82 | 18.18 | 15.91 | 3.03 | 6.06 |  |  |  |
| **Total** | 50.22 | 21.30 | 17.44 | 6.46 | 4.59 |  |  |  |
| **J23. Experiments involving new drugs or technologies are performed systematically on humans in a secret way and without their knowledge or consent.** | | | | | | | | |
| **A** | 54.91 | 21.01 | 16.06 | 4.94 | 3.08 | 0.97 | 1.04 | 1.07 |
| **B** | 56.74 | 21.15 | 15.05 | 4.54 | 2.52 |  |  |  |
| **C** | 53.03 | 22.73 | 15.15 | 5.30 | 3.79 |  |  |  |
| **Total** | 55.38 | 21.06 | 15.78 | 4.84 | 2.94 |  |  |  |
| **J24. Many important pieces of information are deliberately hidden from the public for reasons of interest.** | | | | | | | | |
| **A** | 16.37 | 19.72 | 24.76 | 19.02 | 20.12 | 0.77 | 0.86 | 1.12 |
| **B** | 21.21 | 20.82 | 25.02 | 15.65 | 17.30 |  |  |  |
| **C** | 18.94 | 21.97 | 25.76 | 13.64 | 19.70 |  |  |  |
| **Total** | 17.68 | 20.04 | 24.84 | 18.07 | 19.37 |  |  |  |
| **J25. The spread of certain viruses and/or diseases is the result of deliberate. covert actions of an organization or government.** | | | | | | | | |
| **A** | 52.44 | 18.74 | 17.63 | 6.05 | 5.14 | 0.90 | 0.92 | 1.02 |
| **B** | 58.10 | 18.80 | 14.45 | 4.60 | 4.06 |  |  |  |
| **C** | 56.82 | 22.73 | 12.12 | 5.30 | 3.03 |  |  |  |
| **Total** | 54.00 | 18.80 | 16.72 | 5.65 | 4.83 |  |  |  |
| **J26. It is possible that the earth is flat rather than spherical.** | | | | | | | | |
| **A** | 92.12 | 2.96 | 3.66 | 0.65 | 0.61 | 1.02 | 1.01 | 0.99 |
| **B** | 90.24 | 2.94 | 3.97 | 1.17 | 1.68 |  |  |  |
| **C** | 90.91 | 1.52 | 2.27 | 3.03 | 2.27 |  |  |  |
| **Total** | 91.61 | 2.94 | 3.72 | 0.82 | 0.91 |  |  |  |
| **Average*** |  |  |  |  |  | 1.03 | 1.07 | 1.04 |

**Webtable 16**: rates of accepting conspiracy theories by type of studies

* J5 excluded

|  | **I don’t**  **believe**  **it at all** | | **A little bit** | | **Maybe** | | **Much** | | **Very**  **much** | |  |
| --- | --- | --- | --- | --- | --- | --- | --- | --- | --- | --- | --- |
|  | **N** | % | **N** | % | **N** | % | **N** | % | **N** | % |  |
| **J1. Do you believe that the COVID-19 vaccine was ready even before the virus broke out and they conceal it from us for the benefit of pharmaceutical companies?** | | | | | | | | | | | |
| **All the rest** | 5020 | 53.90 | 1254 | 13.47 | 2165 | 23.25 | 446 | 4.79 | 428 | 4.60 | 1.00 |
| **Medicine** | 1399 | 70.55 | 197 | 9.93 | 288 | 14.52 | 62 | 3.13 | 37 | 1.87 | 1.31 |
| **Nursing** | 96 | 44.04 | 39 | 17.89 | 64 | 29.36 | 11 | 5.05 | 8 | 3.67 | 0.82 |
| **Pharmacy** | 210 | 73.17 | 22 | 7.67 | 45 | 15.68 | 7 | 2.44 | 3 | 1.05 | 1.36 |
| **Dentistry** | 238 | 65.56 | 37 | 10.19 | 69 | 19.01 | 12 | 3.31 | 7 | 1.93 | 1.22 |
| **Fine arts** | 158 | 49.07 | 50 | 15.53 | 87 | 27.02 | 20 | 6.21 | 7 | 2.17 | 0.91 |
| **J2. Do you believe that COVID-19 was created in a laboratory?** | | | | | | | | | | | |
| **All the rest** | 2832 | 30.41 | 1752 | 18.81 | 2600 | 27.92 | 1120 | 12.03 | 1009 | 10.83 | 1.00 |
| **Medicine** | 779 | 39.28 | 396 | 19.97 | 499 | 25.16 | 187 | 9.43 | 122 | 6.15 | 1.29 |
| **Nursing** | 53 | 24.31 | 51 | 23.39 | 65 | 29.82 | 27 | 12.39 | 22 | 10.09 | 0.80 |
| **Pharmacy** | 109 | 37.98 | 51 | 17.77 | 80 | 27.87 | 30 | 10.45 | 17 | 5.92 | 1.25 |
| **Dentistry** | 124 | 34.16 | 73 | 20.11 | 104 | 28.65 | 40 | 11.02 | 22 | 6.06 | 1.12 |
| **Fine arts** | 106 | 32.92 | 64 | 19.88 | 94 | 29.19 | 31 | 9.63 | 27 | 8.39 | 1.08 |
| **J3. Do you think that COVID-19 was created to be used as a biochemical weapon for the extermination of the human population?** | | | | | | | | | | | |
| **All the rest** | 4653 | 49.96 | 1480 | 15.89 | 1818 | 19.52 | 740 | 7.95 | 622 | 6.68 | 1.00 |
| **Medicine** | 1136 | 57.29 | 315 | 15.89 | 329 | 16.59 | 121 | 6.10 | 82 | 4.14 | 1.15 |
| **Nursing** | 74 | 33.94 | 47 | 21.56 | 65 | 29.82 | 19 | 8.72 | 13 | 5.96 | 0.68 |
| **Pharmacy** | 173 | 60.28 | 42 | 14.63 | 43 | 14.98 | 18 | 6.27 | 11 | 3.83 | 1.21 |
| **Dentistry** | 190 | 52.34 | 54 | 14.88 | 75 | 20.66 | 30 | 8.26 | 14 | 3.86 | 1.05 |
| **Fine arts** | 172 | 53.42 | 50 | 15.53 | 63 | 19.57 | 23 | 7.14 | 14 | 4.35 | 1.07 |
| **J4. Do you believe that COVID-19 is related to the 5G technology antenna?** | | | | | | | | | | | |
| **All the rest** | 7749 | 83.21 | 572 | 6.14 | 693 | 7.44 | 178 | 1.91 | 121 | 1.30 | 1.00 |
| **Medicine** | 1738 | 87.64 | 101 | 5.09 | 116 | 5.85 | 16 | 0.81 | 12 | 0.61 | 1.05 |
| **Nursing** | 126 | 57.80 | 39 | 17.89 | 42 | 19.27 | 9 | 4.13 | 2 | 0.92 | 0.69 |
| **Pharmacy** | 255 | 88.85 | 13 | 4.53 | 16 | 5.57 | 1 | 0.35 | 2 | 0.70 | 1.07 |
| **Dentistry** | 305 | 84.02 | 21 | 5.79 | 29 | 7.99 | 4 | 1.10 | 4 | 1.10 | 1.01 |
| **Fine arts** | 278 | 86.34 | 17 | 5.28 | 21 | 6.52 | 4 | 1.24 | 2 | 0.62 | 1.04 |
| **J5. Do you believe that COVID-19 appeared accidentally from human contact with animals and it was something that generally happens and was generally expected?** | | | | | | | | | | | |
| **All the rest** | 2324 | 24.95 | 1870 | 20.08 | 2616 | 28.09 | 1585 | 17.02 | 918 | 9.86 | 1.00 |
| **Medicine** | 352 | 17.75 | 327 | 16.49 | 525 | 26.48 | 473 | 23.85 | 306 | 15.43 | 0.71 |
| **Nursing** | 57 | 26.15 | 47 | 21.56 | 81 | 37.16 | 19 | 8.72 | 14 | 6.42 | 1.05 |
| **Pharmacy** | 52 | 18.12 | 60 | 20.91 | 65 | 22.65 | 74 | 25.78 | 36 | 12.54 | 0.73 |
| **Dentistry** | 66 | 18.18 | 72 | 19.83 | 118 | 32.51 | 80 | 22.04 | 27 | 7.44 | 0.73 |
| **Fine arts** | 77 | 23.91 | 54 | 16.77 | 94 | 29.19 | 58 | 18.01 | 39 | 12.11 | 0.96 |
| **J6. Do you believe that COVID-19 has much lower mortality rate but there is misinformation and terror-inducing propaganda?** | | | | | | | | | | | |
| **All the rest** | 2440 | 26.20 | 1920 | 20.62 | 1999 | 21.46 | 1487 | 15.97 | 1467 | 15.75 | 1.00 |
| **Medicine** | 981 | 49.47 | 360 | 18.15 | 333 | 16.79 | 181 | 9.13 | 128 | 6.45 | 1.89 |
| **Nursing** | 69 | 31.65 | 51 | 23.39 | 64 | 29.36 | 21 | 9.63 | 13 | 5.96 | 1.21 |
| **Pharmacy** | 95 | 33.10 | 57 | 19.86 | 60 | 20.91 | 41 | 14.29 | 34 | 11.85 | 1.26 |
| **Dentistry** | 146 | 40.22 | 72 | 19.83 | 80 | 22.04 | 41 | 11.29 | 24 | 6.61 | 1.54 |
| **Fine arts** | 84 | 26.09 | 62 | 19.25 | 61 | 18.94 | 61 | 18.94 | 54 | 16.77 | 1.00 |
| **J7. Do you think the recommended measures (e.g. wearing face masks, avoid gatherings, stay at home etc.) are an attempt to restrict human rights and lead to some kind of dictatorship rather than to keep the population safer from COVID-19?** | | | | | | | | | | | |
| **All the rest** | 4395 | 47.19 | 1792 | 19.24 | 1473 | 15.82 | 861 | 9.25 | 792 | 8.50 | 1.00 |
| **Medicine** | 1445 | 72.87 | 233 | 11.75 | 162 | 8.17 | 77 | 3.88 | 66 | 3.33 | 1.54 |
| **Nursing** | 103 | 47.25 | 40 | 18.35 | 41 | 18.81 | 20 | 9.17 | 14 | 6.42 | 1.00 |
| **Pharmacy** | 158 | 55.05 | 46 | 16.03 | 55 | 19.16 | 18 | 6.27 | 10 | 3.48 | 1.17 |
| **Dentistry** | 213 | 58.68 | 63 | 17.36 | 47 | 12.95 | 22 | 6.06 | 18 | 4.96 | 1.24 |
| **Fine arts** | 137 | 42.55 | 69 | 21.43 | 48 | 14.91 | 32 | 9.94 | 36 | 11.18 | 0.90 |
| **J8. Do you believe that COVID-19 outbreak is a deliberate creation of the world’s powerful leaders to create a global economic crisis?** | | | | | | | | | | | |
| **All the rest** | 4679 | 50.24 | 1499 | 16.10 | 1765 | 18.95 | 705 | 7.57 | 665 | 7.14 | 1.00 |
| **Medicine** | 1233 | 62.18 | 306 | 15.43 | 284 | 14.32 | 88 | 4.44 | 72 | 3.63 | 1.24 |
| **Nursing** | 95 | 43.58 | 40 | 18.35 | 54 | 24.77 | 19 | 8.72 | 10 | 4.59 | 0.87 |
| **Pharmacy** | 166 | 57.84 | 44 | 15.33 | 52 | 18.12 | 15 | 5.23 | 10 | 3.48 | 1.15 |
| **Dentistry** | 202 | 55.65 | 50 | 13.77 | 65 | 17.91 | 33 | 9.09 | 13 | 3.58 | 1.11 |
| **Fine arts** | 153 | 47.52 | 53 | 16.46 | 62 | 19.25 | 31 | 9.63 | 23 | 7.14 | 0.95 |
| **J9. Do you believe that COVID-19 is a sign of divine power to destroy our planet?** | | | | | | | | | | | |
| **All the rest** | 7490 | 80.43 | 712 | 7.65 | 778 | 8.35 | 174 | 1.87 | 159 | 1.71 | 1.00 |
| **Medicine** | 1657 | 83.56 | 136 | 6.86 | 123 | 6.20 | 40 | 2.02 | 27 | 1.36 | 1.04 |
| **Nursing** | 128 | 58.72 | 40 | 18.35 | 41 | 18.81 | 7 | 3.21 | 2 | 0.92 | 0.73 |
| **Pharmacy** | 244 | 85.02 | 17 | 5.92 | 21 | 7.32 | 5 | 1.74 | 0 | 0.00 | 1.06 |
| **Dentistry** | 293 | 80.72 | 28 | 7.71 | 33 | 9.09 | 7 | 1.93 | 2 | 0.55 | 1.00 |
| **Fine arts** | 289 | 89.75 | 15 | 4.66 | 12 | 3.73 | 4 | 1.24 | 2 | 0.62 | 1.12 |
| **J10. Do you believe that airplanes secretly spray people with various chemicals?** | | | | | | | | | | | |
| **All the rest** | 7233 | 77.67 | 900 | 9.66 | 793 | 8.51 | 237 | 2.54 | 150 | 1.61 | 1.00 |
| **Medicine** | 1729 | 87.19 | 109 | 5.50 | 108 | 5.45 | 19 | 0.96 | 18 | 0.91 | 1.12 |
| **Nursing** | 137 | 62.84 | 36 | 16.51 | 37 | 16.97 | 8 | 3.67 | 0 | 0.00 | 0.81 |
| **Pharmacy** | 241 | 83.97 | 15 | 5.23 | 25 | 8.71 | 4 | 1.39 | 2 | 0.70 | 1.08 |
| **Dentistry** | 295 | 81.27 | 25 | 6.89 | 32 | 8.82 | 9 | 2.48 | 2 | 0.55 | 1.05 |
| **Fine arts** | 274 | 85.09 | 17 | 5.28 | 20 | 6.21 | 5 | 1.55 | 6 | 1.86 | 1.10 |
| **J11. Do you think that vaccines in general are dangerous and should be avoided?** | | | | | | | | | | | |
| **All the rest** | 5700 | 61.20 | 1750 | 18.79 | 1146 | 12.31 | 414 | 4.45 | 303 | 3.25 | 1.00 |
| **Medicine** | 1518 | 76.55 | 235 | 11.85 | 150 | 7.56 | 45 | 2.27 | 35 | 1.77 | 1.25 |
| **Nursing** | 117 | 53.67 | 43 | 19.72 | 50 | 22.94 | 7 | 3.21 | 1 | 0.46 | 0.88 |
| **Pharmacy** | 207 | 72.13 | 45 | 15.68 | 22 | 7.67 | 7 | 2.44 | 6 | 2.09 | 1.18 |
| **Dentistry** | 245 | 67.49 | 51 | 14.05 | 43 | 11.85 | 14 | 3.86 | 10 | 2.75 | 1.10 |
| **Fine arts** | 210 | 65.22 | 45 | 13.98 | 37 | 11.49 | 17 | 5.28 | 13 | 4.04 | 1.07 |
| **J12. The government is secretly involved in the murder of innocent citizens and/or well-known public figures.** | | | | | | | | | | | |
| **All the rest** | 5295 | 56.86 | 1660 | 17.82 | 1409 | 15.13 | 533 | 5.72 | 416 | 4.47 | 1.00 |
| **Medicine** | 1307 | 65.91 | 285 | 14.37 | 250 | 12.61 | 78 | 3.93 | 63 | 3.18 | 1.16 |
| **Nursing** | 119 | 54.59 | 46 | 21.10 | 38 | 17.43 | 11 | 5.05 | 4 | 1.83 | 0.96 |
| **Pharmacy** | 175 | 60.98 | 47 | 16.38 | 48 | 16.72 | 9 | 3.14 | 8 | 2.79 | 1.07 |
| **Dentistry** | 213 | 58.68 | 55 | 15.15 | 54 | 14.88 | 27 | 7.44 | 14 | 3.86 | 1.03 |
| **Fine arts** | 167 | 51.86 | 59 | 18.32 | 54 | 16.77 | 20 | 6.21 | 22 | 6.83 | 0.91 |
| **J13. Global warming and climate change is a greatly exaggerated myth to serve various political and financial interests.** | | | | | | | | | | | |
| **All the rest** | 7266 | 78.02 | 902 | 9.69 | 789 | 8.47 | 215 | 2.31 | 141 | 1.51 | 1.00 |
| **Medicine** | 1593 | 80.33 | 182 | 9.18 | 122 | 6.15 | 51 | 2.57 | 35 | 1.77 | 1.03 |
| **Nursing** | 130 | 59.63 | 38 | 17.43 | 44 | 20.18 | 3 | 1.38 | 3 | 1.38 | 0.76 |
| **Pharmacy** | 233 | 81.18 | 25 | 8.71 | 24 | 8.36 | 5 | 1.74 | 0 | 0.00 | 1.04 |
| **Dentistry** | 278 | 76.58 | 38 | 10.47 | 35 | 9.64 | 10 | 2.75 | 2 | 0.55 | 0.98 |
| **Fine arts** | 273 | 84.78 | 24 | 7.45 | 18 | 5.59 | 4 | 1.24 | 3 | 0.93 | 1.09 |
| **J14. The power held by the heads of state is smaller than that of small unknown groups that really control the world of politics.** | | | | | | | | | | | |
| **All the rest** | 3618 | 38.85 | 2082 | 22.36 | 1990 | 21.37 | 945 | 10.15 | 678 | 7.28 | 1.00 |
| **Medicine** | 1011 | 50.98 | 407 | 20.52 | 336 | 16.94 | 141 | 7.11 | 88 | 4.44 | 1.31 |
| **Nursing** | 94 | 43.12 | 47 | 21.56 | 60 | 27.52 | 12 | 5.50 | 5 | 2.29 | 1.11 |
| **Pharmacy** | 125 | 43.55 | 58 | 20.21 | 63 | 21.95 | 27 | 9.41 | 14 | 4.88 | 1.12 |
| **Dentistry** | 151 | 41.60 | 87 | 23.97 | 77 | 21.21 | 25 | 6.89 | 23 | 6.34 | 1.07 |
| **Fine arts** | 110 | 34.16 | 71 | 22.05 | 82 | 25.47 | 32 | 9.94 | 27 | 8.39 | 0.88 |
| **J15. Secret organizations are communicating with aliens, but they hide it from the public.** | | | | | | | | | | | |
| **All the rest** | 7542 | 80.98 | 756 | 8.12 | 707 | 7.59 | 149 | 1.60 | 159 | 1.71 | 1.00 |
| **Medicine** | 1709 | 86.18 | 136 | 6.86 | 86 | 4.34 | 26 | 1.31 | 26 | 1.31 | 1.06 |
| **Nursing** | 134 | 61.47 | 28 | 12.84 | 49 | 22.48 | 7 | 3.21 | 0 | 0.00 | 0.76 |
| **Pharmacy** | 246 | 85.71 | 20 | 6.97 | 19 | 6.62 | 1 | 0.35 | 1 | 0.35 | 1.06 |
| **Dentistry** | 301 | 82.92 | 21 | 5.79 | 30 | 8.26 | 5 | 1.38 | 6 | 1.65 | 1.02 |
| **Fine arts** | 266 | 82.61 | 21 | 6.52 | 20 | 6.21 | 6 | 1.86 | 9 | 2.80 | 1.02 |
| **J16. Groups of scientists manipulate, invent or conceal evidence to deceive the public.** | | | | | | | | | | | |
| **All the rest** | 4129 | 44.34 | 2314 | 24.85 | 1741 | 18.69 | 651 | 6.99 | 478 | 5.13 | 1.00 |
| **Medicine** | 1222 | 61.62 | 372 | 18.76 | 270 | 13.62 | 74 | 3.73 | 45 | 2.27 | 1.39 |
| **Nursing** | 102 | 46.79 | 49 | 22.48 | 52 | 23.85 | 12 | 5.50 | 3 | 1.38 | 1.06 |
| **Pharmacy** | 160 | 55.75 | 76 | 26.48 | 34 | 11.85 | 15 | 5.23 | 2 | 0.70 | 1.26 |
| **Dentistry** | 193 | 53.17 | 80 | 22.04 | 59 | 16.25 | 15 | 4.13 | 16 | 4.41 | 1.20 |
| **Fine arts** | 138 | 42.86 | 84 | 26.09 | 63 | 19.57 | 22 | 6.83 | 15 | 4.66 | 0.97 |
| **J17. The government allows or commits acts of terrorism on its territory, disguising its involvement as if someone else is responsible.** | | | | | | | | | | | |
| **All the rest** | 4192 | 45.01 | 1964 | 21.09 | 1709 | 18.35 | 821 | 8.82 | 627 | 6.73 | 1.00 |
| **Medicine** | 1213 | 61.17 | 372 | 18.76 | 262 | 13.21 | 78 | 3.93 | 58 | 2.92 | 1.36 |
| **Nursing** | 108 | 49.54 | 48 | 22.02 | 45 | 20.64 | 15 | 6.88 | 2 | 0.92 | 1.10 |
| **Pharmacy** | 149 | 51.92 | 54 | 18.82 | 53 | 18.47 | 24 | 8.36 | 7 | 2.44 | 1.15 |
| **Dentistry** | 203 | 55.92 | 67 | 18.46 | 58 | 15.98 | 22 | 6.06 | 13 | 3.58 | 1.24 |
| **Fine arts** | 109 | 33.85 | 79 | 24.53 | 60 | 18.63 | 44 | 13.66 | 30 | 9.32 | 0.75 |
| **J18. Do you believe that secretly a chip will be included in the COVID-19 vaccine in order to mark people?** | | | | | | | | | | | |
| **All the rest** | 6901 | 74.10 | 928 | 9.96 | 984 | 10.57 | 269 | 2.89 | 231 | 2.48 | 1.00 |
| **Medicine** | 1662 | 83.81 | 143 | 7.21 | 135 | 6.81 | 21 | 1.06 | 22 | 1.11 | 1.13 |
| **Nursing** | 127 | 58.26 | 38 | 17.43 | 46 | 21.10 | 6 | 2.75 | 1 | 0.46 | 0.79 |
| **Pharmacy** | 228 | 79.44 | 29 | 10.10 | 23 | 8.01 | 6 | 2.09 | 1 | 0.35 | 1.07 |
| **Dentistry** | 270 | 74.38 | 40 | 11.02 | 31 | 8.54 | 12 | 3.31 | 10 | 2.75 | 1.00 |
| **Fine arts** | 255 | 79.19 | 27 | 8.39 | 23 | 7.14 | 12 | 3.73 | 5 | 1.55 | 1.07 |
| **J19. A small, secret group of people is responsible for taking all the important decisions, such as starting wars, in a planned way and the reasons are the group’s secret interests.** | | | | | | | | | | | |
| **All the rest** | 4656 | 49.99 | 1905 | 20.46 | 1575 | 16.91 | 651 | 6.99 | 526 | 5.65 | 1.00 |
| **Medicine** | 1204 | 60.72 | 358 | 18.05 | 272 | 13.72 | 89 | 4.49 | 60 | 3.03 | 1.21 |
| **Nursing** | 95 | 43.58 | 50 | 22.94 | 55 | 25.23 | 7 | 3.21 | 11 | 5.05 | 0.87 |
| **Pharmacy** | 156 | 54.36 | 66 | 23.00 | 42 | 14.63 | 16 | 5.57 | 7 | 2.44 | 1.09 |
| **Dentistry** | 204 | 56.20 | 62 | 17.08 | 62 | 17.08 | 19 | 5.23 | 16 | 4.41 | 1.12 |
| **Fine arts** | 165 | 51.24 | 62 | 19.25 | 55 | 17.08 | 25 | 7.76 | 15 | 4.66 | 1.02 |
| **J20. Technology and devices for mind control are used on people without their knowledge.** | | | | | | | | | | | |
| **All the rest** | 5570 | 59.81 | 1454 | 15.61 | 1331 | 14.29 | 553 | 5.94 | 405 | 4.35 | 1.00 |
| **Medicine** | 1396 | 70.40 | 265 | 13.36 | 214 | 10.79 | 68 | 3.43 | 40 | 2.02 | 1.18 |
| **Nursing** | 106 | 48.62 | 53 | 24.31 | 35 | 16.06 | 18 | 8.26 | 6 | 2.75 | 0.81 |
| **Pharmacy** | 185 | 64.46 | 52 | 18.12 | 35 | 12.20 | 6 | 2.09 | 9 | 3.14 | 1.08 |
| **Dentistry** | 230 | 63.36 | 51 | 14.05 | 50 | 13.77 | 19 | 5.23 | 13 | 3.58 | 1.06 |
| **Fine arts** | 206 | 63.98 | 43 | 13.35 | 50 | 15.53 | 17 | 5.28 | 6 | 1.86 | 1.07 |
| **J21. New and advanced technology that will make the existing industry obsolete is being suppressed in a malicious and violent way.** | | | | | | | | | | | |
| **All the rest** | 4915 | 52.78 | 2007 | 21.55 | 1614 | 17.33 | 494 | 5.30 | 283 | 3.04 | 1.00 |
| **Medicine** | 1273 | 64.20 | 319 | 16.09 | 274 | 13.82 | 70 | 3.53 | 47 | 2.37 | 1.22 |
| **Nursing** | 98 | 44.95 | 55 | 25.23 | 49 | 22.48 | 11 | 5.05 | 5 | 2.29 | 0.85 |
| **Pharmacy** | 174 | 60.63 | 54 | 18.82 | 47 | 16.38 | 11 | 3.83 | 1 | 0.35 | 1.15 |
| **Dentistry** | 207 | 57.02 | 68 | 18.73 | 61 | 16.80 | 14 | 3.86 | 13 | 3.58 | 1.08 |
| **Fine arts** | 175 | 54.35 | 69 | 21.43 | 48 | 14.91 | 23 | 7.14 | 7 | 2.17 | 1.03 |
| **J22. Some important events happen due to the activity of a small group who secretly manipulate world events.** | | | | | | | | | | | |
| **All the rest** | 4526 | 48.60 | 2024 | 21.73 | 1652 | 17.74 | 652 | 7.00 | 459 | 4.93 | 1.00 |
| **Medicine** | 1156 | 58.30 | 373 | 18.81 | 306 | 15.43 | 82 | 4.14 | 66 | 3.33 | 1.20 |
| **Nursing** | 94 | 43.12 | 53 | 24.31 | 49 | 22.48 | 11 | 5.05 | 11 | 5.05 | 0.89 |
| **Pharmacy** | 142 | 49.48 | 72 | 25.09 | 53 | 18.47 | 13 | 4.53 | 7 | 2.44 | 1.02 |
| **Dentistry** | 187 | 51.52 | 66 | 18.18 | 70 | 19.28 | 20 | 5.51 | 20 | 5.51 | 1.06 |
| **Fine arts** | 165 | 51.24 | 71 | 22.05 | 47 | 14.60 | 29 | 9.01 | 10 | 3.11 | 1.05 |
| **J23. Experiments involving new drugs or technologies are performed systematically on humans in a secret way and without their knowledge or consent.** | | | | | | | | | | | |
| **All the rest** | 5005 | 53.74 | 2005 | 21.53 | 1495 | 16.05 | 504 | 5.41 | 304 | 3.26 | 1.00 |
| **Medicine** | 1255 | 63.29 | 372 | 18.76 | 262 | 13.21 | 58 | 2.92 | 36 | 1.82 | 1.18 |
| **Nursing** | 95 | 43.58 | 56 | 25.69 | 51 | 23.39 | 8 | 3.67 | 8 | 3.67 | 0.81 |
| **Pharmacy** | 198 | 68.99 | 50 | 17.42 | 31 | 10.80 | 4 | 1.39 | 4 | 1.39 | 1.28 |
| **Dentistry** | 200 | 55.10 | 77 | 21.21 | 63 | 17.36 | 15 | 4.13 | 8 | 2.20 | 1.03 |
| **Fine arts** | 162 | 50.31 | 70 | 21.74 | 68 | 21.12 | 15 | 4.66 | 7 | 2.17 | 0.94 |
| **J24. Many important pieces of information are deliberately hidden from the public for reasons of interest.** | | | | | | | | | | | |
| **All the rest** | 1484 | 15.93 | 1793 | 19.25 | 2300 | 24.70 | 1762 | 18.92 | 1974 | 21.20 | 1.00 |
| **Medicine** | 512 | 25.82 | 426 | 21.48 | 513 | 25.87 | 296 | 14.93 | 236 | 11.90 | 1.62 |
| **Nursing** | 51 | 23.39 | 59 | 27.06 | 60 | 27.52 | 22 | 10.09 | 26 | 11.93 | 1.47 |
| **Pharmacy** | 68 | 23.69 | 67 | 23.34 | 57 | 19.86 | 46 | 16.03 | 49 | 17.07 | 1.49 |
| **Dentistry** | 56 | 15.43 | 84 | 23.14 | 103 | 28.37 | 63 | 17.36 | 57 | 15.70 | 0.97 |
| **Fine arts** | 37 | 11.49 | 73 | 22.67 | 69 | 21.43 | 67 | 20.81 | 76 | 23.60 | 0.72 |
| **J25. The spread of certain viruses and/or diseases is the result of deliberate, covert actions of an organization or government.** | | | | | | | | | | | |
| **All the rest** | 4836 | 51.93 | 1770 | 19.01 | 1623 | 17.43 | 573 | 6.15 | 511 | 5.49 | 1.00 |
| **Medicine** | 1277 | 64.40 | 328 | 16.54 | 243 | 12.25 | 77 | 3.88 | 58 | 2.92 | 1.24 |
| **Nursing** | 98 | 44.95 | 50 | 22.94 | 49 | 22.48 | 12 | 5.50 | 9 | 4.13 | 0.87 |
| **Pharmacy** | 176 | 61.32 | 52 | 18.12 | 43 | 14.98 | 11 | 3.83 | 5 | 1.74 | 1.18 |
| **Dentistry** | 188 | 51.79 | 77 | 21.21 | 70 | 19.28 | 17 | 4.68 | 11 | 3.03 | 1.00 |
| **Fine arts** | 167 | 51.86 | 70 | 21.74 | 60 | 18.63 | 16 | 4.97 | 9 | 2.80 | 1.00 |
| **J26. It is possible that the earth is flat rather than spherical.** | | | | | | | | | | | |
| **All the rest** | 8552 | 91.83 | 265 | 2.85 | 336 | 3.61 | 78 | 0.84 | 82 | 0.88 | 1.00 |
| **Medicine** | 1822 | 91.88 | 56 | 2.82 | 65 | 3.28 | 12 | 0.61 | 28 | 1.41 | 1.00 |
| **Nursing** | 153 | 70.18 | 29 | 13.30 | 29 | 13.30 | 5 | 2.29 | 2 | 0.92 | 0.76 |
| **Pharmacy** | 272 | 94.77 | 6 | 2.09 | 8 | 2.79 | 1 | 0.35 | 0 | 0.00 | 1.03 |
| **Dentistry** | 329 | 90.63 | 8 | 2.20 | 21 | 5.79 | 5 | 1.38 | 0 | 0.00 | 0.99 |
| **Fine arts** | 310 | 96.27 | 3 | 0.93 | 6 | 1.86 | 1 | 0.31 | 2 | 0.62 | 1.05 |
| **Average** |  |  |  |  |  |  |  |  |  |  |  |
| **All the rest** |  |  |  |  |  |  |  |  |  |  | 1.00 |
| **Medicine** |  |  |  |  |  |  |  |  |  |  | 1.25 |
| **Nursing** |  |  |  |  |  |  |  |  |  |  | 0.89 |
| **Pharmacy** |  |  |  |  |  |  |  |  |  |  | 1.15 |
| **Dentistry** |  |  |  |  |  |  |  |  |  |  | 1.09 |
| **Fine arts** |  |  |  |  |  |  |  |  |  |  | 0.99 |

**Webtable 17**: rates of accepting conspiracy theories by specific schools

* J5 excluded

|  | **I don’t**  **believe**  **it at all** | **A little bit** | **Maybe** | **Much** | **Very**  **much** | **A:B** | **A:C** | **B:C** |
| --- | --- | --- | --- | --- | --- | --- | --- | --- |
| **J1. Do you believe that the COVID-19 vaccine was ready even before the virus broke out and they conceal it from us for the benefit of pharmaceutical companies?** | | | | | | | | |
| **No dysphoria/depression** | 57.94 | 13.25 | 21.94 | 3.70 | 3.18 | 1.00 | 1.07 | 1.07 |
| **Dysphoria** | 58.08 | 12.58 | 20.55 | 4.65 | 4.14 |  |  |  |
| **Clinical depression** | 54.34 | 11.94 | 22.12 | 6.11 | 5.49 |  |  |  |
| **Total** | 57.03 | 12.81 | 21.77 | 4.47 | 3.92 |  |  |  |
| **J2. Do you believe that COVID-19 was created in a laboratory?** | | | | | | | | |
| **No dysphoria/depression** | 32.97 | 20.12 | 27.74 | 10.95 | 8.21 | 1.07 | 1.07 | 1.01 |
| **Dysphoria** | 30.93 | 19.33 | 27.10 | 11.55 | 11.09 |  |  |  |
| **Clinical depression** | 30.68 | 16.72 | 27.45 | 12.69 | 12.47 |  |  |  |
| **Total** | 32.06 | 19.12 | 27.57 | 11.49 | 9.76 |  |  |  |
| **J3. Do you think that COVID-19 was created to be used as a biochemical weapon for the extermination of the human population?** | | | | | | | | |
| **No dysphoria/depression** | 53.00 | 16.21 | 19.40 | 6.82 | 4.59 | 1.03 | 1.12 | 1.09 |
| **Dysphoria** | 51.43 | 15.44 | 18.87 | 8.28 | 5.98 |  |  |  |
| **Clinical depression** | 47.15 | 15.57 | 18.83 | 9.03 | 9.43 |  |  |  |
| **Total** | 51.24 | 15.92 | 19.17 | 7.62 | 6.05 |  |  |  |
| **J4. Do you believe that COVID-19 is related to the 5G technology antenna?** | | | | | | | | |
| **No dysphoria/depression** | 84.18 | 5.94 | 7.68 | 1.36 | 0.85 | 0.98 | 1.03 | 1.05 |
| **Dysphoria** | 85.74 | 5.88 | 5.42 | 1.99 | 0.97 |  |  |  |
| **Clinical depression** | 81.39 | 6.64 | 7.75 | 2.30 | 1.92 |  |  |  |
| **Total** | 83.70 | 6.11 | 7.34 | 1.70 | 1.15 |  |  |  |
| **J5. Do you believe that COVID-19 appeared accidentally from human contact with animals and it was something that generally happens and was generally expected?** | | | | | | | | |
| **No dysphoria/depression** | 24.99 | 20.12 | 27.99 | 17.16 | 9.73 | 1.24 | 1.14 | 0.92 |
| **Dysphoria** | 20.19 | 19.33 | 28.12 | 21.42 | 10.94 |  |  |  |
| **Clinical depression** | 21.93 | 18.05 | 28.04 | 19.11 | 12.87 |  |  |  |
| **Total** | 23.45 | 19.46 | 28.02 | 18.33 | 10.73 |  |  |  |
| **J6. Do you believe that COVID-19 has much lower mortality rate but there is misinformation and terror-inducing propaganda?** | | | | | | | | |
|  |  |  |  |  |  |  |  |  |
| **No dysphoria/depression** | 32.17 | 20.54 | 21.09 | 14.23 | 11.96 | 1.08 | 1.17 | 1.08 |
| **Dysphoria** | 29.65 | 21.01 | 19.73 | 14.78 | 14.83 |  |  |  |
| **Clinical depression** | 27.45 | 18.92 | 20.78 | 15.60 | 17.25 |  |  |  |
| **Total** | 30.55 | 20.20 | 20.80 | 14.67 | 13.78 |  |  |  |
| **J7. Do you think the recommended measures (e.g. wearing face masks. avoid gatherings. stay at home etc.) are an attempt to restrict human rights and lead to some kind of dictatorship rather than to keep the population safer from COVID-19?** | | | | | | | | |
| **No dysphoria/depression** | 53.93 | 17.96 | 14.71 | 7.46 | 5.94 | 1.06 | 1.15 | 1.08 |
| **Dysphoria** | 50.77 | 18.92 | 14.16 | 8.44 | 7.72 |  |  |  |
| **Clinical depression** | 47.08 | 17.40 | 14.70 | 9.93 | 10.89 |  |  |  |
| **Total** | 51.67 | 17.96 | 14.62 | 8.25 | 7.50 |  |  |  |
| **J8. Do you believe that COVID-19 outbreak is a deliberate creation of the world’s powerful leaders to create a global economic crisis?** | | | | | | | | |
| **No dysphoria/depression** | 54.00 | 16.36 | 18.07 | 6.50 | 5.08 | 1.04 | 1.11 | 1.07 |
| **Dysphoria** | 52.10 | 17.02 | 16.97 | 8.18 | 5.73 |  |  |  |
| **Clinical depression** | 48.51 | 14.39 | 19.54 | 7.94 | 9.62 |  |  |  |
| **Total** | 52.28 | 15.95 | 18.28 | 7.14 | 6.35 |  |  |  |
| **J9. Do you believe that COVID-19 is a sign of divine power to destroy our planet?** | | | | | | | | |
| **No dysphoria/depression** | 81.13 | 7.50 | 8.28 | 1.67 | 1.42 | 1.01 | 1.01 | 1.00 |
| **Dysphoria** | 80.52 | 8.28 | 7.87 | 2.20 | 1.12 |  |  |  |
| **Clinical depression** | 80.61 | 7.38 | 7.72 | 2.23 | 2.05 |  |  |  |
| **Total** | 80.90 | 7.59 | 8.07 | 1.90 | 1.54 |  |  |  |
| **J10. Do you believe that airplanes secretly spray people with various chemicals?** | | | | | | | | |
| **No dysphoria/depression** | 79.95 | 8.92 | 8.08 | 1.90 | 1.15 | 0.99 | 1.03 | 1.04 |
| **Dysphoria** | 80.47 | 8.59 | 7.06 | 2.15 | 1.74 |  |  |  |
| **Clinical depression** | 77.36 | 8.75 | 8.90 | 3.13 | 1.86 |  |  |  |
| **Total** | 79.36 | 8.83 | 8.13 | 2.26 | 1.43 |  |  |  |
| **J11. Do you think that vaccines in general are dangerous and should be avoided?** | | | | | | | | |
| **No dysphoria/depression** | 65.12 | 17.51 | 11.48 | 3.49 | 2.40 | 1.00 | 1.07 | 1.06 |
| **Dysphoria** | 64.83 | 17.28 | 10.94 | 3.78 | 3.17 |  |  |  |
| **Clinical depression** | 61.14 | 17.12 | 12.25 | 5.43 | 4.06 |  |  |  |
| **Total** | 64.05 | 17.37 | 11.60 | 4.04 | 2.95 |  |  |  |
| **J12. The government is secretly involved in the murder of innocent citizens and/or well-known public figures.** | | | | | | | | |
| **No dysphoria/depression** | 61.39 | 17.30 | 13.89 | 4.30 | 3.12 | 1.13 | 1.15 | 1.02 |
| **Dysphoria** | 54.40 | 18.61 | 16.21 | 6.34 | 4.45 |  |  |  |
| **Clinical depression** | 53.57 | 16.25 | 16.16 | 7.44 | 6.58 |  |  |  |
| **Total** | 58.27 | 17.24 | 14.84 | 5.43 | 4.22 |  |  |  |
| **J13. Global warming and climate change is a greatly exaggerated myth to serve various political and financial interests.** | | | | | | | | |
| **No dysphoria/depression** | 78.18 | 10.33 | 8.35 | 1.89 | 1.25 | 0.99 | 1.00 | 1.01 |
| **Dysphoria** | 78.89 | 8.90 | 7.77 | 2.71 | 1.74 |  |  |  |
| **Clinical depression** | 78.10 | 8.68 | 8.37 | 3.01 | 1.83 |  |  |  |
| **Total** | 78.27 | 9.68 | 8.27 | 2.31 | 1.47 |  |  |  |
| **J14. The power held by the heads of state is smaller than that of small unknown groups that really control the world of politics.** | | | | | | | | |
| **No dysphoria/depression** | 43.24 | 21.80 | 20.60 | 8.91 | 5.45 | 1.15 | 1.15 | 1.00 |
| **Dysphoria** | 37.73 | 22.90 | 21.63 | 11.20 | 6.54 |  |  |  |
| **Clinical depression** | 37.59 | 22.05 | 21.09 | 9.68 | 9.58 |  |  |  |
| **Total** | 40.92 | 22.04 | 20.89 | 9.47 | 6.69 |  |  |  |
| **J15. Secret organizations are communicating with aliens. but they hide it from the public.** | | | | | | | | |
| **No dysphoria/depression** | 82.79 | 7.57 | 7.17 | 1.20 | 1.26 | 1.03 | 1.04 | 1.01 |
| **Dysphoria** | 80.57 | 9.30 | 6.85 | 1.74 | 1.53 |  |  |  |
| **Clinical depression** | 79.81 | 7.66 | 7.85 | 2.23 | 2.45 |  |  |  |
| **Total** | 81.68 | 7.86 | 7.30 | 1.55 | 1.61 |  |  |  |
| **J16. Groups of scientists manipulate. invent or conceal evidence to deceive the public.** | | | | | | | | |
| **No dysphoria/depression** | 49.59 | 24.05 | 17.23 | 5.63 | 3.50 | 1.08 | 1.13 | 1.04 |
| **Dysphoria** | 46.01 | 24.64 | 18.10 | 7.26 | 3.99 |  |  |  |
| **Clinical depression** | 44.08 | 22.83 | 18.80 | 7.32 | 6.98 |  |  |  |
| **Total** | 47.61 | 23.83 | 17.77 | 6.32 | 4.48 |  |  |  |
| **J17. The government allows or commits acts of terrorism on its territory. disguising its involvement as if someone else is responsible.** | | | | | | | | |
| **No dysphoria/depression** | 51.37 | 20.86 | 16.92 | 6.58 | 4.27 | 1.17 | 1.21 | 1.03 |
| **Dysphoria** | 43.76 | 22.19 | 17.94 | 9.71 | 6.39 |  |  |  |
| **Clinical depression** | 42.34 | 19.42 | 18.61 | 10.33 | 9.31 |  |  |  |
| **Total** | 47.85 | 20.70 | 17.52 | 8.04 | 5.90 |  |  |  |
| **J18. Do you believe that secretly a chip will be included in the COVID-19 vaccine in order to mark people?** | | | | | | | | |
| **No dysphoria/depression** | 76.07 | 9.83 | 10.02 | 2.31 | 1.77 | 1.00 | 1.02 | 1.03 |
| **Dysphoria** | 76.18 | 9.82 | 9.41 | 2.45 | 2.15 |  |  |  |
| **Clinical depression** | 74.29 | 9.15 | 10.11 | 3.38 | 3.07 |  |  |  |
| **Total** | 75.63 | 9.65 | 9.95 | 2.61 | 2.16 |  |  |  |
| **J19. A small. secret group of people is responsible for taking all the important decisions. such as starting wars. in a planned way and the reasons are the group’s secret interests.** | | | | | | | | |
| **No dysphoria/depression** | 54.01 | 19.68 | 16.01 | 5.93 | 4.37 | 1.09 | 1.12 | 1.03 |
| **Dysphoria** | 49.74 | 21.11 | 16.87 | 7.62 | 4.65 |  |  |  |
| **Clinical depression** | 48.42 | 20.22 | 17.40 | 6.98 | 6.98 |  |  |  |
| **Total** | 51.90 | 20.05 | 16.51 | 6.46 | 5.09 |  |  |  |
| **J20. Technology and devices for mind control are used on people without their knowledge.** | | | | | | | | |
| **No dysphoria/depression** | 63.50 | 15.44 | 13.43 | 4.69 | 2.94 | 1.04 | 1.10 | 1.05 |
| **Dysphoria** | 60.79 | 15.24 | 13.45 | 6.44 | 4.09 |  |  |  |
| **Clinical depression** | 57.85 | 15.26 | 14.61 | 6.58 | 5.71 |  |  |  |
| **Total** | 61.61 | 15.36 | 13.74 | 5.45 | 3.84 |  |  |  |
| **J21. New and advanced technology that will make the existing industry obsolete is being suppressed in a malicious and violent way.** | | | | | | | | |
| **No dysphoria/depression** | 57.60 | 20.34 | 16.14 | 3.94 | 1.98 | 1.11 | 1.14 | 1.03 |
| **Dysphoria** | 51.69 | 22.24 | 16.77 | 6.39 | 2.91 |  |  |  |
| **Clinical depression** | 50.34 | 20.19 | 18.18 | 6.51 | 4.78 |  |  |  |
| **Total** | 54.80 | 20.60 | 16.76 | 4.99 | 2.85 |  |  |  |
| **J22. Some important events happen due to the activity of a small group who secretly manipulate world events.** | | | | | | | | |
| **No dysphoria/depression** | 52.29 | 21.27 | 16.89 | 5.80 | 3.75 | 1.08 | 1.12 | 1.03 |
| **Dysphoria** | 48.21 | 21.98 | 17.89 | 7.67 | 4.24 |  |  |  |
| **Clinical depression** | 46.74 | 20.94 | 18.39 | 7.23 | 6.70 |  |  |  |
| **Total** | 50.22 | 21.30 | 17.44 | 6.46 | 4.59 |  |  |  |
| **J23. Experiments involving new drugs or technologies are performed systematically on humans in a secret way and without their knowledge or consent.** | | | | | | | | |
| **No dysphoria/depression** | 58.06 | 20.98 | 15.06 | 3.85 | 2.05 | 1.10 | 1.14 | 1.03 |
| **Dysphoria** | 52.71 | 22.70 | 16.46 | 5.67 | 2.45 |  |  |  |
| **Clinical depression** | 50.93 | 20.25 | 17.00 | 6.58 | 5.24 |  |  |  |
| **Total** | 55.38 | 21.06 | 15.78 | 4.84 | 2.94 |  |  |  |
| **J24. Many important pieces of information are deliberately hidden from the public for reasons of interest.** | | | | | | | | |
| **No dysphoria/depression** | 19.94 | 20.96 | 25.73 | 17.19 | 16.18 | 1.29 | 1.44 | 1.11 |
| **Dysphoria** | 15.49 | 18.71 | 24.49 | 20.81 | 20.50 |  |  |  |
| **Clinical depression** | 13.90 | 18.77 | 23.05 | 18.39 | 25.90 |  |  |  |
| **Total** | 17.68 | 20.04 | 24.84 | 18.07 | 19.37 |  |  |  |
| **J25. The spread of certain viruses and/or diseases is the result of deliberate. covert actions of an organization or government.** | | | | | | | | |
| **No dysphoria/depression** | 56.01 | 19.20 | 16.36 | 4.91 | 3.52 | 1.06 | 1.12 | 1.05 |
| **Dysphoria** | 52.86 | 20.19 | 14.72 | 6.54 | 5.67 |  |  |  |
| **Clinical depression** | 50.12 | 17.03 | 18.77 | 6.79 | 7.29 |  |  |  |
| **Total** | 54.00 | 18.80 | 16.72 | 5.65 | 4.83 |  |  |  |
| **J26. It is possible that the earth is flat rather than spherical.** | | | | | | | | |
| **No dysphoria/depression** | 92.31 | 2.85 | 3.48 | 0.62 | 0.75 | 1.02 | 1.02 | 1.00 |
| **Dysphoria** | 90.75 | 3.58 | 3.78 | 1.12 | 0.77 |  |  |  |
| **Clinical depression** | 90.54 | 2.76 | 4.25 | 1.09 | 1.36 |  |  |  |
| **Total** | 91.61 | 2.94 | 3.72 | 0.82 | 0.91 |  |  |  |
| **Average*** |  |  |  |  |  | 1.06 | 1.11 | 1.04 |

**Webtable 18**: rates of accepting conspiracy theories by the presence of depression or dysphoria

* J5 excluded

|  | **I don’t believe it at all** | | **A little bit** | | **Maybe** | | **Much** | | **Very much** | | **Ratio:**  **History/no history** |
| --- | --- | --- | --- | --- | --- | --- | --- | --- | --- | --- | --- |
|  | **N** | **%** | **N** | **%** | **N** | **%** | **N** | **%** | **N** | **%** |  |
| **J1. Do you believe that the COVID-19 vaccine was ready even before the virus broke out and they conceal it from us for the benefit of pharmaceutical companies?** | | | | | | | | | | | |
| **No history** | 5184 | 56.16 | 1217 | 13.19 | 2055 | 22.26 | 425 | 4.60 | 349 | 3.78 | 1.06 |
| **Any history** | 1937 | 59.49 | 382 | 11.73 | 663 | 20.36 | 133 | 4.08 | 141 | 4.33 |  |
| **All Grps** | 7121 | 57.03 | 1599 | 12.81 | 2718 | 21.77 | 558 | 4.47 | 490 | 3.92 |  |
| **J2. Do you believe that COVID-19 was created in a laboratory?** | | | | | | | | | | | |
| **No history** | 2892 | 31.33 | 1807 | 19.58 | 2550 | 27.63 | 1087 | 11.78 | 894 | 9.69 | 1.09 |
| **Any history** | 1111 | 34.12 | 580 | 17.81 | 892 | 27.40 | 348 | 10.69 | 325 | 9.98 |  |
| **All Grps** | 4003 | 32.06 | 2387 | 19.12 | 3442 | 27.57 | 1435 | 11.49 | 1219 | 9.76 |  |
| **J3. Do you think that COVID-19 was created to be used as a biochemical weapon for the extermination of the human population?** | | | | | | | | | | | |
| **No history** | 4654 | 50.42 | 1519 | 16.46 | 1809 | 19.60 | 694 | 7.52 | 554 | 6.00 |  |
| **Any history** | 1744 | 53.56 | 469 | 14.40 | 584 | 17.94 | 257 | 7.89 | 202 | 6.20 |  |
| **All Grps** | 6398 | 51.24 | 1988 | 15.92 | 2393 | 19.17 | 951 | 7.62 | 756 | 6.05 |  |
| **J4. Do you believe that COVID-19 is related to the 5G technology antenna?** | | | | | | | | | | | |
| **No history** | 7656 | 82.95 | 592 | 6.41 | 718 | 7.78 | 157 | 1.70 | 107 | 1.16 | 1.03 |
| **Any history** | 2795 | 85.84 | 171 | 5.25 | 199 | 6.11 | 55 | 1.69 | 36 | 1.11 |  |
| **All Grps** | 10451 | 83.70 | 763 | 6.11 | 917 | 7.34 | 212 | 1.70 | 143 | 1.15 |  |
|  |  |  |  |  |  |  |  |  |  |  |  |
| **No history** | 2259 | 24.47 | 1847 | 20.01 | 2598 | 28.15 | 1636 | 17.72 | 890 | 9.64 | 0.84 |
| **Any history** | 669 | 20.55 | 583 | 17.91 | 901 | 27.67 | 653 | 20.06 | 450 | 13.82 |  |
| **All Grps** | 2928 | 23.45 | 2430 | 19.46 | 3499 | 28.02 | 2289 | 18.33 | 1340 | 10.73 |  |
| **J6. Do you believe that COVID-19 has much lower mortality rate but there is misinformation and terror-inducing propaganda?** | | | | | | | | | | | |
| **No history** | 2800 | 30.34 | 1852 | 20.07 | 1959 | 21.22 | 1342 | 14.54 | 1277 | 13.84 | 1.03 |
| **Any history** | 1015 | 31.17 | 670 | 20.58 | 638 | 19.59 | 490 | 15.05 | 443 | 13.61 |  |
| **All Grps** | 3815 | 30.55 | 2522 | 20.20 | 2597 | 20.80 | 1832 | 14.67 | 1720 | 13.78 |  |
| **J7. Do you think the recommended measures (e.g. wearing face masks, avoid gatherings, stay at home etc.) are an attempt to restrict human rights and lead to some kind of dictatorship rather than to keep the population safer from COVID-19?** | | | | | | | | | | | |
| **No history** | 4772 | 51.70 | 1646 | 17.83 | 1388 | 15.04 | 756 | 8.19 | 668 | 7.24 | 1.00 |
| **Any history** | 1679 | 51.57 | 597 | 18.34 | 438 | 13.45 | 274 | 8.42 | 268 | 8.23 |  |
| **All Grps** | 6451 | 51.67 | 2243 | 17.96 | 1826 | 14.62 | 1030 | 8.25 | 936 | 7.50 |  |
| **J8. Do you believe that COVID-19 outbreak is a deliberate creation of the world’s powerful leaders to create a global economic crisis?** | | | | | | | | | | | |
| **No history** | 4751 | 51.47 | 1494 | 16.19 | 1746 | 18.92 | 671 | 7.27 | 568 | 6.15 | 1.06 |
| **Any history** | 1777 | 54.58 | 498 | 15.29 | 536 | 16.46 | 220 | 6.76 | 225 | 6.91 |  |
| **All Grps** | 6528 | 52.28 | 1992 | 15.95 | 2282 | 18.28 | 891 | 7.14 | 793 | 6.35 |  |
| **J9. Do you believe that COVID-19 is a sign of divine power to destroy our planet?** | | | | | | | | | | | |
| **No history** | 7409 | 80.27 | 702 | 7.61 | 794 | 8.60 | 182 | 1.97 | 143 | 1.55 | 1.03 |
| **Any history** | 2692 | 82.68 | 246 | 7.56 | 214 | 6.57 | 55 | 1.69 | 49 | 1.50 |  |
| **All Grps** | 10101 | 80.90 | 948 | 7.59 | 1008 | 8.07 | 237 | 1.90 | 192 | 1.54 |  |
| **J10. Do you believe that airplanes secretly spray people with various chemicals?** | | | | | | | | | | | |
| **No history** | 7282 | 78.89 | 831 | 9.00 | 782 | 8.47 | 213 | 2.31 | 122 | 1.32 | 1.02 |
| **Any history** | 2627 | 80.68 | 271 | 8.32 | 233 | 7.16 | 69 | 2.12 | 56 | 1.72 |  |
| **All Grps** | 9909 | 79.36 | 1102 | 8.83 | 1015 | 8.13 | 282 | 2.26 | 178 | 1.43 |  |
| **J11. Do you think that vaccines in general are dangerous and should be avoided?** | | | | | | | | | | | |
| **No history** | 5837 | 63.24 | 1615 | 17.50 | 1133 | 12.28 | 380 | 4.12 | 265 | 2.87 | 1.05 |
| **Any history** | 2160 | 66.34 | 554 | 17.01 | 315 | 9.67 | 124 | 3.81 | 103 | 3.16 |  |
| **All Grps** | 7997 | 64.05 | 2169 | 17.37 | 1448 | 11.60 | 504 | 4.04 | 368 | 2.95 |  |
| **J12. The government is secretly involved in the murder of innocent citizens and/or well-known public figures.** | | | | | | | | | | | |
| **No history** | 5438 | 58.92 | 1603 | 17.37 | 1357 | 14.70 | 478 | 5.18 | 354 | 3.84 | 0.96 |
| **Any history** | 1838 | 56.45 | 549 | 16.86 | 496 | 15.23 | 200 | 6.14 | 173 | 5.31 |  |
| **All Grps** | 7276 | 58.27 | 2152 | 17.24 | 1853 | 14.84 | 678 | 5.43 | 527 | 4.22 |  |
| **J13. Global warming and climate change is a greatly exaggerated myth to serve various political and financial interests.** | | | | | | | | | | | |
| **No history** | 7119 | 77.13 | 956 | 10.36 | 796 | 8.62 | 227 | 2.46 | 132 | 1.43 | 1.06 |
| **Any history** | 2654 | 81.51 | 253 | 7.77 | 236 | 7.25 | 61 | 1.87 | 52 | 1.60 |  |
| **All Grps** | 9773 | 78.27 | 1209 | 9.68 | 1032 | 8.27 | 288 | 2.31 | 184 | 1.47 |  |
| **J14. The power held by the heads of state is smaller than that of small unknown groups that really control the world of politics.** | | | | | | | | | | | |
| **No history** | 3822 | 41.41 | 2016 | 21.84 | 1965 | 21.29 | 856 | 9.27 | 571 | 6.19 | 0.95 |
| **Any history** | 1287 | 39.53 | 736 | 22.60 | 643 | 19.75 | 326 | 10.01 | 264 | 8.11 |  |
| **All Grps** | 5109 | 40.92 | 2752 | 22.04 | 2608 | 20.89 | 1182 | 9.47 | 835 | 6.69 |  |
| **J15. Secret organizations are communicating with aliens, but they hide it from the public.** | | | | | | | | | | | |
| **No history** | 7508 | 81.34 | 747 | 8.09 | 686 | 7.43 | 146 | 1.58 | 143 | 1.55 | 1.02 |
| **Any history** | 2690 | 82.62 | 235 | 7.22 | 225 | 6.91 | 48 | 1.47 | 58 | 1.78 |  |
| **All Grps** | 10198 | 81.68 | 982 | 7.86 | 911 | 7.30 | 194 | 1.55 | 201 | 1.61 |  |
| **J16. Groups of scientists manipulate, invent or conceal evidence to deceive the public.** | | | | | | | | | | | |
| **No history** | 4380 | 47.45 | 2235 | 24.21 | 1644 | 17.81 | 575 | 6.23 | 396 | 4.29 | 1.01 |
| **Any history** | 1564 | 48.03 | 740 | 22.73 | 575 | 17.66 | 214 | 6.57 | 163 | 5.01 |  |
| **All Grps** | 5944 | 47.61 | 2975 | 23.83 | 2219 | 17.77 | 789 | 6.32 | 559 | 4.48 |  |
| **J17. The government allows or commits acts of terrorism on its territory, disguising its involvement as if someone else is responsible.** | | | | | | | | | | | |
| **No history** | 4498 | 48.73 | 1938 | 21.00 | 1623 | 17.58 | 689 | 7.46 | 482 | 5.22 | 0.93 |
| **Any history** | 1476 | 45.33 | 646 | 19.84 | 564 | 17.32 | 315 | 9.67 | 255 | 7.83 |  |
| **All Grps** | 5974 | 47.85 | 2584 | 20.70 | 2187 | 17.52 | 1004 | 8.04 | 737 | 5.90 |  |
| **J18. Do you believe that secretly a chip will be included in the COVID-19 vaccine in order to mark people?** | | | | | | | | | | | |
| **No history** | 6898 | 74.73 | 934 | 10.12 | 968 | 10.49 | 237 | 2.57 | 193 | 2.09 | 1.05 |
| **Any history** | 2545 | 78.16 | 271 | 8.32 | 274 | 8.42 | 89 | 2.73 | 77 | 2.36 |  |
| **All Grps** | 9443 | 75.63 | 1205 | 9.65 | 1242 | 9.95 | 326 | 2.61 | 270 | 2.16 |  |
| **J19. A small, secret group of people is responsible for taking all the important decisions, such as starting wars, in a planned way and the reasons are the group’s secret interests.** | | | | | | | | | | | |
| **No history** | 4785 | 51.84 | 1862 | 20.17 | 1532 | 16.60 | 597 | 6.47 | 454 | 4.92 | 1.00 |
| **Any history** | 1695 | 52.06 | 641 | 19.69 | 529 | 16.25 | 210 | 6.45 | 181 | 5.56 |  |
| **All Grps** | 6480 | 51.90 | 2503 | 20.05 | 2061 | 16.51 | 807 | 6.46 | 635 | 5.09 |  |
| **J20. Technology and devices for mind control are used on people without their knowledge.** | | | | | | | | | | | |
| **No history** | 5681 | 61.55 | 1427 | 15.46 | 1286 | 13.93 | 509 | 5.51 | 327 | 3.54 | 1.00 |
| **Any history** | 2012 | 61.79 | 491 | 15.08 | 429 | 13.18 | 172 | 5.28 | 152 | 4.67 |  |
| **All Grps** | 7693 | 61.61 | 1918 | 15.36 | 1715 | 13.74 | 681 | 5.45 | 479 | 3.84 |  |
| **J21. New and advanced technology that will make the existing industry obsolete is being suppressed in a malicious and violent way.** | | | | | | | | | | | |
| **No history** | 5032 | 54.52 | 1945 | 21.07 | 1554 | 16.84 | 461 | 4.99 | 238 | 2.58 | 1.02 |
| **Any history** | 1810 | 55.59 | 627 | 19.26 | 539 | 16.55 | 162 | 4.98 | 118 | 3.62 |  |
| **All Grps** | 6842 | 54.80 | 2572 | 20.60 | 2093 | 16.76 | 623 | 4.99 | 356 | 2.85 |  |
| **J22. Some important events happen due to the activity of a small group who secretly manipulate world events.** | | | | | | | | | | | |
| **No history** | 4629 | 50.15 | 1987 | 21.53 | 1621 | 17.56 | 585 | 6.34 | 408 | 4.42 | 1.00 |
| **Any history** | 1641 | 50.40 | 672 | 20.64 | 556 | 17.08 | 222 | 6.82 | 165 | 5.07 |  |
| **All Grps** | 6270 | 50.22 | 2659 | 21.30 | 2177 | 17.44 | 807 | 6.46 | 573 | 4.59 |  |
| **J23. Experiments involving new drugs or technologies are performed systematically on humans in a secret way and without their knowledge or consent.** | | | | | | | | | | | |
| **No history** | 5096 | 55.21 | 1977 | 21.42 | 1453 | 15.74 | 455 | 4.93 | 249 | 2.70 | 1.01 |
| **Any history** | 1819 | 55.87 | 653 | 20.06 | 517 | 15.88 | 149 | 4.58 | 118 | 3.62 |  |
| **All Grps** | 6915 | 55.38 | 2630 | 21.06 | 1970 | 15.78 | 604 | 4.84 | 367 | 2.94 |  |
| **J24. Many important pieces of information are deliberately hidden from the public for reasons of interest.** | | | | | | | | | | | |
| **No history** | 1696 | 18.37 | 1874 | 20.30 | 2314 | 25.07 | 1649 | 17.87 | 1697 | 18.39 | 0.86 |
| **Any history** | 512 | 15.72 | 628 | 19.29 | 788 | 24.20 | 607 | 18.64 | 721 | 22.14 |  |
| **All Grps** | 2208 | 17.68 | 2502 | 20.04 | 3102 | 24.84 | 2256 | 18.07 | 2418 | 19.37 |  |
| **J25. The spread of certain viruses and/or diseases is the result of deliberate, covert actions of an organization or government.** | | | | | | | | | | | |
| **No history** | 4918 | 53.28 | 1805 | 19.56 | 1575 | 17.06 | 508 | 5.50 | 424 | 4.59 | 1.05 |
| **Any history** | 1824 | 56.02 | 542 | 16.65 | 513 | 15.76 | 198 | 6.08 | 179 | 5.50 |  |
| **All Grps** | 6742 | 54.00 | 2347 | 18.80 | 2088 | 16.72 | 706 | 5.65 | 603 | 4.83 |  |
| **J26. It is possible that the earth is flat rather than spherical.** | | | | | | | | | | | |
| **No history** | 8453 | 91.58 | 286 | 3.10 | 335 | 3.63 | 70 | 0.76 | 86 | 0.93 | 1.00 |
| **Any history** | 2985 | 91.68 | 81 | 2.49 | 130 | 3.99 | 32 | 0.98 | 28 | 0.86 |  |
| **All Grps** | 11438 | 91.61 | 367 | 2.94 | 465 | 3.72 | 102 | 0.82 | 114 | 0.91 |  |
| **Average *** |  |  |  |  |  |  |  |  |  |  | 1.01 |

**Webtable 19**: rates of accepting conspiracy theories by the presence of history of mental disorders

* J5 excluded

***3.6 Determinants of worsening of students’ mental health during the pandemic***

Four MSLRA were performed. The dependent variables were change in anxiety, change in depressive feelings, change in suicidal thoughts and clinical depression separately while in all analyses the same set of independent predictors was used and it included sex (A2) split into dummy variables, age (A3), type of studies (split into dummy variables), people living with (A6), health status (B1-2), vulnerable relative (B4), history of specific mental disorders (B5 split in dummy variables), thoughts pertaining to COVID-19 fears (C1-4), the degree of lockdown (D2), satisfaction by information (D4), family issues (E1-7), conspiracy theories (J1-26) and spirituality/religiosity (P1).

| **Dependent Variable: Change in anxiety**  **R²= 0.238; variance expl 23.8%; F(35,12124)=108.41 p<0.0000 Std.Error of estimate: 0.749** | | | | |
| --- | --- | --- | --- | --- |
|  | **b** | **Std.Err.** | **t(12124)** | **p-value** |
| Intercept | -0.71 | 0.04 | -17.24 | 0.0000 |
| Male sex | 0.09 | 0.02 | 5.48 | 0.0000 |
| Type A studies | 0.04 | 0.02 | 2.49 | 0.0129 |
| Number of people in the house | 0.02 | 0.01 | 4.02 | 0.0001 |
| Condition of general health | 0.12 | 0.01 | 18.27 | 0.0000 |
| Vulnerable person in the family | -0.05 | 0.01 | -3.25 | 0.0012 |
| History of anxiety disorder | -0.1 | 0.02 | -4.36 | 0.0000 |
| History of depression | -0.09 | 0.02 | -4.14 | 0.0000 |
| History of Bipolar disorder | -0.14 | 0.07 | -1.99 | 0.0462 |
| C1. Are you afraid that you will contract the coronavirus? | -0.05 | 0.01 | -6.47 | 0.0000 |
| C3. Does the possibility that a member of your family could contract the coronavirus and die because of it, makes you frightened? | -0.05 | 0.01 | -6.85 | 0.0000 |
| C4. Are you afraid that in case you contract the coronavirus, some people will step away from your life and behave to you in a different way later? | -0.02 | 0.01 | -3.28 | 0.0010 |
| D2. Are you currently locked up in the house? | -0.06 | 0.01 | -7.99 | 0.0000 |
| E2. Do you want to receive emotional support from other members of your family during this period? | -0.12 | 0.01 | -14.77 | 0.0000 |
| E3. Are there any conflicts with the rest of your family members during this period? | -0.1 | 0.01 | -12.54 | 0.0000 |
| E4. Has the overall quality of relationships with the other members of your family changed compared to before the COVID – 19? | 0.13 | 0.01 | 12.65 | 0.0000 |
| E5. Do you manage to maintain a basic daily routine (waking up in the morning, regular meals and sleeping hours, activities) both yourself (if you live alone) or as a family? | 0.12 | 0.01 | 15.05 | 0.0000 |
| E7. How are your finances as a result of the outbreak? | 0.1 | 0.01 | 12.42 | 0.0000 |
| J5. Do you believe that COVID-19 appeared accidentally from human contact with animals and it was something that generally happens and was generally expected? | -0.03 | 0.01 | -5.17 | 0.0000 |
| J15. Secret organizations are communicating with aliens, but they hide it from the public. | 0.04 | 0.01 | 4.15 | 0.0000 |
| J24. Many important pieces of information are deliberately hidden from the public for reasons of interest | -0.04 | 0.01 | -6.58 | 0.0000 |
| J3. Do you think that COVID-19 was created to be used as a biochemical weapon for the extermination of the human population? | 0.02 | 0.01 | 2.45 | 0.0143 |
| J6. Do you believe that COVID-19 has much lower mortality rate but there is misinformation and terror-inducing propaganda? | -0.02 | 0.01 | -2.53 | 0.0113 |
| J7. Do you think the recommended measures (e.g. wearing face masks, avoid gatherings, stay at home etc.) are an attempt to restrict human rights and lead to some kind of dictatorship rather than to keep the population safer from COVID-19? | -0.04 | 0.01 | -5.63 | 0.0000 |
| J8. Do you believe that COVID-19 outbreak is a deliberate creation of the world’s powerful leaders to create a global economic crisis? | -0.03 | 0.01 | -3.87 | 0.0001 |
| J9. Do you believe that COVID-19 is a sign of divine power to destroy our planet? | 0.05 | 0.01 | 5.49 | 0.0000 |
| J14. The power held by the heads of state is smaller than that of small unknown groups that really control the world of politics. | 0.02 | 0.01 | 2.88 | 0.0040 |
| J17. The government allows or commits acts of terrorism on its territory, disguising its involvement as if someone else is responsible. | -0.03 | 0.01 | -4.03 | 0.0001 |
| J18. Do you believe that secretly a chip will be included in the COVID-19 vaccine in order to mark people? | 0.02 | 0.01 | 2.37 | 0.0178 |
| J23. Experiments involving new drugs or technologies are performed systematically on humans in a secret way and without their knowledge or consent. | 0.03 | 0.01 | 3.38 | 0.0007 |
| J26. It is possible that the earth is flat rather than spherical. | -0.03 | 0.01 | -2.28 | 0.0228 |
| P1. Over the last 2-3 weeks, have your religious/spiritual inquiries been increased? | 0.02 | 0.01 | 2.03 | 0.0426 |
|  |  |  |  |  |
| **Dependent Variable: Change in depressive emotions**  **R²= 0.195; explained var 19.5%. F(35,12124)=83.896 p<0.0000 Std.Error of estimate: 0.763** | | | | |
|  | **b** | **Std.Err.** | **t(12124)** | **p-value** |
| Intercept | -0.9 | 0.07 | -12.11 | 0.0000 |
| Male sex | 0.04 | 0.02 | 2.51 | 0.0120 |
| Age | 0.01 | 0 | 2.7 | 0.0069 |
| Type A studies | 0.05 | 0.02 | 3.02 | 0.0025 |
| Number of people in the house | 0.03 | 0.01 | 4.52 | 0.0000 |
| B1. General health over the last month | 0.1 | 0.01 | 15.47 | 0.0000 |
| Vulnerable person in the family | -0.04 | 0.01 | -2.84 | 0.0045 |
| History of depression | -0.12 | 0.02 | -5.32 | 0.0000 |
| C1. Are you afraid that you will contract the coronavirus? | -0.03 | 0.01 | -3.18 | 0.0015 |
| C2. Do you believe that the precautions work effectively or that if you are about to contract the disease, you will contract it anyway? | 0.05 | 0.02 | 3.4 | 0.0007 |
| C3. Does the possibility that a member of your family could contract the coronavirus and die because of it, makes you frightened? | -0.02 | 0.01 | -2.72 | 0.0065 |
| C4. Are you afraid that in case you contract the coronavirus, some people will step away from your life and behave to you in a different way later? | -0.03 | 0.01 | -4.11 | 0.0000 |
| D2. Are you currently locked up in the house? | -0.08 | 0.01 | -9.94 | 0.0000 |
| E1. Do you feel the need to communicate with other members of your family during this period? | 0.03 | 0.01 | 2.71 | 0.0068 |
| E2. Do you want to receive emotional support from other members of your family during this period? | -0.12 | 0.01 | -12.07 | 0.0000 |
| E3. Are there any conflicts with the rest of your family members during this period? | -0.08 | 0.01 | -9.07 | 0.0000 |
| E4. Has the overall quality of relationships with the other members of your family changed compared to before the COVID – 19? | 0.14 | 0.01 | 13.24 | 0.0000 |
| E5. Do you manage to maintain a basic daily routine (waking up in the morning, regular meals and sleeping hours, activities) both yourself (if you live alone) or as a family? | 0.12 | 0.01 | 14.18 | 0.0000 |
| E7. How are your finances as a result of the outbreak? | 0.06 | 0.01 | 7.52 | 0.0000 |
| J1. Do you believe that the COVID-19 vaccine was ready even before the virus broke out and they conceal it from us for the benefit of pharmaceutical companies? | 0.02 | 0.01 | 2.63 | 0.0084 |
| J5. Do you believe that COVID-19 appeared accidentally from human contact with animals and it was something that generally happens and was generally expected? | -0.02 | 0.01 | -4.02 | 0.0001 |
| J6. Do you believe that COVID-19 has much lower mortality rate but there is misinformation and terror-inducing propaganda? | -0.02 | 0.01 | -3.65 | 0.0003 |
| J7. Do you think the recommended measures (e.g. wearing face masks, avoid gatherings, stay at home etc.) are an attempt to restrict human rights and lead to some kind of dictatorship rather than to keep the population safer from COVID-19? | -0.04 | 0.01 | -6.03 | 0.0000 |
| J9. Do you believe that COVID-19 is a sign of divine power to destroy our planet? | 0.04 | 0.01 | 3.98 | 0.0001 |
| J13. Global warming and climate change is a greatly exaggerated myth to serve various political and financial interests. | 0.02 | 0.01 | 2.38 | 0.0174 |
| J15. Secret organizations are communicating with aliens, but they hide it from the public. | 0.04 | 0.01 | 4.33 | 0.0000 |
| J17. The government allows or commits acts of terrorism on its territory, disguising its involvement as if someone else is responsible. | -0.03 | 0.01 | -4.63 | 0.0000 |
| J18. Do you believe that secretly a chip will be included in the COVID-19 vaccine in order to mark people? | 0.06 | 0.01 | 5.66 | 0.0000 |
| J23. Experiments involving new drugs or technologies are performed systematically on humans in a secret way and without their knowledge or consent. | 0.02 | 0.01 | 2.64 | 0.0084 |
| J24. Many important pieces of information are deliberately hidden from the public for reasons of interest. | -0.03 | 0.01 | -4.65 | 0.0000 |
| J26. It is possible that the earth is flat rather than spherical. | -0.03 | 0.01 | -2.03 | 0.0423 |
|  |  |  |  |  |
| **Dependent Variable: Change in suicidal thoughts**  **R²= 0.062; explained var: 6.2% F(29,12130)=27.920 p<0.0000 Std.Error of estimate: 0.672** | | | | |
|  | **b** | **Std.Err.** | **t(12130)** | **p-value** |
| Intercept | 0.16 | 0.03 | 5.34 | 0.0000 |
| non-binary gender | 0.12 | 0.06 | 2.03 | 0.0427 |
| Type B studies | 0.03 | 0.01 | 2.41 | 0.0159 |
| B1. General health over the last month | -0.05 | 0.01 | -7.57 | 0.0000 |
| History of depression | 0.14 | 0.02 | 7.01 | 0.0000 |
| History of Bipolar disorder | 0.25 | 0.06 | 4.16 | 0.0000 |
| History of Psychosis | 0.12 | 0.06 | 1.97 | 0.0492 |
| C1. Are you afraid that you will contract the coronavirus? | 0.02 | 0.01 | 3.07 | 0.0021 |
| C4. Are you afraid that in case you contract the coronavirus, some people will step away from your life and behave to you in a different way later? | 0.02 | 0.01 | 2.88 | 0.0040 |
| D2. Are you currently locked up in the house? | 0.03 | 0.01 | 4.48 | 0.0000 |
| E2. Do you want to receive emotional support from other members of your family during this period? | 0.05 | 0.01 | 7.56 | 0.0000 |
| E3. Are there any conflicts with the rest of your family members during this period? | 0.05 | 0.01 | 6.57 | 0.0000 |
| E4. Has the overall quality of relationships with the other members of your family changed compared to before the COVID – 19? | -0.05 | 0.01 | -5.42 | 0.0000 |
| E5. Do you manage to maintain a basic daily routine (waking up in the morning, regular meals and sleeping hours, activities) both yourself (if you live alone) or as a family? | -0.05 | 0.01 | -6.81 | 0.0000 |
| E7. How are your finances as a result of the outbreak? | -0.02 | 0.01 | -2.99 | 0.0028 |
| J7. Do you think the recommended measures (e.g. wearing face masks, avoid gatherings, stay at home etc.) are an attempt to restrict human rights and lead to some kind of dictatorship rather than to keep the population safer from COVID-19? | 0.02 | 0.01 | 2.81 | 0.0049 |
| J11. Do you think that vaccines in general are dangerous and should be avoided? | -0.03 | 0.01 | -3.67 | 0.0002 |
| J12. The government is secretly involved in the murder of innocent citizens and/or well-known public figures. | 0.02 | 0.01 | 2.24 | 0.0250 |
| J14. The power held by the heads of state is smaller than that of small unknown groups that really control the world of politics. | 0.02 | 0.01 | 3.25 | 0.0012 |
| J15. Secret organizations are communicating with aliens, but they hide it from the public. | 0.02 | 0.01 | 2.06 | 0.0395 |
| J17. The government allows or commits acts of terrorism on its territory, disguising its involvement as if someone else is responsible. | 0.02 | 0.01 | 3.39 | 0.0007 |
| J18. Do you believe that secretly a chip will be included in the COVID-19 vaccine in order to mark people? | -0.03 | 0.01 | -3.38 | 0.0007 |
| J20. Technology and devices for mind control are used on people without their knowledge | -0.02 | 0.01 | -2.22 | 0.0267 |
| J26. It is possible that the earth is flat rather than spherical | -0.04 | 0.01 | -3.38 | 0.0007 |
|  |  |  |  |  |
| **Dependent Variable: Clinical depression**  **R²= 0.184; Explained var 18.4%; F(26,12133)=105.33 p<0.0000 Std.Error of estimate: 0.393** | | | | |
|  | **b** | **Std.Err.** | **t(12133)** | **p-value** |
| Intercept | 0.33 | 0.02 | 16.30 | 0.0000 |
| Males | -0.08 | 0.01 | -9.06 | 0.0000 |
| non-binary gender | 0.07 | 0.03 | 2.05 | 0.0408 |
| Type A studies | -0.02 | 0.01 | -2.87 | 0.0041 |
| Number of people in the house | -0.01 | 0.00 | -3.06 | 0.0022 |
| B1. General health over the last month | -0.04 | 0.00 | -12.66 | 0.0000 |
| History of anxiety disorder | 0.07 | 0.01 | 5.43 | 0.0000 |
| History of depression | 0.21 | 0.01 | 18.08 | 0.0000 |
| History of Bipolar disorder | 0.26 | 0.04 | 7.41 | 0.0000 |
| History of psychosis | 0.23 | 0.04 | 6.47 | 0.0000 |
| C2. Do you believe that the precautions work effectively or that if you are about to contract the disease, you will contract it anyway? | -0.03 | 0.01 | -3.13 | 0.0018 |
| C3. Does the possibility that a member of your family could contract the coronavirus and die because of it, makes you frightened? | 0.01 | 0.00 | 3.48 | 0.0005 |
| C4. Are you afraid that in case you contract the coronavirus, some people will step away from your life and behave to you in a different way later? | 0.02 | 0.00 | 6.82 | 0.0000 |
| D2. Are you currently locked up in the house? | 0.01 | 0.00 | 2.37 | 0.0180 |
| E1. Do you feel the need to communicate with other members of your family during this period? | -0.02 | 0.01 | -3.50 | 0.0005 |
| E2. Do you want to receive emotional support from other members of your family during this period? | 0.05 | 0.00 | 9.74 | 0.0000 |
| E3. Are there any conflicts with the rest of your family members during this period? | 0.03 | 0.00 | 7.87 | 0.0000 |
| E4. Has the overall quality of relationships with the other members of your family changed compared to before the COVID – 19? | -0.03 | 0.01 | -5.54 | 0.0000 |
| E5. Do you manage to maintain a basic daily routine (waking up in the morning, regular meals and sleeping hours, activities) both yourself (if you live alone) or as a family? | -0.08 | 0.00 | -18.41 | 0.0000 |
| E6. If you have children, how difficult is it to manage their daily life and behavior? | 0.03 | 0.02 | 2.07 | 0.0385 |
| E7. How are your finances as a result of the outbreak? | -0.02 | 0.00 | -6.03 | 0.0000 |
| J3. Do you think that COVID-19 was created to be used as a biochemical weapon for the extermination of the human population? | 0.01 | 0.00 | 3.59 | 0.0003 |
| J5. Do you believe that COVID-19 appeared accidentally from human contact with animals and it was something that generally happens and was generally expected? | 0.02 | 0.00 | 6.42 | 0.0000 |
| J17. The government allows or commits acts of terrorism on its territory, disguising its involvement as if someone else is responsible. | 0.01 | 0.00 | 3.72 | 0.0002 |
| J23. Experiments involving new drugs or technologies are performed systematically on humans in a secret way and without their knowledge or consent. | 0.01 | 0.00 | 3.00 | 0.0027 |
| J24. Many important pieces of information are deliberately hidden from the public for reasons of interest. | 0.01 | 0.00 | 2.23 | 0.0257 |
| P1. Over the last 2-3 weeks, have your religious/spiritual inquiries been increased? | 0.02 | 0.00 | 3.23 | 0.0012 |
|  |  |  |  |  |

**Webtable 20:** Multiple linear stepwise regression analysis with changes in anxiety, depression or suicidality and the presence of clinical depression as dependent variables separately. The independent variables were sex, age, type of studies, history of specific mental disorders and believing in conspiracy theories

**References**

1. von Elm E, Altman DG, Egger M, et al. The Strengthening the Reporting of Observational Studies in Epidemiology (STROBE) Statement: guidelines for reporting observational studies. *Int J Surg.* 2014;12(12):1495-1499.

2. Fountoulakis KN, Papadopoulou M, Kleanthous S, et al. Reliability and psychometric properties of the Greek translation of the State-Trait Anxiety Inventory form Y: preliminary data. *Ann Gen Psychiatry.* 2006;5:2.

3. Fountoulakis K, Iacovides A, Kleanthous S, et al. Reliability, validity and psychometric properties of the Greek translation of the Center for Epidemiological Studies-Depression (CES-D) Scale. *BMC Psychiatry.* 2001;1:3.

4. Fountoulakis KN, Pantoula E, Siamouli M, et al. Development of the Risk Assessment Suicidality Scale (RASS): a population-based study. *J Affect Disord.* 2012;138(3):449-457.

5. MacDonald PL, Gardner RC. Type I Error Rate Comparisons of Post Hoc Procedures for I j Chi-Square Tables. *Educational and Psychological Measurement.* 2016;60(5):735-754.

6. Fountoulakis KN, Apostolidou MK, Atsiova MB, et al. Self-reported changes in anxiety, depression and suicidality during the COVID-19 lockdown in Greece. *J Affect Disord.* 2021;279:624-629.

7. Foster C. Do People Really Think Earth Might Be Flat? *Scientific American.* 2018;<https://blogs.scientificamerican.com/observations/do-people-really-think-earth-might-be-flat/>.
